# A randomised, double-blind, multicentre, placebo-controlled phase 3 efficacy, safety, and immunogenicity study of an Ad26.RSV.preF/RSV prefusion F-protein vaccine for the prevention of respiratory syncytial virus-mediated lower respiratory tract disease in adults aged 60 years and older

**DOI:** 10.1016/j.ebiom.2026.106513

**Published:** 2026-11-04

**Authors:** Christy A. Comeaux, Marco Testa, Efi Gymnopoulou, Arangassery Rosemary Bastian, Mauricio L. Nogueira, Hongxing Pan, Fengcai Zhu, Viktoriia Fursenko, William B. Smith, Patricia Junquera, Joris Menten, Matteo Loi, Edem Bassey Effiom, Archana M. Jastorff, Karin Weber, Ann R. Falsey, Benoit Callendret, Esther Heijnen

**Affiliations:** a Johnson & Johnson, Leiden, the Netherlands; b Janssen-Cilag, Cologno Monzese, Italy; c Johnson & Johnson, Beerse, Belgium; d Faculdade de Medicina de São José do Rio Preto, São José do Rio Preto, São Paulo, Brazil; e Jiangsu Provincial Center for Disease Control and Prevention, Nanjing, Jiangsu Sheng, China; f IQVIA Poland, Warsaw, Poland; g AMR Clinical, Knoxville, TN, USA; h Prestige Clinical Research Center, Inc., Coral Gables, FL, USA; i Johnson & Johnson, IDV, Vienna, Austria; j University of Rochester School of Medicine, Rochester, NY, USA

**Keywords:** Respiratory syncytial virus, Ad26.RSV.preF/RSV preF protein, Lower respiratory tract disease, Older adults

## Abstract

**Background:**

Respiratory syncytial virus (RSV) is a leading cause of lower respiratory tract disease (LRTD) in older adults. This study evaluated efficacy, immunogenicity, and safety of the investigational recombinant, replication-incompetent, adenovirus type 26 vector RSV preF protein (Ad26.RSV.preF/RSV preF vaccine).

**Methods:**

EVERGREEN was a randomised, double-blind, placebo-controlled phase 3 trial conducted in 14 countries. Adults aged 60 years or older (stratified by age and risk of severe RSV disease) were randomly assigned 2:1 using an interactive web response system to receive one dose of vaccine or placebo. Participants, study site personnel, investigators, and sponsor were blinded to vaccine allocation. The primary endpoint was the first occurrence of RSV-mediated LRTD, defined as new onset/worsening of at least three symptoms occurring 14 or more days postvaccination. The study had at least 90% power to demonstrate statistically significant vaccine efficacy greater than 20% against the primary endpoint and to achieve a point estimate for vaccine efficacy of more than 50%. The trial is registered at ClinicalTrials.gov, NCT04908683. The study is completed.

**Findings:**

The study was conducted between 21 July 2021 and 20 July 2023. At the time of the primary analysis, 26,299 individuals were screened, 23,707 were enrolled and 23,555 were vaccinated, 15,698 (67%) in the active vaccine group and 7857 (33%) in the placebo group. In all 23,555 vaccinated participants, 13,515 (54%) were female, 11,558 (46%) were male and the mean age was 70.2 years (SD 5.24). In the per protocol efficacy set, 17 of 15,337 participants (0.1%) in the vaccine group and 31 of 7683 participants (0.4%) in the placebo group had an RSV-mediated LRTD event. Vaccine efficacy was 72.7% (96.326% confidence interval, 47.2–86.4) for prevention of RSV-mediated LRTD over a mean follow-up time of 233.9 days (database cutoff: 15 September 2022). Solicited local and systemic adverse events were more frequent with vaccine versus placebo; none were grade four. The frequency of serious adverse events was similar in the vaccine (n = 842, 5.0%) and placebo (n = 457, 5.5%) groups, of which 15 (0.1%) and two (<0.1%), respectively, were considered vaccine related. There were 105 (0.63%) deaths in the vaccine group and 61 (0.73%) in the placebo group. None were considered related to study vaccine by the investigator. The study and vaccine development were discontinued in March 2023 due to a strategic decision by the study sponsor.

**Interpretation:**

In adults aged 60 years and older, the Ad26.RSV.preF/RSV preF protein vaccine was efficacious in preventing RSV-mediated LRTD and had an acceptable safety profile.

**Funding:**

Johnson & Johnson.

**Research in context**

**Evidence before this study**

We conducted a systematic literature search of PubMed since inception until 17 October 2025, for publications reporting results of clinical trials of the Ad26.RSV.preF/RSV preF protein vaccine. We used the search term “Ad26.RSV.preF/RSV preF” using the randomised controlled trial filter and identified eight manuscripts describing clinical trial results, two were dose- or regimen-selection studies, three were immunogenicity and safety studies, and three were efficacy studies. In the key Phase 2b CYPRESS study, the efficacy of Ad26.RSV.preF/RSV preF protein vaccine against respiratory syncytial virus-mediated lower respiratory tract disease in adults aged 60 years and older, vaccine efficacy was 80.0% during the first season and 76.1% over seasons 2 and 3.

**Added value of this study**

Following successful demonstration of efficacy in phase 2, we initiated a phase 3 confirmatory efficacy study to further delineate the efficacy of a single dose of Ad26.RSV.preF/RSV preF protein vaccine for the prevention of lower respiratory tract disease caused by RSV in older adults for a follow-up of at least 2 years. The primary endpoint was based on the case definition that was demonstrated to be most clinically relevant in phase 2 encompassing both moderate and severe forms of RSV LRTD. Vaccine efficacy of 72.7% is consistent with previous studies of this vaccine.

**Implications of all the available evidence**

The results of this large Phase 3 efficacy study together with previous phase 2 and 3 studies confirm that a single dose of Ad26.RSV.preF/RSV preF protein vaccine is effective in preventing RSV-mediated LRTD in adults 60 years and older and provides durable protection over at least three seasons. The vaccine safety profile is acceptable, and the benefit risk ratio appears positive. Nevertheless, the study was terminated early by the sponsor due to strategic reprioritization reasons. All participants were followed up for 6 months post-vaccination for safety data collection.

## Introduction

Respiratory syncytial virus (RSV) poses a substantial global health burden across all age groups, with a global age-standardised mortality rate of 4.79 out of 100,000 reported in 2019.^1^ In older adults, RSV can cause serious respiratory infection,^2^^,^^3^ with relatively high annual case numbers, hospitalisations, and deaths.^4^ Despite this burden, there are no pathogen-specific therapeutics approved for this population. Only recently have vaccines been approved following reports demonstrating the safety and efficacy of two RSV preF (conformation-stabilised RSV preF protein)-based vaccines and an mRNA-based RSV vaccine (encoding the conformation-stabilised RSV preF protein) for prevention of RSV lower respiratory tract disease (LRTD) in older adults.^5^, ^6^, ^7^, ^8^, ^9^, ^10^

In a phase 2b proof-of-concept study, a combination vaccine consisting of Ad26.RSV.preF (a recombinant, replication-incompetent, adenovirus type 26 vector encoding the RSV preF protein) and recombinant RSV preF protein demonstrated 80.0% vaccine efficacy for prevention of RSV-mediated LRTD after the first RSV season.^11^ Additionally, the vaccine induced RSV-specific humoural and cellular immune responses, with an acceptable safety profile, in adults aged ≥65 years. Here, we report the results of the primary and final analysis of the phase 3 study, EVERGREEN (NCT04908683). EVERGREEN aimed to further evaluate the efficacy, safety, and immunogenicity of Ad26.RSV.preF/RSV preF protein for prevention of RSV-mediated LRTD in adults aged ≥60 years.

## Methods

### Study design

EVERGREEN was a randomised, double-blind, placebo-controlled trial conducted at 249 centres in 14 countries (Australia, Brazil, Canada, Chile, China, Estonia, Finland, New Zealand, Poland, South Africa, Taiwan, Thailand, the United Kingdom, and the United States). The first participant was screened on 21 July 2021 and the last participant visit was on 20 July 2023. The primary objective was to demonstrate efficacy of Ad26.RSV.preF/RSV preF protein vaccine in the prevention of reverse transcriptase polymerase chain reaction (RT-PCR)-confirmed RSV-mediated LRTD when compared to placebo in adults aged 60 years and above. Interim analyses were conducted by the Independent Data Monitoring Committee (IDMC) using unblinded data to determine whether the pre-defined success criteria had been met, namely that the lower limit of the adjusted 95% confidence interval (CI) for vaccine efficacy exceeded 20% and the point estimate exceeded 50%. If these criteria were met at an interim analysis, the study transitioned to the primary analysis by the sponsor using the corresponding database cut-off. If not, follow-up and endpoint accrual continued until the next planned analysis or final primary analysis. Across analyses, statistical inference accounted for the pre-specified alpha spending-approach to preserve the overall type I error rate.

The trial was prospectively registered at ClinicalTrials.gov (NCT04908683) on 27 May 2021.

### Ethics statement and study oversight

The final trial protocol and amendments were approved by an independent ethics committee or institutional review board at participating sites (see Supplementary Material 1). The trial was conducted in accordance with the International Council of Harmonisation guidelines on Good Clinical Practice and the Declaration of Helsinki. The manuscript adheres to the 2024 Declaration of Helsinki ethical principles. All participants provided written informed consent. Safety endpoints were monitored by an IDMC (see Supplementary Material 3) consisting of at least one medical expert and at least one statistician.

### Participants

Adults 60 years of age or older in stable health were eligible to participate. Individuals with underlying illnesses, including hypertension, congestive heart failure, chronic obstructive pulmonary disease, type 2 diabetes, hyperlipoproteinaemia, or hypothyroidism, were eligible if symptoms were stable and medically controlled according to investigator judgement at vaccination. Participants with underlying chronic heart disease (congestive heart failure, coronary artery disease [e.g., angina pectoris, ischaemic cardiomyopathy, history of myocardial infarct, history of coronary artery bypass graft, or coronary artery stent]) and chronic lung diseases (e.g., asthma or chronic obstructive pulmonary disease) were considered at increased risk of severe RSV disease. No participants were hospitalised or institutionalised at enrolment. Additional eligibility information is provided in the protocol (Supplementary Material 1).

### Randomisation and masking

Participants were randomly assigned in a 2:1 ratio (stratified by age [60–64 years, 65–74 years, 75–84 years, equal to or greater than 85 years]) and risk level [increased or no increased risk of severe RSV disease] to Ad26.RSV.preF/RSV preF protein vaccine or placebo on Day 1. The vaccination window for the primary analysis was from 21 July 2021 until 31 August 2022.

Randomisation was based on a computer-generated randomisation algorithm prepared before the study by or under the supervision of the sponsor. Participants were allocated a unique code by an interactive web response system, which dictated study group assignment. In addition to stratification by age and risk level, within the strata, minimisation was applied for each study subset (being part of the safety subset only, the immunogenicity subset only, both subsets, or none), region (North America, Europe, Southern Hemisphere), site, and stratum combination. Participants, study site personnel, investigators, and sponsor were blinded to vaccine allocation. An unblinded pharmacist or qualified individual with no other study role, was responsible for the preparation and labelling of syringes, providing the Ad26/protein preF RSV vaccine and placebo in a blinded manner to the study vaccine administrator for injection.

The participants, study site personnel, and investigator remained blinded until the end of the study. Participants remained blinded as to the outcome of RSV reverse transcription polymerase chain reaction test results until study unblinding.

### Procedures

The Ad26.RSV.preF/RSV preF protein vaccine was prepared prior to administration by mixing the two components (the Ad26.RSV.preF and RSV preF protein components) together. The Ad26.RSV.preF vector expresses the full-length codon optimised F gene from the RSV A2 strain, using amino acid substitutions to stabilise it in its prefusion conformation. The RSV preF protein is a trimeric soluble RSV F protein derived from the RSV A2 strain and stabilised in the prefusion conformation. It was produced in Chinese Hamster Ovary cells transfected with DNA constructs using electroporation. Vaccine and placebo (0.9% saline) were administered as a single intramuscular injection (1 ml) into the deltoid muscle on Day 1. One vaccine dose contained 1 × 10^11^ viral particles of Ad26.RSV.preF with 150 μg RSV preF protein.

After study vaccinations, twice-weekly acute respiratory infection (ARI) surveillance was conducted via electronic survey. The cutoff for ARI onset was 1 September 2022 and the cutoff for ARI data to be included in the primary analysis was 15 September 2022. Participants who experienced new onset or worsening ARI symptoms (nasal congestion, sore throat, cough, short of breath, coughing up phlegm [sputum], and wheezing) completed a daily electronic diary beginning on ARI Day 1 (day of onset), which included the RSV-specific Respiratory Infection Intensity and Impact Questionnaire (RiiQ™)^12^ and the Patient Global Impression Return to Usual Health questionnaire,^13^ until symptoms resolved or returned to baseline (status on study Day 1) for at least 2 days. On ARI Day 1 or 2, participants collected a nasal swab at home. On ARI Days 3–5, a nasal swab, sputum sample (when possible), and blood sample were collected during clinical visits. To confirm RSV infection, nasal (analysed locally) and sputum (analysed centrally) samples underwent RT-PCR as described in Supplementary Material 1.

### Outcomes

The primary endpoint was first occurrence of RT-PCR–confirmed RSV-mediated LRTD, defined as new onset/worsening of at least three symptoms (cough, short of breath, coughing up phlegm, and wheezing, as collected by the RiiQ™) at least 14 days after vaccination, as assessed by a clinical evaluation committee consisting of independent medical experts in the relevant therapeutic area. Efficacy analyses were performed in the per protocol efficacy (PPE) population, as well as in age-based subgroups and participants at increased risk for severe RSV disease.

Secondary confirmatory endpoints were the first occurrence of confirmed RSV-mediated ARI (new onset/worsening of at least one symptom) with onset at least 4 days after vaccination; first occurrence of RT-PCR-confirmed RSV-mediated LRTD and ARI during the second year, with onset after study Day 365; and first occurrence of predefined clinically relevant disease associated with RT-PCR-confirmed RSV-mediated ARI over the whole study with onset at least 14 days after dosing of study vaccine. Assessment of immunogenicity, safety, and changes in the area under the curve for symptom scores in participants with an RT-PCR-confirmed RSV-mediated ARI over the whole study were secondary non-confirmatory endpoints.

Due to early study termination, a limited selection of exploratory efficacy endpoints was analysed. These were assessment of vaccine efficacy by study year and RSV strain. Exploration of the correlation between immune responses and risk of RSV infection was conducted in a paired analysis with another study and will be published separately.

Blood samples were collected on Days 1 (pre-vaccination) and 15 for immunogenicity assessments, including those in the immunogenicity subset. RSV A2 neutralising antibodies were quantified using a virus neutralising assay. RSV preF A- and preF B-specific immunoglobulin G (IgG) antibodies were measured by enzyme-linked immunosorbent assay. RSV B neutralising antibodies were not assessed by the data cutoff date. Assessment of RSV-F–specific T-cell responses was accomplished using interferon-γ enzyme-linked immune absorbent spot. Assay details are in Supplementary Material 1.

Solicited local and systemic adverse events (AEs) and unsolicited AEs were recorded in a safety subset for 7 and 28 days postvaccination, respectively. AE severity was graded using a modified version of the US Food and Drug Administration Toxicity Grading Scale for Healthy Adult and Adolescent Volunteers Enrolled in Preventive Vaccine Clinical Trials (September 2007).^14^

Serious AEs (SAEs) and potential AEs of special interest (AESIs; thrombosis and thrombocytopenia) were collected from Day 1 until 6 months postvaccination in the full analysis set (FAS). AEs leading to study discontinuation were recorded from Day 1 until the end of the study. Changes in procedures for capturing, recording and reporting AESIs during the study are provided in Supplementary Material 1. Thrombosis with thrombocytopenia syndrome is a rare SAE occurring after vaccination with adenovirus vectored COVID-19 vaccines.^15^ Potential AESIs with concurrent thrombosis and thrombocytopenia qualified for assessment and further evaluation by a thrombosis with thrombocytopenia syndrome assessment committee consisting of two independent external experts. Cases were evaluated against three case definitions (Brighton Collaboration, United States Centres for Disease Control and European Medicine’s Agency’s Pharmacovigilance Risk Assessment Committee).^16^, ^17^, ^18^

### Statistical analysis

The primary and final statistical analyses were performed by the study sponsor using Statistical Analysis System (SAS; Cary, NC, USA) software version 9.4 and R software version 4.1.2 (The R Foundation for Statistical Computing, Vienna, Austria). Amendments made to the statistical analysis plan are outlined in Supplementary Material 2. The data cut-off dates were 15 September 2022 for the analysis of the primary efficacy endpoint, and 20 July 2023 for the final analysis of secondary and exploratory endpoints.

The study and vaccine development were discontinued in March 2023 due to a strategic decision by the study sponsor. At that time, a majority of participants (93%, n = 24,415) had completed one study year and 15,042 (65%) were included in the analysis of the second study year. The early study termination did not compromise analysis of the primary and secondary endpoints as sufficient events were accrued; but provided limited ability to assess exploratory endpoints during the truncated second RSV season (mean 136 days of follow-up). All participants had safety data collected for a minimum of 6 months follow-up.

The PPE population included all randomised and vaccinated participants, excluding those with major protocol deviations expected to impact efficacy outcomes. If a major protocol deviation occurred, data from that year (i.e., the first or second year) and subsequent years were excluded. Participants with RT-PCR–confirmed RSV-mediated ARI onset within 14 days after vaccination, participants who discontinued the study within 14 days after vaccination, and participants who received vaccination within 14 days before the database cutoff date were excluded from the PPE population.

The safety population was the FAS that included all vaccinated participants, regardless of the occurrence of protocol deviations; analyses of solicited and unsolicited AEs were restricted to the safety subset. The per protocol immunogenicity subset was the primary immunogenicity analysis population and included all randomised and vaccinated participants with available immunogenicity data. Samples were excluded from the per protocol immunogenicity subset from participants with a major protocol deviation that was anticipated to impact immunogenicity outcomes or after an RSV infection reported during the study.

Primary efficacy endpoint analysis evaluated first occurrence of RT-PCR–confirmed RSV-mediated LRTD with onset of at least 14 days after vaccination in the active vaccine group compared with the placebo group in the PPE population. The null hypothesis (vaccine efficacy less than or equal to 20%) was tested versus the alternative hypothesis (vaccine efficacy >20%). In addition, an exact Poisson regression was fitted with event rate (i.e., the number of cases over the follow-up time) as the dependent variable and the vaccination group and stratification factors as independent variables. Vaccine efficacy against the primary endpoint was tested using a one-sided significance level of 2.5%, corrected for multiplicity (=α′). Overall, if the lower limit of the exact two-sided CI (1–2 × α′) for vaccine efficacy ([1–incidence rate ratio] × 100) calculated from the exact Poisson regression model was greater than 20%, and the observed vaccine efficacy point estimate was greater 50%, vaccine efficacy was considered to have been demonstrated for the primary endpoint. The multiple imputation model was used to evaluate the impact of missing RT-PCR results on the primary endpoint (Supplementary Material 2). Efficacy against RT-PCR–confirmed RSV ARIs was included in the testing strategy and analysed using the same α′ as the primary endpoint; vaccine efficacy for this endpoint was demonstrated if the lower limit of the CI was equal to or greater than 0%. For details regarding sensitivity analyses, see Supplementary Material 2.

The IDMC could evaluate vaccine efficacy for the primary endpoint against success criteria at four prespecified timepoints. At the first prespecified interim analysis timepoint (cutoff date: 15 September 2022), the IDMC indicated that the success criteria had been met. To account for multiple interim analyses, the total alpha was controlled at 2.5% using a Pocock-like alpha spending function^19^ with a target number of 76 events to spend the alpha over the different analyses. Using this approach, the 95% CI was corrected for multiplicity, resulting in a 96.326% CI for vaccine efficacy. No additional bias adjustments of effect point estimates were planned.

For participants with RSV ARIs, the area under the curve (AUC) of change from baseline in RiiQ™ total symptom scores was calculated. The time to return to usual health for participants with RSV ARIs was determined by Kaplan–Meier analysis, and a hazard ratio was calculated using a Cox proportional hazard model.

### Sample size estimation

The sample size was determined assuming a vaccine efficacy (primary endpoint) of 65% during the first and second year, an incidence of RT-PCR confirmed RSV-mediated LRTD of at least 0.2% during the first (2021–2022) and 0.5% in later RSV seasons in placebo recipients, an exclusion rate of 10%, and a one-sided α of 2.5% (potentially corrected for multiplicity). Approximately 15,340 participants in the vaccine group and 7670 participants in the placebo group were required to provide 90% power to demonstrate vaccine efficacy (total sample size, approximately 23,000 participants). Additional methods are provided in Supplementary Material 2.

The percentage risk difference with Wilson 95% CIs was calculated in a *post hoc* exploratory analysis of safety. This analysis was not pre-specified in the protocol or statistical analysis plan, and the study was not powered for these comparisons. No claims of statistical significance should be made for these exploratory analyses.

### Role of the funding source

This work was supported by Johnson & Johnson during all stages of the trial, including its analysis and the development and publishing of the manuscript, including scientific writing assistance and statistical analyses.

## Results

### Participants

In total, 26,299 participants were screened and 23,555 were included in the FAS for the primary analysis (Fig. 1). There were 23,020 participants included in the PPE population, of whom 15,337 participants received the vaccine and 7683 received placebo. For approximately two-thirds of participants, the follow-up period spanned one typical RSV season in the North and Southern hemisphere. Demographics and baseline characteristics were similar between vaccine and placebo recipients in the PPE population (Table 1). Median participant age was 69 years (2033 were ages 60–64 years: 8.8%; 16,642 were ages 65–74 years: 72.3%; 2862 were ages 75–84 years: 17.8%; 250 were ages ≥85 years: 1.1%). Risk factors for severe RSV disease were present in 3737 (24.4%) of participants in the vaccine group and 1935 (25.2%) in the placebo group.

**Fig. 1:**
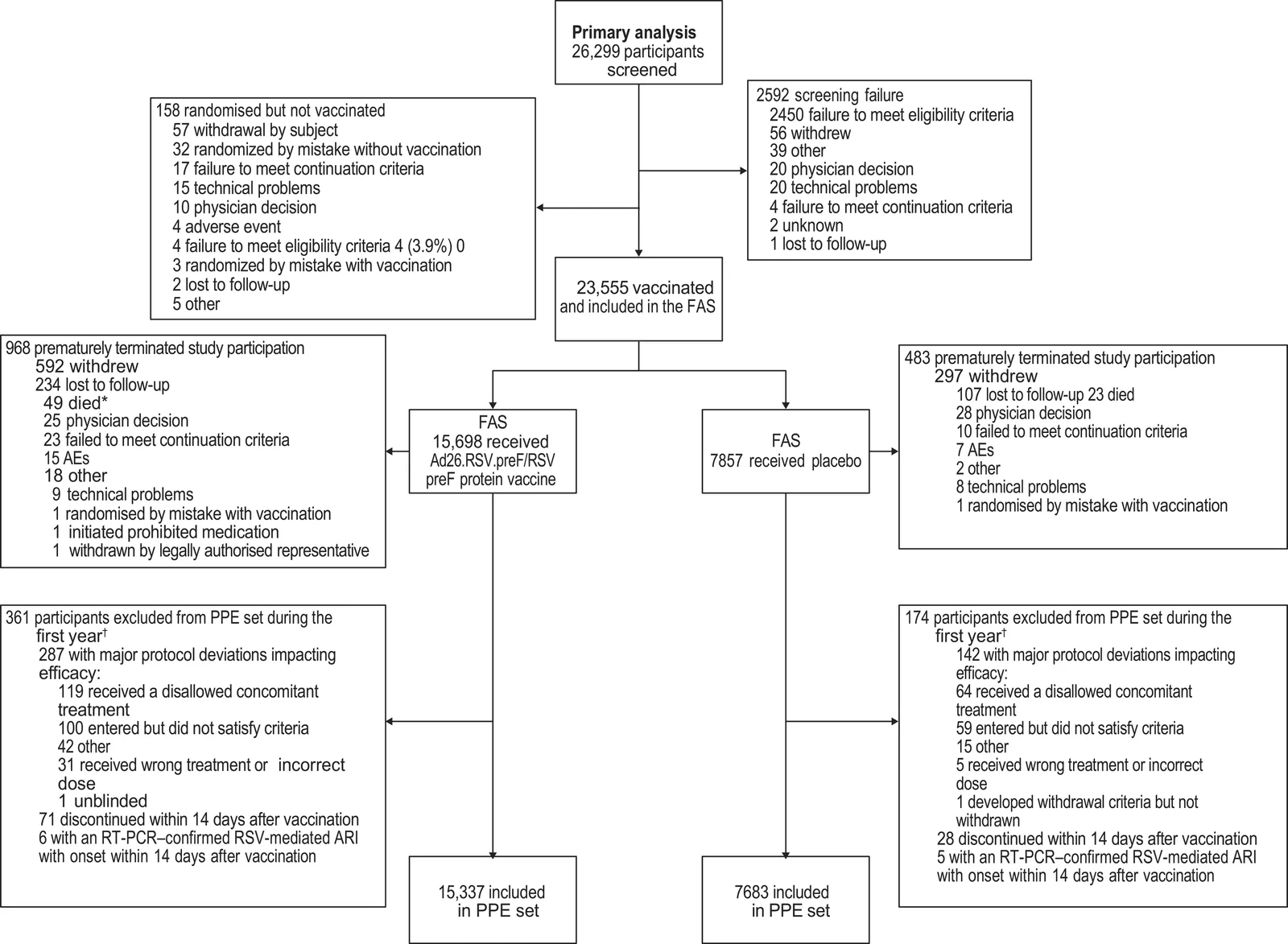

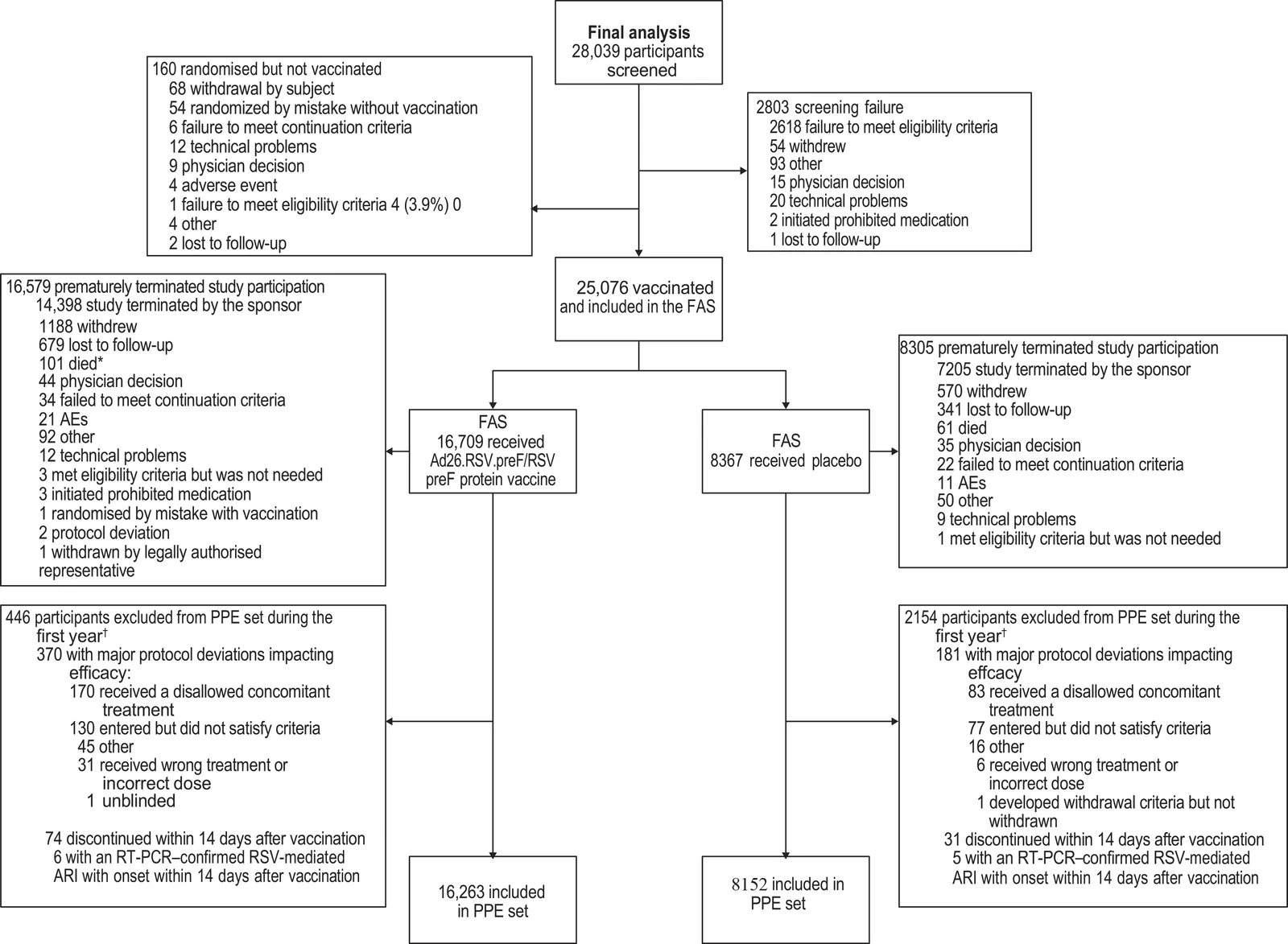
**Participant disposition.** Ad26, adenovirus type 26; AEs, adverse events; ARI, acute respiratory infection; FAS, full analysis set; PPE, per protocol efficacy; preF, prefusion conformation-stabilised RSV F protein; RSV, respiratory syncytial virus; RT-PCR, reverse transcription polymerase chain reaction. ∗One participant discontinued study participation due to an AE and died 4 days later. ^†^Participants may appear in more than one category.

**Table 1:** Baseline characteristics of the trial participants in the per protocol efficacy population for the primary analysis.

	Ad26.RSV.preF/RSV preF protein (n = 15,337)	Placebo (n = 7683)
Age, median (Q1, Q3), years	69 (66, 73)	69 (66, 73)
Age category, n (%)		
60–64	1356 (8.8)	677 (8.8)
65–74	11,089 (72.3)	5553 (72.3)
75–84	2726 (17.8)	1369 (17.8)
≥85	166 (1.1)	84 (1.1)
Sex,^a^ n (%)		
Female	8461 (55.2)	4144 (53.9)
Male	6875 (44.8)	3537 (46.0)
Unknown	1 (<0.1)	2 (<0.1)
Region, n (%)		
North America	10,595 (69.1)	5288 (68.8)
Europe	2117 (13.8)	1067 (13.9)
Africa	1244 (8.1)	629 (8.2)
Asia	640 (4.2)	324 (4.2)
Australia/New Zealand	462 (3.0)	233 (3.0)
South America	279 (1.8)	142 (1.8)
Country, n (%)		
Australia	23 (0.1)	12 (0.2)
Brazil	116 (0.8)	60 (0.8)
Canada	547 (3.6)	259 (3.4)
Chile	163 (1.1)	82 (1.1)
China	424 (2.8)	213 (2.8)
Estonia	160 (1.0)	71 (0.9)
Finland	35 (0.2)	20 (0.3)
New Zealand	439 (2.9)	221 (2.9)
Poland	1006 (6.6)	513 (6.7)
Taiwan	11 (0.1)	6 (0.1)
Thailand	205 (1.3)	105 (1.4)
United States	10,048 (65.5)	5029 (65.5)
United Kingdom	916 (6.0)	463 (6.0)
South Africa	1244 (8.1)	629 (8.2)
Race,^a^ n	15,697	7857
White, n (%)	12,336 (80.4)	6147 (80.0)
Black or African American, n (%)	1780 (11.6)	917 (11.9)
Asian, n (%)	744 (4.9)	385 (5.0)
Multiple, n (%)	187 (1.2)	99 (1.3)
Native Hawaiian or other Pacific Islander, n (%)	97 (0.6)	48 (0.6)
Not reported, n (%)	84 (0.5)	46 (0.6)
American Indian or Alaska Native, n (%)	56 (0.4)	17 (0.2)
Unknown, n (%)	52 (0.3)	25 (0.3)
Ethnicity, n (%)		
Not Hispanic or Latino	13,570 (88.5)	6929 (88.9)
Hispanic or Latino	1526 (9.9)	728 (9.5)
Not reported	197 (1.3)	93 (1.2)
Unknown	44 (0.3)	33 (0.4)
Smoking status, n	15,697	7857
Smoker, n (%)	6514 (42.5)	3231 (42.1)
Non-smoker, n (%)	8822 (57.5)	4452 (57.9)
At increased risk of severe RSV disease, n (%)		
No increased risk	11,600 (75.6)	5748 (74.8)
Increased risk^b^	3837 (24.4)	1935 (25.2)
Asthma	1446 (9.4)	755 (9.8)
COPD	967 (6.3)	477 (6.2)
CHF	282 (1.8)	152 (2.0)
Other chronic heart diseases	1535 (10.0)	768 (10.0)
Other chronic lung diseases^c^	227 (1.5)	111 (1.4)

Ad26, adenovirus type 26; CHF, congestive heart failure; COPD, chronic obstructive pulmonary disease; preF, prefusion conformation-stabilised RSV F protein; Q, quartile; RSV, respiratory syncytial virus.

a Sex and race were self-reported.

b Participants at increased risk of severe RSV disease were defined as those with underlying chronic heart disease (CHF, coronary artery disease [e.g., angina pectoris, ischaemic cardiomyopathy, history of myocardial infarction, history of coronary artery bypass graft, or coronary artery stent]) and chronic lung diseases (e.g., asthma and chronic COPD).

c The electronic case report form included a category labelled “other chronic lung diseases” that could be chosen at the discretion of the investigator.

After the primary analysis, a further 1521 participants were enrolled, providing 25,076 participants for the final analysis (Fig. 1). The PPE population resembled the FAS in terms of demographic characteristics (Supplementary Table S1).

### Vaccine efficacy

The primary endpoint was met and vaccine efficacy for prevention of RSV-mediated LRTD was demonstrated. In the PPE population, RSV-mediated LRTD occurred in 17 (0.1%) participants receiving study vaccine and 31 (0.4%) receiving placebo. Vaccine efficacy for prevention of RSV-mediated LRTD was 72.7% (96.326% CI, 47.2–86.4; Fig. 2). Sensitivity analyses including analysis of the primary endpoint in the FAS population, were consistent with the primary analysis (Supplementary Fig. S1). Vaccine efficacy based on the multiple imputation model was 67.0% (96.326% CI, 38.1–82.4), which accounted for 13% of missing RT-PCR results in the PPE. RSV-mediated ARIs were reported in 32 (0.2%) and 49 (0.6%) participants from the vaccine and placebo groups, respectively. Approximately 81% of participants in both the vaccine (n = 12,458) and placebo groups (n = 6275) maintained ≥90% ARI surveillance compliance. Vaccine efficacy for prevention of RSV ARIs was 67.5% (96.326% CI, 46.7–80.5); therefore, the first secondary confirmatory endpoint was met and vaccine efficacy against RSV ARI was demonstrated. Vaccine efficacy for both LRTD (Fig. 3 A-C) and ARI (Fig. 4 A-C) was maintained in participant subgroups by age and risk level, although was less pronounced for the RSV subtype A.

**Fig. 2:**
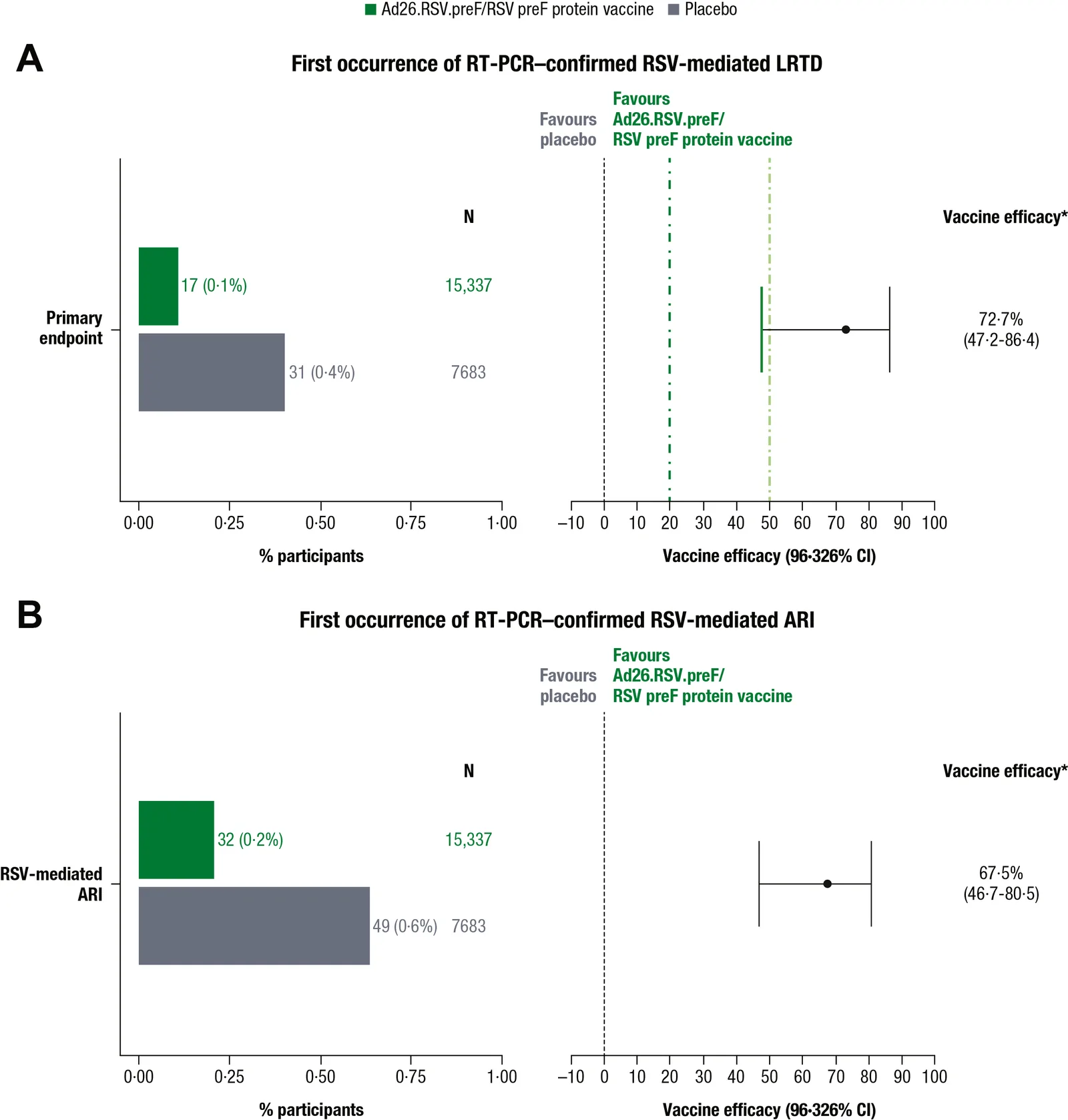
**(A) Efficacy of Ad26.RSV.preF/RSV preF protein vaccine for prevention of RT-PCR–confirmed RSV-mediated LRTD. (B) Efficacy of Ad26.RSV.preF/RSV preF protein vaccine for prevention of RT-PCR–confirmed RSV-mediated ARI.** Success criteria specified the lower limit of the CI corrected for multiplicity of the vaccine efficacy must be >20% (dark green dotted line) and the point estimate must be >50% (light-green dotted line). Ad26, adenovirus type 26; ARI, acute respiratory infection; CI, confidence interval; LRTD, lower respiratory tract disease; preF, prefusion conformation-stabilised RSV F protein; RT-PCR, reverse transcription polymerase chain reaction; RSV, respiratory syncytial virus. ∗Vaccine efficacy was calculated with an exact Poisson regression model, including vaccination group and stratification variables as covariates. Vaccine efficacy is a 95% CI corrected for multiplicity using a Pocock-like alpha spending function with a target number of events of 76 to spend the alpha over the different analyses.

**Fig. 3:**
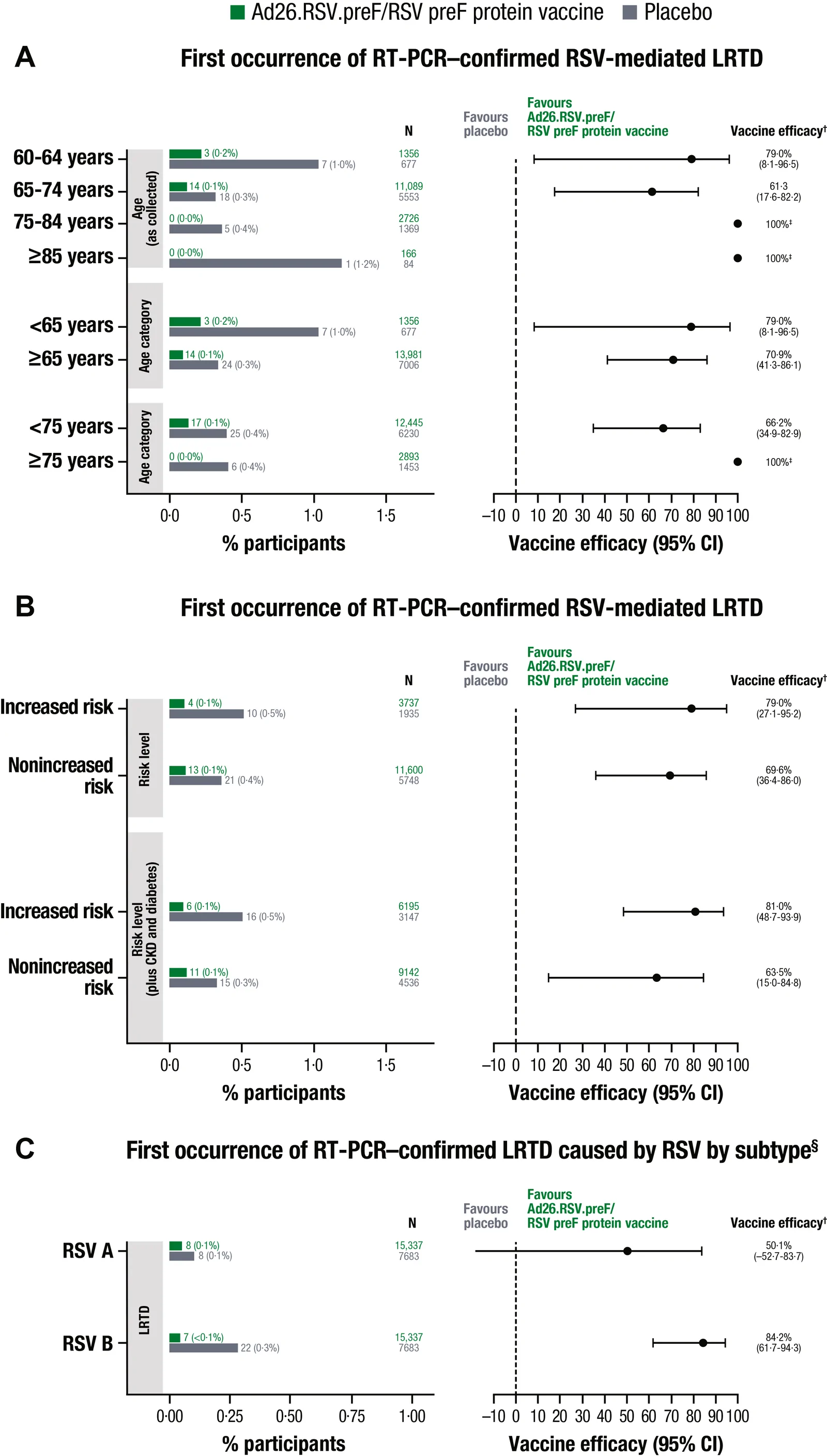
**Efficacy of Ad26.RSV.preF/RSV preF protein vaccine for prevention of RT-PCR–confirmed RSV-mediated LRTD according to age (A), risk∗ (B), and RSV subtype (C).** Ad26, adenovirus type 26; CHF, congestive heart failure; CI, confidence interval; CKD, chronic kidney disease; COPD, chronic obstructive pulmonary disease; LRTD, lower respiratory tract disease; PPE, per protocol efficacy; preF, prefusion conformation-stabilised RSV F protein; RSV, respiratory syncytial virus; RT-PCR, reverse transcription polymerase chain reaction. ∗Participants were considered at increased risk for severe RSV-mediated LRTD if they had underlying chronic heart disease (CHF, coronary artery disease [e.g., angina pectoris, ischaemic cardiomyopathy, history of myocardial infarction, history of coronary artery bypass graft, or coronary artery stent]) or chronic lung disease (asthma and COPD). ^†^Vaccine efficacy was calculated using an exact Poisson regression model, including vaccination group and stratification variable as covariates. ^‡^Exact CIs could not be calculated by SAS. ^§^In the PPE population, subtyping was missing for eight participants (5 Ad26.RSV.preF/RSV preF protein vaccine; 3 placebo). Two participants were coinfected with RSV A and B.

**Fig. 4:**
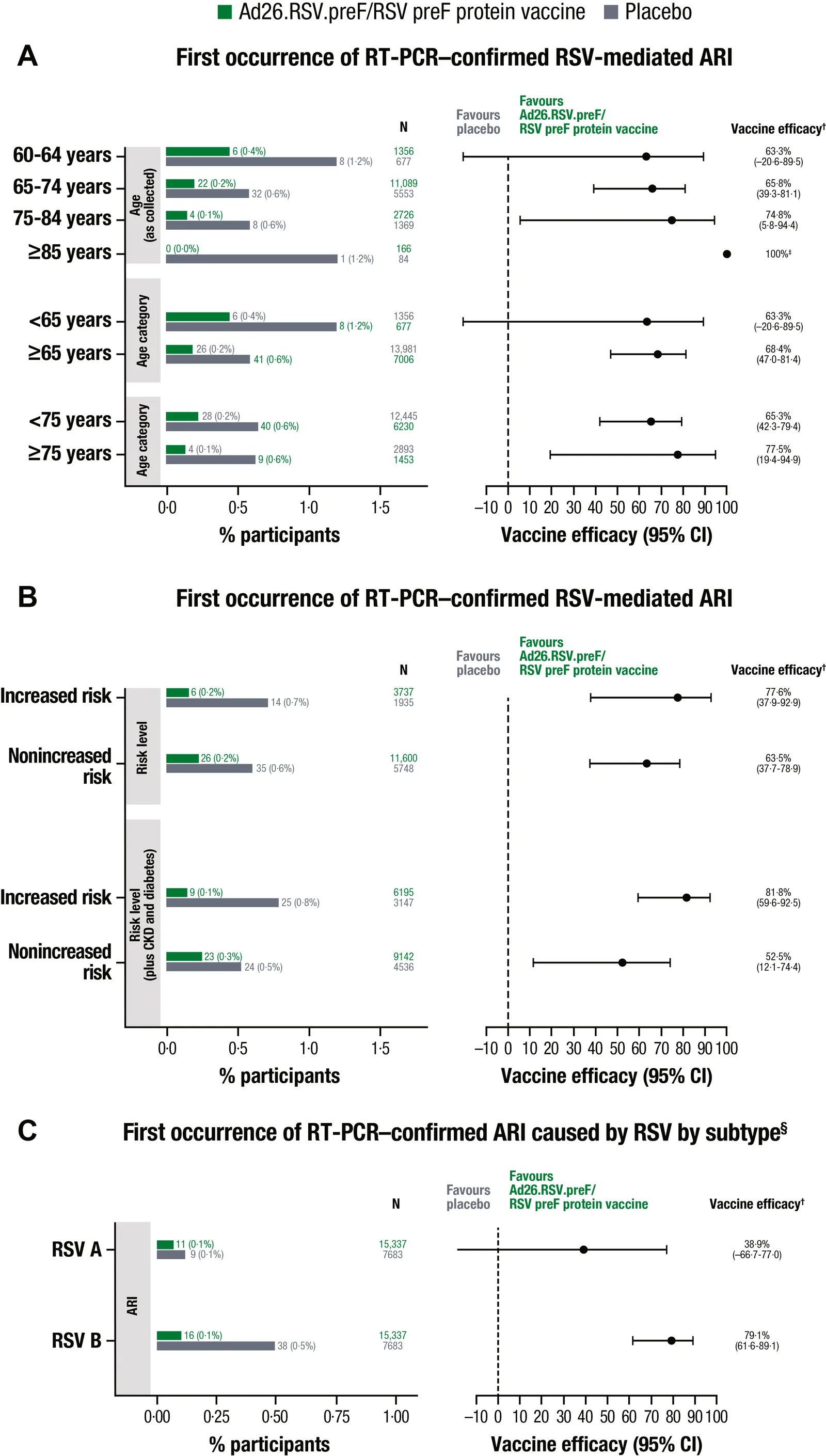
**Efficacy of Ad26.RSV.preF/RSV preF protein vaccine for prevention of RT-PCR–confirmed RSV-mediated ARI according to age (A), risk∗ (B), and RSV subtype (C).** Ad26, adenovirus type 26; ARI, acute respiratory infection; CHF, congestive heart failure; CI, confidence interval; CKD, chronic kidney disease; COPD, chronic obstructive pulmonary disease; PPE, per protocol efficacy; preF, prefusion conformation-stabilised RSV F protein; RSV, respiratory syncytial virus; RT-PCR, reverse transcription polymerase chain reaction. ∗Participants were considered at increased risk for severe RSV-mediated ARI if they had underlying chronic heart disease (CHF, coronary artery disease [e.g., angina pectoris, ischaemic cardiomyopathy, history of myocardial infarction, history of coronary artery bypass graft, or coronary artery stent]) or chronic lung disease (asthma and COPD). ^†^Vaccine efficacy was calculated using an exact Poisson regression model, including vaccination group and stratification variable as covariates. ^‡^Exact CIs could not be calculated by SAS. ^§^In the PPE population, subtyping was missing for 8 participants (5 Ad26.RSV.preF/RSV preF protein vaccine; 3 placebo). Two participants were coinfected with RSV A and B.

The remaining three secondary confirmatory efficacy endpoints were tested hierarchically at the final analysis. Efficacy of vaccination was demonstrated for all three secondary endpoints, as the lower limit of the 95% CI for each endpoint was greater than 0%. Vaccine efficacy was 72.3% (95% CI: 47.3%, 86.0%) against RT-PCR-confirmed RSV-mediated LRTD during the second year, 55.8% (95% CI: 30.4%, 72.0%) against RT-PCR-confirmed RSV-mediated ARI during the second year, and 81.8% (95% CI: 38.7%, 95.8%) against clinically relevant disease associated with RT-PCR-confirmed RSV-mediated ARI during the whole study (Supplementary Table S2).

RiiQ scores indicate disease severity, and a lower score indicates less severe disease. In participants with any RT-PCR-confirmed RSV-mediated ARI, the median area under the curve of the change from baseline for the RiiQ Total Symptom score was significantly lower in the vaccine group (median change from baseline 82 [first and third quartiles: 21, 141]) than in the placebo group (median change from baseline 107 [first and third quartiles: 48, 218]I) during the whole study (p-value 0.0010) (Supplementary Fig. S2).

The time to return to health was an exploratory endpoint and is presented here as potentially indicative of reduced disease severity. The median time to return to usual health after RSV LRTD was 12 days in the vaccine group versus 20 days in the placebo group (hazard ratio: 2.52 [95% CI, 1.27–4.99]). For those with RSV ARIs, the median time to return to usual health was 12 and 17 days in vaccine and placebo groups, respectively (hazard ratio: 1.49 [95% CI, 0.91–2.45]) (Supplementary Fig. S3).

The results of other exploratory endpoints are provided in Supplementary Figs. S4–S6 and Supplementary Tables S3–S7.

### Immunogenicity

The immunogenicity subset consisted of 325 participants (vaccine: n = 216; placebo: n = 109), most of whom were White (74.2%) and from North America (38.8%; Supplementary Table S8). Of these, 300 participants provided humoural immunogenicity data and 253 provided cellular immunogenicity data. In the vaccine group, geometric mean titres (GMTs) of RSV A2 neutralising antibodies increased 12.0-fold from baseline to Day 15; GMTs of RSV preF A and RSV preF B IgG serum antibodies each increased 11.3-fold (Supplementary Figs. S7–S9). Median RSV-F–specific T-cell frequency increased from below the lower limit of quantification at Day 1–533 spot-forming cells/10^6^ peripheral blood mononuclear cells at Day 15 in the vaccine group. No changes in immunogenicity outcomes were observed for participants who received placebo. Humoural and cellular immunogenicity was comparable across participant age and risk level groups (Supplementary Figs. S10–S16).

### Safety

In the safety subset (vaccine: n = 2305; placebo: n = 1155; Supplementary Table S9), solicited local AEs within 7 days post-vaccination were reported for 1076 (46.7%) and 169 (14.6%) participants in the vaccine and placebo groups, respectively; most were grade 1 or 2 (Table 2). The median time to onset across all solicited local AEs was 2–3 days, with a median duration of 2 days for participants who received the vaccine and 1 day for those who received placebo. The most common solicited local AE for both groups was injection-site pain/tenderness (vaccine: 1065 [46.2%]; placebo: 168 [14.5%]; Supplementary Fig. S17).

**Table 2:** Summary of adverse events (safety subset and FAS, final analysis).

	Ad26.RSV.preF/RSV preF protein	Placebo	Risk difference in % (Wilson 95% CI)^f^
	n (%)	n (%)	
Solicited AEs (safety subset, 7 days postvaccination):	**n = 2305**	**n = 1155**	
Participants with 1 or more:			
Solicited AE	1333 (57.8%)	307 (26.6%)	31.25 (26.60–35.72)
Solicited AE of worst grade 3	55 (2.4%)	8 (0.7%)	1.69 (0.48–2.74)
Solicited AE of worst grade 4	0	0	0.00 (−0.33 to 0.17)
Solicited local AE^a^	1076 (46.7%)	169 (14.6%)	32.05 (27.86–36.01)
Solicited local AE of worst grade 3	9 (0.4%)	4 (0.3%)	0.04 (−0.68 to 0.61)
Solicited local AE of worst grade 4	0	0	0.00 (−0.33 to 0.17)
Solicited systemic AE^b^	1018 (44.2%)	236 (20.4%)	23.73 (19.29–27.99)
Solicited systemic AE of worst grade 3	51 (2.2%)	5 (0.4%)	1.78 (0.68–2.71)
Solicited systemic AE of worst grade 4	0	0	0.00 (−0.33 to 0.17)
Solicited systemic AE thought to be related to study vaccine	986 (42.8%)	216 (18.7%)	24.08 (19.72–28.25)
Unsolicited AEs (safety subset, 28 days postvaccination):	**n = 2330**	**n = 1165**	
Participants with 1 or more:			
Unsolicited AE	554 (23.8%)	206 (17.7%)	6.09 (2.11–9.95)
Unsolicited AE of worst grade 3	109 (4.7%)	33 (2.8%)	1.85 (−0.06 to 3.59)
Unsolicited AE of worst grade 4	1 (<0.1%)	1 (0.1%)	−0.04 (−0.48 to 0.23)
Unsolicited AE thought to be related to study vaccine	342 (14.7%)	132 (11.3%)	3.35 (0.02–6.54)
SAEs, AESIs, and AEs leading to discontinuation (FAS, until database cut-off)	**n = 16,709**	**n = 8367**	
Participants with 1 or more:			
SAE	842 (5.0)	457 (5.5)	−0.42 (−1.25 to 0.39)
SAE thought to be related to study vaccine	15 (0.1)	2 (<0.1)	0.07 (−0.03 to 0.14)
Potential AESI (programmed definition)^c^	164 (1.0)	81 (1.0)	0.01 (−0.36 to 0.36)
Potential AESI (programmed definition) considered related by the investigator	11 (0.1)	1 (<0.1)	0.05 (−0.03 to 0.12)
Potential AESI identified by the investigator^d^	141 (0.8)	70 (0.8)	0.01 (−0.34 to 0.33)
Potential AESI identified by the investigator and considered related by the investigator	9 (0.1)	1 (<0.1)	0.04 (−0.04 to 0.10)
Potential AESI qualified for assessment	15 (0.1)	8 (0.1)	−0.01 (−0.13 to 0.10)
AE with fatal outcome	105 (0.6)	61 (0.7)	−0.10 (−0.42 to 0.19)
AE with fatal outcome thought to be related to study vaccine	0	0	0.00 (−0.05 to 0.02)
AEs leading to vaccine discontinuation^e^	24 (0.1)	12 (0.1)	0.00 (−0.15 to 0.13)
AE leading to study discontinuation	21 (0.1)	11 (0.1)	−0.01 (−0.15 to 0.12)

Ad26, adenovirus type 26; AE, adverse event; AESI, adverse event of special interest; FAS, full analysis set; preF, prefusion conformation-stabilised RSV F protein; RSV, respiratory syncytial virus; SAE, serious adverse event.

a Solicited local AEs included erythema, injection-site pain/tenderness, and swelling.

b Solicited systemic AEs included fatigue, headache, myalgia, nausea, and pyrexia.

c A retrospective case retrieval was performed to allow assessment of cases that occurred prior to protocol amendment 2 using standardised *Medical Dictionary for Regulatory Activities* (Version 24.1) queries that captured thrombotic and thrombocytopenic events; such events were described as potential AESIs by programmed definition.

d Prior to protocol amendment 2, symptoms, signs, and conditions indicating thrombosis and/or thrombocytopenia were prospectively reported as potential AESIs by investigators.

e Study vaccination discontinuation information was collected until the implementation of protocol amendment 4 (8 April 2022).

f Post hoc exploratory analysis. This analysis was not pre-specified in the protocol or statistical analysis plan and the study was not powered for these comparisons. No claims of statistical significance should be made for these exploratory analyses.

Solicited systemic AEs were reported by 1018 (44.2%) of participants in the vaccine group and 236 (20.4%) of those who received placebo, the most common of which were fatigue (vaccine: 789 [34.2%]; placebo: 165 [14.3%]), headache (vaccine: 597 [25.9%]; placebo 128 [11.1%]), and myalgia (vaccine: 614 [26.6%]; placebo:119 [10.3%]) (Supplementary Fig. S18). The median time to onset across all solicited systemic AEs was 2 days in the vaccine group and 2–4 days in the placebo group; the median duration was 1–2 days. No grade four solicited local or systemic AEs were reported.

Unsolicited AEs within 28 days post-vaccination were reported in 554 (23.8%) of vaccine recipients and 206 (17.7%) of placebo recipients. Unsolicited AEs reported by at least 5% of participants and considered related to study vaccination by the Investigators were increased systolic blood pressure (vaccine: 147 [6.3%]; placebo: 60 [5.2%]), and increased respiratory rate (vaccine: 89 [3.8%]; placebo: 47 [4.2%]). Unsolicited AEs of at least grade three were reported in 110 (4.7%) participants who received vaccine and 34 (2.9%) participants who received placebo (Supplementary Table S10). Of these, 86 (3.7%) and 26 (2.2%), respectively, were considered related to study vaccine.

Among the FAS, SAE frequency was similar between groups (n = 842, 5.0% versus n = 457, 5.5%, respectively; Table 2) until database cut-off. Of these, 15 SAEs (0.1%) in the vaccine group and two SAEs (<0.1%) in the placebo group were considered related to study vaccine. SAEs reported in at least 30 study participants in either group were pneumonia (vaccine: 46 [0.3%]; placebo: 26 [0.3%]), and chronic obstructive pulmonary disease (vaccine: 31 [0.2%]; placebo: 14 [0.2%]).

AEs leading to study discontinuation (vaccine: 21 [0.1%]; placebo: 11 [0.1%]) or death (vaccine: 105 [0.6%]; placebo: 61 [0.7%]) were infrequent and similar between groups; no fatal outcomes were assessed as related to the study vaccine (Supplementary Table S11).

Potential AESIs (programmed definition) were reported in 164 (1.0%) participants who received vaccine and 81 (1.0%) who received placebo, of which 11 (0.1%) and one (<0.1%), respectively, were considered related to vaccination (Table 2). The most common (≥20 participants in either group) potential AESIs were myocardial infarction (vaccine: 24 [0.1%]; placebo: 12 [0.1%]), pulmonary embolism (vaccine: 31 [0.2%]; placebo: 9 [0.1%]), and cerebrovascular accident (vaccine: 20 [0.1%]; placebo: 8 [0.1%]). Deep vein thrombosis was reported by 19 (0.1%) participants in the vaccine group and six (0.1%) in the placebo group.

Potential AESIs that qualified for assessment of TTS were reported for 15 (0.1%) participants who received the vaccine and eight (0.1%) who received placebo. None was classified as vaccine-induced immune thrombotic thrombocytopenia by the assessment committee.

The type and frequency of use of concomitant medications in the safety subset was similar in the vaccine and placebo groups (Supplementary Table S12).

## Discussion

The findings of this phase 3 study are consistent with those observed in phase 2b.^11^ A single dose of the Ad26.RSV.preF/RSV preF protein vaccine demonstrated 72.7% and 67.5% vaccine efficacy for prevention of RSV-mediated LRTD and ARI, respectively, in adults 60 years of age and older. Vaccine efficacy of Ad26.RSV.preF/RSV preF protein vaccine in preventing of RSV-mediated LRTD and ARI was maintained across age groups and in participants at increased risk for severe RSV disease. Vaccine efficacy against LRTD and ARI was maintained during the second year. Furthermore, ARI symptom severity was reported to be lower in vaccinated participants and return to usual health was reported to be faster by vaccinated participants than by those who received placebo, although this analysis was limited to the small subset of participants who experienced RSV and symptom severity was self-reported.

Vaccine efficacy is comparable to those of three other trials investigating RSV preF–associated vaccines in adults 60 years of age and older, all of which demonstrated greater than 82% vaccine efficacy against RSV-related LRTD and greater than 62% efficacy against RSV-related ARI.^5^^,^^6^^,^^9^ However, because there is no standardised case definition for RSV LRTD or RSV ARI, different case definitions were used in these studies, complicating detailed comparisons of vaccine efficacy between these trials. In our study, Ad26.RSV.preF/RSV preF vaccine efficacy was less pronounced against the RSV A versus RSV B subtype, despite the highly conserved nature of the RSV F glycoprotein across the two RSV subtypes.^5^ This difference may have been due to the relatively low number of RSV A cases observed.

Consistent with previous studies, the Ad26.RSV.preF/RSV preF protein vaccine elicited robust humoural and cellular immune responses that were not impacted by participant age or risk level for severe RSV disease.^11^^,^^20^^,^^21^ Ad26.RSV.preF/RSV preF protein was also generally well-tolerated and had an acceptable safety profile. The incidence of potential AESIs was similar in both study groups. A slight increase in pulmonary embolism/deep vein thrombosis was observed in participants exposed to the Ad26.RSV.preF/RSV preF protein vaccine versus placebo; however, given the relatively low frequency of the events, the presence of confounding comorbidities, and the 2:1 randomisation used in the current study, it is unclear if the frequency is truly increased. No cases of vaccine-induced immune thrombotic thrombocytopenia were confirmed by the assessment committee of internal and external experts.

Strengths of this study were the large, globally-recruited sample of older adults and the inclusion of those at high risk for severe RSV disease. While study populations were generally reflective of the regions in which they were conducted, participants from North America and White participants were overrepresented. Additionally, around 19% of enrolled individuals were aged 75 years or older.

One limitation of the study was that recruitment occurred during the COVID-19 pandemic, which resulted in off-season RSV outbreaks and lower-than-expected circulation during the 2020 to 2021 season.^22^^,^^23^ Consequently, the recruitment window likely extended into the RSV seasonal epidemic for some geographical locations. Other study limitations were the exclusion of institutionalised older adults and immunocompromised individuals who are at increased risk of RSV, and the small participant subset for reactogenicity and immunogenicity analyses. The study and vaccine development were discontinued due to a strategic decision by the study sponsor. At that time fewer than 1% of participants had completed the planned 24 months of follow-up, and there was limited follow-up available for countries/regions included later in the trial, precluding country/region-specific analyses. Despite the limited number of RSV cases during the second year, the study results are consistent with Ad26.RSV.preF/RSV preF protein vaccine efficacy reported previously.^11^ Despite these limitations, this study highlights that vectored approaches for RSV vaccination are feasible.

In conclusion, the Ad26.RSV.preF/RSV preF protein vaccine was efficacious for prevention of RSV-mediated LRTD and ARI and elicited humoural and cellular immune responses, with an acceptable safety profile, in a phase 3 study of adults 60 years of age or older after the first RSV season.

## Contributors

C.A.C. contributed to the conceptualisation of the study, development and design of the methodology, management and coordination responsibility for the research activity planning and execution, data collection, analysis, interpretation, and review and editing of the manuscript. M.T. contributed to the conceptualisation of the study and formal analyses of data, medical and safety monitoring activities during the study, review of data, and review and editing of the manuscript. E.G. contributed to the conceptualisation of the study, formal analyses of data, and review and editing of the manuscript. A.R.B. contributed to the conceptualisation of the study, development and design of the methodology, management and coordination responsibility for the biomarker analysis activity strategy, planning, and execution, data analysis and interpretation, and review and editing of the manuscript. M.L.N. contributed to data collection, analysis, interpretation, and review and editing of the manuscript. H.P. contributed to data collection, analysis, and interpretation. F.Z. contributed to data collection, analysis, and interpretation. V.F. contributed to the review of data and statistical analyses, and review and editing of the manuscript. W.B.S. contributed to the management and coordination responsibility for the research activity planning and execution, data collection, analysis, interpretation, and review and editing of the manuscript. P.J. contributed to the investigation and review and editing of the manuscript. J.M. contributed to the development and design of study methodology; data collection, analysis, interpretation, and review and editing the manuscript. M.L. contributed to the conceptualisation of the study, development and design of the methodology, management and coordination responsibility for the research activity planning and execution, data collection, analysis, interpretation, validation of the results, and review and editing of the manuscript. E.B.E. contributed to the development and design of study methodology, management and coordination responsibility for the research activity planning and execution, data collection, analysis, and interpretation, validation of results, and review and editing of the manuscript. A.M.J. contributed to the conduct of study and review and editing of the manuscript. K.W. contributed to the study design and review and editing of the manuscript. A.R.F. contributed to data analysis, interpretation, and review and editing of the manuscript. B.C. contributed to the conceptualisation of study design, development and design of the methodology, interpretation of results and data, and review and editing of the manuscript. E.H. contributed to the conceptualisation of the study, development and design of the methodology, management and coordination responsibility for the research activity planning, data collection, analysis, and interpretation, and review and editing of the manuscript. B.C. and A.R.F. had access to and verified the data. All authors reviewed and approved the manuscript for initial submission. All authors also approved the final version, with the exception of F.Z. who was unavailable to review or approve the final resubmission.

## Data sharing statement

The data sharing policy of Johnson & Johnson is available at https://innovativemedicine.jnj.com/our-innovation/clinical-trials/transparency. As noted on this site, requests for access to the study data can be submitted through Yale Open Data Access [YODA] Project site at http://yoda.yale.edu.

## Declaration of interests

C.A.C., M.T., E.G., A.R.B., A.M.J., K.W., B.C., and E.H. are former employees of Johnson & Johnson. V.F. is a consultant formerly employed by IQVIA and funded by Johnson & Johnson to provide statistical support. W.B.S. is an employee of Alliance for Multispecialty Research, LLC, a clinical research site. J.M. is a current employee of and holds shares or stock options in Johnson & Johnson. M.L. and E.B.E. are employees of Johnson & Johnson. A.R.F. received research grants from Janssen, Merck Sharp & Dohme, Pfizer, BioFire Diagnostics, Moderna, Vax Co., and CyanVac; consulting fees from Sanofi Pasteur, ADMA Biologics, and Arrowhead; and personal fees for serving on a data safety monitoring board for Novavax. M.L.N, H.P., F.Z., and P.J. have no conflicts of interest to declare.

## Appendix A Supplementary data

REDACTED_VAC18193RSV3001_Protocol Amend 5_1168310

REDACTED_SAP-FD-VAC18193RSV3001-395835_1168317

REDACTED_DMC-FD-VAC18193RSV3001-429851_1578678
