# Supplementary material for: A randomised, double-blind, multicentre, placebo-controlled phase 3 efficacy, safety, and immunogenicity study of an Ad26.RSV.preF/RSV prefusion F-protein vaccine for the prevention of respiratory syncytial virus-mediated lower respiratory tract disease in adults aged 60 years and older
Source: eBioMedicine. 2026 Nov 4;133:106513. doi: 10.1016/j.ebiom.2026.106513 (PMC13760371; doi:10.1016/j.ebiom.2026.106513)
Supplement: Supplementary file 1 [file mmc1.pdf]

Janssen Vaccines & Prevention B.V.*

Clinical Protocol

A Randomized, Double-blind, Placebo-controlled Phase 3 Efficacy Study of an Ad26.RSV.preF-based Vaccine in the Prevention of Lower Respiratory Tract Disease Caused by RSV in Adults Aged 60 Years and Older

EVERGREEN

Protocol VAC18193RSV3001; Phase 3
AMENDMENT 5

VAC18193 (Ad26.RSV.preF [JNJ-64400141]/ RSV preF Protein [JNJ-64213175])

* Janssen Vaccines & Prevention B.V. is a Janssen pharmaceutical company of Johnson & Johnson and is hereafter referred to as the sponsor of the study. The sponsor is identified on the Contact Information page that accompanies the protocol.

United States (US) sites of this study will be conducted under US Food & Drug Administration Investigational New Drug (IND) regulations (21 CFR Part 312).

EudraCT NUMBER: 2020-005458-97

**Status:**	Approved
**Date:**	11 October 2022
**Prepared by:**	Janssen Vaccines & Prevention B.V.
**EDMS number:**	EDMS-ERI-207539578, 10.0

GCP Compliance: This study will be conducted in compliance with Good Clinical Practice, and applicable regulatory requirements.

Confidentiality Statement
The information provided herein contains Company trade secrets, commercial or financial information that the Company customarily holds close and treats as confidential. The information is being provided under the assurance that the recipient will maintain the confidentiality of the information under applicable statutes, regulations, rules, protective orders or otherwise.

PROTOCOL AMENDMENT SUMMARY OF CHANGES TABLE

DOCUMENT HISTORY	
**Document**	**Date**
Amendment 5	11 October 2022
Amendment 4	8 April 2022
Amendment 3	10 November 2021
Amendment 2	19 July 2021
Amendment 1	3 May 2021
Original Protocol	30 November 2020

Amendment 5 (11 October 2022)

Overall Rationale for the Amendment:

The protocol is amended to provide clarification around the timing of the end-of-study analyses and population included, to align risk language with the most recent safety data available, and to provide additional clarification around follow-up that is conducted via phone call versus onsite visit. The changes made to this clinical protocol as part of Protocol Amendment 5 are listed below, including the rationale of each change and a list of all applicable sections. Changes made in previous protocol amendments are listed in Section 10.15, Appendix 15: Protocol Amendment History.

Section Number and Name	Description of Change	Brief Rationale
1.1 Synopsis, 1.3.1 Schedule of Activities, 4.1 Overall Design, 7.2 Participant Discontinuation/Withdrawal from the Study, 8 Study Assessments and Procedures	The Month 24 telephone call or telemedicine will be an onsite visit for participants who need to return eDevices to the site.<br><br>In addition, participants who withdraw before study completion will be encouraged to return the eDevice provided by the site, if applicable.	To allow participants to return eDevices to the site.
1.1 Synopsis, 4.1 Overall Design	The total number of participants was updated from approximately 27,500 participants (with up to approximately 4,500 participants in specific Asian countries/territories) to approximately 27,200 participants (with up to approximately 4,200 participants in specific Asian countries/territories).	No participants will be enrolled in South Korea, resulting in a smaller total number of participants for the study.
1.1 Synopsis, 4.1 Overall Design, 9 STATISTICAL CONSIDERATIONS, 9.2.1 Efficacy, 9.3 Populations for Analysis, 9.5.2 Additional Analyses, 9.5.3 End-of-Study Analyses	The timing of the end-of-study analysis and the population used was clarified.	To clarify the data used for the planned end-of-study analysis.
1.1 Synopsis, 4.1 Overall Study Design, 8.1 ARI Assessments and Procedures;	Allowance has been made for use of an equivalent version of the BioFire Filmarray Respiratory panel in	To reflect local regulations.

Section Number and Name	Description of Change	Brief Rationale
8.2.4 Diagnosis of RSV and Other Respiratory Infections	regions where the panel is not approved.	
1.3.1 Schedule of Activities, Footnotes a, b, and c, 8 STUDY ASSESSMENTS AND PROCEDURES	The timing of the Month 6 (Day 182) safety follow-up relative to the End of first RSV season visit, based on different enrollment scenarios, was clarified.	To clarify the process for the End of first RSV season/Day 182 follow-up.
1.3.1 Schedule of Activities, Footnote o, 8.1 ARI Assessments and Procedures	The RiiQ, the PGI-H and the EQ-5D-5L questionnaires do not need to be completed at Month 6.	To clarify the timing of assessments.
2.2 Background, Thrombosis with Thrombocytopenia Syndrome<br><br>8.5.6.1 Thrombosis with Thrombocytopenia Syndrome	The risk language around TTS has been updated.	To reflect the most recent safety data available with regards to vaccine-induced TTS in the RSV program as well as to align on the terminology used by regulatory authorities.
11 REFERENCES	Updated date of the Adenoviral Vaccine Safety Database Report (from V6.0 to V7.0).<br><br>Reference to the 2021 Investigator Brochure was removed.	The Adenoviral Vaccine Safety Database Report reference was updated to reflect the most current report available.<br><br>A reference to the latest Investigator Brochure was included in text.
Throughout	Updated “country” to “country/territory”.	Language was updated to accurately reflect the various geographies included in this study.

TABLE OF CONTENTS

PROTOCOL AMENDMENT SUMMARY OF CHANGES TABLE 2

TABLE OF CONTENTS 4

LIST OF IN-TEXT TABLES AND FIGURES 7

1. PROTOCOL SUMMARY 8
1.1. Synopsis 8
1.2. Schema 23
1.3. Schedule of Activities 24
1.3.1. Schedule of Activities – Assessments for All Participants 24
1.3.2. Schedule of Activities – Assessments for Participants with an ARI Episode 29

2. INTRODUCTION 32
2.1. Study Rationale 32
2.2. Background 32
2.3. Benefit-Risk Assessment 35
2.3.1. Risks of Study Participation 35
2.3.2. Benefits of Study Participation 37
2.3.3. Benefit-Risk Assessment of Study Participation 38

3. OBJECTIVES AND ENDPOINTS 38

4. STUDY DESIGN 43
4.1. Overall Design 43
4.2. Scientific Rationale for Study Design 47
4.2.1. Study-Specific Ethical Design Considerations 48
4.3. Justification for Dose 48
4.4. End of Study Definition 49

5. STUDY POPULATION 49
5.1. Inclusion Criteria 50
5.2. Exclusion Criteria 51
5.3. Lifestyle Considerations 53
5.4. Screen Failures 54
5.5. Criteria for Temporarily Delaying Administration of Study Vaccine 54

6. STUDY VACCINE AND CONCOMITANT THERAPY 54
6.1. Study Vaccine Administration 54
6.2. Preparation/Handling/Storage/Accountability 55
6.3. Measures to Minimize Bias: Randomization and Blinding 56
6.4. Study Vaccination Compliance 58
6.5. Dose Modification 58
6.6. Continued Access to Study Vaccine After the End of the Study 58
6.7. Overdose 59
6.8. Concomitant Therapy 59

7. DISCONTINUATION OF STUDY VACCINATION AND PARTICIPANT DISCONTINUATION/WITHDRAWAL 60
7.1. Discontinuation of Study Vaccination 60
7.2. Participant Discontinuation/Withdrawal From the Study 60
7.2.1. Withdrawal From the Use of Research Samples 61
7.3. Lost to Follow-up 61

8. STUDY ASSESSMENTS AND PROCEDURES 62
8.1. ARI Assessments and Procedures 64
8.1.1. Respiratory Infection Intensity and Impact Questionnaire (RiiQ™v2) 70

8.1.2. EuroQoL, 5-Dimension, 5-Level Questionnaire 72
8.1.3. Patient Global Impression Scores 72
8.2. Efficacy Assessments 72
8.2.1. Case Definition for RT-PCR-confirmed RSV-mediated LRTD 73
8.2.2. Case Definition for RT-PCR-confirmed RSV-mediated ARI 74
8.2.3. Predefined Clinically Relevant Disease Associated with RT-PCR-confirmed RSV-mediated ARI 74
8.2.4. Diagnosis of RSV and Other Respiratory Infections 75
8.3. Immunogenicity Assessments 76
8.4. Safety Assessments 77
8.4.1. Physical Examinations 79
8.4.2. Vital Signs 79
8.5. Adverse Events, Serious Adverse Events, Adverse Events of Special Interest, and Other Safety Reporting 79
8.5.1. Time Period and Frequency for Collecting Adverse Event, Serious Adverse Event, and Adverse Event of Special Interest Information 80
8.5.2. Method of Detecting Adverse Events, Serious Adverse Events, and Adverse Events of Special Interest 81
8.5.3. Follow-up of Adverse Events, Serious Adverse Events, and Adverse Events of Special Interest 82
8.5.4. Regulatory Reporting Requirements for Serious Adverse Events 82
8.5.5. Pregnancy 82
8.5.6. Adverse Events of Special Interest 83
8.5.6.1. Thrombosis with Thrombocytopenia Syndrome 83
8.6. Medical Resource Utilization and Health Economics 85
8.7. Participant Medical Information Prior to, During and After the Study (Real-world Data) 86

9. STATISTICAL CONSIDERATIONS 86
9.1. Statistical Hypotheses 86
9.2. Sample Size Determination 87
9.2.1. Efficacy 87
9.2.2. Immunogenicity 88
9.2.3. Safety 89
9.3. Populations for Analyses 90
9.4. Statistical Analyses 91
9.4.1. Efficacy Analysis 91
9.4.1.1. Primary Endpoint 91
9.4.1.2. Secondary Efficacy Endpoints 93
9.4.1.3. Testing Strategy for Primary and Confirmatory Secondary Endpoints 94
9.4.1.4. Exploratory Endpoints 94
9.4.2. Immunogenicity Analyses 96
9.4.3. Safety Analyses 97
9.4.4. Other Analyses 98
9.4.4.1. Patient-reported Outcomes 98
9.4.4.2. Medical Resource Utilization Analyses 98
9.5. Planned Analyses 98
9.5.1. Analysis Plan for the Interim and Primary Efficacy Analyses 98
9.5.2. Additional Analyses 101
9.5.3. End-of-Study Analysis 101
9.6. Committees 101
9.6.1. Independent Data Monitoring Committee 101
9.6.2. Clinical Event Adjudication Committee 102

10. SUPPORTING DOCUMENTATION AND OPERATIONAL CONSIDERATIONS 103
10.1. Appendix 1: Abbreviations 103
10.2. Appendix 2: Regulatory, Ethical, and Study Oversight Considerations 105
10.2.1. Regulatory and Ethical Considerations 105
10.2.2. Financial Disclosure 108

10.2.3. Informed Consent Process 108
10.2.4. Data Protection 109
10.2.5. Long-Term Retention of Samples for Additional Future Research 110
10.2.6. Committees Structure 110
10.2.6.1. Independent Data Monitoring Committee 110
10.2.6.2. Clinical Event Adjudication Committee 110
10.2.7. Publication Policy/Dissemination of Clinical Study Data 111
10.2.8. Data Quality Assurance 112
10.2.9. Case Report Form Completion 112
10.2.10. Source Documents 113
10.2.11. Monitoring 113
10.2.12. On-Site Audits 114
10.2.13. Record Retention 114
10.2.14. Study and Site Start and Closure 115
10.3. Appendix 3: Adverse Events, Serious Adverse Events, Adverse Event of Special Interest, Product Quality Complaints, and Other Safety Reporting: Definitions and Procedures for Recording, Evaluating, Follow-up, and Reporting 116
10.3.1. Adverse Event Definitions and Classifications 116
10.3.2. Attribution Definitions 117
10.3.3. Severity Criteria 117
10.3.4. Special Reporting Situations 118
10.3.5. Procedures 118
10.3.6. Product Quality Complaint Handling 120
10.3.7. Contacting Sponsor Regarding Safety, Including Product Quality 120
10.4. Appendix 4: Toxicity Grading Scale 121
10.5. Appendix 5: Definition of Acute Respiratory Infection for Participants Aged ≥60 Years 123
10.6. Appendix 6: Medically-attended ARI Form 124
10.7. Appendix 7: ARI Surveillance Assessment 128
10.8. Appendix 8: Respiratory Infection Intensity and Impact Questionnaire (RiiQ™v2) – Symptom Scale and Impact on Daily Activity Scale 129
10.9. Appendix 9 : EuroQoL, 5-Dimension, 5-Level Questionnaire (EQ-5D-5L) 130
10.10. Appendix 10: Patient Global Impression Scales 133
10.11. Appendix 11: Medical Resources Utilization Questionnaire 134
10.12. Appendix 12: Missed Working Days 136
10.13. Appendix 13: Guidance on Study Conduct During a Natural Disaster 137
10.14. Appendix 14: Thrombotic Events to be Reported as Potential AESIs 139
10.15. Appendix 15: Protocol Amendment History 140

11. REFERENCES 154

INVESTIGATOR AGREEMENT 156

LIST OF IN-TEXT TABLES AND FIGURES

TABLES

Table 1: Clinical Studies with RSV PreF Protein and/or Ad26.RSV.preF in the Adult Population 34
Table 2: Study Design: VAC18193RSV3001 44
Table 3: Strategy for Local Site and Central Testing of the Nasal Swabs and Sputum Samples 76
Table 4: Laboratory Tests That May Be Performed Upon Sponsor Request on Immunogenicity Samples Collected on Day 1 and 15 After Potential AESI Reporting 84
Table 5: Laboratory Tests That May Be Requested by the Sponsor to be Performed at the Central Laboratory After Potential AESI Reporting 84
Table 6: Distance from the Mean to the Limits of 95% CI Around the Actual Value at Day 15 for Pre-F ELISA, VNA, and ELISpot 89
Table 7: Probability of Observing at Least One Adverse Event or Serious Adverse Event at a Given True Adverse Event Rate in the Active Vaccine Group (Group 1) 90
Table 8: Secondary Endpoints: Overview of the Testing Order, Timing of the Testing in the Testing Strategy and the Corresponding Alpha 94
Table 9: Alpha-spending at the Prespecified Analysis Timepoints 99

FIGURES

Figure 1: Schematic Overview of the Study for all Participants 23
Figure 2: Schematic Overview of the ARI Assessments and Procedures 65
Figure 3: Interim and Primary Analysis Plan 100

1. PROTOCOL SUMMARY

1.1. Synopsis

A Randomized, Double-blind, Placebo-controlled Phase 3 Efficacy Study of an Ad26.RSV.preF-based Vaccine in the Prevention of Lower Respiratory Tract Disease Caused by RSV in Adults Aged 60 Years and Older

The respiratory syncytial virus (RSV) vaccine that will be investigated in this study, Ad26/protein preF RSV vaccine, includes 2 components that are being mixed prior to administration as a single intramuscular (IM) injection:

- Ad26.RSV.preF (JNJ-64400141), a replication-incompetent adenovirus serotype 26 (Ad26) containing a deoxyribonucleic acid (DNA) transgene encoding the pre-fusion conformation-stabilized F protein (preF) derived from the RSV A2 strain, and
- RSV preF protein (JNJ-64213175), a pre-fusion conformation-stabilized F protein derived from the RSV A2 strain.

OBJECTIVES AND ENDPOINTS

The primary objective of this study is to demonstrate efficacy of the Ad26.RSV.preF-based study vaccine in the prevention of reverse transcriptase polymerase chain reaction (RT-PCR)-confirmed RSV-mediated lower respiratory tract disease (LRTD) when compared to placebo in adults aged 60 years and above. Interim analyses to evaluate the primary objective will be performed by the Independent Data Monitoring Committee (IDMC). The primary, secondary and exploratory endpoints and objectives of the study are presented below.

Primary Objective, Secondary Efficacy Objectives and Exploratory Objectives

Objectives	Endpoints
**PRIMARY**	
• To demonstrate the efficacy of the active Ad26.RSV.preF-based study vaccine in the prevention of RT-PCR-confirmed RSV-mediated LRTD when compared to placebo in adults aged 60 years and above	• First occurrence of RT-PCR-confirmed, RSV-mediated LRTD with onset at least 14 days after dosing of study vaccine
**CONFIRMATORY SECONDARY***	
• To demonstrate the efficacy of active study vaccine in the prevention of any RT-PCR-confirmed RSV-mediated acute respiratory infection (ARI) when compared to placebo	• First occurrence of any RT-PCR-confirmed RSV-mediated ARI with onset at least 14 days after dosing of study vaccine
• To demonstrate the efficacy of active study vaccine in the prevention of RT-PCR-confirmed RSV-mediated LRTD during the second year when compared to placebo in adults aged 60 years and above	• First occurrence of RT-PCR-confirmed RSV-mediated LRTD during the second year, with onset after study Day 365
• To demonstrate the efficacy of active study vaccine in the prevention of any RT-PCR-confirmed RSV-mediated ARI during the second year when compared to placebo	• First occurrence of any RT-PCR-confirmed RSV-mediated ARI during the second year, with onset after study Day 365

Objectives	Endpoints
• To demonstrate the efficacy of active study vaccine in the prevention of predefined clinically relevant disease associated with RT-PCR-confirmed RSV-mediated ARI over the whole study when compared to placebo	• First occurrence of predefined clinically relevant disease associated with RT-PCR-confirmed RSV-mediated ARI over the whole study with onset at least 14 days after dosing of study vaccine
**NON-CONFIRMATORY SECONDARY**	
• To evaluate safety in terms of serious adverse events (SAEs) and adverse events of special interest (AESIs) until 6 months after vaccination	• Occurrence and relationship of vaccination to SAEs and AESIs until 6 months after vaccination
• In the Safety Subset and in subgroups of the Safety Subset (including but not limited to participants at increased risk of severe RSV disease), to evaluate the safety and reactogenicity in terms of solicited local and systemic adverse events (AEs) during 7 days after vaccination, and in terms of unsolicited AEs during 28 days after vaccination	• Occurrence, intensity, duration and relationship to vaccination of solicited local and systemic AEs during 7 days after vaccination and of unsolicited AEs during 28 days after vaccination
• In the Immuno Subset and in subgroups of the Immuno Subset (including but not limited to participants at increased risk of severe RSV disease), to evaluate the immunogenicity of active study vaccine when compared to placebo	• Characterization of the humoral and cellular immune responses in the Immuno Subset with emphasis on neutralizing and binding antibodies and antigen-specific cytokine production by T-cells
• To assess the reduction of symptom severity in participants with an RT-PCR-confirmed RSV-mediated ARI when compared to placebo over the whole study	• In participants with an RT-PCR-confirmed RSV-mediated ARI over the whole study: the area under the curve (AUC) of the change from baseline in Respiratory Infection Intensity and Impact Questionnaire (RiiQ[a]) Total Symptom score
**EXPLORATORY**	
• To demonstrate the efficacy of active study vaccine in the prevention of RT-PCR-confirmed RSV-mediated LRTD during the first year when compared to placebo in adults aged 60 years and above	• First occurrence of RT-PCR-confirmed RSV-mediated LRTD during the first year, with onset at least 14 days after dosing of study vaccine, and prior to study Day 365
• To demonstrate the efficacy of active study vaccine in the prevention of RT-PCR-confirmed RSV-mediated ARI during the first year when compared to placebo	• First occurrence of RT-PCR-confirmed RSV-mediated ARI during the first year, with onset at least 14 days after dosing of study vaccine, and prior to study Day 365
• To explore the effect of active study vaccine on RSV A and B infection when compared to placebo	• Assessment of the RSV A and B viral load by quantitative RT-PCR

[a] The Respiratory Infection Intensity and Impact Questionnaire used in this study is the RiiQ Symptom Scale and the RiiQ Impact on Daily Activities Scale of the RiiQ™ Version 2 (hereafter referred to as RiiQ).

Objectives	Endpoints
• To explore the efficacy of active study vaccine in the prevention of any RT-PCR-confirmed RSV-mediated LRTD caused by an RSV A and/or B strain when compared to placebo<br>○ during the first year<br>○ during the second year<br>○ over the whole study	• First occurrence of any RT-PCR-confirmed RSV-mediated LRTD caused by an RSV A strain and/or RSV B strain, respectively, during the considered years<br>○ with onset at least 14 days after dosing of study vaccine and prior to study Day 365 (first year comparison)<br>○ with onset after study Day 365 (second year comparison)<br>○ with onset at least 14 days after dosing of study vaccine (whole study comparison)
• To explore the efficacy of active study vaccine in the prevention of any RT-PCR-confirmed RSV-mediated ARI caused by an RSV A and/or B strain when compared to placebo<br>○ during the first year<br>○ during the second year<br>○ over the whole study	• First occurrence of any RT-PCR-confirmed RSV-mediated ARI caused by an RSV A strain and/or RSV B strain, respectively, during the considered years<br>○ with onset at least 14 days after dosing of study vaccine and prior to study Day 365 (first year comparison)<br>○ with onset after study Day 365 (second year comparison)<br>○ with onset at least 14 days after dosing of study vaccine (whole study comparison)
• To explore the efficacy of active study vaccine in the prevention of at least mild, at least moderate, and at least severe RT-PCR-confirmed RSV LRTD as assessed by the Clinical Event Adjudication Committee (CEC) compared to placebo<br>○ during the first year<br>○ during the second year<br>○ over the whole study	• First occurrence of any RT-PCR-confirmed RSV LRTD assessed by the CEC as:<br>- at least mild lower respiratory tract infection (LRTI)<br>- at least moderate LRTI<br>- at least severe LRTI<br>during the considered year:<br>○ with onset at least 14 days after dosing of study vaccine and prior to study Day 365 (first year comparison)<br>○ with onset after study Day 365 (second year comparison)<br>○ with onset at least 14 days after dosing of study vaccine (whole study comparison)
• To explore the reduction of symptom severity and time to return to usual health in participants with an RT-PCR-confirmed RSV-mediated ARI and in participants with RT-PCR-confirmed RSV-mediated LRTD when compared to placebo<br>○ during the first year<br>○ during the second year<br>○ over the whole study	• In participants with an RT-PCR-confirmed RSV-mediated ARI and in participants with RT-PCR-confirmed RSV-mediated LRTD (during the first year, the second year, or over the whole study, respectively):<br>○ the AUC of the change from baseline in RiiQ Total Symptom score<br>○ the time to return to usual health

Objectives	Endpoints
• To explore the reduction of disease severity in participants with an RT-PCR-confirmed RSV-mediated ARI and in participants with RT-PCR-confirmed RSV-mediated LRTD when compared to placebo, by subtype (RSV A, RSV B)<br>○ during the first year<br>○ during the second year<br>○ over the whole study	• In participants with an RT-PCR-confirmed RSV-mediated ARI and in participants with RT-PCR-confirmed RSV-mediated LRTD (during the first year, the second year or over the whole study respectively), by subtype (RSV A, RSV B):<br>○ the AUC of the change from baseline in RiiQ Total Symptom score<br>○ the time to return to usual health
• To explore the impact of active study vaccine on the course of an RT-PCR-confirmed RSV-mediated ARI and of human metapneumovirus (hMPV)- and influenza-mediated respiratory infections, general health status and health-related quality of life (HRQoL) measures when compared to placebo	• HRQoL and health status reported by participants on the RiiQ, patient global impression scales, and EuroQoL, 5-Dimension, 5-Level (EQ-5D-5L) in participants with an RT-PCR-confirmed RSV-mediated ARI or hMPV and influenza-mediated respiratory infections
• To explore the effect of active study vaccine on each of the following separately: the potential complications (including pneumonia), hospitalizations, emerging therapeutic use and medical resource utilization (MRU) of an RT-PCR-confirmed RSV-mediated ARI, an RT-PCR-confirmed RSV-mediated LRTD, and of hMPV and respiratory infections when compared to placebo<br>○ during the first year<br>○ during the second year<br>○ over the whole study<br>For influenza, the objective is to explore the incidence of these endpoints.	• For each of the following separately: (1) complications, (2) pneumonia, (3) hospitalizations, (4) emerging therapeutic interventions and (5) MRU, all associated with RT-PCR-confirmed RSV-mediated ARI or RT-PCR-confirmed RSV-mediated LRTD during the first year, the second year and over the whole study<br>○ with onset at least 14 days after dosing of study vaccine and prior to study Day 365 (first year comparison)<br>○ with onset after study Day 365 (second year comparison)<br>○ with onset at least 14 days after dosing of study vaccine (whole study comparison)<br>• Complications, pneumonia, hospitalizations, emerging therapeutic use and MRU associated with hMPV and influenza-mediated respiratory infections will be defined similarly
• To explore the efficacy of active study vaccine in the prevention of RT-PCR-confirmed hMPV-mediated ARI and RT-PCR-confirmed hMPV-mediated LRTD when compared to placebo in adults aged 60 years and above	• First occurrence of RT-PCR-confirmed, hMPV-mediated ARI and RT-PCR-confirmed hMPV-mediated LRTD with onset at least 14 days after dosing of study vaccine
• To explore the immune response biomarkers in study participants as correlates of risk of RSV disease and as correlates of protection induced by the active study vaccine	• Assessment of the correlation of immune responses with emphasis on neutralizing and binding antibodies with the risk of RSV disease and protection induced by the vaccine
• To explore biomarkers for the diagnosis of RSV infection, RSV-mediated LRTD and hMPV and influenza- mediated respiratory infections	• Assessment of blood samples collected during ARI episodes for biomarkers that correlate with RSV infection, RSV-mediated LRTD and hMPV and influenza-mediated respiratory infections

Objectives	Endpoints
• To explore additional vaccine-elicited immune responses in the Immuno Subset	• Assays that may be used include, but are not limited to:<br>○ RSV cross-neutralization of B and/or other A strain(s)<br>○ Antigen specific antibody functional and molecular characterization<br>○ Analysis of neutralizing antibodies to Ad26<br>○ Antigen-specific T-cell immune responses using detailed immunoprofiling
• To increase the information on prior medical history (electronic health records, claims, laboratory data from other care settings) in order to further evaluate its potential effect on the response to immunization and the impact of immunization on efficacy and duration of efficacy as well as AEs that may occur during and after completion of the study	• Utilization of tokenization and matching procedures for exploratory analysis of participant's medical data prior to, during, and following participation in the study (real-world data). Analysis will be performed to relate real-world data to vaccine immune responses, efficacy and duration of protection, and AEs

*Included in the testing strategy

Hypotheses

The study is designed to test the primary hypothesis of VE against RT-PCR-confirmed RSV-mediated LRTD in the PPE population.

- The hypothesis for the primary endpoint is:
  - Null hypothesis: the VE against RT-PCR-confirmed RSV-mediated LRTD of Group 1 vs placebo is ≤20%.
  - Alternative hypothesis: the VE against RT-PCR-confirmed RSV-mediated LRTD of Group 1 vs placebo is >20%.

The study is successful if:

- The lower limit (LL) of the 95% 2-sided confidence interval (CI, potentially corrected as described in Section 9.4.1.3) around the VE (1-relative risk rate) calculated from the Exact Poisson regression model is above 20% and additionally a point-estimate of VE >50% is observed for Group 1

OVERALL DESIGN

This is a multicenter, randomized, double-blind, placebo-controlled Phase 3 confirmatory efficacy study in participants aged 60 years and older. The primary objective of the study is to further establish the efficacy of the active Ad26.RSV.preF-based study vaccine in the prevention of RT-PCR-confirmed RSV-mediated LRTD.

Up to approximately 27,200 participants (with up to approximately 23,000 in the Global cohort, and up to approximately 4,200 participants in specific Asian countries/territories under local protocols) will be enrolled and randomized in parallel in a 2:1 ratio to receive active study vaccine or placebo.

Note that some additional local cohort enrollment beyond the Global cohort enrollment may be allowed if required by local health authorities for the purpose of local health authority consideration.

- The Global cohort is defined as all participants (including the Safety subset and the Immuno subset) who are recruited up to the point that global enrollment is stopped.

- Local cohorts are defined as participants from the considered country/territory and under local protocols.

For analyses purposes, data from the Global cohort and the local cohorts may be aggregated. Analyses for local cohort enrollment will be described separately, but they will also be included in the primary analysis and final analysis if their enrollment started prior to the database cut-off of the primary analysis or final analysis, respectively.

The study includes 1 dose of study vaccine at Day 1.

The study will be discontinued if the primary analysis is performed by the sponsor and the outcome is negative; however, if at that timepoint, some participants have not completed 6 months of follow-up after their vaccination, the study will continue until this follow-up period is complete.

Study Design: VAC18193RSV3001

Group	N	Day 1
1	15,340	Ad26/protein preF RSV vaccine (1×10^{11} vp/150 µg)
2	7,670	Placebo

N number of participants, vp viral particles

ARI ASSESSMENTS AND PROCEDURES

From the study vaccination until 24 months after vaccination (ie, the full duration of the study), participants will be followed up to identify potential cases of RSV infection. After administration of study vaccine, ARI surveillance will be conducted twice weekly via the participant's eDevice (or desktop computer, where applicable) for the duration of the study. The surveillance questions ask participants if they have experienced any ARI symptoms, or for participants who have one or more of these symptoms at baseline, if they have experienced any additional ARI symptoms or a worsening of their baseline symptoms. Participants who experience any symptoms suggestive of an ARI (new onset or worsening, in the setting that baseline symptoms are present) are instructed to indicate this (refer to Appendix 7: ARI Surveillance Assessment) and start completing the required ARI episode questionnaires[a] on a daily basis (preferably in the evening). Ideally within the next 24 hours, the site and participant should have telephone or telemedicine contact with each other in order for the site to confirm that the participant is experiencing symptoms consistent with an ARI[b] and to give instructions to the participant on the further ARI episode procedures to be followed. Participants will collect a nasal swab at home on the day of symptom onset or the day thereafter (ARI Day 1-2).

On ARI Day 3-5, participants will present to the site, where vital signs will be measured, and a nasal swab, a sputum sample (in participants with a productive cough, when possible), and blood samples (to measure the immune responses at the time of exposure and for exploration of biomarkers that correlate with respiratory infection and disease severity) may be taken by a member of the study staff (see Section 8.1 to

[a] The following questionnaires will need to be completed on a daily basis: the RiiQ Symptom Scale and the RiiQ Impact on Daily Activities Scale, the European Quality of Life, 5 Dimension, 5 Level (EQ 5D 5L), the Patient Global Impression of Health (PGI H) scale, the Patient Global Impression of Severity (PGI S) scale, the Patient Global Impression of Change (PGI C) scale, and the Return to Usual Health question. Note that the PGI C scale and Return to Usual Health question will not be completed on ARI Day 1.

[b] This will be assessed by the site by confirming that at least one of the symptoms used to confirm the ARI from the RiiQ Symptom Scale is worse than baseline. As defined in Appendix 5, symptoms used to confirm an ARI episode are: nasal congestion, sore throat, cough, short of breath, coughing up phlegm (sputum), and wheezing (from the RiiQ Symptom Scale). As it is possible that the participants will make errors when they answer the RiiQ Symptom Scale, the telephone or telemedicine contact will be used by the sites to also confirm the correctness of the answers provided by the participant and to confirm the ARI episode was correctly triggered.

determine if the blood samples can be omitted). Ideally within 24 hours after the ARI Day 3-5 visit (but no later than 48 hours after the ARI Day 3-5 visit), a qualified member of the study staff will perform a local RT-PCR of all available nasal swabs with the BioFire® Filmarray Respiratory panel, which will be provided to all sites (both US and non-US); changes to the process might be implemented at the local level based on local requirements. Participants will subsequently be contacted by telephone and given further instructions, as detailed below, on how to further proceed based on the outcome of the RT-PCR tests. Sputum samples (where applicable) will be sent to the central laboratory for testing using the BioFire Filmarray Pneumonia (PN) panel. For all ARI episodes where a sputum sample is collected, the ARI episode will be followed up until ARI resolution (ie, with daily questionnaires and an ARI Day 29 visit) (refer to the procedures in the first bullet point below), independent of the test results from the collected nasal swabs. Participants who are under self-isolation because of a (possible) positive test for SARS-CoV-2 will not come to the site for ARI Day 3-5 assessments but will self-collect the nasal swab.

- In the unlikely event where at least one nasal swab from an ARI episode cannot be tested at the site with the BioFire Filmarray Respiratory panel (eg, in the setting of instrument malfunction, unavailability of the device in the country/territory or at the site, or shortage of testing reagents at the site), this sample(s) should be sent to the central laboratory for testing with the BioFire Filmarray Respiratory panel, and the ARI episode will be followed up until ARI resolution (ie, with daily questionnaires and an ARI Day 29 visit) (refer to the procedures in the first bullet point below), independent of the results from other collected samples for that ARI episode. Further details on the strategy for local site and central testing of the nasal swabs and sputum samples are provided in Section 8.2.4.
- Participants with an ARI and a RT-PCR positive for RSV, hMPV, or influenza-mediated ARI will continue filling out the ARI episode questionnaires daily until resolution of the ARI episode (defined as 2 consecutive days with no symptoms listed on the RiiQ Symptom Scale, or, for participants who have RiiQ symptoms at baseline [assessed prevaccination], 2 consecutive days where all symptoms on the RiiQ Symptom Scale have returned to the same severity level as reported at baseline [or lower]). After resolution of the ARI episode, the participant will return to ARI surveillance with twice weekly reminders.

  On ARI Day 29 (±7 days), regardless of whether the ARI episode has resolved, participants with a RT-PCR positive for RSV, hMPV, or influenza-mediated ARI will be asked to return to the site. During this visit, the MRU questionnaire will be completed based on interview with the participant, participants will complete the RiiQ, the EQ-5D-5L and each of the Patient Global Impression (PGI) scales and the Return to Usual Health question, and the site staff will also collect information on days missed from work and on complications, hospitalizations and concomitant medications related to the ARI and record in the electronic case report form (eCRF). The ARI Day 29 visit can also take place by telephone or telemedicine contact.

  For medically-attended RT-PCR positive for RSV, hMPV, or influenza-mediated ARIs, including those resulting in hospitalization, a standard question list will be provided to collect additional information on any other diagnostic tests (eg, chest x-rays, spirometry, pulmonary function tests, etc.) or on any interventions during the clinical course of the ARI.

  ***Note*: the same procedures apply for participants with a sputum sample taken and for participants with at least one nasal swab that cannot be tested at the site with the BioFire Filmarray Respiratory panel.**
  Participants will remain blinded as to the outcome of RSV, hMPV and influenza RT-PCR test results until study unblinding and the test results should only be disclosed in the event of safety concerns. RT-PCR test results related to the detection of other respiratory pathogens can be communicated to the participants. The RT-PCR results that are obtained with the BioFire Filmarray testing under the study procedures will be used for research purposes only and will not be used by the investigator for therapeutic decisions. A locally approved diagnostic test external to the study procedures, including

the RSV RT-PCR, should be obtained in case the participant's own healthcare provider considers it warranted (eg, based on symptoms and/or risk of severe disease) and/or according to local/site specific guidelines. If a sample tests positive for SARS-CoV-2, the participant will be referred to their own healthcare provider for locally approved diagnostic test and further management, in accordance with local/site specific guidelines.

- Participants with negative RT-PCR results for RSV, hMPV, or influenza will be informed by telephone or telemedicine contact that they can stop filling out the daily ARI episode questionnaires and the site will end the ARI episode and return the participant to ARI surveillance.
- For participants with a positive SARS-CoV-2 test and a negative test for RSV, hMPV, and influenza, the collection of ARI data by the study site will be stopped for the ARI episode. The participant will be informed by telephone or telemedicine contact that they can stop filling out the daily ARI episode questionnaires, and the site will end the ARI episode and return the participant to ARI surveillance with twice weekly reminders. Participants who have a positive SARS-CoV-2 RT-PCR test as part of the study procedures should be referred to their own healthcare provider for locally approved diagnostic test and further management, in accordance with local/site specific guidelines. Participants whose standard-of-care testing during an ARI episode is positive for SARS-CoV-2, or who have been in contact with someone that tested positive for SARS-CoV-2 and therefore must self-isolate according to the local guidelines, will not come to the site for ARI Day 3-5 assessments but will self-collect the nasal swab. In this circumstance, other ARI Day 3-5 assessments (vital signs, sputum sample and blood samples) might not be performed.

  Participants with positive RT-PCR results for RSV (or hMPV or influenza) should keep on filling out the daily ARI episode questionnaires until resolution of the ARI episode independently of any other co-infection (including SARS-CoV-2).

When the end of RSV season one telephone call or telemedicine contact occurs within 6 months after vaccination, the participants will be contacted again by telephone or telemedicine at 6 months post-vaccination to collect information on SAEs and AESIs (applies to Visit 3 [end of season 1/6-month follow-up]).

At Month 24, all participants will be contacted by telephone or telemedicine (or come to the site if they have eDevices to return) to ensure the participants complete the RiiQ, the PGI-H and the EQ-5D-5L.

NUMBER OF PARTICIPANTS

Up to approximately 27,200 participants (with up to approximately 23,000 in the Global cohort, and up to approximately 4,200 participants in specific Asian countries/territories under local protocols) will be enrolled and randomized in parallel in a 2:1 ratio to receive active study vaccine or placebo.

VACCINE GROUPS AND DURATION

The investigational medicinal products to be administered to participants in this study are Ad26.RSV.preF, RSV preF protein, and placebo. The Ad26/protein preF RSV vaccine to be used in this study is composed of Ad26.RSV.preF and RSV preF protein, to be administered as a single injection in the deltoid muscle. Participants will be randomized to receive active vaccine or placebo as described in section "OVERALL DESIGN" above. All injections will be 1 mL in volume.

The study duration for an individual participant will be approximately 24 months.

EFFICACY EVALUATIONS

Throughout the study, nasal swabs and sputum samples will be collected when an ARI occurs. Confirmation of RSV infection by RT-PCR for the primary endpoint will be done in nasal swabs or sputum samples.

- Confirmation of RSV infection by RT-PCR in nasal swabs will be performed at the site laboratory with the BioFire® Filmarray Respiratory panel, which will be provided to all sites (both US and non-US). Both the home swab and the site swab will be analyzed at the site. The central RSV confirmation of the nasal swab with the BioFire Filmarray Respiratory panel will not be taken into account for the primary endpoint, unless in the unlikely event where at least one nasal swab from an ARI episode cannot be tested at the site (eg, in the setting of instrument malfunction, unavailability of the device in the country/territory or at the site, or shortage of testing reagents at the site).
- Confirmation of RSV infection by RT-PCR in sputum samples (when collected) will be performed in the central laboratory with the BioFire Filmarray PN panel.
- For participants hospitalized with symptoms of an ARI, confirmation of RSV infection using a locally approved RT-PCR test at the hospital laboratory can also be used. These results will be used in the primary analysis only if approved by an independent expert with expertise in diagnostic testing. The approval of the RT-PCR results by the expert will be based on the specifications of the test performed, the qualifications of the testing laboratory and the procedures followed in the laboratory.

One positive sample (defined as any sample with a value above the limit of detection) is sufficient.

The first occurrence of a considered endpoint is defined as the start of the first episode of the endpoint in the study (regardless of RSV A or B strain, unless otherwise specified).

Case Definition for RT-PCR-confirmed RSV-mediated LRTD

A participant will be considered to have RT-PCR-confirmed RSV-mediated LRTD if the following criteria are met:

- New onset or worsening from baseline of 3 or more of the below symptoms as captured on the RiiQ at the same assessment time point:
  - Cough
  - Short of breath
  - Coughing up phlegm (sputum)
  - Wheezing

  AND
- Confirmation of RSV by RT-PCR in one or more of the nasal swabs, or in the sputum sample (when available)

Counting of the number of symptoms with new onset or worsening will be done per assessment. If a participant completed the RiiQ more than once per day, the case definitions cannot be met by combining symptoms from different assessments on that day, the required number of symptoms must be attained at one assessment.

This definition will be programmed. The CEC will review all available data of all RSV-mediated ARIs and will confirm if the above criteria are met. If the CEC confirms that the above criteria for LRTD are met, the case will be counted as a primary endpoint in the statistical analysis.

Case Definition for RT-PCR-confirmed RSV-mediated ARI

A participant will be considered to have RT-PCR-confirmed RSV-mediated ARI if the following criteria are met:

- ARI episode initiated by the participant and confirmed by the site with symptoms consistent with an ARI (new symptoms or worsening from baseline of at least one of the symptoms as captured on the RiiQ):
  - Sore throat

- Nasal congestion
- Cough
- Short of breath
- Coughing up phlegm (sputum)
- Wheezing

AND

- Confirmation of RSV by RT-PCR in one or more of the nasal swabs, or in the sputum sample (when available)

Predefined Clinically Relevant Disease Associated with RT-PCR-confirmed RSV-mediated ARI

A participant will be considered to have clinically relevant disease with specific parameters associated with an RT-PCR-confirmed RSV-mediated ARI if the following criteria are met:

- The participant has an RT-PCR-confirmed RSV-mediated ARI as defined above.
- Any of the following is associated with the ARI:
  - Hospitalization
  - Emergency department visit
  - Per clinical judgement, at least one of the following complications: asthma exacerbation, chronic obstructive pulmonary disease (COPD) exacerbation, respiratory distress, bronchitis, bronchial hyperreactivity, congestive heart failure (CHF) exacerbation, cardiac arrhythmia, renal impairment or the presence of X-ray or radiological confirmed pneumonia, respiratory arrest and/or failure, pulmonary embolism, pleural effusion, atelectasis, acute coronary events, acute cerebrovascular events, altered mental status, seizure, syncope, systemic inflammatory response syndrome (SIRS), new neurological deficit, asthenia, dehydration or metabolic disturbances
  - For measurements at the site, decreased oxygen saturation is defined as oxygen saturation of <92% for participants with a baseline oxygen saturation of ≥92% at randomization; for participants with baseline oxygen saturation <92%, decreased oxygen saturation is defined as a ≥3% decrease in their oxygen saturation from baseline[a].
    Decreased oxygen saturation based on measurements during medically-attended ARIs (ie, hospitalization) is defined as oxygen saturation of <92% supporting LRTD.
  - Tachypnea (at least emerging Grade 2, see Appendix 4: Toxicity Grading Scale)
  - Need of supplemental oxygen
  - Hypotension (emerging Grade 3, see Appendix 4: Toxicity Grading Scale)
  - Pulmonary function test results supporting diagnosis of LRTD
  - Arterial blood gas results supporting diagnosis of LRTD

IMMUNOGENICITY EVALUATIONS

Blood samples will be collected from all participants for immunogenicity assessments before and 14 days after vaccination, and at 1 year after vaccination for the analysis of correlates of risk of RSV disease and correlates of protection.

For participants in the Immuno Subset (consisting of at least 360 participants, of whom ~50% will be at increased risk for severe RSV disease and ~50% will be 75 years or older), blood will be collected for analysis of humoral and cellular immune responses before study vaccination and at 2, 12 and 24 weeks, and 1 and 1.5 years after vaccination.

Immunogenicity assessments may include (but are not limited to) the assays summarized below.

---

[a] Based on vital signs collected at baseline and during the ARI at the site.

Humoral Assays	Purpose
Secondary endpoints	
RSV neutralization A	Analysis of neutralizing antibodies to an A strain
F protein antibodies (ELISA; pre F and/or post F)	Analysis of antibodies binding to RSV F protein in pre fusion and/or post fusion form
Exploratory endpoints	
RSV strain cross neutralization	Analysis of cross neutralizing antibodies to B and/or a different A strain(s) using virus neutralization assays and other surrogate antibody binding assays
Adenovirus neutralization assay	Analysis of neutralizing antibodies to adenovirus
Functional and molecular antibody characterization	Analysis of antibody characteristics including, but not limited to, ADCC, ADCP, avidity, epitope mapping, Ig isotype, functional VNAs to other respiratory viruses, and antibody assessments for antibody repertoire

ADCC=antibody dependent cell mediated cytotoxicity, ADCP=antibody dependent cellular phagocytosis, ELISA =enzyme linked immunosorbent assay, F=fusion, Ig=immunoglobulin, VNA=virus neutralizing antibody
Note: Antibody analyses may be performed in nasosorption samples and serum.

Cellular Assays [a]	Purpose
Secondary endpoints	
IFN γ ELISpot	T cell IFN γ responses to RSV F protein peptides
Exploratory endpoints	
Immunophenotyping	May include analysis of T cell and B cell responses to RSV F protein peptide stimulated PBMC (including, but not limited to, $CD4^+/CD8^+$, Th1/Th2, IL 2, IFN γ, TNF α, activation markers and memory) using flow cytometry or mass cytometry
Immune repertoire analysis	May include using antigen specific single cell sequencing or bulk sequencing in order to assess T cell and B cell repertoire analysis

[a] Cellular assays will be performed in the Immuno Subset only where PBMCs are collected.
ELISpot=enzyme linked immunospot, F=fusion; IFN γ=interferon gamma, IL 2 =interleukin 2, PBMC=peripheral blood mononuclear cells, TNF α=tumor necrosis factor alpha

Nasosorption samples using synthetic absorptive matrix (SAM) will be collected from Immuno Subset participants prior to vaccination and at the 2 weeks and 1-year post-vaccination visits and will be used for immunogenicity assessments that may include antigen-specific immunoglobulins (ie, IgG and IgA), microbiome, and other viral pathogen analyses.

The blood samples that are collected between 2 and 4 days after symptom onset (ARI Day 3-5) from participants with an RSV, hMPV, or influenza-mediated ARI episode will be assayed by serology (which may include pre-F/post-F enzyme-linked immunosorbent assay (ELISA) and RSV virus neutralizing antibodies [VNA], as available and applicable) to assess immune responses and correlates that are associated with RSV infection. The samples will also be used for exploration of biomarkers that correlate with RSV infection and RSV disease severity (which may include RT-PCR for RSV, ribonucleic acid [RNA] transcriptomics to assess gene regulation [clusters] and expression patterns, cytokine/chemokine-dependent immunophenotyping, and immune repertoire analysis, as available and applicable).

SAFETY EVALUATIONS

For All Participants

All (S)AEs and special reporting situations related to study procedures or to non-investigational (concomitant) Janssen products will be collected from informed consent form (ICF) signature onwards until the end of the study for all participants. All other SAEs and AESIs will be collected from administration of study vaccine (Day 1) until 6 months after vaccination. AEs leading to discontinuation from the study will be collected for the duration of the study. All COVID-19 cases will be collected for all participants for the duration of the study.

ARIs and complications related to RSV, hMPV, and influenza-mediated ARIs that classify as SAE will be captured in all participants and reported as SAEs in the eCRF continuously from the time of vaccination until 24 months after vaccination.

All participants will be closely observed for a minimum of 15 minutes after vaccination to monitor for the development of any acute reactogenicity. This post-vaccination observation period should be extended for participants whom in the investigator's opinion would require further observation or for any occurrence of a localized reaction or (minor) systemic symptoms during the post-vaccination observation that require monitoring for any progression.

Vital signs will be collected prior to vaccination for all participants. At non-vaccination visits, vital signs will be measured if deemed necessary by the investigator.

For Participants in the Safety Subset

Additional procedures will be carried out in the Safety Subset participants (consisting of at least 1,050 participants, of whom ~50% of all participants in each region will be at increased risk of severe RSV disease and ~50% of all participants in the Safety Subset in each region will be 75 years or older). Safety Subset participants will have provided consent for the additional study procedures[a]:

- At the end of the 15-minute observation period, vital signs (body temperature, blood pressure, heart rate, respiratory rate, oxygen saturation) will be obtained[b], and any unsolicited and solicited local and systemic AEs will be documented in the eCRF by study personnel.
- Participants will record solicited local (at the injection site) AEs, solicited systemic AEs, and body temperatures in the eDiary, beginning on the evening of the vaccine dosing day and on a daily basis for the following 7 days. If a solicited local or systemic AE is not resolved within 7 days after vaccination (Day 8), safety follow-up should be performed until the symptom resolves. The study-site personnel will be instructed to record the date of last symptoms and maximum severity until resolution in the source document and eCRF.
- All other AEs (unsolicited) and special reporting situations will be reported from the time of vaccination through the following 28 days. Participants will be contacted by telephone or telemedicine at 28 days (+3 days) after vaccination to collect information on unsolicited AEs and associated concomitant medications.
- All ARIs and all complications related to RSV, hMPV, and influenza-mediated ARIs that do not classify as SAEs will be reported as AEs in the eCRF from the time of vaccination through the following 28 days.

MEDICAL RESOURCE UTILIZATION

Medical resource utilization (MRU) data associated with medical encounters will be collected by the investigator and study site personnel at 28 days after onset of each ARI episode (ARI Day 29 ±7 days) for all ARIs that are followed until their resolution (ie, for RSV, hMPV, or influenza-positive ARIs, for ARIs with sputum collection, and for ARIs where at least one nasal swab cannot be tested at the site with the BioFire Filmarray Respiratory panel). Protocol-mandated procedures, tests, and encounters are excluded. The data collected may be used to conduct exploratory economic analyses and will include:

- Number and type of medical consultations (including primary care and specialist visits), whether these consultations were related to ARIs and a reason for each medical encounter

[a] Note that participants can participate in both the Immuno Subset and the Safety Subset.
[b] Actual values (normal and abnormal) will only be documented in the eCRF for body temperature.

- Number and duration of hospital and/or institutional care admissions (total days length of stay, including duration by wards; eg, intensive care unit), whether these admissions were related to ARIs, and a reason for each admission
- Number and type of emergency department visits and whether these emergency department visits were related to ARIs
- Where applicable, information on whether supplemental oxygen or mechanical ventilation was used
- Whether participation in the study (ie, contact with healthcare providers during study visits and procedures) increased or decreased the participant's use of medical resources outside of the study

PARTICIPANT MEDICAL INFORMATION PRIOR TO, DURING AND AFTER THE STUDY (REAL-WORLD DATA)

For participants (from the US only) who have provided consent for this, medical data (electronic health records, claims and laboratory data from other care settings) from 5 years prior to study enrollment until 5 years after study completion may be accessed utilizing tokenization and matching procedures (ie, the generation of anonymous identifiers or "tokens" [hashed and encrypted combinations of identifying elements] to allow linking of participant data from different sources without compromising the participant's confidentiality). These data may be used for exploratory analyses to enhance our understanding of the impact of prior medical history on the response to immunization and the impact of immunization on efficacy and duration of efficacy as well as adverse events that may occur during and after completion of the study. The analyses will be described in detail in a dedicated analysis plan.

STATISTICAL METHODS

Sample Size Determination

The required sample size was determined using the following assumptions:

- a VE for RT-PCR-confirmed RSV-mediated LRTD of 65% during the first year and during the second year,
- an incidence of RT-PCR-confirmed RSV-mediated LRTD of at least 0.2% during the first RSV season (2021-2022) and an incidence of 0.5% in later RSV seasons in 60^+-year-old placebo recipients,
- a randomization ratio of 2:1 (active vaccine: placebo),
- an analysis plan as described in Section 9.5.1,
- a 1-sided α of 2.5% (potentially corrected for multiplicity), and
- 10% of exclusions (due to dropout, major protocol violations, etc.) per year

Simulations performed in R show that with ~15,340 participants in the active vaccine group (Group 1) and ~7,670 participants in the placebo group (Group 2) the study has at least 90% total power to demonstrate VE against the primary endpoint statistically significant >20% and a point-estimate for VE >50%. This leads to a sample size of ~23,000 participants.

This sample size is also required to reach approximately 80% power for the secondary endpoint of first occurrence of RT-PCR-confirmed RSV-mediated LRTD during the second year.

Efficacy Analysis

<u>Primary Endpoint</u>

The analysis of the primary endpoint will evaluate the first occurrence of RT-PCR-confirmed RSV-mediated LRTD with onset of at least 14 days after vaccination in the active vaccine group compared to the placebo group in the PPE population. The null hypothesis of LL ≤20% for RT-PCR-confirmed RSV-

mediated LRTD will be tested versus the alternative hypothesis of LL >20%. Additionally, the observed VE should be >50%.

Exact Poisson regression will be fitted with the event rate, defined as the number of cases (with onset at least 14 days after vaccination) over the follow-up time (off-set) as dependent variable and the vaccination group, being at increased risk of RSV disease, and age stratum (both variables as stratified) as independent variables. This model will be based on the PPE population.

The study is successful if:

- The LL of the 95% 2-sided CI (potentially corrected as described in Section 9.4.1.3) around the VE (1-relative risk rate) calculated from the Exact Poisson regression model is above 20% and additionally a point-estimate of VE >50% is observed for Group 1

As a sensitivity analysis, the above model will be repeated based on the Full Analysis Set (FAS), not taking into account the restriction on the onset (at least 14 days). Additional sensitivity analyses will be performed as well.

<u>Secondary Endpoints</u>

The following confirmatory secondary endpoints will only be tested when the primary endpoint shows statistical significance.

1. First occurrence of any RT-PCR-confirmed RSV ARI, with onset at least 14 days after dosing of study vaccine
2. First occurrence of any RT-PCR-confirmed RSV-mediated LRTD during the second year with onset after study Day 365
3. First occurrence of any RT-PCR-confirmed RSV ARI during the second year with onset after study Day 365
4. First occurrence of predefined clinically relevant disease associated with RT-PCR-confirmed RSV-mediated ARI over the whole study, with onset at least 14 days after dosing of study vaccine

The testing strategy will test those endpoints hierarchically. A similar Exact Poisson regression model as for the primary endpoint will be used for the analyses of the secondary endpoints #1, #2, #3, and #4 (see Section 9.4.1.2). For those secondary endpoints, the null hypothesis of VE ≤0% will be tested versus the alternative hypothesis VE >0%. Formal inference for secondary endpoint #1, as part of the testing strategy, will occur at the time of the primary analysis. For secondary endpoints 2, 3, and 4, the formal inference as part of the testing strategy will only occur at study end.

Immunogenicity Analyses

<u>Immunogenicity Subset</u>

No formal hypothesis on immunogenicity will be tested. Descriptive statistics (such as geometric mean and 95% CI for ELISA and RSV neutralization assay, and median and quartiles for interferon gamma [IFN-γ] enzyme-linked immunospot [ELISpot]) will be calculated for continuous immunogenicity parameters at all available timepoints. For the humoral assays, geometric mean fold rises from baseline with 95% Cis may additionally be calculated. Baseline is considered as the last available assessment before vaccination. Graphical representations of immunogenicity parameters will be made as applicable.

<u>Correlates of Protection</u>

If VE is demonstrated, correlates of protection will be further explored in samples collected from all participants. Immunogenicity markers are considered correlates of protection if VE is explained through

the effect of the vaccine on the immunogenicity markers. More details with appropriate methods will be provided in a separate analysis plan.

Safety Analyses

No formal statistical testing of safety data is planned. The safety analysis will include the descriptive summary of solicited local AEs, solicited systemic AEs, unsolicited AEs, AESIs, SAEs, and COVID-19 infections. The overall frequencies per group as well as frequencies according to severity will be calculated for solicited and unsolicited AEs. The analysis of solicited, and unsolicited AEs will be restricted to a subset of the FAS (Safety Subset). For the analyses of AESIs, SAEs, and COVID-related AEs, the entire FAS population will be used.

Independent Data Monitoring Committee

The study will be formally monitored by the IDMC. In general, the IDMC will monitor the safety data on a regular basis to ensure the continuing safety of the participants. The IDMC will also formally monitor the primary efficacy endpoint. The IDMC will evaluate in an unblinded fashion if the success criteria have been met.

Clinical Event Adjudication Committee

Prior to database lock for all planned analyses, the CEC will review in a blinded manner all available data of all RSV-mediated ARIs and will confirm if the RT-PCR-confirmed RSV LRTD definition criteria were met. Further for all RT-PCR-confirmed RSV LRTD cases the CEC will assess their severity (mild, moderate or severe).

Details on the review approach will be provided in the CEC Charter.

1.2. Schema

Figure 1: Schematic Overview of the Study for all Participants

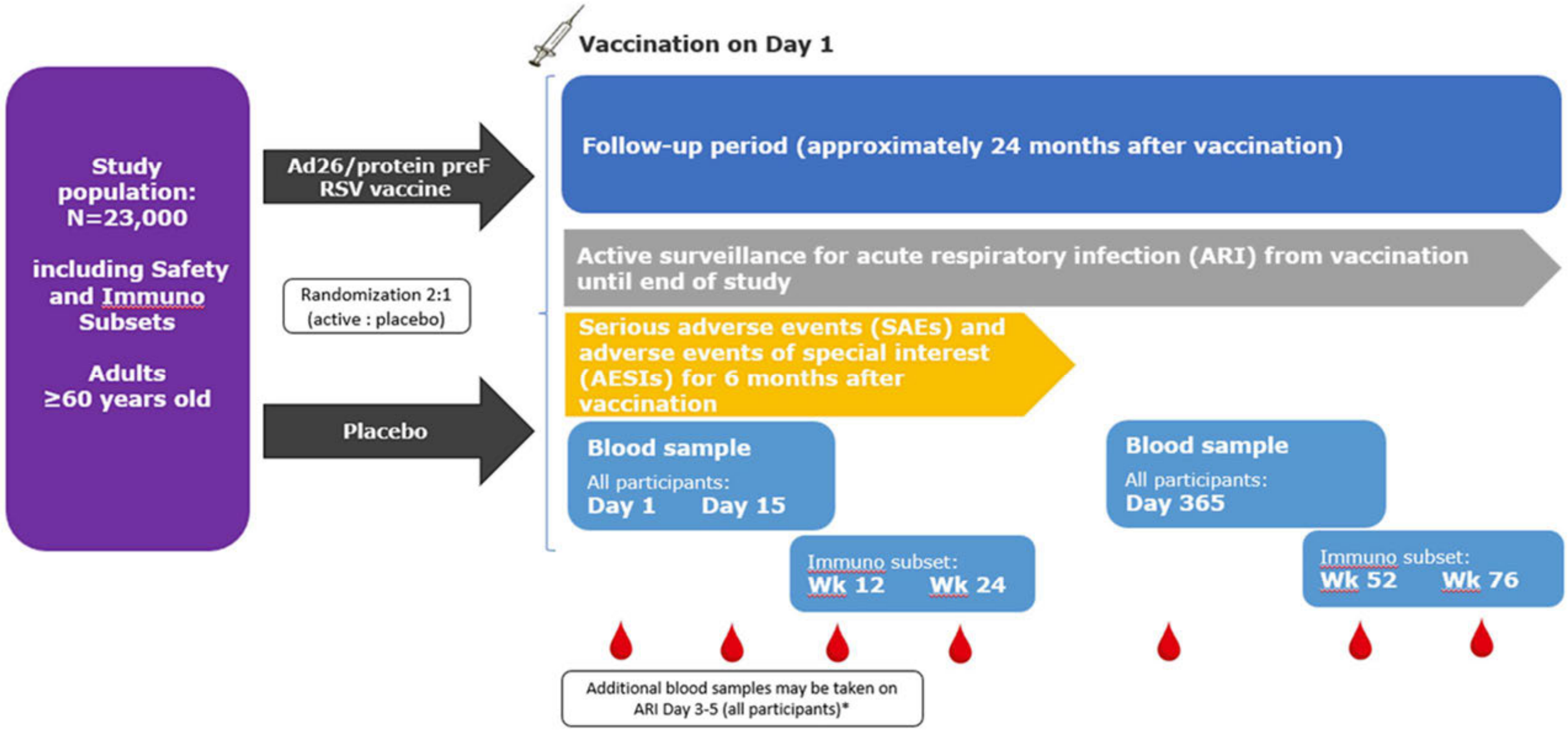

Additional assessments will be performed for participants in the Safety and Immuno Subsets.
Refer to the Schedule of Activities for assessments to be performed for participants with an ARI episode.
Schematic overviews for participants in specific Asian countries/territories are provided in their respective local protocols
*Refer to Section 1.3.2, footnote j to determine whether a blood sample is required during an ARI Day 3-5 visit

1.3. Schedule of Activities

1.3.1. Schedule of Activities – Assessments for All Participants

Visit #	1	2	2a ☎	2b	2c[a]	3[a,b] ☎	4[d]	4a[d]	5[d]
Visit Timing	Vac 1	Vac 1 +14 d	Vac 1 +28 d	Vac 1 +84 d	Vac 1 +24 we	End 1st RSV Season[c] or Month 6	Month 12	Month 18	Month 24
Visit Week	0	2	4	12	24	26	52	76	104
Visit Day	1	15[e]	29[e]	85[e]	169[e]	182	365[e]	533[e]	730[e]
Visit Window		+3 d	+3 d	±7 d	±14 d	-7/+14 d	-7/+14 d	±14 d	-7/+14 d
Visit Type	Screening and VACCINATION[f]	ALL PARTICIPANTS	SAFETY SUBSET ONLY	IMMUNO SUBSET ONLY	IMMUNO SUBSET ONLY	ALL PARTICIPANTS	ALL PARTICIPANTS	IMMUNO SUBSET ONLY	ALL PARTICIPANTS
Written informed consent[g, h]	❶								
Inclusion/exclusion criteria	❶								
Contraindications to vaccination	❶								
Demographics	❶								
Relevant medical history[i]	❶								
Smoking status	❶								
Pre vaccination medications[j]	❶								
Check for SARS CoV 2 and seasonal influenza vaccination[k]	*Continuous*								
Vital signs (including height and weight)[l]	❶								
Randomization[m, n]	❶								
eDiary training and distribution[p]	●	●[q]							
RiiQ™ v2[o]	❶					●[o]	●		●
EQ 5D 5L[o]	❶					●[o]	●		●
Patient Global Impression of Health (PGI H)[o]	❶					●[o]	●		●
Nasal swab kit training and distribution	●	●[q]							
Vaccination	●								
15 Minute post vaccination observation[r]	●								
ARI surveillance assessment[w]	*Twice per week via the eDiary from vaccination until Month 24*								
SAEs[s, t]	*Continuous for 6 months after vaccination*								
AESIs[t]	*Continuous for 6 months after vaccination*								

**Visit #**	**1**	**2**	**2a ☎**	**2b**	**2c[a]**	**3[a,b] ☎**	**4[d]**	**4a[d]**	**5[d]**
**Visit Timing**	**Vac 1**	**Vac 1 +14 d**	**Vac 1 +28 d**	**Vac 1 +84 d**	**Vac 1 +24 we**	**End 1st RSV Season[c] or Month 6**	**Month 12**	**Month 18**	**Month 24**
**Visit Week**	**0**	**2**	**4**	**12**	**24**	**26**	**52**	**76**	**104**
**Visit Day**	**1**	**15[e]**	**29[e]**	**85[e]**	**169[e]**	**182**	**365[e]**	**533[e]**	**730[e]**
**Visit Window**		**+3 d**	**+3 d**	**±7 d**	**±14 d**	**-7/+14 d**	**-7/+14 d**	**±14 d**	**-7/+14 d**
**Visit Type**	Screening and VACCINATION[f]	ALL PARTICIPANTS	SAFETY SUBSET ONLY	IMMUNO SUBSET ONLY	IMMUNO SUBSET ONLY	ALL PARTICIPANTS	ALL PARTICIPANTS	IMMUNO SUBSET ONLY	ALL PARTICIPANTS
AEs leading to discontinuations[s]	*Continuous*								
COVID 19 cases[u]	*Continuous*								
Concomitant medications[v]	*Continuous*								
Humoral immunity sample, mL	❶[x] 15	●[x] 15					●[x] 15		
Whole blood sample for immunological analysis, mL	❶ 2	● 2					● 2		
IMMUNO SUBSET ONLY:									
Humoral immunity sample, mL	❶[x] 20	●[x]20		● 20	● 20		●[x]20	●[x]20	
Cellular immunity sample, mL	❶ 40	● 40		● 40	● 40		● 40	● 40	
Nasosorption sample (SAM)	❶	●					●		
SAFETY SUBSET ONLY:									
Thermometer training and distribution	●								
Ruler training and distribution	●								
Solicited AE recording[y]	●								
Unsolicited AE recording[z]	*Continuous*								
Concomitant medications	*Continuous*								
Vital signs[aa]	❶●								
eDiary review of solicited AEs by study staff		●							
Blood Draw Volumes: IMMUNO SUBSET PARTICIPANTS									
Approx. daily blood draw, mL	62	62		60	60		62	60	
Approx. cumulative blood draw, mL	62	124	124	184	244	244	306	366	**366**
Blood Draw Volumes: NON-IMMUNO SUBSET PARTICIPANTS									
Approx. daily blood draw, mL	17	17					17		
Approx. cumulative blood draw, mL	17	34	34	34	34	34	51	51	**51**

❶ pre-dose

Note: At any clinic visit, an abbreviated, symptom directed physical examination may be performed if deemed necessary by the investigator based on any clinically relevant issues, clinically relevant symptoms, and medical history.

Footnotes:

a. For Immuno Subset participants, where the timing of the 24-week post-vaccination visits and end of first RSV season or Month 6 (Day 182) visits may overlap, procedures can be combined.

b. For the Northern Hemisphere (NH), the end of the first RSV season is defined as 15 April 2022. For the Southern Hemisphere (SH), the end of the first RSV season is defined as 30 September 2022. This visit (Visit 3) is then referred to as "End of first RSV season" visit. End of first RSV season procedures may be conducted on the same day as Visit 1, or before Visits 2, 2a, 2b, and 2c depending on the date of enrollment of the participant. If a participant is enrolled after the maximum window of the "End of first RSV season" visit (ie, after 29 Apr 2022 for NH and after 14 Oct 2022 for SH), only a "Month 6" (182 days, -7/+14 days post vaccination) safety follow-up call is required to collect information on SAEs and potential AESIs.

c. If the "End of first RSV season" visit for a participant is at least 6 months (or more) post-vaccination, the SAE and potential AESI safety follow-up is collected at the "End of first RSV season" visit. In this scenario, **no additional Safety follow-up is required at Month 6 (Day 182 -7/+14 days)**. If the "End of first RSV season" visit for a participant occurs less than 6 months (-7 days) post-vaccination, **a separate Safety follow-up call is required (182 days, -7/+14 days post vaccination)** to collect additional information on SAEs and potential AESIs. This Safety follow-up call is in addition to the "End of first RSV season" visit.

d. Visit 4 through Visit 5 will only take place if the study is not discontinued earlier. At Visit 5 (Month 24), participants may be required to come to the site to return their eDevices, if applicable.

e. The timings of the post-vaccination visits will be determined relative to the actual day of vaccination using the "Visit Timing" from the upper part of the table.

f. Screening and vaccination may be split into 2 visits after consultation with the sponsor or its delegate. Every effort should be made for split visits to occur preferably within 3 to 5 days and no later than 14 days, and pre-vaccination vital signs should be repeated on the day of vaccination if this visit is split.

g. Signing of the ICF must be done before any study-related activity. Participants in the Immuno Subset and/or Safety Subset will need to consent for the additional study procedures in these subsets.

h. Participants (from the US only) will be asked for optional consent to allow access to their medical data (electronic health records, claims, laboratory data from other care settings) from 5 years prior to study enrollment until 5 years after study completion utilizing tokenization and matching procedures (see Section 8.7). Participants will be informed that consent can be withdrawn at any given time. The sponsor will then remove the token generated and any associated linked real-world data.

i. Only relevant medical history is to be collected, in particular: congenital abnormalities, history of cancer, history of immunodeficiency or conditions treated with immunomodulators, major psychiatric illness, major cardiovascular or lung diseases, type 2 diabetes and chronic kidney diseases (CKD), history of an allergy to vaccination, ongoing comorbidities, history of any comorbidity known to be associated with an increased risk of progression to severe RSV disease, history of hepatitis B or hepatitis C infection, and history of any thrombotic events and/or thrombocytopenia. Participants with stable/well-controlled HIV infection are allowed to enroll in the study. These participants will be encouraged to have HIV RNA viral load and CD4 cell count assessed at least twice a year and to provide these data for inclusion in the eCRF.

j. Therapies administered up to 30 days before vaccination will be recorded.

k. Any seasonal influenza vaccination must occur at least 14 days before or after study vaccination. Live-attenuated SARS-CoV-2 vaccines and viral-vectored SARS-CoV-2 vaccines must occur at least 28 days before or after study vaccination; non-live SARS-CoV-2 vaccines must occur at least 14 days before or after study vaccination. If seasonal influenza vaccination or SARS-CoV-2 vaccination occurs after Day 15, the information will be recorded at the next site contact or at the end of the RSV season, whichever comes first.

l. Vital signs (sitting systolic and diastolic blood pressure, heart rate, respiratory rate, oxygen saturation [after at least 5 minutes rest] weight and body temperature). At non-vaccination visits, vital signs will be measured if deemed necessary by the investigator. Height will only be collected at screening.

m. A cap will be installed to ensure that ~50% of participants enrolled in the Immuno Subset and ~50% of participants enrolled in the Safety Subset will be at increased risk of severe RSV disease, ie, have underlying chronic heart disease (congestive heart failure [CHF], coronary artery disease [eg, angina pectoris, ischemic cardiomyopathy, history of myocardial infarct, history of coronary artery bypass graft, or coronary artery stent]) and chronic lung disease (asthma and chronic obstructive pulmonary disease [COPD]). A cap will also be installed to ensure that ~50% of participants in these subsets will be 75 years or older.
n. Participants will be randomized at Day 1 in a 2:1 ratio to receive active vaccine (Group 1) or placebo (Group 2) (see Section 6.3).
o. The RiiQ, EQ-5D-5L, and the PGI-H will be completed by the participant in the eDiary. The questionnaires need to be completed at the "End of first RSV season" visit but not at the Month 6 (Day 182) follow-up.
p. Participants may use their own eDevice (or desktop computer, where applicable) using a study-specific application instead if their device (smartphone or tablet) is compatible. eDevices provided to the participant should be returned to the site at the end of the study.
q. Participants will be re-trained on how to use the eDiary, if needed, and on how to collect a nasal swab. Participants will be provided with 2 nasal swab kits.
r. All participants will be closely observed for a minimum of 15 minutes post-vaccination to monitor for the development of any acute reactogenicity. This post-vaccination observation period should be extended for participants whom in the investigator's opinion would require further observation or for any occurrence of a localized reaction or (minor) systemic symptoms during the post-vaccination observation that require monitoring for any progression.
s. All (S)AEs and special reporting situations related to study procedures or to non-investigational (concomitant) Janssen products will be collected from ICF signature onwards until the end of the study for all participants. All other SAEs will be collected from administration of study vaccine (Day 1) until 6 months after vaccination. AEs leading to discontinuation from the study will be collected for the duration of the study.
t. AESIs, including potential AESIs, are to be reported to the sponsor within 24 hours of awareness from the moment of vaccination until 6 months after vaccination (See Section 8.5.6).
u. All COVID-19 cases will be collected for all participants for the duration of the study. COVID-19 cases reported by the participant to the investigator and any ARI positive for SARS-CoV-2 will be reported in the eCRF by the investigator.
v. Concomitant medications will be collected for all participants for the duration of the study when associated with any SAEs and AESIs and/or with any AEs leading to discontinuation from the study.
w. ARI surveillance will be via the eDiary which participants must complete at least twice weekly. Procedures in the event of an ARI are described in the Schedule of Activities for participants with an ARI episode.
x. Aliquots of serum samples collected for immunogenicity tests can be reconverted for participant's safety purposes upon sponsor request. Please refer to Table 4 for a non-exhaustive list of tests that may be requested to be performed on these samples in case of potential AESI reporting.
y. Solicited local and solicited systemic AEs will be documented by study site personnel following the 15 minutes post-vaccination observation period and will be collected via the eDiary from vaccination until 7 days after vaccination for Safety Subset participants. If a solicited local or systemic AE is not resolved within 7 days after vaccination (Day 8), safety follow-up should be performed until the symptom resolves. The study-site personnel will be instructed to record the date of last symptoms and maximum severity until resolution in the source document and eCRF.
z. All AEs and special reporting situations related to study procedures or to non-investigational (concomitant) Janssen products will be reported from ICF signature onwards until the end of the study for all participants. All other AEs (unsolicited) and special reporting situations will be reported from vaccination through the following 28 days for Safety Subset participants only.
aa. Vital signs (sitting systolic and diastolic blood pressure, heart rate, respiratory rate, oxygen saturation [after at least 5 minutes rest] and body temperature) will be obtained prior to vaccination and at the end of the observation period. Emerging vital sign abnormalities will be summarized based on the planned visits. Clinically relevant abnormalities for systolic and diastolic blood pressure, heart rate, respiratory rate, and oxygen saturation collected during unscheduled visits will be documented as AEs.

AE=adverse event, AESI=adverse event of special interest, ARI=acute respiratory infection, d=day, eCRF=electronic case report form, EQ-5D-5L= EuroQoL, 5-Dimension, 5-Level, HIV=human immunodeficiency virus, ICF=informed consent form, NH=Northern Hemisphere, PGI-H=Patient Global Impression of Health, RiiQ=Respiratory Infection Intensity and Impact Questionnaire (RiiQ™) v2, RNA=ribonucleic acid, RSV=respiratory syncytial virus, SAE=serious adverse event, SAM=synthetic absorptive matrix, SH-Southern Hemisphere, vac=vaccination, we=week

1.3.2. Schedule of Activities – Assessments for Participants with an ARI Episode

Timing relative to ARI onset[a]	ARI onset (ARI Day 1)	ARI Day 1-2	ARI Day 3-5	Daily after ARI onset[b]	ARI Day 29 (±7 days)
Location	Home	Home	Site[c]	Home	Site or ☎[d]
Participant to contact study site as soon as symptoms of possible ARI occur or site to contact participant if any ARI symptom is recorded in eDiary	●				
Nasal swab (collected by the participant at home)		●[e]			
Nasal swab (collected by study staff)			●[f,g]		
Sputum sample, when possible (collected by study staff)			●		
Nasal swab kit distribution and training (if needed)			●		
Vital signs[h]			●		
Serology blood sample to assess immune correlates associated with infection [j]			15		
Whole blood PAXgene sample[i, j] mL			2.5		
Whole blood sample with PROT1 stabilizer[i, j] mL			4		
eDiary (completed by the participant)[k]					
RiiQ™v2	---------- Daily ----------				●
EQ-5D-5L	---------- Daily ----------				●
Patient Global Impression of Health (PGI-H)	---------- Daily ----------				●
Patient Global Impression of Severity (PGI-S)	---------- Daily ----------				●
Patient Global Impression of Change (PGI-C)		---------- Daily ----------			●
Return to Usual Health question		---------- Daily ----------			●
MRU questionnaire (collected by interview with participant)					●
Days missed from work question (collected by interview with participant)					●
ARIs and complications of ARIs[l]	---------- Continuous ----------				
Capture medical information from medical visits for ARIs and complications of ARIs (medically-attended ARI Form)[m]	---------- Continuous ----------				
Concomitant medications associated with ARIs and their complications [n]	---------- Continuous ----------				

Footnotes:

a. For participants with a positive SARS-CoV-2 test and a negative test for RSV, hMPV, and influenza, the collection of ARI data by the study site will be stopped for the ARI episode. The participant will be informed by telephone or telemedicine contact that they can stop filling out the daily ARI episode questionnaires, and the site will end the ARI episode and return the participant to ARI surveillance with twice weekly reminders. Participants who have a positive SARS-CoV-2 RT-PCR test as part of the study procedures should be referred to their own healthcare provider for a locally approved diagnostic test and further management, in accordance with local/site specific guidelines.

b. Daily until resolution (defined as 2 consecutive days with no symptoms listed on the RiiQ Symptom Scale, or, for participants who have RiiQ symptoms at baseline (assessed pre-vaccination), 2 consecutive days where all symptoms on the RiiQ Symptom Scale have returned to the same severity level as reported at baseline (or lower) or until the RT-PCR results (from ARI Day 1-2 and ARI Day 3-5 samples) are negative for RSV, hMPV and influenza upon which participants will be informed by the site that they can stop filling out the daily ARI episode questionnaires. In the setting that the site confirms that the reported symptoms do not qualify as an ARI episode, or that an ARI episode has ended despite long-term ongoing symptoms above baseline, the site staff can manually end an ARI

episode. For all ARIs, a start date of the ARI should be entered into eCRF; for all RSV, hMPV, or influenza-mediated ARIs, an end date of the ARI should be entered in the eCRF. For ARIs that are RSV, hMPV and influenza negative, the date that the site ends the ARI (after the RT-PCR test results are known) should also be entered in the eCRF.

c. Home visits by a healthcare professional are allowed in the event that the participant is unable to come to the site.

d. Participants will be asked to return to the site if a sputum sample was provided by the participant at ARI Day 3-5 visit, regardless of the swab RT-PCR test results, AND/OR at least one of the nasal swab samples collected could not be tested at the study site with the BioFire Filmarray Respiratory panel (RT-PCR test) and had to be shipped for a central laboratory test AND/OR at least one nasal swab sample had a positive RT-PCR result for either RSV, hMPV, or influenza when tested at the study site.

e. The nasal swab collected by the participant at home should preferably be taken between 12 to 24 hours after onset of symptoms. Participants who are under self-isolation because of a (possible) positive test for SARS-CoV-2 will not come to the site for ARI Day 3-5 assessments but will self-collect the nasal swab. In this circumstance the other planned assessments for ARI Day 3-5 visit may be omitted.

f. The nasal swab collected by the study staff at the site or at home should ideally be taken at least 24 hours after the nasal swab collected by the participant at home.

g. For participants hospitalized with symptoms of an ARI, confirmation of RSV infection using a locally approved RT-PCR test at the hospital laboratory can also be used. These results will be used in the primary analysis only if approved by an independent expert with expertise in diagnostic testing. The approval of the RT-PCR results by the expert will be based on the specifications of the test performed, the qualifications of the testing laboratory and the procedures followed in the laboratory.

h. Vital signs (sitting systolic and diastolic blood pressure, heart rate, respiratory rate, oxygen saturation [after at least 5 minutes rest] and body temperature).

i. For exploration of biomarkers correlating with RSV infection.

j. To reduce the burden on the participant and to avoid collection of blood samples (ie, serology blood sample, whole blood PAXgene sample and whole blood sample with PROT1 stabilizer), not related to an RSV/hMPV/influenza related ARI, participants and investigators can choose the preferred option during each Day 3-5 visit between: 1) Collecting blood samples before the nasal swab samples are tested or in the setting where sputum sample is collected, independent of the RT-PCR results; and 2) Collecting blood samples after the nasal swab RT-PCR results are available, according to nasal swab test results, and for ARIs where there is no sputum sample collection. If this is the preferred option by the participant and investigator, the participant will wait at the site for the time needed to test the swab samples (estimated time: 2 hours). In this scenario, if the swab samples are negative for RSV, hMPV and influenza AND if no sputum sample can be collected, the blood samples collection at the ARI Day 3-5 timepoint can be omitted. If the swab samples are positive for RSV, hMPV or influenza OR if sputum sample is collected, the blood samples collection must be performed. Whenever a sputum sample is collected during the ARI Day 3-5 visit, the blood samples must still be collected as the RT-PCR test on the sputum sample will be performed at the central laboratory and not on site.

k. Participants complete the eDiary to respond to the ARI Surveillance Assessment, the RiiQ Symptom Scale and the RiiQ Impact on Daily Activity Scale, the EQ-5D-5L, PGI-H, the PGI-S, the PGI-C, and the Return to Usual Health question once daily (ie, either at home or during the site visit, if applicable). (Note: the PGI-C scale and the Return to Usual Health question will not be completed on ARI Day 1). Participants with a RT-PCR positive for RSV, hMPV, or influenza-mediated ARI will continue filling out the questionnaires until resolution of the ARI episode. Participants with negative RT-PCR results for RSV, hMPV, or influenza will be informed by telephone or telemedicine that they can stop filling out the questionnaires and the site will end the ARI episode and return the participant to ARI surveillance. Participants with a sputum sample taken and participants with at least one nasal swab that cannot be tested at the site with the BioFire Filmarray Respiratory panel will follow the same procedures as participants with a RT-PCR positive for RSV, hMPV, or influenza-mediated ARI. Further details are provided in Appendix 7: ARI Surveillance Assessment.

l. All ARIs will be captured in the eCRF for all participants who experience an ARI episode for the duration of the study. All complications (including pneumonia), hospitalizations, concomitant medications, and MRU data associated with medical encounters, and whether they were related to ARIs will be captured in the eCRF for all participants who experience an ARI episode for the duration of the study. ARIs and complications related to ARIs that classify as SAEs will be captured in all participants and reported as SAEs in the eCRF continuously from the time of vaccination until 6 months after vaccination. In addition, ARIs and complications related to ARIs that do not classify as SAEs will be reported as AEs in the eCRF from the time of vaccination through the following 28 days for Safety Subset

participants only.

m. For medically-attended RT-PCR positive for RSV, hMPV, or influenza-mediated ARIs, including those resulting in hospitalization, a standard question list will be provided (Appendix 6: Medically-attended ARI Form) to collect additional information on any other diagnostic tests (eg, chest x-rays, spirometry, pulmonary function tests, etc.) or on any interventions during the clinical course of the ARI.

n. Concomitant medications associated with RSV, hMPV, and influenza-mediated ARI episodes and with complications of RSV, hMPV, and influenza-mediated ARIs will be recorded for all participants for the duration of the study.

AE=adverse event, ARI=acute respiratory infection, eCRF=electronic case report form, EQ-5D-5L= EuroQoL, 5-Dimension, 5-Level, hMPV=human metapneumovirus, MRU=Medical Resource Utilization, PGI-C=Patient Global impression of Change, PGI-H=Patient Global Impression of Health, PGI-S=Patient Global Impression of Severity, RiiQ=Respiratory Infection Intensity and Impact Questionnaire (RiiQ™) v2, RNA=ribonucleic acid, RSV=respiratory syncytial virus, RT-PCR=reverse transcriptase polymerase chain reaction, SAE=serious adverse event

2. INTRODUCTION

The respiratory syncytial virus (RSV) vaccine that will be investigated in this study, Ad26/protein preF RSV vaccine, includes 2 components that are being mixed prior to administration as a single intramuscular (IM) injection:

- Ad26.RSV.preF (JNJ-64400141), a replication-incompetent adenovirus serotype 26 (Ad26) containing a deoxyribonucleic acid (DNA) transgene encoding the pre-fusion conformation-stabilized F protein (preF) derived from the RSV A2 strain, and
- RSV preF protein (JNJ-64213175), a pre-fusion conformation-stabilized F protein derived from the RSV A2 strain.

For the most comprehensive nonclinical and clinical information regarding Ad26/protein preF RSV vaccine, refer to the latest version of the Investigator's Brochure (IB) for Ad26/protein preF RSV vaccine.

The term 'study vaccine' throughout the protocol refers to the Ad26/protein preF RSV vaccine or placebo as defined in Section 6.1, Study Vaccine Administration.

The term 'sponsor' throughout the protocol refers to the entities listed in the Contact Information page, which will be provided as a separate document.

The term 'participant' throughout the protocol refers to the common term 'subject'.

2.1. Study Rationale

Following successful demonstration of efficacy in preventing lower respiratory tract disease (LRTD) due to RSV after one season in the Phase 2b proof-of-concept efficacy study VAC18193RSV2001, the current study will further investigate the efficacy of Ad26/protein preF RSV vaccine in participants aged 60 years and older, with surveillance for acute respiratory infection (ARI) from the time of study vaccination until 24 months after vaccination. The primary endpoint is based on a case definition that was demonstrated to be relevant in study VAC18193RSV2001 as it encompassed both moderate and severe forms of RSV LRTD (see Section 2.2).

The primary objective of this study is to demonstrate efficacy of the Ad26.RSV.preF-based study vaccine in the prevention of reverse transcriptase polymerase chain reaction (RT-PCR)-confirmed RSV-mediated LRTD when compared to placebo in adults aged 60 years and above, as outlined in Section 9.5, Planned Analyses. Recruitment will be conducted both in the Northern Hemisphere (NH) and Southern Hemisphere (SH). Interim analyses to evaluate the primary objective will be performed by the IDMC (see Section 9.5.1).

2.2. Background

RSV is an important cause of serious respiratory infections in adults aged 60 years and older, in immunocompromised individuals, and in individuals with underlying chronic cardiopulmonary conditions (Falsey 2005). Exact numbers on the burden of RSV disease in adults aged 60 years

and older are limited. In long-term care facilities, RSV has been estimated to infect 5% to 10% of the residents per year, with significant rates of pneumonia (10% to 20%) and death (2% to 5%) as a consequence (Falsey 2000).

In the United States (US), about 177,000 hospital admissions and 10,000 to 14,000 deaths per year are due to severe RSV infections in adults aged 65 years and older (Thompson 2003). In Europe (data available from 14 studies for 8 countries), RSV accounted for 1% to 11% of all influenza-like illness in patients of all ages (Htar 2020). In adults (aged 50 years and older), RSV accounted for 2% to 18% of influenza-like illness. Even though data on the burden of RSV disease in older adults remain scarce in Europe, statistical modelling indicates that RSV is associated with substantial hospitalization and mortality in this population (Fleming 2015, Hardelid 2013, Jansen 2007, van Asten 2012). In Japan, a prospective cohort study of adults aged ≥65 showed that the attack rate of RSV infection is 2.4% (study VAC18193RSV0002), and the rate is comparable to other countries (Falsey 2000). These data support the importance of developing a safe and effective vaccine for certain adult populations, such as those aged ≥60 years. Currently, there are no licensed vaccines or treatments available for RSV in the adult population.

Ad26.RSV.preF and RSV preF Protein Nonclinical Data

Both Ad26.RSV.preF and the RSV preF protein have been evaluated for induction of immune responses in multiple species (mice, cotton rats, bovine calves [Ad26.RSV.preF only], and non-human primates [NHPs]). Ad26.RSV.preF induced RSV-specific cellular and humoral immune responses, whereas the RSV preF protein mainly induced humoral immunity. The combination of both vaccine components improved the humoral and/or cellular immune response over each vaccine component individually.

Efficacy of the Ad26.RSV.preF-induced immune response was shown in mice, cotton rats, and the bovine RSV challenge model, and it was shown for the RSV preF protein alone and the combination of both vaccine components in the cotton rat RSV challenge model. In the cotton rat RSV challenge model, efficacy was higher in Ad26.RSV.preF vaccinated animals compared to the RSV preF protein. A combination of Ad26.RSV.preF and the RSV preF protein further improved efficacy in the upper respiratory tract.

Ad26.RSV.preF and RSV preF Protein Clinical Data

The clinical studies completed or currently ongoing are included in Table 1.

Table 1: Clinical Studies with RSV PreF Protein and/or Ad26.RSV.preF in the Adult Population

Study Identifier	Clinical Phase	Description	Vaccine
Completed			
VAC18193RSV1003	1	First-in-human	Ad26.RSV.preF
VAC18193RSV1005	1	Shedding assessment	Ad26.RSV.preF
VAC18193RSV2002	2a	Challenge study	Ad26.RSV.preF
VAC18193RSV2003	2a	Influenza vaccine co-administration	Ad26.RSV.preF
Ongoing			
VAC18193RSV1006	1	Phase 1 study in Japanese	Ad26/protein preF RSV vaccine
VAC18193RSV1004*	1/2a	Regimen selection	Ad26.RSV.preF<br>RSV preF protein<br>Ad26/protein preF RSV vaccine
VAC18193RSV2005	2a	Dose ranging	Ad26/protein preF RSV vaccine
VAC18193RSV2001*	2b	Proof-of-concept	Ad26/protein preF RSV vaccine

* Primary analysis has been completed

The results from the first-in-human study VAC18193RSV1003 confirmed the immunogenicity of a single Ad26.RSV.preF immunization. The 1×10^{11} vp (viral particles) dose of Ad26.RSV.preF was subsequently evaluated in the human challenge study VAC18193RSV2002. These results showed a reduction in the nasal wash viral load compared to placebo. The reduction was associated with a major reduction in the total clinical symptom score.

The immunological benefit of combining Ad26.RSV.preF with RSV preF protein was first shown in study VAC18193RSV1004. A significant increase in virus neutralizing titers was observed at 28 days post dose 1 in the groups combining Ad26.RSV.preF and RSV preF protein compared to Ad26.RSV.preF 1×10^{11} vp alone. Based on this study, a vaccine combining Ad26.RSV.preF with RSV preF protein was selected for future clinical development.

This vaccine is currently being tested in study VAC18193RSV2001, a multicenter, randomized, double-blind, placebo-controlled Phase 2b proof-of-concept efficacy study in ~5,800 participants aged ≥65 years, with and without comorbidities. The study is designed to demonstrate efficacy of the Ad26.RSV.preF/ RSV preF protein vaccine (1×10^{11} vp/150 µg) for at least 1 of the 3 case definitions of RSV-mediated LRTD. The primary analysis after the end of the first RSV season showed high, statistically significant VE against RSV-mediated LRTD for all case definitions, ranging from 80% for the most severe endpoint (case definition #1) to 69.8% for the mildest endpoint (case definition #3). The vaccine was safe and well-tolerated. Based on these data, case definition #1 (see Section 8.2.1) was selected for use as primary endpoint in the present Phase 3 study. Study VAC18193RSV2001 is currently ongoing to evaluate the duration of protection and immune responses beyond 1 year in a subset of participants.

Clinical Safety Experience With Ad26-based Vaccines

Safety data of Ad26-vectored vaccines from the adenoviral vaccine safety database (Advac Safety Database 2022), including vaccines against Ebola virus (Ad26.ZEBOV), HIV (Ad26.ENVA.01, Ad26.Mos.HIV, and Ad26.Mos4.HIV), malaria (Ad26.CS.01), RSV (Ad26.RSV.FA2 and

Ad26.RSV.preF), filovirus (Ad26.Filo), zikavirus (Ad26.ZIKV.001), and HPV (Ad26.HPV16 and Ad26.HPV18) have been evaluated in adults.

All the above Ad26-based vaccines were found to be well tolerated in adults, without significant safety issues identified. Several other studies are currently ongoing, and no safety concerns have been raised to date.

Thrombosis With Thrombocytopenia Syndrome

Thrombosis with thrombocytopenia syndrome (TTS), in some cases accompanied by internal bleeding, has been observed very rarely following vaccination with the Janssen COVID-19 (Ad26.COV2.S) vaccine. Reports of vaccine-induced TTS, also known as vaccine-induced immune thrombotic thrombocytopenia (VITT), include severe cases of venous thrombosis at unusual sites such as cerebral venous sinus thrombosis (CVST), splanchnic vein thrombosis and arterial thrombosis, in combination with thrombocytopenia. The associated symptoms began approximately 1 to 2 weeks after vaccination, mostly in women under 60 years of age. Vaccine-induced TTS can be fatal. The exact pathophysiology of the syndrome is unclear. As of 21 July 2022, no cases of vaccine-induced TTS have been identified following vaccination with any other Janssen Ad26-based vaccines. Participants should be instructed to seek immediate medical attention if they develop symptoms such as shortness of breath, chest pain, leg swelling, persistent abdominal pain, severe or persistent headaches, blurred vision, skin bruising or petechiae beyond the site of vaccination.

2.3. Benefit-Risk Assessment

More detailed information about the known and expected benefits and risks of Ad26/protein preF RSV vaccine may be found in the Investigator's Brochure for Ad26/protein preF RSV vaccine.

2.3.1. Risks of Study Participation

The following potential risks for Ad26/protein preF RSV vaccine will be monitored during this study:

Risks Related to Ad26/Protein preF RSV Vaccine

As of October 27, 2021, the Ad26/protein preF RSV vaccine has been studied for its safety and/or its ability to prevent RSV disease in ongoing trials in 8,499 participants with and without underlying medical conditions, primarily in adults aged 60 years and above. Of the above participants, 137 were aged 18-59 years of age. The vaccine is being further studied in several ongoing trials.

In adults, the most commonly reported local symptom was injection site pain/tenderness and swelling (mild to moderate). The reported body symptoms were mostly mild to moderate; the most frequently seen were fatigue, muscle pain, headache, chills, joint pain and nausea. In all participants, these symptoms were short-lived and resolved within days. Results from the trials show that the vaccine is tolerated well and there were no safety concerns.

General Risks Related to Vaccination

In general, IM injection may cause local itching, warmth, pain, tenderness, erythema or redness, induration, swelling, arm discomfort, or bruising of the skin. Participants may exhibit general signs and symptoms associated with IM injection of a vaccine and/or placebo, including fever, chills, rash, myalgia, nausea or vomiting, headache, dizziness, arthralgia, general itching, and fatigue. These side effects will be monitored, but they are generally short-term and do not require treatment.

Syncope can occur in association with administration of injectable vaccines. Syncope can be accompanied by falls. Procedures should be in place to avoid falling injury. If syncope develops, participants should be observed until symptoms resolve. Fear of injection might lead to fainting and fast breathing.

Participants may have an allergic reaction to the vaccination. An allergic reaction may cause a rash, urticaria, or even anaphylaxis. Severe reactions are rare. Participants with a known or suspected allergy, or with a history of anaphylaxis or other serious adverse reactions to vaccines or their excipients (including specifically the excipients of the study vaccine) will be excluded from the study.

After vaccination, participants will remain at the study site for at least 15 minutes and will be closely observed by the study staff. Necessary emergency equipment and medications must be available in the clinic to treat severe allergic reactions.

Pregnancy and Birth Control

The effect of the Ad26/protein preF RSV vaccine on a fetus or nursing baby is unknown. Participants may therefore only participate if they are postmenopausal (defined as no menses for 12 months without an alternative medical cause) and not intending to conceive by any methods. Participants who are surgically sterile are also eligible for the study. Follow-up information regarding the outcome of the pregnancy will be required.

Because the effect on sperm is unknown, participants must inform the study-site personnel if their partner becomes pregnant during the study. Follow-up information regarding the outcome of the pregnancy will be requested upon the consent provided by the partner.

Participants with Immunosuppression/Reduced Immune Response

Participants with abnormal function of the immune system will be excluded from the study. Limited evidence indicates that inactivated vaccines (or nonreplicating viral vaccines) generally have the same safety profile in immunocompromised patients as in immunocompetent individuals. However, the magnitude, breadth, and persistence of the immune response to vaccination may be reduced or absent in immunocompromised persons.

Risks from Collection of Nasal Swabs and Nasosorption Samples

Collection of a nasal swab may cause a nosebleed.

Nasosorption sampling is more comfortable and less invasive than using a conventional swab.

Risks from Blood Draws

Blood draws may cause pain, tenderness, bruising, bleeding, dizziness, vasovagal response, syncope, and rarely, infection at the site where the blood is taken. Participants with contraindications to IM injections and blood draws (eg, bleeding disorders) will be excluded.

Concomitant Vaccination

Concomitant vaccination might have an influence on both the safety profile and immunogenicity of the Ad26/protein preF RSV vaccine. Likewise, the Ad26/protein preF RSV vaccine might have an influence on both the safety profile and immunogenicity of any concomitant vaccination. As a result, licensed live attenuated vaccines should be given at least 28 days before or after study vaccination. Other licensed (not live) vaccines (eg, tetanus, hepatitis A, hepatitis B, rabies) should be given at least 14 days before or after study vaccination to avoid potential confusion of adverse reactions and potential immune interference. If a vaccine is indicated in a postexposure setting (eg, rabies or tetanus), it must take priority over the study vaccine.

For SARS-CoV-2 vaccines either licensed or available under Emergency Use Authorization: live attenuated vaccines should be given at least 28 days before or after study vaccination; non-live vaccines should be given at least 14 days before or after study vaccination. A viral-vectored SARS-CoV-2 vaccine is not to be given within 28 days before or after study vaccination.

Unknown Risks

There may be other risks that are not known. If any significant new risks are identified, the investigators and participants will be informed.

2.3.2. Benefits of Study Participation

Participants may benefit from clinical testing.

The clinical benefits of Ad26/protein preF RSV vaccine are yet to be established.

The Ad26/protein preF RSV vaccine is under development for prophylaxis of RSV and VE is being evaluated in ongoing studies. Results from the primary analysis of study VAC18193RSV2001 with approximately 5,800 participants showed the potential for the vaccine to prevent lower respiratory tract disease caused by RSV in participants 65 years and older.

2.3.3. Benefit-Risk Assessment of Study Participation

Based on the available data and proposed safety measures, the overall benefit-risk assessment for this clinical study is considered acceptable for the following reasons:

- The primary analysis after the end of the first RSV season in the ongoing proof-of-concept efficacy study VAC18193RSV2001 showed high, statistically significant VE of Ad26.RSV.preF/ RSV preF protein vaccine (1×10^{11} vp/150 µg) against all RSV for all case definitions, ranging from 80% for the most severe endpoint to 69.8% for the mildest endpoint. The vaccine was safe and well-tolerated (see Section 2.2).
- Only participants who meet all inclusion criteria and none of the exclusion criteria (specified in Section 5, Study Population) will be allowed to participate in this study. The selection criteria include adequate provisions to minimize the risk and to protect the well-being of the participants in the study.
- Safety will be closely monitored throughout the study:

  In general, safety evaluations will be performed at scheduled visits during the study, as indicated in the Schedule of Activities.

  After vaccination, participants will remain at the study site for at least 15 minutes and will be closely observed by the study staff. Necessary emergency equipment and medications must be available in the clinic to treat severe allergic reactions.

- Participants in the Safety Subset will use an eDiary to document solicited signs and symptoms. Details are provided in Section 8.4, Safety Assessments.

  The investigator or designee will document unsolicited AEs as indicated in Section 8.4, Safety Assessments, Section 8.5, Adverse Events, Serious Adverse Events, and Other Safety Reporting, and Appendix 3: Adverse Events, Serious Adverse Events, Adverse Event of Special Interest, Product Quality Complaints, and Other Safety Reporting: Definitions and Procedures for Recording, Evaluating, Follow-up, and Reporting .

  Any clinically significant abnormalities (including those persisting at the end of the study or upon early withdrawal) will be followed by the investigator until resolution or until clinically stable.

- Safety measures are included in this protocol to minimize the potential risk to participants, including the following:

  Temporary contraindications to study vaccination are included in Section 5.5, Criteria for Temporarily Delaying Administration of Study Vaccine.

3. OBJECTIVES AND ENDPOINTS

The primary objective of this study is to demonstrate efficacy of the Ad26.RSV.preF-based study vaccine in the prevention of RT-PCR-confirmed RSV-mediated LRTD when compared to placebo in adults aged 60 years and above. Interim analyses to evaluate the primary objective will be performed by the Independent Data Monitoring Committee (IDMC). The primary, secondary and exploratory objectives and endpoints of the study are presented below.

Primary Objective, Secondary Efficacy Objectives and Exploratory Objectives

Objectives	Endpoints
**PRIMARY**	
• To demonstrate the efficacy of the active Ad26.RSV.preF-based study vaccine in the prevention of RT-PCR-confirmed RSV-mediated LRTD when compared to placebo in adults aged 60 years and above	• First occurrence of RT-PCR-confirmed, RSV-mediated LRTD with onset at least 14 days after dosing of study vaccine
**CONFIRMATORY SECONDARY***	
• To demonstrate the efficacy of active study vaccine in the prevention of any RT-PCR-confirmed RSV-mediated acute respiratory infection (ARI) when compared to placebo	• First occurrence of any RT-PCR-confirmed RSV-mediated ARI with onset at least 14 days after dosing of study vaccine
• To demonstrate the efficacy of active study vaccine in the prevention of RT-PCR-confirmed RSV-mediated LRTD during the second year when compared to placebo in adults aged 60 years and above	• First occurrence of RT-PCR-confirmed RSV-mediated LRTD during the second year, with onset after study Day 365
• To demonstrate the efficacy of active study vaccine in the prevention of any RT-PCR-confirmed RSV-mediated ARI during the second year when compared to placebo	• First occurrence of any RT-PCR-confirmed RSV-mediated ARI during the second year, with onset after study Day 365
• To demonstrate the efficacy of active study vaccine in the prevention of predefined clinically relevant disease associated with RT-PCR-confirmed RSV-mediated ARI over the whole study when compared to placebo	• First occurrence of predefined clinically relevant disease associated with RT-PCR-confirmed RSV-mediated ARI over the whole study with onset at least 14 days after dosing of study vaccine
**NON-CONFIRMATORY SECONDARY**	
• To evaluate safety in terms of serious adverse events (SAEs) and adverse events of special interest (AESIs) until 6 months after vaccination	• Occurrence and relationship of vaccination to SAEs and AESIs until 6 months after vaccination
• In the Safety Subset and in subgroups of the Safety Subset (including but not limited to participants at increased risk of severe RSV disease), to evaluate the safety and reactogenicity in terms of solicited local and systemic adverse events (AEs) during 7 days after vaccination, and in terms of unsolicited AEs during 28 days after vaccination	• Occurrence, intensity, duration and relationship to vaccination of solicited local and systemic AEs during 7 days after vaccination and of unsolicited AEs during 28 days after vaccination
• In the Immuno Subset and in subgroups of the Immuno Subset (including but not limited to participants at increased risk of severe RSV disease), to evaluate the immunogenicity of active study vaccine when compared to placebo	• Characterization of the humoral and cellular immune responses in the Immuno Subset with emphasis on neutralizing and binding antibodies and antigen-specific cytokine production by T-cells

Objectives	Endpoints
• To assess the reduction of symptom severity in participants with an RT-PCR-confirmed RSV-mediated ARI when compared to placebo over the whole study	• In participants with an RT-PCR-confirmed RSV-mediated ARI over the whole study: the area under the curve (AUC) of the change from baseline in Respiratory Infection Intensity and Impact Questionnaire (RiiQ[a]) Total Symptom score
**EXPLORATORY**	
• To demonstrate the efficacy of active study vaccine in the prevention of RT-PCR-confirmed RSV-mediated LRTD during the first year when compared to placebo in adults aged 60 years and above	• First occurrence of RT-PCR-confirmed RSV-mediated LRTD during the first year, with onset at least 14 days after dosing of study vaccine, and prior to study Day 365
• To demonstrate the efficacy of active study vaccine in the prevention of RT-PCR-confirmed RSV-mediated ARI during the first year when compared to placebo	• First occurrence of RT-PCR-confirmed RSV-mediated ARI during the first year, with onset at least 14 days after dosing of study vaccine, and prior to study Day 365
• To explore the effect of active study vaccine on RSV A and B infection when compared to placebo	• Assessment of the RSV A and B viral load by quantitative RT-PCR
• To explore the efficacy of active study vaccine in the prevention of any RT-PCR-confirmed RSV-mediated LRTD caused by an RSV A and/or B strain when compared to placebo<br>○ during the first year<br>○ during the second year<br>○ over the whole study	• First occurrence of any RT-PCR-confirmed RSV-mediated LRTD caused by an RSV A strain and/or RSV B strain, respectively, during the considered years<br>○ with onset at least 14 days after dosing of study vaccine and prior to study Day 365 (first year comparison)<br>○ with onset after study Day 365 (second year comparison)<br>○ with onset at least 14 days after dosing of study vaccine (whole study comparison)
• To explore the efficacy of active study vaccine in the prevention of any RT-PCR-confirmed RSV-mediated ARI caused by an RSV A and/or B strain when compared to placebo<br>○ during the first year<br>○ during the second year<br>○ over the whole study	• First occurrence of any RT-PCR-confirmed RSV-mediated ARI caused by an RSV A strain and/or RSV B strain, respectively, during the considered years<br>○ with onset at least 14 days after dosing of study vaccine and prior to study Day 365 (first year comparison)<br>○ with onset after study Day 365 (second year comparison)<br>○ with onset at least 14 days after dosing of study vaccine (whole study comparison)

[a] The Respiratory Infection Intensity and Impact Questionnaire used in this study is the RiiQ Symptom Scale and the RiiQ Impact on Daily Activities Scale of the RiiQ™ Version 2 (hereafter referred to as RiiQ).

Objectives	Endpoints
• To explore the efficacy of active study vaccine in the prevention of at least mild, at least moderate, and at least severe RT-PCR-confirmed RSV LRTD as assessed by the Clinical Event Adjudication Committee (CEC) compared to placebo<br>○ during the first year<br>○ during the second year<br>○ over the whole study	• First occurrence of any RT-PCR-confirmed RSV LRTD assessed by the CEC as:<br>- at least mild lower respiratory tract infection (LRTI)<br>- at least moderate LRTI<br>- at least severe LRTI<br>during the considered year:<br>○ with onset at least 14 days after dosing of study vaccine and prior to study Day 365 (first year comparison)<br>○ with onset after study Day 365 (second year comparison)<br>○ with onset at least 14 days after dosing of study vaccine (whole study comparison)
• To explore the reduction of symptom severity and time to return to usual health in participants with an RT-PCR-confirmed RSV-mediated ARI and in participants with RT-PCR-confirmed RSV-mediated LRTD when compared to placebo<br>○ during the first year<br>○ during the second year<br>○ over the whole study	• In participants with an RT-PCR-confirmed RSV-mediated ARI and in participants with RT-PCR-confirmed RSV-mediated LRTD (during the first year, the second year, or over the whole study, respectively):<br>○ the AUC of the change from baseline in RiiQ Total Symptom score<br>○ the time to return to usual health
• To explore the reduction of disease severity in participants with an RT-PCR-confirmed RSV-mediated ARI and in participants with RT-PCR-confirmed RSV-mediated LRTD when compared to placebo, by subtype (RSV A, RSV B)<br>○ during the first year<br>○ during the second year<br>○ over the whole study	• In participants with an RT-PCR-confirmed RSV-mediated ARI and in participants with RT-PCR-confirmed RSV-mediated LRTD (during the first year, the second year or over the whole study respectively), by subtype (RSV A, RSV B):<br>○ the AUC of the change from baseline in RiiQ Total Symptom score<br>○ the time to return to usual health
• To explore the impact of active study vaccine on the course of an RT-PCR-confirmed RSV-mediated ARI and of human metapneumovirus (hMPV)- and influenza-mediated respiratory infections, general health status and health-related quality of life (HRQoL) measures when compared to placebo	• HRQoL and health status reported by participants on the RiiQ, patient global impression scales, and EuroQoL, 5-Dimension, 5-Level (EQ-5D-5L) in participants with an RT-PCR-confirmed RSV-mediated ARI or hMPV and influenza-mediated respiratory infections

Objectives	Endpoints
• To explore the effect of active study vaccine on each of the following separately: the potential complications (including pneumonia), hospitalizations, emerging therapeutic use and medical resource utilization (MRU) of an RT-PCR-confirmed RSV-mediated ARI, an RT-PCR-confirmed RSV-mediated LRTD, and of hMPV and respiratory infections when compared to placebo<br>○ during the first year<br>○ during the second year<br>○ over the whole study<br>For influenza, the objective is to explore the incidence of these endpoints.	• For each of the following separately: (1) complications, (2) pneumonia, (3) hospitalizations, (4) emerging therapeutic interventions and (5) MRU, all associated with RT-PCR-confirmed RSV-mediated ARI or RT-PCR-confirmed RSV-mediated LRTD during the first year, the second year and over the whole study<br>○ with onset at least 14 days after dosing of study vaccine and prior to study Day 365 (first year comparison)<br>○ with onset after study Day 365 (second year comparison)<br>○ with onset at least 14 days after dosing of study vaccine (whole study comparison)<br>• Complications, pneumonia, hospitalizations, emerging therapeutic use and MRU associated with hMPV and influenza-mediated respiratory infections will be defined similarly
• To explore the efficacy of active study vaccine in the prevention of RT-PCR-confirmed hMPV-mediated ARI and RT-PCR-confirmed hMPV-mediated LRTD when compared to placebo in adults aged 60 years and above	• First occurrence of RT-PCR-confirmed, hMPV-mediated ARI and RT-PCR-confirmed hMPV-mediated LRTD with onset at least 14 days after dosing of study vaccine
• To explore the immune response biomarkers in study participants as correlates of risk of RSV disease and as correlates of protection induced by the active study vaccine	• Assessment of the correlation of immune responses with emphasis on neutralizing and binding antibodies with the risk of RSV disease and protection induced by the vaccine
• To explore biomarkers for the diagnosis of RSV infection, RSV-mediated LRTD and hMPV and influenza- mediated respiratory infections	• Assessment of blood samples collected during ARI episodes for biomarkers that correlate with RSV infection, RSV-mediated LRTD and hMPV and influenza-mediated respiratory infections
• To explore additional vaccine-elicited immune responses in the Immuno Subset	• Assays that may be used include, but are not limited to:<br>○ RSV cross-neutralization of B and/or other A strain(s)<br>○ Antigen specific antibody functional and molecular characterization<br>○ Analysis of neutralizing antibodies to Ad26<br>○ Antigen-specific T-cell immune responses using detailed immunoprofiling
• To increase the information on prior medical history (electronic health records, claims, laboratory data from other care settings) in order to further evaluate its potential effect on the response to immunization and the impact of immunization on efficacy and duration of efficacy as well as AEs that may occur during and after completion of the study	• Utilization of tokenization and matching procedures for exploratory analysis of participant's medical data prior to, during, and following participation in the study (real-world data). Analysis will be performed to relate real-world data to vaccine immune responses, efficacy and duration of protection, and AEs

*Included in the testing strategy

Refer to Section 8, Study Assessments and Procedures for evaluations related to endpoints.

HYPOTHESIS

The study is designed to test the primary hypothesis of VE against RT-PCR-confirmed RSV-mediated LRTD in the PPE population.

- The hypotheses are:

  Null hypothesis: the VE against RT-PCR-confirmed RSV-mediated LRTD of Group 1 vs placebo is ≤20%.

  Alternative hypothesis: the VE against RT-PCR-confirmed RSV-mediated LRTD of Group 1 vs placebo is >20%.

Refer to Section 9.3, Populations for Analyses, for a definition of the PPE population.

4. STUDY DESIGN

4.1. Overall Design

This is a multicenter, randomized, double-blind, placebo-controlled Phase 3 confirmatory efficacy study in participants aged 60 years and older. The primary objective of the study is to further establish the efficacy of the active Ad26.RSV.preF-based study vaccine in the prevention of RT-PCR-confirmed RSV-mediated LRTD.

Up to approximately 27,200 participants (with up to approximately 23,000 in the Global cohort and up to approximately 4,200 participants in specific Asian countries/territories under local protocols) will be enrolled and randomized in parallel in a 2:1 ratio to receive active study vaccine or placebo (Table 2).

Note that some additional local cohort enrollment, beyond the global cohort enrollment may be allowed if required by local health authorities for the purpose of local health authority consideration.

- The Global cohort is defined as all participants (including the Safety subset and the Immuno subset) who are recruited up to the point that global enrollment is stopped.
- Local cohorts are defined as participants from the considered country/territory and under local protocols.

For analyses purposes, data from the Global cohort and the local cohorts may be aggregated. Analyses for local cohort enrollment will be described separately, but they will also be included in the primary analysis and final analysis if their enrollment started prior to the database cut-off of the primary analysis or final analysis, respectively.

Table 2: Study Design: VAC18193RSV3001

Group	N	Day 1
1	15,340	Ad26/protein preF RSV vaccine (1×10^{11} vp/150 µg)
2	7,670	Placebo

Note: The study will be discontinued if the primary analysis is performed by the sponsor and the outcome is negative; however, if at this timepoint, some participants have not completed 6 months of follow up after their vaccination, the study will continue until this follow up period is complete.
N number of participants, vp viral particles

ARI ASSESSMENTS AND PROCEDURES

From study vaccination until 24 months after vaccination (ie, the full duration of the study), participants will be followed up to identify potential cases of RSV infection as further detailed in Section 8.1. After administration of study vaccine, ARI surveillance will be conducted twice weekly via the participant's eDevice (or desktop computer, where applicable) for the duration of the study. The surveillance questions ask participants if they have experienced any ARI symptoms, or for participants who have one or more of these symptoms at baseline, if they have experienced any additional ARI symptoms or a worsening of their baseline symptoms. Participants who experience any symptoms suggestive of an ARI (new onset or worsening, in the setting that baseline symptoms are present) are instructed to indicate this (refer to Appendix 7) and start completing the required ARI episode questionnaires on a daily basis (preferably in the evening) (Appendix 8, Appendix 9, and Appendix 10).[a] Ideally within the next 24 hours, the site and participant should have telephone or telemedicine contact with each other in order for the site to confirm that the participant is experiencing symptoms consistent with an ARI[b] and to give instructions to the participant on the further ARI episode procedures to be followed. Participants will collect a nasal swab at home on the day of symptom onset or the day thereafter (ARI Day 1-2).

On ARI Day 3-5, participants will present to the site, where vital signs will be measured, and a nasal swab, a sputum sample (in participants with a productive cough, when possible), and blood samples (to measure the immune responses at the time of exposure and for exploration of biomarkers that correlate with respiratory infection and disease severity) may be taken by a member of the study staff (see Section 8.1 to determine if the blood samples can be omitted). Ideally within 24 hours after the ARI Day 3-5 visit (but no later than 48 hours after the ARI Day 3-5 visit), a qualified member of the study staff will perform a local RT-PCR of all available nasal swabs with the BioFire® Filmarray Respiratory panel, which will be provided to all sites (both US and non-US); changes to the process might be implemented at the local level based on local requirements. Participants will subsequently be contacted by telephone and given further instructions, as detailed below, on how to further proceed based on the outcome of the RT-PCR

a Note that the PGI-C scale and Return to Usual Health question will not be completed on ARI Day 1.
b This will be assessed by the site by confirming that at least one of the symptoms used to confirm the ARI from the RiiQ Symptom Scale is worse than baseline. As defined in Appendix 5, symptoms used to confirm an ARI episode are: nasal congestion, sore throat, cough, short of breath, coughing up phlegm (sputum), and wheezing (from the RiiQ Symptom Scale). As it is possible that the participants will make errors when they answer the RiiQ Symptom Scale, the telephone or telemedicine contact will be used by the sites to also confirm the correctness of the answers provided by the participant and to confirm the ARI episode was correctly triggered.

tests. Sputum samples (where applicable) will be sent to the central laboratory for testing using the BioFire Filmarray Pneumonia (PN) panel. For all ARI episodes where a sputum sample is collected, the ARI episode will be followed up until ARI resolution (ie, with daily questionnaires and an ARI Day 29 visit) (refer to the procedures in the first bullet point below), independent of the test results from the collected nasal swabs. Participants who are under self-isolation because of a (possible) positive test for SARS-CoV-2 will not come to the site for ARI Day 3-5 assessments but will self-collect the nasal swab.

- Participants with an ARI and a RT-PCR positive for RSV, hMPV, or influenza-mediated ARI will continue filling out the ARI episode questionnaires daily (Appendix 8, Appendix 9, and Appendix 10) until resolution of the ARI episode (defined as 2 consecutive days with no symptoms listed on the RiiQ Symptom Scale, or, for participants who have RiiQ symptoms at baseline [assessed pre-vaccination], 2 consecutive days where all symptoms on the RiiQ Symptom Scale have returned to the same severity level as reported at baseline [or lower]). After resolution of the ARI episode, the participant will return to ARI surveillance with twice weekly reminders.

  On ARI Day 29 (±7 days), regardless of whether the ARI episode has resolved, participants with a RT-PCR positive for RSV, hMPV, or influenza-mediated ARI will be asked to return to the site. During this visit, the MRU questionnaire will be completed (Appendix 11) based on interview with the participant, participants will complete the RiiQ (Appendix 8), the EQ-5D-5L (Appendix 9) and each of the Patient Global Impression (PGI) scales and the Return to Usual Health question (Appendix 10), and the site staff will also collect information on days missed from work (Appendix 12) and on complications, hospitalizations and concomitant medications related to the ARI and record in the electronic case report form (eCRF). The ARI Day 29 visit can also take place by telephone or telemedicine contact.

  For medically-attended RT-PCR positive for RSV, hMPV, or influenza-mediated ARIs, including those resulting in hospitalization, a standard question list will be provided (Appendix 6) to collect additional information on any other diagnostic tests (eg, chest x-rays, spirometry, pulmonary function tests, etc.) or on any interventions during the clinical course of the ARI.

  Note: the same procedures apply for participants with a sputum sample taken and for participants with at least one nasal swab that cannot be tested at the site with the BioFire Filmarray Respiratory panel.
  Participants will remain blinded as to the outcome of RSV, hMPV and influenza RT-PCR test results until study unblinding and the test results should only be disclosed in the event of safety concerns. RT-PCR test results related to the detection of other respiratory pathogens can be communicated to the participants. The RT-PCR results that are obtained with the BioFire Filmarray testing under the study procedures will be used for research purposes only and will not be used by the investigator for therapeutic decisions. A locally approved diagnostic test external to the study procedures, including the RSV RT-PCR, should be obtained in case the participant's own healthcare provider considers it warranted (eg, based on symptoms and/or risk of severe disease) and/or according to local/site specific guidelines. If a sample tests positive for SARS-CoV-2, the participant will be referred to their own healthcare provider for locally approved diagnostic test and further management, in accordance with local/site specific guidelines.

- Participants with negative RT-PCR results for RSV, hMPV, or influenza will be informed by telephone or telemedicine contact that they can stop filling out the daily ARI episode questionnaires and the site will end the ARI episode and return the participant to ARI surveillance.
- For participants with a positive SARS-CoV-2 test and a negative test for RSV, hMPV, and influenza, the collection of ARI data by the study site will be stopped for the ARI episode. The participant will be informed by telephone or telemedicine contact that they can stop filling out the daily ARI episode questionnaires, and the site will end the ARI episode and return the participant to ARI surveillance with twice weekly reminders. Participants who have a positive SARS-CoV-2 RT-PCR test as part of the study procedures should be referred to their own healthcare provider for locally approved diagnostic test and further management, in accordance with local/site specific guidelines. Participants whose standard-of-care testing during an ARI episode is positive for SARS-CoV-2, or who have been in contact with someone that tested positive for SARS-CoV-2 and therefore must self-isolate according to the local guidelines, will not come to the site for ARI Day 3-5 assessments but will self-collect the nasal swab. In this circumstance, other ARI Day 3-5 assessments (vital signs, sputum sample and blood samples) might not be performed.

  Participants with positive RT-PCR results for RSV (or hMPV or influenza) should keep on filling out the daily ARI episode questionnaires until resolution of the ARI episode independently of any other co-infection (including SARS-CoV-2).

When the end of RSV season one telephone call or telemedicine contact occurs within 6 months after vaccination, the participants will be contacted again by telephone or telemedicine at 6 months post-vaccination to collect information on SAEs and AESIs (applies to Visit 3 [end of season one/6-month follow-up]).

At Month 24, all participants will be contacted by telephone or telemedicine (or come to the site if they have eDevices to return) to ensure the participants complete the RiiQ, the PGI-H and the EQ-5D-5L.

Additional study procedures and assessments for efficacy, immunogenicity, safety, and MRU will be performed as described in Section 8.

Throughout the study, RT-PCR results from the nasal swabs and sputum sample (where available) will be used to determine whether the infection was caused by RSV. If at least one of these samples is positive for RSV, the collected information will be applied against the clinical case definition.

The study will be discontinued if the primary analysis is performed by the sponsor and the outcome is negative; however, if at that timepoint, some participants have not completed 6 months of follow-up after their vaccination, the study will continue until this follow-up period is complete.

The study duration for an individual participant is approximately 24 months.

An IDMC will be commissioned for this study. Refer to Committees Structure in Appendix 2: Regulatory, Ethical, and Study Oversight Considerations for details.

A Clinical Event Adjudication Committee (CEC) will be constituted. The CEC will review all available data of all RSV-mediated ARIs and will confirm if the RT-PCR-confirmed RSV LRTD definition criteria were met. Further for all RT-PCR-confirmed RSV LRTD cases the CEC will assess their severity (mild, moderate or severe). Further details are provided in Section 9.6.2.

A diagram of the study design is provided in Section 1.2, Schema.

4.2. Scientific Rationale for Study Design

Blinding, Control, Study Phase/Periods, Vaccine Groups

A placebo control will be used to establish the frequency and magnitude of changes in clinical endpoints that may occur in the absence of active vaccination. Randomization will be used to minimize bias in the assignment of participants to vaccine groups (active vaccine or placebo), to increase the likelihood that known and unknown participant attributes (eg, demographic and baseline characteristics) are evenly balanced across vaccine groups, and to enhance the validity of statistical comparisons across the vaccine groups. Blinded vaccination will be used to reduce potential bias during data collection and evaluation of clinical endpoints.

Medical Resource Utilization Data Collection

Prophylaxis of RSV infection may reduce the need for and duration of supportive care (such as hospitalization, oxygen supplementation, etc.). The study will evaluate the impact of active study vaccine versus placebo on the development and clinical course of RSV disease.

Participant Medical Information Prior to, During and After the Study (Real-world Data)

Real-world data plays a critical role in improving understanding of factors that may influence response to immunization and the effectiveness and safety of a vaccine product during and after completion of the study. This may be important in gaining insight in terms of duration of efficacy and incidence of AEs after study completion. This may be especially important in the event that efficacy of Ad26/protein preF RSV vaccine or another vaccine is shown and follow-up in a randomized manner is compromised.

To allow the linking of participant records from different sources, ie, data collected as part of the study as specified in the Schedule of Activities and longitudinal real-world data (from 5 years prior to enrollment in the study until 5 years after study completion) such as electronic health records, claims, and laboratory data from other care settings, without compromising the participant's confidentiality, tokenization and matching procedures will be utilized (for participants from the US only). The tokenization process starts with each data provider generating a token behind the firewall via a proprietary software. Personal information such as names and dates of birth from study participants are removed from real-world data sources and replaced with encrypted, one-way, hashed identifiers, and then further encrypted using asymmetric keys in compliance with Health Insurance Portability and Accountability Act (HIPAA 1996). This encrypted anonymized information is sent for matching to the anonymized participant master index. While it is not possible to reverse the hash, source-specific tokens can be decrypted and re-encrypted so that

records can be linked across sources. The result of the process is a unique anonymized identifier for each participant, which can be used to link participant records across sources.

4.2.1. Study-Specific Ethical Design Considerations

Potential participants will be fully informed of the risks and requirements of the study and, during the study, participants will be given any new information that may affect their decision to continue participation. They will be told that their consent to participate in the study is voluntary and may be withdrawn at any time with no reason given and without penalty or loss of benefits to which they would otherwise be entitled. Only participants who are fully able to understand the risks, benefits, and potential AEs of the study, and provide their consent voluntarily will be enrolled.

The primary ethical concern is that this study will be performed in adult participants who will receive no direct benefit from participation in the study, except for participant reimbursement for the time and inconveniences that may arise from participation in the study. See Section 2.3 for details on potential and known benefits and risks, and for the safety measures taken to minimize risk to participants.

The total blood volume to be collected is considered to be an acceptable amount of blood to be collected over this time period from the population in this study based upon the standard of the US Department of Health and Human Services Office for Human Research Protections, and US FDA guidelines of 550 mL in any 8-week period (OHRP 1998, FDA 1998).

For participants (from the US only) who consent to the optional collection of real-world medical data, the sponsor is committed to protect their data and privacy. Tokenization and matching procedures will be utilized to allow for those participant's medical data to be obtained without violation of participant confidentiality. Participants will be informed that consent to this part of the study is completely optional and that they can withdraw their consent at any given time. In the event of withdrawal of consent, the sponsor will remove the token generated and any associated linked real-world data. Participation in or withdrawal from this optional part of the study will not affect the participation in the main study.

4.3. Justification for Dose

The dose levels for Ad26.RSV.preF and RSV preF protein used in this study were determined from the primary analysis of Cohort 2 in Study VAC18193RSV1004. From the primary analysis, a significant increase in virus neutralizing antibodies (VNA) A2 titers was observed in the groups combining Ad26.RSV.preF and RSV preF protein compared to Ad26.RSV.preF 1×10^{11} vp alone. No relevant differences between the mixture groups and Ad26.RSV.preF alone were observed for other immunogenicity assays available at the time of the primary analysis. All regimens had acceptable safety and reactogenicity profiles, which were similar across groups. Based on these data, and on the outcome of the primary analysis of the proof-of-concept efficacy study VAC18193RSV2001 (see Section 2.2), the mixture of Ad26.RSV.preF 1×10^{11} vp and 150 µg of the RSV preF protein was selected for the present study.

4.4. End of Study Definition

End of Study Definition

The end of study is considered as the last visit for the last participant in the Global cohort of the study.

The final data from the study site will be sent to the sponsor (or the designee) after completion of the final participant visit at that study site, in the time frame specified in the Clinical Trial Agreement.

Study Completion Definition

A participant will be considered to have completed study vaccination if the participant has received a vaccination.

A participant will be considered to have completed the study if the participant has completed all assessments at the final visit.

5. STUDY POPULATION

Participants will be adult men and women ≥60 years of age on the day of signing the Informed Consent Form (ICF). Participants must be in stable health (on the basis of medical history and vital signs measurement performed at Day 1).

Participants in this age range with congestive heart failure (CHF), coronary artery disease (such as angina pectoris, ischemic cardiomyopathy, history of myocardial infarct, or history of coronary artery bypass graft or coronary artery stent), and chronic lung disease (such as asthma and chronic obstructive pulmonary disease [COPD]) are generally at higher risk for severe RSV disease (CDC 2020); hereafter, this population will be referred to as "increased risk". Although no required minimum is set in the overall population, it is expected that ~25% of the participants enrolled in the study will have medical conditions that place them in this "increased risk" group.

The randomization will be set-up to ensure that ~50% of participants in the Safety Subset, and ~50% of participants in the Immuno Subset will be at increased risk of severe RSV disease and ~50% of them will be 75 years or older (refer to Section 6.3, Measures to Minimize Bias: Randomization and Blinding).

A cap will be installed on the number of participants in the 60-64 year age group (see Section 6.3 for information related to caps).

Screening and vaccination may be split into 2 visits after consultation with the sponsor or its delegate. Every effort should be made for split visits to occur preferably within 3 to 5 days and no later than 14 days, and pre-vaccination vital signs should be repeated on the day of vaccination if this visit is split.

The inclusion and exclusion criteria for enrolling participants in this study are described below. If there is a question about these criteria, the investigator must consult with the appropriate sponsor

representative and resolve any issues before enrolling a participant in the study. Waivers are not allowed.

For a discussion of the statistical considerations of participant selection, see Section 9.2, Sample Size Determination.

5.1. Inclusion Criteria

Each potential participant must satisfy all of the following criteria to be enrolled in the study:

1. Must sign an ICF indicating that the participant understands the purpose, procedures and potential risks and benefits of the study, and is willing to participate in the study.
2. Willing and able to adhere to the prohibitions and restrictions specified in this protocol.
3. Must be ≥60 years old on the day of signing the ICF and expected to be available for the duration of the study.
4. Before randomization, a participant must be:
    - postmenopausal (postmenopausal state is defined as no menses for 12 months without an alternative medical cause); and
    - not intending to conceive by any methods.

    Note: Hysterectomized participants are also eligible for the study.
5. Criterion modified per Amendment 3

    5.1 Criterion modified per Amendment 4

    5.2 In the investigator's clinical judgment, the participant must be in stable health at the time of vaccination. Participants may have underlying illnesses such as hypertension, CHF, COPD, type 2 diabetes, hyperlipoproteinemia, or hypothyroidism, as long as their symptoms and signs are stable and medically controlled in the judgement of the investigator at the time of vaccination, and these conditions receive routine follow-up by the participant's healthcare provider. Participants will be included on the basis of medical history and vital signs[a] taken between ICF signature and vaccination.
6. From the time of vaccination through 3 months after vaccination, participant agrees not to donate blood.
7. Must be able to read, understand, and complete questionnaires in the eDiary.
8. Must be willing to provide verifiable identification, has means to be contacted and to contact the investigator during the study.
9. Must be able to work with smartphones/tablets/computers.

---

[a] Participants can be enrolled with ≤Grade 3 values for vital signs measurements (other than temperature), if these are deemed not clinically significant by the investigator (refer to Appendix 4: Toxicity Grading Scale). Confirmation of investigator's clinical assessment must be provided in the participant's medical record.

5.2. Exclusion Criteria

Any potential participant who meets any of the following criteria will be excluded from participating in the study:

1. Has a serious clinically unstable condition, (eg, end-stage renal disease with or without dialysis, clinically unstable cardiac disease), Alzheimer's disease, or any other condition for which, in the opinion of the investigator, participation would not be in the best interest of the participant (eg, compromise well-being) or that could prevent, confound, or limit the protocol-specified assessments.
2. History of malignancy within 5 years before screening not in the following categories:
   a. Participants with squamous and basal cell carcinomas of the skin and carcinoma in situ of the cervix may be enrolled at the discretion of the investigator.
   b. Participants with a history of malignancy within 5 years, which is considered cured with minimal risk of recurrence per investigator's judgement, can be enrolled.
3. Had major surgery (eg, major cardiopulmonary or abdominal operations) as per the investigator's judgment within 4 weeks before vaccination, or will not have fully recovered from surgery, or has major surgery planned during the time the participant is expected to participate in the study.
4. Criterion modified per Amendment 1
   4.1 Criterion modified per Amendment 3
   4.2 Has abnormal function of the immune system resulting from:
      a. Clinical conditions (eg, autoimmune disease or immunodeficiency) expected to have an impact on the immune response elicited by the study vaccine.

         Participants with autoimmune disease (eg, autoimmune-mediated thyroid disease, autoimmune inflammatory rheumatic disease such as rheumatoid arthritis, and type 1 diabetes) that is stable and inactive without the use of systemic immunomodulators and glucocorticoids may be enrolled at the discretion of the investigator.
      b. Chronic or recurrent use of systemic corticosteroids within 2 months before administration of study vaccine and during the study. A substantially immunosuppressive steroid dose is considered to be ≥2 weeks of daily receipt of 20 mg of prednisone or equivalent.

         Note: Ocular, topical, intra-articular, or inhaled steroids are allowed.
      c. Administration of antineoplastic and immunomodulating agents, eg, cancer chemotherapeutic agents, or radiotherapy within 6 months before administration of study vaccine and during the study.

         Note: Ocular and topical antineoplastic and immunomodulating agents are allowed.
5. Criterion modified per Amendment 1
   5.1 Received or plans to receive:
      a. Licensed live-attenuated vaccines within 28 days before or after planned administration of study vaccination

b. Other licensed (not live) vaccines within 14 days before or after planned administration of study vaccination.

6. Has an acute illness (including acute respiratory illnesses) or body temperature of ≥38.0ºC (≥100.4ºF) within 24 hours prior to administration of study vaccine.

   Note: Enrollment at a later date is permitted.

7. Has had major psychiatric illness and/or drug or alcohol abuse which in the investigator's opinion would compromise the participant's safety and/or compliance with the study procedures.

8. Known or suspected allergy or history of anaphylaxis or other serious adverse reactions to vaccines or vaccine components (including any of the constituents of the study vaccine). Refer to the latest version of the Investigator's Brochure for Ad26/protein preF RSV vaccine.

9. History of acute polyneuropathy (eg, Guillain-Barré syndrome) or chronic idiopathic demyelinating polyneuropathy (CIDP).

10. Criterion modified per Amendment 1

    10.1 Received hematopoietic stem cell transplant in medical history, immunoglobulins in the 2 months, immunoglobulins specific to RSV, hMPV, or parainfluenza viruses in the 12 months, apheresis therapies in the 4 months, or blood products in the 4 months before the planned administration of the study vaccine or has any plans to receive such treatment during the study.

    Note: Use of monoclonal antibodies (mAbs) before administration of study vaccine and during the study conduct is permitted with the exception of those targeting T cells (anti-CD3, CD4, CD33, and CD52), B cells (anti-CD45, CD19, CD20, CD22, CD27, CD38, and CD138) and check point inhibitors (PD-1, PDL-1 and CTLA-4). Examples of not allowed MABS are: muromonab-CD3, gemtuzumab ozogamicin, alemtuzumab, bevacizumab, rituximab, ofatumumab, ocrelizumab, tositumomab, veltuzumab, obinutuzumab, epratuzumab, pembrolizumab, nivolumab, atezolizumab, avelumab, durvalumab and CTLA-4 targeting ipilimumab. Participants receiving these mAbs in the 2 months before the planned administration of the study vaccine or who plan to receive these mAbs during the study will be excluded from participating in the study.

11. Criterion modified per Amendment 3

    11.1 Received an investigational drug or used an invasive investigational medical device within 30 days or received an investigational vaccine within 6 months before the planned administration of the study vaccine or is currently enrolled or plans to participate in another investigational study during this study.

    Note: Participation in an observational clinical study (ie, without intervention) or in the observational phase of interventional studies is allowed upon approval of the sponsor or its delegate.

12. Contraindication to IM injections and blood draws (eg, bleeding disorders).

13. Criterion modified per Amendment 1

    13.1 Received an RSV vaccine in a previous RSV vaccine study.

14. Criterion deleted per Amendment 1

15. Employee of the investigator or study site, with direct involvement in the proposed study or other studies under the direction of that investigator or study site, as well as family members of the employees or the investigator, or an employee of the sponsor.
16. Criterion modified per Amendment 4

    16.1 Who, in the opinion of the investigator, is unlikely to adhere to the requirements of the study, or is unlikely to complete the vaccination and observation.
17. Cannot communicate reliably with the investigator.
18. **For participants in the Safety Subset only**: Participants who have significant scarring, tattoos, abrasions, cuts, or infections over the deltoid region of both arms that, in the investigator's opinion, could interfere with evaluation of injection site local reactions.
19. Criterion added per Amendment 1

    19.1 Criterion modified per Amendment 4

    19.2 Received or plans to receive a SARS-CoV-2 vaccine:

    a. Live-attenuated SARS-CoV-2 vaccine within 28 days before or after planned administration of the study vaccine.

    b. Non-live SARS-CoV-2 vaccine within 14 days before or after planned administration of the study vaccine.

    c. A viral-vectored SARS-CoV-2 vaccine within 28 days before or after planned administration of the study vaccine.
20. Criterion added per Amendment 1

    20.1 Criterion modified per Amendment 4

    20.2 Received or plans to receive an Ad26-vectored vaccine at any time prior to randomization until 28 days after study vaccination (this does not apply to SARS-CoV-2 vaccines; please refer to exclusion criterion 19).
21. Criterion added per Amendment 2

    21.1 History of TTS or heparin-induced thrombocytopenia and thrombosis (HITT).

NOTE: Investigators must ensure that all study enrollment criteria have been met prior to the first dose. If a participant's clinical status changes (including any available laboratory results or receipt of additional medical records) after screening but before vaccination such that the participant no longer meets all eligibility criteria, then the participant must be excluded from participation. The required documentation to support meeting the enrollment criteria is described under Source Documents in Appendix 2: Regulatory, Ethical, and Study Oversight Considerations.

5.3. Lifestyle Considerations

Potential participants must be willing and able to adhere to the following lifestyle restrictions during the study to be eligible for participation:

1. See Section 6.8, Concomitant Therapy for details on prohibited and restricted therapy during the study.

2. Agree to follow all requirements that must be met during the study as noted in the Inclusion and Exclusion Criteria.

5.4. Screen Failures

Participant Identification, Enrollment, and Screening Logs

The investigator agrees to complete a participant identification and enrollment log to permit easy identification of each participant during and after the study. This document will be reviewed by the sponsor study site contact for completeness.

The participant identification and enrollment log will be treated as confidential and will be filed by the investigator in the study file. To ensure participant confidentiality, no copy will be made. All reports and communications relating to the study will identify participants by participant identification and age at initial informed consent. In cases where the participant is not randomized into the study, the date seen and age at initial informed consent will be used.

The investigator must also complete a participant screening log, which reports on all participants who were seen to determine eligibility for inclusion in the study.

Individuals who do not meet the criteria for participation in this study (screen failure) but at some point in the future are expected to meet the eligibility criteria may be rescreened. Individuals who are rescreened will be assigned a new participant number and will undergo the informed consent process, and then restart a new screening phase.

5.5. Criteria for Temporarily Delaying Administration of Study Vaccine

The following events constitute a temporary contraindication to study vaccination:

- Clinically significant acute illness at the time of vaccination.
- Fever (body temperature ≥38.0°C) within 24 hours prior to the planned time of vaccination.

If any of these events occur at the scheduled time for the vaccination, randomization at a later date is permitted at the discretion of the investigator and after consultation with the sponsor (refer to the Schedule of Activities).

If the vaccination visit cannot be rescheduled within the allowed window or the contraindications to vaccination persist, the sponsor should be contacted for further guidance.

6. STUDY VACCINE AND CONCOMITANT THERAPY

6.1. Study Vaccine Administration

The investigational medicinal products to be administered to participants in this study are Ad26.RSV.preF, RSV preF protein, and placebo. The Ad26/protein preF RSV vaccine to be used in this study is composed of Ad26.RSV.preF and RSV preF protein, to be administered as a single injection (1.0 mL) in the deltoid muscle:

- Ad26.RSV.preF (JNJ-64400141) will be supplied at a concentration of 2×10^{11} vp/1 mL in single-use vials. A dose level of 1×10^{11} vp will be used.
- RSV preF protein (JNJ-64213175) will be supplied at a concentration of 0.3 mg/1 mL in single-use vials. A dose level of 150 µg will be used.
- Placebo for Ad26.RSV.preF and for RSV preF protein.

The unblinded pharmacist, or other qualified individual, who will have no other study function will prepare the appropriate syringe, labeled with the participant's study identification number, and provide the syringes for Ad26/protein preF RSV vaccine and placebo in a blinded manner to the blinded study vaccine administrator who will perform the injection.

Study vaccine administration must be captured in the source documents and the eCRF.

The full details of study vaccine preparation will be provided in the Site Investigational Product Procedures Manual and in the Investigational Product Preparation Instruction.

6.2. Preparation/Handling/Storage/Accountability

Ad26.RSV.preF/RSV preF protein will be manufactured and provided under the responsibility of the sponsor. Refer to the Investigator's Brochure for Ad26/protein preF RSV vaccine for a list of excipients.

Preparation/Handling/Storage

All study vaccine must be stored in a secured location with no access for unauthorized personnel and at controlled temperatures as indicated on the clinical labels. If study vaccine is exposed to temperatures outside the specified temperature range, all relevant data will be sent to the sponsor to determine if the affected supplies can be used or will be replaced. The affected study vaccine must be quarantined and not used until further instruction from the sponsor is received.

Refer to the Site Investigational Product Procedures Manual and to the Investigational Product Preparation Instruction for additional guidance on study vaccine preparation, handling, and storage.

Accountability

The investigator is responsible for ensuring that all study vaccine received at the site is inventoried and accounted for throughout the study. The study vaccine administered to the participant must be documented on the vaccine accountability form. All study vaccine will be stored and disposed of according to the sponsor's instructions. Study site personnel must not combine contents of the study vaccine containers.

Study vaccine must be handled in strict accordance with the protocol and the container label, and must be stored at the study site in a limited-access area or in a locked cabinet under appropriate environmental conditions. Unused study vaccine must be available for verification by the sponsor's study site monitor during on-site monitoring visits. The return to the sponsor of unused study vaccine will be documented on the Investigational Product Destruction Form. When the

study site is an authorized destruction unit and study vaccine supplies are destroyed on-site, this must also be documented on the Investigational Product Destruction Form.

Potentially hazardous materials containing hazardous liquids, such as used ampules, needles, syringes and vials, should be disposed of immediately in a safe manner and therefore will not be retained for vaccine accountability purposes.

Study vaccine must be dispensed under the supervision of the investigator or a qualified member of the study site personnel. Study vaccine will be supplied only to participants participating in the study. Study vaccine may not be relabeled or reassigned for use by other participants. The investigator agrees neither to dispense the study vaccine from, nor store it at, any site other than the study sites agreed upon with the sponsor.

Refer to the Investigational Product Preparation Instructions and Study Site Investigational Product and Procedures Manual for additional guidance on the final disposition of unused study vaccines.

6.3. Measures to Minimize Bias: Randomization and Blinding

Study Vaccine Allocation

Central randomization will be implemented in this study. On Day 1, participants will be randomly assigned in a 2:1 ratio to 1 of 2 groups (active vaccine or placebo) based on a computer-generated randomization algorithm prepared before the study by or under the supervision of the sponsor. Participants who have provided additional informed consent will be enrolled in the Safety Subset and/or in the Immuno Subset.

The interactive web response system (IWRS) will assign a unique code, which will dictate the group assignment and matching study vaccine kit for the participant. The requestors must use their own user identification and personal identification number when contacting the IWRS and will then give the relevant participant details to uniquely identify the participant.

To ensure balance across arms, the randomization will be stratified by age category (ie, 60-64 years, 65-74 years, 75-84 years, ≥85 years), and by being at increased risk of severe RSV disease (yes/no). Within strata, minimization will be applied for subset (being part of the Safety Subset only, the Immuno Subset only, both subsets or none), region (North America, Europe, SH), site, and stratum combination. The following caps will be installed for the global cohort:

- The total number of 60-64 year old participants will be a maximum of 2,000 participants. Depending on recruitment, caps might be installed with the aim to have ~10% 60-64 year old participants per region.
- The Safety Subset (including participants who are only in the Safety Subset and who are in both the Safety and Immuno Subset) will include at least 1,050 participants, ~50% of them will be at increased risk for severe RSV disease, ~50% of them will be 75 years or older.

  The Safety Subset should contain ~750 participants from North America and ~300 from each of the other regions in the setting that these are part of the study. In each region,

~50% of the Safety Subset will be at increased risk of severe RSV disease and ~50% will be 75 years or older.

- The Immuno Subset (including participants who are only in the Immuno Subset and who are in both the Safety and Immuno Subset) will include at least 360 participants, ~50% of them will be at increased risk for severe RSV disease and ~50% will be 75 years or older.

  The Immuno Subset should contain at least 180 participants from North America, at least 180 from Europe, and ~120 from each of the other regions in the setting that these are part of the study. In each region, ~50% of the Immuno Subset will be at increased risk of severe RSV disease and ~50% will be 75 years or older.

No other caps are installed for the Global cohort.

In each of the regions, the non-increased risk Safety Subset must be fully enrolled before further enrollment of non-increased risk participants. Also, the 60-74 years Safety Subset must be fully enrolled before further enrollment of participants aged 60-74 years. Similarly, in each region, the increased risk Safety Subset must be fully enrolled before further enrollment of increased risk participants. Also, the 75-years-and-older Safety Subset must be fully enrolled before further enrollment of participants aged 75 years and older. Immuno Subset participants will be enrolled only at a selection of sites.

Additional local cohort enrollment, beyond the Global cohort enrollment, may be allowed if required by local health authorities for the purpose of local regulatory approval consideration. This will also be handled via the IWRS and will follow the same randomization ratio as the Global cohort. Stratification and minimization will be done in a similar way as for the Global cohort. The number of 60-64 year old participants in those local cohorts will be a maximum 10% of the total cohort. Caps for the Immuno Subset and the Safety Subset will depend on local requirements.

Blinding

The investigator will not be provided with randomization codes. The codes will be maintained within the IWRS, which has the functionality to allow the investigator to break the blind for an individual participant.

In general, for the Global cohort, randomization codes will be disclosed fully only if the study is completed and the clinical database is closed. The participants, study site personnel (except for those with primary responsibility for study vaccine preparation and dispensing), and investigator will be blinded to the study vaccine allocation until the end of the study. The sponsor will be blinded to Day 1 study vaccine allocation until the database lock for the primary efficacy analysis except for the sponsor representatives involved in independent drug monitoring.

From the primary efficacy analysis onwards, group level results may be shared externally as needed, however, efforts will be made to preserve the blinding to the individual participant allocation until the end of the study.

For local cohorts, similar blinding principles hold. The randomization codes will be disclosed fully if the study of the local cohort is completed and the respective clinical database is closed, but not

before the global clinical database is closed. Local study teams will be blinded to study vaccination until the primary analysis as specified per local requirements, but this cannot be conducted prior to the primary analysis of the Global cohort.

For countries/territories that contribute to both the Global and the local cohorts, the randomization codes will be disclosed fully if the study of the local cohort is completed and the respective clinical database is closed, but not before the global clinical database is closed. The global study team of the sponsor will be unblinded. The local study teams will be blinded to study vaccination until the primary analysis as specified per local requirements, but this cannot be conducted prior to the primary analysis of the Global cohort.

The investigator may in an emergency determine the identity of the vaccination by contacting the IWRS. While the responsibility to break the vaccination code in emergency situations resides solely with the investigator, it is recommended that the investigator contact the sponsor or its designee if possible, to discuss the particular situation before breaking the blind. Telephone contact with the sponsor or its designee will be available 24 hours per day, 7 days per week. In the event the blind is broken, the sponsor must be informed as soon as possible. The date, time, and reason for the unblinding must be documented by the IWRS, in the appropriate section of the eCRF, and in the source document. The documentation received from the IWRS indicating the code break must be retained with the participant's source documents in a secure manner. The investigator is advised not to reveal the study vaccine assignment to the study site personnel or sponsor personnel/sponsor representative.

If the randomization code is broken by the sponsor for safety reporting purposes, the participant may remain in the study (if the randomization code is still blinded to the study site personnel and the participant).

Participants who withdraw will not be replaced.

6.4. Study Vaccination Compliance

Study vaccine (active or placebo) will be administered IM by a blinded vaccine administrator a trained and qualified study nurse, medical doctor, or otherwise qualified healthcare professional.

Details of study vaccine administration will be recorded in the eCRF (including date and time of injection and deltoid).

For details on blinding procedures, see Section 6.3, Measures to Minimize Bias: Randomization and Blinding.

6.5. Dose Modification

Not applicable.

6.6. Continued Access to Study Vaccine After the End of the Study

Participants will be instructed that study vaccine will not be made available to them after they have completed study vaccination.

6.7. Overdose

For this study, any dose of vaccine greater than the assigned dose will be considered an overdose. The sponsor does not recommend specific treatment for an overdose. In the event of a known overdose, the investigator should:

- Contact the medical monitor immediately.
- Closely monitor the participant for AE/SAE/AESI/medically-attended AE (MAAE) (ie, the participant will remain at the study site for at least 1 hour and will be closely monitored for allergic or other reactions by study staff. Follow-up telephone calls or telemedicine contacts 12 hours and 24 hours post-dose will be made).
- Document the quantity of the excess dose in the source document.
- Report as a special reporting situation.

6.8. Concomitant Therapy

For All Participants

Prestudy specific therapies such as analgesics/antipyretics and non-steroidal anti-inflammatory drugs (NSAIDs) administered up to 30 days before the dose of study vaccine must be recorded at screening. Use of analgesics/antipyretics and NSAIDs for routine prophylaxis prior to study vaccine administration is discouraged, however, should be used only in case of medical need and in line with local standard of care.

Concomitant therapies associated with SAEs, AESIs, and with AEs leading to discontinuation from study will be collected and recorded in the eCRF from the time of vaccination through the end of the study. Concomitant medications associated with RSV, hMPV, and influenza-mediated ARI episodes and with complications of RSV, hMPV, and influenza-mediated ARIs will be captured in the eCRF for the duration of the study.

The use of systemic corticosteroids must be documented throughout the study. Antineoplastic and immunomodulating agents, eg, cancer chemotherapeutic agents or chronic or recurrent use of systemic corticosteroids, or radiotherapy are prohibited throughout the study. If the use of systemic corticosteroids, antineoplastic or immunomodulating agents becomes medically indicated during the study for any participant, the sponsor should be contacted.

Vaccination with licensed live-attenuated vaccines within 28 days of the study vaccination (ie, before or after) is prohibited. Other licensed (not live) vaccines (eg, tetanus, hepatitis A or B, rabies) should be given at least 14 days before (or at least 14 days after) administration of study vaccine. If a vaccine is indicated in a post-exposure setting (eg, rabies or tetanus), it must take priority over the study vaccine.

For SARS-CoV-2 vaccines either licensed or available under Emergency Use Authorization: live-attenuated vaccines should be given at least 28 days before or after study vaccination; non-live vaccines should be given at least 14 days before or after study vaccination. A viral-vectored SARS-CoV-2 vaccine is not to be given within 28 days before or after study vaccination. Any history of

SARS-COV-2 vaccination (name/manufacturer of the vaccine and date of administration, whenever possible) prior to and during the study will be collected in the eCRF.

Seasonal influenza vaccination is recommended, but must occur at least 14 days before or at least 14 days after the study vaccine administration. Any history of influenza vaccination (name/manufacturer of the vaccine and date of administration, whenever possible) within 3 months prior to enrolment in the study to and during the study will be collected in the eCRF.

The sponsor must be notified in advance (or as soon as possible thereafter) of any instances in which prohibited therapies are administered.

For Participants in the Safety Subset

For participants in the Safety Subset, concomitant therapies associated with unsolicited AEs will be collected and recorded in the eCRF from the time of the study vaccine administration through 28 days after vaccination. Concomitant therapies associated with solicited AEs will be collected by the participants and recorded in the eCRF from the time of vaccine administration through 7 days after vaccination.

Information on concomitant use of herbal supplements or vitamins will not be collected.

7. DISCONTINUATION OF STUDY VACCINATION AND PARTICIPANT DISCONTINUATION/WITHDRAWAL

7.1. Discontinuation of Study Vaccination

Not applicable.

7.2. Participant Discontinuation/Withdrawal From the Study

A participant will be withdrawn from the study for any of the following reasons:

- Lost to follow-up
- Withdrawal of consent
- Death
- Repeated failure to comply with protocol requirements
- Any AE that requires discontinuation from the study
- Decision by the sponsor or the investigator to stop or cancel the study
- Decision by local regulatory authorities and Institutional Review Board (IRB)/ Independent Ethics Committee (IEC) to stop or cancel the study
- If the randomization code is broken by the investigator or the study site personnel.
  Note: If the randomization code is broken by the sponsor for safety reporting purposes, the participant may remain in the study (if the randomization code is still blinded to the study site personnel and the participant).

When a participant withdraws before study completion, the reason for withdrawal is to be documented in the eCRF and in the source document. All efforts should be made for the participant to return the eDevice provided by the site, as applicable. If the reason for withdrawal from the study is withdrawal of consent then no additional assessments are allowed.

Withdrawal of Consent

A participant declining to return for scheduled visits does not necessarily constitute withdrawal of consent. Alternate follow-up mechanisms that the participant agreed to when signing the consent form apply as local regulations permit.

7.2.1. Withdrawal From the Use of Research Samples

Withdrawal From the Use of Samples in Future Research

The participant may withdraw consent for use of samples for research (refer to Long-Term Retention of Samples for Additional Future Research in Appendix 2: Regulatory, Ethical, and Study Oversight Considerations). In such a case, samples will be destroyed after they are no longer needed for the clinical study. Details of the sample retention for research are presented in the main ICF.

7.3. Lost to Follow-up

To reduce the chances of a participant being deemed lost to follow-up, prior to randomization, attempts should be made to obtain contact information from each participant, eg, home, work, and mobile telephone numbers and email addresses for both the participant as well as appropriate family members.

A participant will be considered lost to follow-up if the participant repeatedly fails to return for scheduled visits and is unable to be contacted by the study site. A participant cannot be deemed lost to follow-up until all reasonable efforts made by the study site personnel to contact the participant are deemed futile. The following actions must be taken if a participant fails to return to the study site for a required study visit:

- The study site personnel must attempt to contact the participant to reschedule the missed visit as soon as possible, to counsel the participant on the importance of maintaining the assigned visit schedule, to ascertain whether the participant wishes to or should continue in the study.
- Before a participant is deemed lost to follow up, the investigator or designee must make every reasonable effort to regain contact with the participant (where possible, 3 telephone calls, telemedicine contacts, e-mails, fax, and, if necessary, a certified letter to the participant's last known mailing address, or local equivalent methods. These contact attempts should be documented in the participant's medical records.
- Should participants continue to be unreachable, they will be considered to have withdrawn from the study.

Should a study site close, eg, for operational, financial, or other reasons, and the investigator cannot reach the participant to inform them, their contact information will be transferred to another study site.

8. STUDY ASSESSMENTS AND PROCEDURES

Overview

The Schedule of Activities summarizes the frequency and timing of all measurements applicable to this study. Unscheduled visits may be performed based on the investigator's clinical judgment and may include further evaluations, as needed.

If multiple assessments are scheduled for the same timepoint, it is recommended that procedures be performed in the following sequence: electronic patient-reported outcomes (ePROs), nasal swabbing and/or nasosorption sampling, blood draws. If needed, assessments may be performed on another day within the applicable visit window. Actual dates and times of assessments will be recorded in the source documents and/or eCRF and/or eDevice.

eDevices will be provided for participants who require them. Instructions for completing eDiaries on the provided eDevice or the participant's own eDevice[a] (or desktop computer, where applicable) will be provided to all participants on the day of study vaccination, together with a nasal swab sample kit. Participants in the Safety Subset will receive additional instructions for completing safety eDiaries on the eDevice (to record body temperature and solicited local [at the injection site] and systemic signs and symptoms), and these participants will also be provided with a thermometer (to measure body temperature) and a ruler (to measure local injection site reactions).

The eDiary instructions on how to capture the data and grading scales to assess severity of the signs and symptoms will be provided to participants. The study staff is responsible for providing appropriate (re)training to the participant to avoid missing or incorrect data. The eDiary will be reviewed by study personnel at the visits indicated in the Schedule of Activities. If the eDiary review is missed, the eDiary will be reviewed during the next visit.

If a participant is unable to complete the eDiary, as a fall back option, a study staff member can collect information by contacting the participant by telephone or telemedicine (or visit the participant at home in the event that the participant is unable to come to the site), reading the questions aloud to the participant and entering the participant's responses on the participant's behalf. If the participant requires it, the participant's caregiver can assist the participant to complete the eDiary assessment by reading the eDiary questions aloud to the participant and recording the participant's responses in the eDiary following the procedures for caregivers (using the caregiver's unique identifier and PIN). Procedures for caregivers to collect and report the participant's responses to the eDiary questions will be detailed in the instructions for caregivers.

Participants will also be trained on when and how to collect a nasal swab if they experience any symptoms that could indicate an ARI. Participants will be provided with 2 nasal swab kits.

Over the entire study, the total blood volume scheduled to be collected from each participant in the Immuno Subset will be approximately 366 mL when the study is continued to Month 24, and

[a] Participants may access the eDiary via their own eDevice if their device (smartphone or tablet) is compatible.

244 mL when the study does not progress beyond the first year. For all other participants, the total blood volume scheduled to be collected will be 51 mL when the study is continued to Month 24, and 34 mL when the study does not progress beyond the first year. The maximum volume of blood to be drawn at any given visit would be 62 mL for participants in the Immuno Subset and 17 mL for all other participants. During each ARI episode, 21.5 mL additional blood will be sampled.

Repeat or unscheduled samples may be taken for safety reasons or for technical issues with the samples.

Patient-reported Outcomes

Participants will complete self-assessments of their health, symptoms, and functional status in questionnaires as well as ARI surveillance in an eDiary on their own eDevice or one provided for this study or desktop computer (where applicable).

Requirements for investigators and study monitors will be detailed in separate documents.

All ePRO instruments will be provided in the local language in accordance with local guidelines. The ePRO and AE data will not be reconciled with one another.

Visit Windows

The timings of post-vaccination visits will be determined relative to the actual day of vaccination.

The following visit windows will be allowed as indicated:

Visit #	Visit Day/ Visit Week	Visit Window	Primary Purpose
2	15/ 2	+3 days	2 weeks after vaccination, Safety and Immuno visit
2a[a]	29/ 4	+3 days	4 weeks after vaccination, Safety visit by telephone or telemedicine contact
2b[b]	85/ 12	±7 days	12 weeks after vaccination, Immuno visit
2c[b]	169/ 24	±14 days	24 weeks after vaccination, Immuno visit
3[c]	182/"End of first RSV season"/26	-7/+14 days	26 weeks after vaccination (Month 6)/End of season 1 assessments. Safety and efficacy visit
4	365/ 52	-7/+14 days	52 weeks after vaccination (Month 12). Safety and Immuno visit
4a[b]	533/ 76	±14 days	76 weeks after vaccination. Immuno visit
5	730// 104	-7/+14 days	104 weeks after vaccination (Month 24) assessments. Follow-up visit

a. Safety Subset only.
b. Immuno Subset only.
c. Please refer to the Schedule of Activities for the exact timing of visits and assessments to be performed.

Sample Collection and Handling

The actual dates and times of sample collection for the immunogenicity assessments must be recorded in the eCRF or laboratory requisition form.

Refer to the Schedule of Activities for the timing and frequency of all sample collections.

Instructions for the collection, handling, storage, and shipment of the samples can be found in the Laboratory Manual that will be provided. Collection, handling, storage, and shipment of the samples must be under the specified, and where applicable, controlled temperature conditions as indicated in the Laboratory Manual.

Study-Specific Materials

The investigator will be provided with the following supplies:

- Investigator's Brochure for Ad26/protein preF RSV vaccine
- Site Investigational Product Procedures Manual
- Investigational Product Preparation Instruction
- Laboratory Manual
- IWRS Manual
- Electronic Data Capture (eDC) Manual/eCRF completion guidelines
- Sample ICF
- ePRO completion guides
- ePRO participant information sheets
- eDiaries and instructions for use
- Rulers (to measure diameter of any erythema and swelling)
- Thermometers
- Nasal swab sample kits, including cooling bags
- RT-PCR machines and supplies for performing respiratory panel tests
- Pulse oximeters
- Laboratory kits
- Contact information page(s)

8.1. ARI Assessments and Procedures

From study vaccination until 24 months after vaccination (ie, full duration of the study), participants will be followed up to identify potential cases of RSV infection (refer to Appendix 7).

After administration of study vaccine, ARI surveillance will be conducted for the duration of the study. To help participants remember to report symptoms of a possible ARI, ideally on the day of

symptom onset, written instructions are provided. A reminder will be sent to the participants twice every week until the end of the study, to remind participants to fill out the ARI surveillance question, which is always available, and to answer the question if they have experienced any ARI symptoms, or for participants who have one or more of these symptoms at baseline, if they have experienced any additional ARI symptoms or a worsening of their baseline symptoms. It is expected that the participants answer these questions at least twice a week, preferably on the days that the reminder is sent. Participants who experience any symptoms suggestive of an ARI are instructed to indicate this (refer to Appendix 7) and start completing the required ARI episode questionnaires on a daily basis (preferably in the evening) (Appendix 8, Appendix 9, and Appendix 10). Ideally within the next 24 hours, the site and participant should have telephone or telemedicine contact with each other in order for the site to confirm that the participant is experiencing symptoms consistent with an ARI[a] and to give instructions to the participant on the ARI episode procedures to be followed (detailed in the subsequent section).

Figure 2: Schematic Overview of the ARI Assessments and Procedures

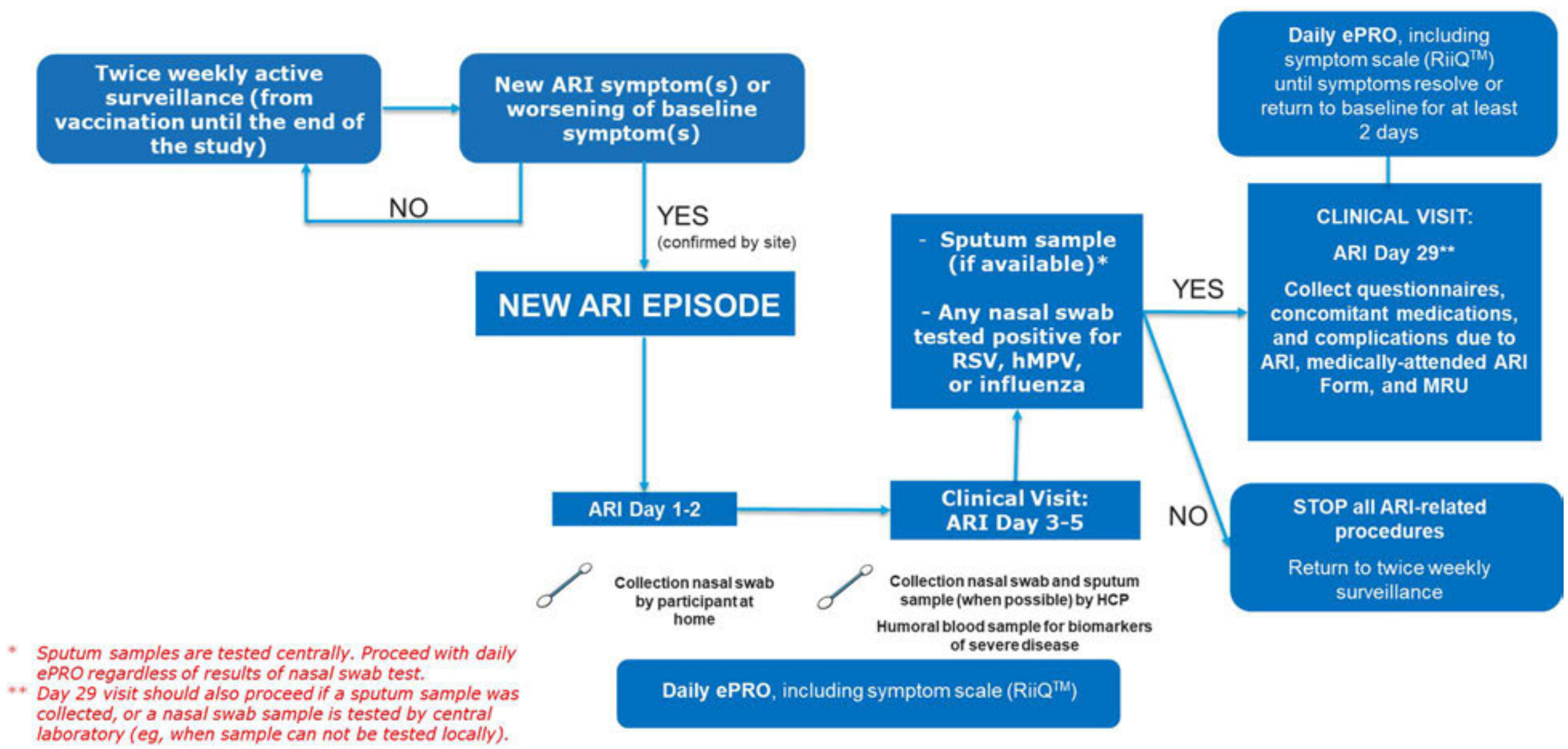

Participants with a sputum sample taken and participants with at least one nasal swab that cannot be tested at the site with the BioFire Filmarray Respiratory panel will follow the same procedures as participants with a RT PCR positive for RSV, hMPV, or influenza mediated ARI. Participants will remain blinded as to the outcome of RT PCR test results until study unblinding and the test results should only be disclosed in the event of safety concerns. RT PCR test results related to the detection of other respiratory pathogens can be communicated to the participants. The RT PCR results that are obtained with the BioFire Filmarray testing under the study procedures will be used for research purposes only and will not be used by the investigator for therapeutic decisions. A locally approved diagnostic test external to the study procedures, including the RSV RT PCR, should be obtained in case the participant's own healthcare provider considers it warranted (eg, based on symptoms and/or risk of severe disease) and/or according

---

[a] This will be assessed by the site by confirming that at least one of the symptoms used to confirm the ARI from the RiiQ Symptom Scale is worse than baseline. As defined in Appendix 5, symptoms used to confirm an ARI episode are: nasal congestion, sore throat, cough, short of breath, coughing up phlegm (sputum), and wheezing (from the RiiQ Symptom Scale). As it is possible that the participants will make errors when they answer the RiiQ Symptom Scale, the telephone or telemedicine contact will be used by the sites to also confirm the correctness of the answers provided by the participant and to confirm the ARI episode was correctly triggered.

to local/site specific guidelines. If a sample tests positive for SARS CoV 2, the participant will be referred to their own healthcare provider for locally approved diagnostic test and further management, in accordance with local/site specific guidelines.

In the setting that the site confirms that the reported symptoms do not qualify as an ARI episode, or that an ARI episode has ended despite long-term ongoing symptoms above baseline, the site staff can manually end an ARI episode. For all ARIs, a start date of the ARI should be entered into the eCRF. For all RSV, hMPV, or influenza-mediated ARIs, an end date of the ARI should be entered in the eCRF. For ARIs that are RSV, hMPV and influenza negative, the date that the site ends the ARI (after the RT-PCR test results are known) should also be entered in the eCRF.

Procedures for Participants who Experience an ARI Episode

When any participant experiences any symptoms of an ARI (see Appendix 5 Definition of Acute Respiratory Infection for Participants Aged ≥60 Years), the following will take place:

- The participant should indicate on the ARI Surveillance Assessment that the participant is experiencing new onset or worsening (in setting that baseline symptoms are present) symptoms of an ARI, ideally on the day of symptoms onset.
- The participant will contact the site as soon as possible if the participant experiences any symptoms suggestive of an ARI (ie, sore throat, nasal congestion, cough, short of breath, coughing up phlegm [sputum], wheezing), or the site will contact the participant by telephone or telemedicine, ideally within 24 hours if new onset or worsening of ARI symptoms are recorded in the eDiary and the participant has not called the site. During this telephone or telemedicine contact, the site may confirm if the reported symptoms qualify as ARI episode, in order to determine whether to proceed with the ARI procedures. The site should enter an ARI start date in the eCRF. The participant will then be reminded to:
- **On ARI Days 1 and 2 (day of ARI onset and day thereafter)**
  - Complete the RiiQ (Appendix 8) in the eDiary every evening (preferably), beginning on the evening of the day of symptom onset (ARI Day 1).
  - In addition to the RiiQ on those days, the eDiary will also ask participants to complete the following scales:
    - EuroQoL, 5-Dimension, 5-Level (EQ-5D-5L) (Appendix 9)
    - Patient Global Impression of Health (PGI-H) scale,
    - Patient Global Impression of Severity (PGI-S) scale,
    - Patient Global Impression of Change (PGI-C) scale, and
    - Return to Usual Health question (Appendix 10).
      Note that the PGI-C scale and Return to Usual Health question will not be completed on ARI Day 1.
  - Collect a nasal swab at home on the day of symptom onset or the day thereafter (ARI Day 1-2). If the participant requires it, the participant's caregiver can assist the participant to collect the nasal swab. Ensure the nasal swab collected at home is provided to the study staff within 4 days (preferably) after collection.

- **From ARI Day 3 Until the Diagnostic Results Are Known**
  - ➢ Continue completing the RiiQ (Appendix 8) (until RT-PCR results known, see below), in the eDiary every evening (preferably).

    Note that the RiiQ needs to be completed only once per day, ie, either at home or during the site visit, if applicable.
  - ➢ Continue completing the following scales (until RT-PCR results known, see below), consisting of:
    - EuroQoL, 5-Dimension, 5-Level (EQ-5D-5L) (Appendix 9)
    - Patient Global Impression of Health (PGI-H) scale,
    - Patient Global Impression of Severity (PGI-S) scale,
    - Patient Global Impression of Change (PGI-C) scale, and
    - Return to Usual Health question (Appendix 10).

    Note that the scales need to be completed only once per day, ie, either at home or during the site visit, if applicable.
- The site should schedule a clinical visit with the participant between 2 and 4 days after ARI symptom onset (ARI Day 3-5), or if a site visit is not feasible, a member of the study staff could visit the participant at home (or at the hospital, if needed) during this time frame.
- To reduce the burden on the participant and to avoid collection of blood samples (ie, serology blood sample, whole blood PAXgene sample and whole blood sample with PROT1 stabilizer), not related to an RSV/hMPV/influenza related ARI, participants and investigators can choose the preferred option during each Day 3-5 visit between: 1) Collecting blood samples before the nasal swab samples are tested or in the setting where a sputum sample is collected, independent of the RT-PCR results; and 2) Collecting blood samples after the nasal swab RT-PCR results are available, according to nasal swab test results, and for ARIs where there is no sputum sample collection. If this is the preferred option by the participant and investigator, the participant will wait at the site for the time needed to test the swab samples (estimated time: 2 hours). In this scenario, if the swab samples are negative for RSV, hMPV and influenza AND if no sputum sample can be collected, the blood sample collection at the ARI Day 3-5 timepoint can be omitted. If the swab samples are positive for RSV, hMPV or influenza OR if sputum sample is collected, the blood samples collection must be performed. Whenever a sputum sample is collected during the ARI Day 3-5 visit, the blood sample must still be collected as the RT-PCR test on the sputum sample will be performed at the central laboratory and not on site.
- During the ARI Day 3-5 visit, vital signs will be measured, and a nasal swab, a sputum sample (in participants with a productive cough, when possible), and blood samples (to measure the immune responses at the time of exposure and for exploration of biomarkers that correlate with respiratory infection and disease severity) may be taken by a member of the study staff.
- Ideally within 24 hours after the ARI Day 3-5 visit (but no later than 48 hours after the ARI Day 3-5 visit), a qualified member of the study staff will perform a local RT-PCR of all available nasal swabs with the BioFire® Filmarray Respiratory panel, which will be provided to all sites (both US and non-US); changes to the process might be implemented at the local

level based on local requirements. Participants will subsequently be contacted by telephone or telemedicine and given further instructions, as detailed below, on how to further proceed based on the outcome of the RT-PCR tests.

Sputum samples (where applicable) will be sent to the central laboratory for testing using the BioFire Filmarray Pneumonia (PN) panel. For all ARI episodes where a sputum sample is collected, the ARI episode will be followed up until ARI resolution (ie, with daily questionnaires and an ARI Day 29 visit), independent of the test results from the collected nasal swabs. Participants who are under self-isolation because of a (possible) positive test for SARS-CoV-2 will not come to the site for ARI Day 3-5 assessments but will self-collect the nasal swab.

- In the unlikely event where at least one nasal swab from an ARI episode cannot be tested at the site with the BioFire Filmarray Respiratory panel (eg, in the setting of instrument malfunction, unavailability of the device in the country/territory or at the site, or shortage of testing reagents at the site), this sample(s) should be sent to the central laboratory for testing with the BioFire Filmarray Respiratory panel, and the ARI episode will be followed up until ARI resolution (ie, with daily questionnaires and an ARI Day 29 visit), independent of the results from other collected samples for that ARI episode. Further details on the strategy for local site and central testing of the nasal swabs and sputum samples are provided in Section 8.2.4 below.

- For participants with a positive SARS-CoV-2 test and a negative test for RSV, hMPV, and influenza, the collection of ARI data by the study site will be stopped for the ARI episode. The participant will be informed by telephone or telemedicine contact that they can stop filling out the daily ARI episode questionnaires, and the site will end the ARI episode and return the participant to ARI surveillance with twice weekly reminders. Participants who have a positive SARS-CoV-2 RT-PCR test as part of the study procedures should be referred to their own healthcare provider for follow-up diagnostics and further management, in accordance with local/site specific guidelines. Participants whose standard-of-care testing during an ARI episode is positive for SARS-CoV-2, or who have been in contact with someone that tested positive for SARS-CoV-2 and therefore must self-isolate according to the local guidelines, will not come to the site for ARI Day 3-5 assessments but will self-collect the nasal swab. In this circumstance, other ARI Day 3-5 assessments (vital signs, sputum sample and blood samples) might not be performed.

Participants with positive RT-PCR results for RSV (or hMPV or influenza) should keep on filling out the daily ARI episode questionnaires until resolution of the ARI episode independently of any other co-infection (including SARS-CoV-2).

Further Procedures for Participants with an RSV, hMPV, or Influenza-mediated ARI[a]

- Continue completing the RiiQ (Appendix 8) until resolution* of the ARI episode in the eDiary every evening (preferably).
- Continue completing the following scales until resolution* of the ARI episode:
  - EuroQoL, 5-Dimension, 5-Level (EQ-5D-5L) (Appendix 9),
  - Patient Global Impression of Health (PGI-H) scale,
  - Patient Global Impression of Severity (PGI-S) scale,
  - Patient Global Impression of Change (PGI-C) scale, and
  - Return to Usual Health question (Appendix 10).

  * A resolved ARI episode is defined as 2 consecutive days with no symptoms listed on the RiiQ Symptom Scale, or, for participants who have RiiQ symptoms at baseline (assessed pre-vaccination), 2 consecutive days where all symptoms on the RiiQ Symptom Scale have returned to the same severity level as reported at baseline (or lower). After resolution of the ARI episode, the participant will return to ARI surveillance with twice weekly reminders.
- On ARI Day 29 (±7 days), participants will be asked to return to the site if a sputum sample was provided by the participant at ARI Day 3-5 visit, regardless of the swab RT-PCR test results, AND/OR at least one of the nasal swab samples collected could not be tested at the study site with the BioFire Filmarray Respiratory panel (RT-PCR test) and had to be shipped for central laboratory test AND/OR at least one nasal swab sample had a positive RT-PCR result for either RSV, hMPV, or influenza when tested at the study site. The ARI Day 29 visit can take place by telephone or telemedicine contact. During this visit:
  - The MRU questionnaire will be completed (Appendix 11) based on interview with the participant.
  - Participants will complete the RiiQ (Appendix 8), the EQ-5D-5L (Appendix 9) and each of the PGI scales and the Return to Usual Health question (Appendix 10) on the participant's eDiary.
  - The site staff will also collect information on days missed from work (Appendix 12) and on complications, hospitalizations and concomitant medications related to the ARI and record in the eCRF.
  - If the ARI episode is resolved by the time of the ARI Day 29 visit, the investigator should enter the end date in the CRF during the ARI Day 29 visit.
  - If the ARI episode is ongoing at the time of the ARI Day 29 visit, the participant should continue filling in the ARI questionnaires until the end of the ARI episode. The

[a] Participants with a sputum sample taken and participants with at least one nasal swab that cannot be tested at the site with a BioFire Filmarray Respiratory panel will follow the same procedures as participants with a RT PCR positive for RSV, hMPV, or influenza mediated ARI.

investigator should follow up with the participant to collect the ARI end date and enter it in the CRF.

- For medically-attended RT-PCR positive for RSV, hMPV, or influenza-mediated ARIs, including those resulting in hospitalization, a standard question list will be provided (Appendix 6) to collect additional information on any other diagnostic tests (eg, chest x-rays, spirometry, pulmonary function tests, etc.) or on interventions during the clinical course of the ARI.

Further Procedures for Participants with an ARI that is Negative for RSV, hMPV, and Influenza

- The site will end the ARI episode and inform the participant by telephone or telemedicine that they can stop filling out the daily ARI episode questionnaires.
- The date that the site ends the ARI (after the RT-PCR test results are known) should also be entered in the eCRF.
- The participant will return to ARI surveillance with twice weekly reminders 10 days after the day of onset of this ARI.

All Participants

At the "End of first RSV season" visit, all participants will complete the RiiQ, the PGI-H and the EQ-5D-5L in their eDiary.

Throughout the study, if an expected eDiary recording has been missed by a participant, the participant or the participant's caregiver should be contacted the next morning to confirm that the eDiary is being completed as required.

Throughout the study, RT-PCR results from the nasal swabs and sputum sample (where available) will be used to determine whether the infection was caused by RSV. If at least one of these samples is positive for RSV, the collected information will be applied against the clinical case definition.

Local RT-PCR testing will be performed using the BioFire Filmarray Respiratory panel. In case the BioFire Filmarray Respiratory panel cannot be performed at the site (eg, in the setting of instrument malfunction, unavailability of the device in the country/territory or at the site, or shortage of testing reagents at the site), central RT-PCR testing with the BioFire Filmarray Respiratory panel will be used as a backup option.

For participants hospitalized with symptoms of an ARI, confirmation of RSV infection using a locally approved RT-PCR test at the hospital laboratory can also be used. These results will be used in the primary analysis only if approved by an independent expert with expertise in diagnostic testing. The approval of the RT-PCR results by the expert will be based on the specifications of the test performed, the qualifications of the testing laboratory and the procedures followed in the laboratory.

8.1.1. Respiratory Infection Intensity and Impact Questionnaire (RiiQ™v2)

The RiiQ will be used to identify possible ARI episodes, to define cases of RSV-mediated LRTD (for participants with RT-PCR-confirmed RSV infection), to evaluate severity of RSV disease

episodes and assess the impact of an ARI episode on the participant's daily life (see Appendix 8). The RiiQ was adapted for RSV based on the Flu-iiQ™ originally developed and validated for the monitoring of influenza symptoms and their impact in vaccine studies (Osborne 2011, van Essen 2014). The RiiQ includes 3 additional lower respiratory tract symptoms common in patients with RSV-related illness and 1 additional daily activity item to address limitations in opportunity for activity for patients hospitalized for treatment of RSV disease.

The RiiQ asks participants to rate level of severity or impairment on a 4-point scale with higher scores indicating greater severity or negative impact of an ARI episode. The questions ask the participants to report symptoms at their worst during the past 24 hours to monitor the severity of RSV-related illness, but also identify pre-existing symptoms and functional limitations at baseline associated with comorbidities common in adults at risk for more severe illness due to RSV infection.

The RiiQ consists of 4 scales that are scored separately:

- RiiQ Symptom Scale (Question 1). Each symptom is rated on the following scale: 0 None, 1 Mild, 2 Moderate, and 3 Severe. The RiiQ Symptom scale can be scored as a mean of all scores (Total Symptom Score) or 2 subscales scores, Respiratory Symptoms and Systemic Symptoms:

  The RiiQ Respiratory Symptoms Subscale score is the mean of 6 symptoms, ie, 2 upper respiratory tract infection (URTI) symptoms (Nasal congestion and Sore throat) and 4 lower respiratory tract infection (LRTI) symptoms (Cough, Wheezing, Shortness of breath, and Coughing up phlegm/sputum)

  The RiiQ Systemic Symptom subscale score is the mean of 7 systemic symptoms (Headache, Feeling feverish, Neck pain, Body aches and pain, Fatigue/tiredness, Interrupted sleep, and Loss of appetite).

- RiiQ Impact Scales (Questions 2 to 4):

  RiiQ Impact on Daily Activity scale (Question 2) consists of 7 activities. Ability to perform each activity item is rated on the following scale: 0 No difficulty, 1 Some difficulty, 2 Moderate Difficulty, and 3 Great difficulty. Scale scores are calculated as the mean of all 7 items (range 0-3).

  RiiQ Impact on Emotions scale (Question 3) consists of rating 4 negative emotions, rated on the following scale: 0 Not at all, 1 Somewhat, 2 Moderately, and 3 Extremely. Scale scores are calculated as the mean of all 4 items (range 0-3).

  RiiQ Impact on Relationships scale (Question 4) consists of 5 problems that a respiratory infection may cause in relationships with others, rated on the following scale: 0 Not at all concerned, 1 Somewhat concerned, 2 Moderately concerned, and 3 Extremely concerned) with scale scores calculated as the mean of all 5 items (range 0-3).

In the setting of the present study, only the RiiQ Symptom Scale (Question 1) and the RiiQ Impact on Daily Activities Scale (Question 2) are being used (Appendix 8).

8.1.2. EuroQoL, 5-Dimension, 5-Level Questionnaire

The EQ-5D-5L questionnaire is a standardized instrument for use as a measure of health outcome, primarily designed for self-completion by the participant. It consists of the EQ-5D-5L descriptive system and EQ visual analogue scale (VAS) (see Appendix 9). The descriptive system includes the following dimensions: mobility, self-care, usual activities, pain/discomfort, and anxiety/depression. Each of these 5 dimensions is divided into 5 levels of perceived problems (1 no problem, 2 slight problems, 3 moderate problems, 4 severe problems; 5 extreme problems) (https://euroqol.org/eq-5d-instruments/).

8.1.3. Patient Global Impression Scores

Patient Global Impression scores (Appendix 10) developed for this study will be collected. The questions will be used to evaluate overall health.

- Patient Global Impression of Health (PGI-H). Participants report their overall impression of their health status today on the following scale: 0 Very good, 1 Good, 2 Fair, 3 Poor, 4 Very poor.
- Patient Global Impression of Severity (PGI-S). Participants rate the severity of their respiratory illness on the following scale: 0 None, 1 Mild, 2 Moderate, 3 Severe.
- Patient Global Impression of Change (PGI-C). Participants rate the amount of change in their health each day during an ARI episode on the following scale: -3 Much better, -2 Somewhat better, -1 A little better, 0 About the same, 1 A little worse, 2 Somewhat worse, 3 Much worse
- Return to Usual Health. Participants are being asked whether they have returned to their usual health after developing symptoms suggesting an ARI.

8.2. Efficacy Assessments

In the below sections, new onset or worsening of symptoms is defined as follows:

- Symptoms present during an ARI episode but not at the baseline assessment will be considered as symptoms with a new onset.
- Symptoms present at the baseline assessment that become worse (higher severity) during the ARI episode will be considered as worsening symptoms.

The Day 1 assessment is considered the baseline.

Confirmation of RSV infection by RT-PCR for the primary endpoint will be done in nasal swabs or sputum samples.

- Confirmation of RSV infection by RT-PCR in nasal swabs will be performed at the site laboratory with the BioFire Filmarray Respiratory panel, which will be provided to all sites (both US and non-US). Both the home swab and the site swab will be analyzed at the site. The central RSV confirmation of the nasal swab with the BioFire Filmarray Respiratory panel, will not be taken into account for the primary endpoint, unless in the unlikely event where at least one nasal swab from an ARI episode cannot be tested at the site (eg, in the setting of

instrument malfunction, unavailability of the device in the country/territory or at the site, or shortage of testing reagents at the site).

- Confirmation of RSV infection by RT-PCR in sputum samples (when collected) will be performed in the central laboratory with the BioFire Filmarray PN panel.
- For participants hospitalized with symptoms of an ARI, confirmation of RSV infection using a locally approved RT-PCR test at the hospital laboratory can also be used. These results will be used in the primary analysis only if approved by an independent expert with expertise in diagnostic testing. The approval of the RT-PCR results by the expert will be based on the specifications of the test performed, the qualifications of the testing laboratory and the procedures followed in the laboratory.

One positive sample (defined as any sample with a value above the limit of detection) is sufficient.

The first occurrence of a considered endpoint is defined as the start of the first episode of the endpoint in the study (regardless of RSV A or B strain, unless otherwise specified).

8.2.1. Case Definition for RT-PCR-confirmed RSV-mediated LRTD

A participant will be considered to have RT-PCR-confirmed RSV-mediated LRTD if the following criteria are met:

- New onset or worsening from baseline of 3 or more of the below symptoms as captured on the RiiQ at the same assessment time point:

  Cough

  Short of breath

  Coughing up phlegm (sputum)

  Wheezing

 AND

- Confirmation of RSV by RT-PCR in one or more of the nasal swabs, or in the sputum sample (when available)

Counting of the number of symptoms with new onset or worsening will be done per assessment. If a participant completed the RiiQ more than once per day, the case definitions cannot be met by combining symptoms from different assessments on that day, the required number of symptoms must be attained at one assessment.

This definition will be programmed. Prior to database lock for all planned analyses (see Section 9.5), the CEC will review in a blinded manner all available data of all RSV-mediated ARIs and will confirm if the above criteria are met. If the CEC confirms that the above criteria for LRTD are met, the case will be counted as a primary endpoint in the statistical analysis.

8.2.2. Case Definition for RT-PCR-confirmed RSV-mediated ARI

A participant will be considered to have RT-PCR-confirmed RSV-mediated ARI if the following criteria are met:

- ARI episode initiated by the participant and confirmed by the site with symptoms consistent with an ARI (new symptoms or worsening from baseline of at least one of the symptoms as captured on the RiiQ):

  Sore throat

  Nasal congestion

  Cough

  Short of breath

  Coughing up phlegm (sputum)

  Wheezing

AND

- Confirmation of RSV by RT-PCR in one or more of the nasal swabs, or in the sputum sample (when available)

8.2.3. Predefined Clinically Relevant Disease Associated with RT-PCR-confirmed RSV-mediated ARI

A participant will be considered to have clinically relevant disease with specific parameters associated with an RT-PCR-confirmed RSV-mediated ARI if the following criteria are met:

- The participant has an RT-PCR-confirmed RSV-mediated ARI as defined in Section 8.2.2

- Any of the following is associated with the ARI:

  Hospitalization

  Emergency department visit

  Per clinical judgement, at least one of the following complications: asthma exacerbation, COPD exacerbation, respiratory distress, bronchitis, bronchial hyperreactivity, CHF exacerbation, cardiac arrhythmia, renal impairment or the presence of X-ray or radiological confirmed pneumonia, respiratory arrest and/or failure, pulmonary embolism, pleural effusion, atelectasis, acute coronary events, acute cerebrovascular events, altered mental status, seizure, syncope, systemic inflammatory response syndrome (SIRS), new neurological deficit, asthenia, dehydration or metabolic disturbances

  For measurements at the site, decreased oxygen saturation is defined as oxygen saturation of <92% for participants with a baseline oxygen saturation of ≥92% at randomization; for participants with baseline oxygen saturation <92%, decreased oxygen saturation is

defined as a ≥3% decrease in their oxygen saturation from baseline[a].
Decreased oxygen saturation based on measurements during medically-attended ARI (ie, hospitalization) is defined as oxygen saturation of <92% supporting LRTD.

Tachypnea (at least emerging Grade 2, see Appendix 4: Toxicity Grading Scale)

Need of supplemental oxygen

Hypotension (emerging Grade 3, see Appendix 4: Toxicity Grading Scale)

Pulmonary function test results supporting diagnosis of LRTD

Arterial blood gas results supporting diagnosis of LRTD

8.2.4. Diagnosis of RSV and Other Respiratory Infections

After initiation of an ARI episode, the nasal swabs taken after symptom onset (ARI Day 1-2 and ARI Day 3-5) will be analyzed at the local study site using an FDA -approved rapid RT-PCR based assay (BioFire Filmarray Respiratory panel) to determine any positivity for RSV, hMPV, or influenza virus. In the unlikely event where at least one nasal swab from an ARI episode cannot be tested at the site with the BioFire Filmarray Respiratory panel (eg, in the setting of instrument malfunction, unavailability of the device in the country/territory or at the site, or shortage of testing reagents at the site), this sample(s) should be sent to the central laboratory for testing with the BioFire Filmarray Respiratory panel.

For ARIs where the optional sputum sample is collected at ARI Day 3-5, this sputum sample will be shipped for central testing using the FDA-approved rapid RT-PCR-based assay (BioFire Filmarray PN panel) to determine any positivity for RSV, hMPV, or influenza virus.

All nasal swabs tested at the local study sites which are positive for RSV will be shipped to the central laboratory for positive confirmation of RSV, using the BioFire Filmarray Respiratory panel.

For all RSV positive samples (based on the results from both local testing for nasal swabs and central testing on sputum samples and nasal swabs), the RSV subtype and viral load will be also determined using the DDL qRT-PCR assay for RSV. For nasal swabs and sputum samples, the presence of other respiratory pathogens will also be determined by the BioFire Filmarray Respiratory panel and the BioFire Filmarray PN panel, respectively.

Table 3 shows strategy for local study site and centralized testing of the nasal swabs and sputum samples collected.

[a] Based on vital signs collected at baseline and during the ARI at the site.

Table 3: Strategy for Local Site and Central Testing of the Nasal Swabs and Sputum Samples

	Central[a]/ Local Site Testing	Purpose of the Test	Type of Samples
BioFire Filmarray RP2.1 EZ[d]	Local site	Presence of RSV, hMPV or influenza virus	All nasal swab samples
BioFire Filmarray Pneumonia panel	Central	Presence of RSV, hMPV or influenza virus	All sputum samples
BioFire Filmarray 2.0	Central	Presence of RSV, hMPV or influenza virus	Nasal swab samples[b]
BioFire Filmarray 2.0	Central	Confirmation of RSV	Nasal swab samples[c]
DDL qRT PCR	Central	RSV subtype, viral load	RT PCR+ nasal swabs and sputum samples

Note: The BioFire Respiratory panel and Pneumonia panel will test for a panel of viral and bacterial infections.

a DDL Diagnostic Laboratory (Rijswijk, The Netherlands)

b Test will be performed on nasal swab samples that cannot be tested at the site only

c Confirmation will be performed on local site RSV positive nasal swab samples only

d When the BioFire Filmarray RP2.1 EZ panel is not locally approved in a region and its use under research use only is not approved in that region, another locally approved version may be used at the local site, provided that it has an equivalent performance for detecting the pathogens of interest (ie, RSV, hMPV and influenza) with the BioFire Filmarray RP2.1 EZ panel.

Participants will remain blinded as to the outcome of RSV, hMPV and influenza RT-PCR test results until study unblinding and the test results should only be disclosed in the event of safety concerns, with the exception of SARS-CoV-2 infection. If a sample tests positive for SARS-CoV-2, the participant will be referred to their own healthcare provider for locally approved diagnostic test and further management, in accordance with local/site specific guidelines. RT-PCR test results related to the detection of other respiratory pathogens can be communicated to the participants.

For participants hospitalized with symptoms of an ARI, confirmation of RSV infection using a locally approved RT-PCR test at the hospital laboratory can also be used. These results will be used in the primary analysis only if approved by an independent expert with expertise in diagnostic testing. The approval of the RT-PCR results by the expert will be based on the specifications of the test performed, the qualifications of the testing laboratory and the procedures followed in the laboratory. In these instances, attempts will be made to obtain a sample to ship to the central laboratory for positive confirmation of the RSV infection with the BioFire Filmarray Respiratory assay, RSV subtyping, viral load using the DDL qRT-PCR assay, and, if applicable, viral sequencing.

8.3. Immunogenicity Assessments

Blood samples will be collected from all participants for humoral immunogenicity assessments before and 14 days after vaccination, and at 1 year after vaccination for the analysis of correlates of risk of RSV disease and correlates of protection.

For participants in the Immuno Subset (consisting of at least 360 participants, of whom ~50% will be at increased risk for severe RSV disease and ~50% will be 75 years or older), blood will be collected for analysis of humoral and cellular immune responses before study vaccination and at 2, 12, and 24 weeks, and 1 and 1.5 years after vaccination.

Immunogenicity assessments may include (but are not limited to) the assays summarized below.

Humoral Assays	Purpose
Secondary endpoints	
RSV neutralization A	Analysis of neutralizing antibodies to an A strain
F protein antibodies (ELISA; pre F and/or post F)	Analysis of antibodies binding to RSV F protein in pre fusion and/or post fusion form
Exploratory endpoints	
RSV strain cross neutralization	Analysis of cross neutralizing antibodies to B and/or a different A strain(s) using virus neutralization assays and other surrogate antibody binding assays
Adenovirus neutralization assay	Analysis of neutralizing antibodies to adenovirus
Functional and molecular antibody characterization	Analysis of antibody characteristics including, but not limited to, ADCC, ADCP, avidity, epitope mapping, Ig isotype, functional VNAs to other respiratory viruses, and antibody assessments for antibody repertoire

ADCC=antibody dependent cell mediated cytotoxicity, ADCP=antibody dependent cellular phagocytosis, ELISA =enzyme linked immunosorbent assay, F=fusion, Ig=immunoglobulin, VNA=virus neutralizing antibody
Note: Antibody analyses may be performed in nasosorption samples and serum.

Cellular Assays [a]	Purpose
Secondary endpoints	
IFN γ ELISpot	T cell IFN γ responses to RSV F protein peptides
Exploratory endpoints	
Immunophenotyping	May include analysis of T cell and B cell responses to RSV F protein peptide stimulated PBMC (including, but not limited to, $CD4^+$/$CD8^+$, Th1/Th2, IL 2, IFN γ, TNF α, activation markers and memory)
Immune repertoire analysis	May include using antigen specific single cell sequencing or bulk sequencing in order to assess T cell and B cell repertoire analysis

[a] Cellular assays will be performed in the Immuno Subset only where PBMCs are collected.
ELISpot=enzyme linked immunospot, F=fusion; IFN γ=interferon gamma, IL 2 =interleukin 2, PBMC=peripheral blood mononuclear cells, TNF α=tumor necrosis factor alpha

Nasosorption samples using synthetic absorptive matrix (SAM) will be collected from Immuno Subset participants prior to vaccination and at the 2 weeks and 1-year post-vaccination visits and will be used for immunogenicity assessments that may include antigen-specific immunoglobulins (ie, IgG and IgA), microbiome, and other viral pathogen analyses.

The blood samples that are collected between 2 and 4 days after symptom onset (ARI Day 3-5) from participants with an RSV, hMPV, or influenza-mediated ARI episode will be assayed by serology (which may include pre-F/post-F enzyme-linked immunosorbent assay [ELISA] and RSV VNA, as available and applicable) to assess immune responses and correlates that are associated with RSV infection. The samples will also be used for exploration of biomarkers that correlate with RSV infection and RSV disease severity (which may include RT-PCR for RSV, ribonucleic acid [RNA] transcriptomics to assess gene regulation [clusters] and expression patterns, cytokine/chemokine-dependent immunophenotyping, and immune repertoire analysis, as available and applicable).

8.4. Safety Assessments

For All Participants

All (S)AEs and special reporting situations related to study procedures or to non-investigational (concomitant) Janssen products will be collected from ICF signature onwards until the end of the

study for all participants. All other SAEs and AESIs (see Section 8.5.6) will be collected from administration of study vaccine (Day 1) until 6 months after vaccination. AEs leading to discontinuation from the study will be collected for the duration of the study. All COVID-19 cases will be collected for all participants for the duration of the study.

ARIs and complications related to RSV, hMPV, and influenza-mediated ARIs that classify as SAEs will be captured in all participants and reported as SAEs in the eCRF continuously from the time of vaccination until 24 months after vaccination.

All participants will be closely observed for a minimum of 15 minutes after vaccination to monitor for the development of any acute reactogenicity. This post-vaccination observation period should be extended for participants whom in the investigator's opinion would require further observation or for any occurrence of a localized reaction or (minor) systemic symptoms during the post-vaccination observation that require monitoring for any progression.

For Participants in the Safety Subset

Additional procedures will be carried out in the Safety Subset participants; ~50% of the participants in the Safety Subset will be at increased risk of severe RSV disease and ~50% will be 75 years or older (Section 6.3, Measures to Minimize Bias: Randomization and Blinding). Safety Subset participants will have provided consent for the additional study procedures[a]:

- At the end of the 15-minute observation period, vital signs (body temperature, blood pressure, heart rate, respiratory rate, oxygen saturation) will be obtained[b], and any unsolicited and solicited local and systemic AEs will be documented in the eCRF by study personnel.
- Participants will record solicited local (at the injection site) AEs, solicited systemic AEs, and body temperatures in the eDiary, beginning on the evening of the vaccine dosing day and on a daily basis for the following 7 days. If a solicited local or systemic AE is not resolved within 7 days after vaccination (Day 8), safety follow-up should be performed until the symptom resolves. The study-site personnel will be instructed to record the date of last symptoms and maximum severity until resolution in the source document and eCRF.
- All other AEs (unsolicited) and special reporting situations will be reported from the time of vaccination through the following 28 days. Participants will be contacted by telephone or telemedicine at 28 days (+3 days) after vaccination to collect information on unsolicited AEs and associated concomitant medications.
- All ARIs and all complications related to RSV, hMPV, and influenza-mediated ARIs that do not classify as SAEs will be reported as AEs in the eCRF from the time of vaccination through the following 28 days.

For more details on AE reporting, refer to Section 8.5, Adverse Events, Serious Adverse Events, and Other Safety Reporting. In addition to AE reporting, the study includes the following safety evaluations of safety according to the timepoints provided in the Schedule of Activities.

[a] Note that participants can participate in both the Immuno Subset and the Safety Subset.
[b] Actual values (normal and abnormal) will only be documented in the eCRF for body temperature.

8.4.1. Physical Examinations

At any clinic visit, an abbreviated, symptom-directed physical examination may be performed if deemed necessary by the investigator based on any clinically relevant issues, clinically relevant symptoms, and medical history.

Physical examinations will be performed by the investigator or appropriately trained delegate. Any clinically relevant abnormalities or changes in severity noted during the review of body systems should be documented in the eCRF as an AE or SAE if it meets the criteria for an AE or SAE according to the protocol reporting requirements.

8.4.2. Vital Signs

Vital signs will be collected prior to vaccination for all participants:

- Heart rate, respiratory rate, sitting systolic blood pressure, sitting diastolic blood pressure Note: vital signs will also be collected post-vaccination from participants in the Safety Subset.
- Body temperature (oral route preferred, or in accordance with the local standard of care). Note: Additional temperature recordings will be collected from participants in the Safety Subset (until 7 days post-vaccination in the eDiary).
- Oxygen saturation (SpO_2)
- Height (at screening only) and weight

At non-vaccination visits, vital signs will be measured if deemed necessary by the investigator. Any clinically relevant abnormalities or changes in severity should be documented in the eCRF as an AE or SAE if it meets the criteria for an AE or SAE according to the protocol reporting requirements.

8.5. Adverse Events, Serious Adverse Events, Adverse Events of Special Interest, and Other Safety Reporting

Timely, accurate, and complete reporting and analysis of safety information, including (S)AEs, AESIs (including potential AESIs, refer to Section 8.5.6) and Product Quality Complaints (PQCs), from clinical studies are crucial for the protection of participants, investigators, and the sponsor, and are mandated by regulatory agencies worldwide. The sponsor has established Standard Operating Procedures in conformity with regulatory requirements worldwide to ensure appropriate reporting of safety information; all clinical studies conducted by the sponsor or its affiliates will be conducted in accordance with those procedures.

Further details on AEs, SAEs, AESIs, and PQCs can be found in Appendix 3: Adverse Events, Serious Adverse Events, Adverse Event of Special Interest, Product Quality Complaints, and Other Safety Reporting: Definitions and Procedures for Recording, Evaluating, Follow-up, and Reporting.

8.5.1. Time Period and Frequency for Collecting Adverse Event, Serious Adverse Event, and Adverse Event of Special Interest Information

All Adverse Events

AEs and special reporting situations, whether serious or non-serious, that are related to study procedures or that are related to non-investigational sponsor products will be reported from the time a signed and dated ICF is obtained until the end of the study/early withdrawal.

Clinically relevant medical events not meeting the above criteria and occurring between signing of ICF and moment of vaccination will be collected on the Medical History eCRF page as pre-existing conditions.

Solicited AEs, collected through an eDiary, will be recorded from the time of vaccination until 7 days post-vaccination for Safety Subset participants.

All other unsolicited AEs and special reporting situations, whether serious or non-serious, will be reported from the time of vaccination until 28 days post-vaccination for Safety Subset participants. Unsolicited AEs with the onset date outside the timeframe defined above (>28 days after study vaccination), which are ongoing at Month 12, should be recorded as such for Safety Subset participants only.

All AEs leading to discontinuation from the study (regardless of the causal relationship) are to be reported from administration of study vaccine until the end of the study. The sponsor will evaluate any safety information that is spontaneously reported by an investigator beyond the time frame specified in the protocol.

All COVID-19 cases will be collected for all participants for the duration of the study.

All AEs will be followed until resolution or until clinically stable.

Serious Adverse Events

All SAEs within 6 months after vaccination, as well as PQCs, occurring during the study must be reported to the appropriate sponsor contact person by study site personnel within 24 hours of their knowledge of the event.

SAEs, including those spontaneously reported to the investigator within 6 months after vaccination must be reported. The sponsor will evaluate any safety information that is spontaneously reported by an investigator beyond the time frame specified in the protocol.

Information regarding SAEs will be transmitted to the sponsor using the SAE form and Safety Report form of the eCRF, which must be completed and reviewed by a physician from the study site, and transmitted to the sponsor within 24 hours. The initial and follow-up reports of an SAE should be transmitted electronically or by facsimile (fax). Telephone reporting should be the exception and the reporter should be asked to complete the appropriate form(s) first.

AESIs

TTS is considered to be an AESI. Thrombotic events and/or thrombocytopenia (defined as platelet count below the lower limit of normal [LLN] range for the testing lab) are considered to be potential AESIs. All AESIs, including potential AESIs, will be reported to the sponsor within 24 hours of awareness from the moment of first study vaccination until 6 months after study vaccination (see Section 8.5.6).

8.5.2. Method of Detecting Adverse Events, Serious Adverse Events, and Adverse Events of Special Interest

Care will be taken not to introduce bias when detecting (S)AEs or AESIs. Open-ended and nonleading verbal questioning of the participant is the preferred method to inquire about AE occurrence.

Solicited Adverse Events (for Participants in the Safety Subset Only)

Solicited AEs are used to assess the reactogenicity of the study vaccine and are predefined local (at injection site) and systemic events for which the participant is specifically questioned and which are noted by participants in their eDiary.

After vaccination, participants will remain under observation at the study site for at least 15 minutes and will be closely observed by the study staff. Solicited AEs will be documented by study site personnel following the 15 minutes post-vaccination observation period for participants in the Safety Subset.

In addition, participants in the Safety Subset will record solicited signs and symptoms in an eDiary for 7 days post-vaccination. Participants will be provided with an eDiary and instructions on how to complete the eDiary (refer to Overview in Section 8, Study Assessments and Procedures). Electronic diary information will be transferred from the eDiary source to the sponsor. After review and verbal discussion of the initial eDiary entries with the participant, the investigators will complete their own assessment in the relevant sections of the eCRF. Once a solicited sign or symptom from an eDiary is considered to be of severity Grade 1 or above, it will be recorded as a solicited AE.

Solicited Injection Site (Local) Adverse Events

Participants in the Safety Subset will be asked to note in the eDiary occurrences of injection site pain/tenderness, erythema and swelling at the study vaccine injection site daily for 7 days post-vaccination (day of vaccination and the subsequent 7 days). The extent (largest diameter) of erythema and swelling should be measured (using the ruler supplied) and recorded daily. Case definitions for solicited injection site events can be found in the references (Gidudu 2012, Kohl 2007).

Solicited Systemic Adverse Events

Participants in the Safety Subset will be instructed on how to record daily temperature using a thermometer provided for home use. Participants should record the temperature in the eDiary in

the evening of the day of vaccination, and then daily for the next 7 days approximately at the same time each day. If more than one measurement is made on any given day, the highest temperature of that day will be used in the eCRF.

Fever is defined as endogenous elevation of body temperature ≥38° C, as recorded in at least one measurement (Marcy 2004).

Participants in the Safety Subset will also be instructed on how to note signs and symptoms in the eDiary on a daily basis for 7 days post-vaccination (day of vaccination and subsequent 7 days) for the following events: fatigue, headache, nausea, and myalgia.

Unsolicited Adverse Events

Unsolicited AEs are all AEs for which the participant is not specifically questioned in the participant eDiary.

For details regarding AESIs, refer to Section 8.5.6.

8.5.3. Follow-up of Adverse Events, Serious Adverse Events, and Adverse Events of Special Interest

The investigator is obligated to perform or arrange for the conduct of supplemental measurements and evaluations as medically indicated to elucidate the nature and causality of the (S)AE, AESI, or PQC as fully as possible. This may include additional laboratory tests or investigations, histopathological examinations, or consultation with other healthcare professionals.

AEs, including pregnancy, will be followed by the investigator as specified in Appendix 3: Adverse Events, Serious Adverse Events, Adverse Event of Special Interest, Product Quality Complaints, and Other Safety Reporting: Definitions and Procedures for Recording, Evaluating, Follow-up, and Reporting.

8.5.4. Regulatory Reporting Requirements for Serious Adverse Events

The sponsor assumes responsibility for appropriate reporting of AEs to the regulatory authorities. The sponsor will also report to the investigator (and the head of the investigational institute where required) all suspected unexpected serious adverse reactions (SUSARs). The investigator (or sponsor where required) must report SUSARs to the appropriate IEC/IRB that approved the protocol unless otherwise required and documented by the IEC/IRB. A SUSAR will be reported to regulatory authorities unblinded. Participating investigators and IEC/IRB will receive a blinded SUSAR summary, unless otherwise specified.

8.5.5. Pregnancy

All initial reports of pregnancy in participants or partners of male participants must be reported to the sponsor by the study site personnel within 24 hours of their knowledge of the event using the appropriate pregnancy notification form. Abnormal pregnancy outcomes (eg, spontaneous abortion, fetal death, stillbirth, congenital anomalies, ectopic pregnancy) are considered SAEs and must be reported using an SAE reporting form.

If a participant becomes pregnant during the study, follow-up information regarding the outcome of the pregnancy will be required. If the partner of a male participant becomes pregnant during the study, follow-up information regarding the outcome of the pregnancy will be requested upon the consent provided by the partner.

8.5.6. Adverse Events of Special Interest

AESIs (including potential AESIs) are significant AEs that are judged to be of special interest because of clinical importance, known or suspected class effects, or based on nonclinical signals. AESIs and potential AESIs will be carefully monitored during the study by the sponsor. AESIs and potential AESIs must be reported to the sponsor within 24 hours of awareness irrespective regardless of seriousness (ie, serious and nonserious AEs) or causality following the procedure described above for SAEs.

AESIs must be reported using the AESI form in the eCRF using the eCRF completion guidelines.

Specific requirements for the AESI are described below.

8.5.6.1. Thrombosis with Thrombocytopenia Syndrome

TTS is a syndrome characterized by a combination of both a thrombotic event and thrombocytopenia. (American Society of Hematology 2021, Brighton Collaboration 2021). As described in Section 2.2, vaccine-induced TTS has been observed very rarely following vaccination with the Janssen COVID vaccine.

TTS is considered to be an AESI in this study. Because this syndrome is very rare and not completely understood, all cases of thrombosis and/or thrombocytopenia should be reported to the sponsor within 24 hours of awareness using the AESI form. Each case will be reviewed to identify a vaccine-induced TTS case. A potential TTS case is defined as:

- Thrombotic events: suspected deep vessel venous or arterial thrombotic events as detailed in Section 10.14, Appendix 14,

  and/or

- Thrombocytopenia, defined as platelet count below LLN for the testing lab

Responsibilities and procedures for identification, review, and evaluation of potential TTS cases are outlined in a separate Thrombosis with Thrombocytopenia Syndrome Adjudication Committee Charter.

Symptoms, signs, or conditions suggestive of a thrombotic event or thrombocytopenia should be recorded and reported as a potential AESI even if the final or definitive diagnosis has not yet been determined, and alternative diagnoses have not yet been eliminated or shown to be less likely. Follow-up information and final diagnoses, if applicable, should be submitted to the sponsor as soon as they become available.

In the event of thrombocytopenia, study site personnel should report the absolute value for the platelet count and the reference range for the laboratory test used.

For either a thrombotic event or thrombocytopenia, complete blood count including platelet count and a coagulation profile available from participant's medical records should be entered in the eCRF. In case these results are not available at the time of the event report in the eCRF, the study site is recommended to obtain a complete blood count including platelet count and a coagulation profile (to be performed at the local laboratory). Repeat testing at the local laboratory may be requested for confirmation upon sponsor discretion.

Aliquots of serum samples collected for immunogenicity test can be reconverted for participant's safety purposes upon sponsor request (Table 4).

Table 4 provides non-exhaustive list(s) of laboratory tests that may be performed (upon sponsor request).

In addition, the sponsor may request additional laboratory tests on additional blood samples obtained as soon as possible after the potential AESI onset, either during an ad hoc unscheduled visit or the next scheduled visit, whichever comes first. Table 5 provides a non-exhaustive list of laboratory tests that may be requested by the sponsor in case of potential AESI reporting, for which additional samples may be needed.

Table 4: Laboratory Tests That May Be Performed Upon Sponsor Request on Immunogenicity Samples Collected on Day 1 and 15 After Potential AESI Reporting

Parameters	Timepoints
• Serum samples for assay such as but not limited to:<br>o Heparin Induced Thrombocytopenia (HIT)/PF4 Ab, IgG·(HIT assay)<br>If the above test is positive, also consider:<br>– Anti-cardiolipin antibody<br>– Beta-2 glycoprotein	• Days 1 and 15 visits (aliquots of serum samples collected for immunogenicity test can be reconverted for participant's safety purposes).

Note: results of the test should be reported in the narrative of the event and/or in the TTS AESI pages of the eCRF.

Table 5: Laboratory Tests That May Be Requested by the Sponsor to be Performed at the Central Laboratory After Potential AESI Reporting

Parameters	Timepoints
• Serum/plasma/whole blood samples for coagulation-related assays such as but not limited to:<br>o Fibrinogen<br>o D-dimer·<br>o Lupus anticoagulant·<br>o Anti-cardiolipin antibody<br>o Beta-2 glycoprotein·<br>o Heparin Induced Thrombocytopenia (HIT)/PF4 Ab, IgG·(HIT assay)<br>o Platelet activation assay (if HIT/PF4 is positive)<br>o Homocysteine·<br>o COVID-19 serological test	• As soon as possible after the potential AESI onset upon sponsor request (during an ad hoc unscheduled visit or the next scheduled visit, whichever comes first).

Note: results of the test should be reported in the narrative of the event and/or in the TTS AESI pages of the eCRF. Irrespective on samples for central laboratory tests collection, relevant data for TTS assessment reported in the medical records of the participant should be reported in eCRF narrative of the event and/or in the TTS AESI pages of the eCRF.

AESIs, including potential AESIs, will require enhanced data collection and evaluation. Every effort should be made to report as much information as possible about the event to the sponsor in a reasonable timeframe. Relevant laboratory results can be entered on the AESI form in the eCRF, using the eCRF completion guidelines.

If the investigator is not the treating physician, every effort should be made to collect the information requested in the form from the treating physician and enter the available information in the eCRF.

If an event meets the criteria for an SAE (Section 10.3.1), it must be reported using the same process as for other SAEs.

Treatment and Follow-up Recommendation

The medical management of thrombotic events with thrombocytopenia is different from the management of isolated thromboembolic diseases. Study site personnel and/or treating physicians should follow available guidelines for treatment of thrombotic thrombocytopenia (eg, American Society of Hematology 2021, British Society for Haematology 2021, CDC 2021). The use of heparin may be harmful and alternative treatments may be needed. Consultation with a hematologist is strongly recommended.

8.6. Medical Resource Utilization and Health Economics

MRU data associated with medical encounters will be collected by the investigator and study site personnel at 28 days after onset of each ARI episode (ARI Day 29 ±7 days) for all ARIs that are followed until their resolution (ie, for RSV, hMPV, or influenza-positive ARIs, for ARIs with sputum collection, and for ARIs where at least one nasal swab cannot be tested at the site with the BioFire Filmarray Respiratory panel). Protocol-mandated procedures, tests, and encounters are excluded. The data collected may be used to conduct exploratory economic analyses and will include:

- Number and type of medical consultations (including primary care and specialist visits), whether these consultations were related to ARIs and a reason for each medical encounter
- Number and duration of hospital and/or institutional care admissions (total days length of stay, including duration by wards; eg, intensive care unit), whether these admissions were related to ARIs, and a reason for each admission
- Number and type of emergency department visits and whether these emergency department visits were related to ARIs
- Where applicable, information on whether supplemental oxygen or mechanical ventilation was used
- Whether participation in the study (ie, contact with healthcare providers during study visits and procedures) increased or decreased the participant's use of medical resources outside of the study

8.7. Participant Medical Information Prior to, During and After the Study (Real-world Data)

For participants (from the US only) who have provided consent for this, medical data (electronic health records, claims and laboratory data from other care settings) from 5 years prior to study enrollment until 5 years after study completion may be accessed utilizing tokenization and matching procedures (ie, the generation of anonymous identifiers or “tokens” [hashed and encrypted combinations of identifying elements] to allow linking of participant data from different sources without compromising the participant’s confidentiality). These data may be used for exploratory analyses to enhance our understanding of the impact of prior medical history on the response to immunization and the impact of immunization on efficacy and duration of efficacy as well as adverse events that may occur during and after completion of the study. The analyses will be described in detail in a dedicated analysis plan.

9. STATISTICAL CONSIDERATIONS

Statistical analysis will be done by the sponsor or under the authority of the sponsor. A general description of the statistical methods to be used to analyze the efficacy, safety and immunogenicity data is outlined below. Specific details will be provided in the Statistical Analysis Plan. This section focusses on the key statistical considerations for the Global cohort. Analyses for local cohort enrollment will be described separately, but local cohorts will also be included in the primary analysis and final analysis if their enrollment started prior to the database cut-off of the primary analysis or final analysis, respectively.

9.1. Statistical Hypotheses

The study is designed to test the primary hypothesis of VE against RT-PCR-confirmed RSV-mediated LRTD in the PPE population.

- The hypothesis for the primary endpoint is:

  Null hypothesis: the VE against RT-PCR-confirmed RSV-mediated LRTD of Group 1 vs placebo is ≤20%.

  Alternative hypothesis: the VE against RT-PCR-confirmed RSV-mediated LRTD of Group 1 vs placebo is >20%.

Refer to Section 9.3 for the definition of the PPE population and to Section 9.4.1.1 for details on the primary endpoint. The analysis plan for the interim and primary analyses is provided in Section 9.5.1.

The study is successful if:

- The LL of the 95% 2-sided confidence interval (CI, potentially corrected as described in Section 9.4.1.3) around the VE (1-relative risk rate) calculated from the Exact Poisson regression model is above 20% and additionally a point-estimate of VE >50% is observed for Group 1

If VE for the primary endpoint is demonstrated, the following secondary endpoints will be tested:

1. First occurrence of any RT-PCR-confirmed RSV ARI, with onset at least 14 days after dosing of study vaccine
2. First occurrence of any RT-PCR-confirmed RSV-mediated LRTD during the second year, with onset after study Day 365
3. First occurrence of any RT-PCR-confirmed RSV ARI during the second year, with onset after study Day 365
4. First occurrence of predefined clinically relevant disease associated with RT-PCR-confirmed RSV-mediated ARI over the whole study, with onset at least 14 days after dosing of study vaccine

The following null and alternative hypothesis will be used for those secondary endpoints:

- The null hypothesis is that the VE for the considered secondary endpoint is ≤0%.
- The alternative hypothesis is that the VE for the considered secondary endpoint is >0%.

Formal inference for secondary endpoint #1, as part of the testing strategy, will occur at the time of the primary analysis. For secondary endpoints 2, 3, and 4, the formal inference as part of the testing strategy will only occur at study end. For more details on the secondary endpoints and the testing strategy, see Section 9.4.1.2 and Section 9.4.1.3.

9.2. Sample Size Determination

9.2.1. Efficacy

The required sample size was determined using the following assumptions:

- a VE for RT-PCR-confirmed RSV-mediated LRTD of 65% during the first year and during the second year,
- an incidence of RT-PCR-confirmed RSV-mediated LRTD of at least 0.2% during the first RSV season (2021-2022) and an incidence of 0.5% in later RSV seasons in 60^{+}-year-old placebo recipients,
- a randomization ratio of 2:1 (active vaccine: placebo),
- an analysis plan as described in Section 9.5.1
- a 1-sided α of 2.5% (potentially corrected for multiplicity), and
- 10% of exclusions (due to dropout, major protocol violations, etc.) per year

Simulations performed in R show that with ~15,340 participants in the active vaccine group (Group 1) and ~7,670 participants in the placebo group (Group 2) the study has at least 90% total power to demonstrate VE against the primary endpoint statistically significant >20% and a point-estimate for VE >50%. This leads to a sample size of ~23,000 participants.

This sample size is also required to reach approximately 80% power for the secondary endpoint of first occurrence of RT-PCR-confirmed RSV-mediated LRTD during the second year.

Operating characteristics of the methods used with the sample size will be described in a separate modeling and simulations report.

In the proof-of-concept study (VAC18193RSV2001), which was performed in participants of at least 65 years of age, the observed VE for RSV-mediated LRTD (Case Definition 1) was 80% with corresponding 94.211% CI (52.2%; 92.2%). To account for uncertainty, a VE of 65% is used for the sample size determination. Further, an incidence of 1.1% for the primary endpoint selected for Phase 3 (Case Definition 1) was observed in study VAC18193RSV2001. The assumed incidence for the present study is decreased to 0.5% during the second RSV season to account for seasonal variability (Belongia 2018), and the potential impact of social distancing measures (anti-COVID) on the RSV incidence. As the impact of the anti-COVID measures on the first RSV season is unknown, the study covers for an incidence of RSV-mediated LRTD during the first RSV season of at least 0.2%.

Additional local cohort enrollment beyond the Global cohort enrollment, may be allowed if required by local health authorities for the purpose of local regulatory approval consideration. Any such local cohort enrollment, not part of the Global cohort, will be analyzed as a separate dataset apart from the Global cohort, but they will also be included in the primary analysis and final analysis if their enrollment started prior to the database cut-off of the primary analysis or final analysis, respectively.

9.2.2. Immunogenicity

Immunogenicity will be assessed in the Immuno Subset, including ~360 participants in Europe and North America, of whom ~240 are active study vaccine participants. Additionally, immunogenicity may be assessed in ~120 from each of the other regions in the setting that these are part of the study. Approximately 50% of the participants in the Immuno Subset in each region will be at increased risk of severe RSV disease and ~50% will be 75 years or older. If further local immunogenicity data are needed per country/territory specific guidelines, this will be described in a separate document.

Table 6 shows the actual distance from the mean to the limits of the 95% CI around the actual value at Day 15 for the different assays, accounting for ~10% of discontinuations.

Table 6: Distance from the Mean to the Limits of 95% CI Around the Actual Value at Day 15 for Pre-F ELISA, VNA, and ELISpot

		Pre-F ELISA (SD=1.3)		VNA (SD=1.5)		ELISpot (SD=1.2)	
	N (Active Group)*	Distance[a] from Mean to Limits 95% CI	95% CI if Observed Mean is eg, 6,000	Distance[a] from Mean to Limits 95% CI	95% CI if Observed Mean is eg, 6,500	Distance[a] from Mean to Limits 95% CI	95% CI if Observed Mean is eg, 400
All	240	0,174	(5318;6769)	0,201	(5655;7472)	0,161	(358;447)
Increased Risk	120	0,248	(5052;7125)	0,286	(5331; 7925)	0,229	(341;469)

* Not yet including Southern Hemisphere and eventual other local 89ucces data.

CI=confidence interval, ELISA=enzyme-linked immunosorbent assay, ELISpot=enzyme-linked immunospot, SD=standard deviation, VNA=virus neutralization assay

[a] Calculated on the log_2-scale.

9.2.3. Safety

While mild to moderate reactogenicity (local injection site and systemic reactions) are expected, AEs that preclude further vaccine administration (if applicable) are not anticipated.

Solicited and unsolicited AEs will be captured in the Safety Subset, including ~1,050 participants from Europe and North America (~700 active and ~350 placebo participants). Additionally, solicited and unsolicited AEs will be captured in ~300 participants from each of the other regions in the setting that these are part of the study. Approximately 50% of all participants in the Safety Subset in each region will be at increased risk of severe RSV disease and ~50% of all participants in the Safety Subset in each region will be 75 years or older.

If further local solicited and unsolicited AE data are needed per country/territory specific guidelines, this will be described in separate documentation.

SAEs/AESIs will be captured in all participants. Outside the Safety Subset and Immuno Subset, no cap is installed on the number of participants at increased risk of severe RSV disease. It is expected that participants at increased risk of severe RSV disease will represent ~25% of the overall population, so it is estimated that SAE/AESI information will be available for ~3,835 participants at increased risk of severe RSV disease in the active vaccine group (Group 1).

For SAEs/AESIs, the observation of 0 events in the database would be associated with 95% confidence that the true rate is less than 0.025%. Assuming that ~3,835 participants in the total active vaccine group (Group 1) are at increased risk of severe RSV disease, the observation of 0 SAEs/AESIs in participants at increased risk of severe RSV disease would be associated with 95% confidence that the true rate is less than 0.1%.

For unsolicited AEs, which are captured in the Safety Subset of at least 700 active participants from Europe and North-America, the observation of 0 unsolicited AEs in the active group would be associated with 95% confidence that the true rate is less than 0.5%. In active participants at increased risk of severe RSV disease (~350 active participants from Europe and North-America), this would be less than 1%.

Table 7 shows the probabilities of observing at least one event (solicited, unsolicited or serious AE) in one of the arms at given true AE rates (overall population and participants at increased risk of severe RSV disease).

Table 7: Probability of Observing at Least One Adverse Event or Serious Adverse Event at a Given True Adverse Event Rate in the Active Vaccine Group (Group 1)

True Adverse	Probability of Observing at Least One Adverse Event in N Participants			
	Overall		Increased Risk	
Event Rate	Solicited and Unsolicited Adverse Events N=700[a]	Serious Adverse Events in Group 1 N=15,340	Solicited and Unsolicited Adverse Events N=350[a]	Serious Adverse Events in Group 1 N=3,835[b]
0.01%	7%	78%	3%	32%
0.1%	50%	100%	30%	98%
0.5%	97%	100%	83%	100%
1%	100%	100%	97%	100%
2.5%	100%	100%	100%	100%

9.3. Populations for Analyses

Vaccination assignment will follow the as-treated principle. Note that all analyses will focus on the participants enrolled in the Global cohort. Any local cohort enrolment beyond what is included in the Global cohort will be analyzed as a separate dataset apart from the Global cohort, but they will also be included in the primary analysis and final analysis if their enrollment started prior to the database cut-off of the primary analysis or final analysis, respectively.

For purposes of analysis, the following populations are defined:

- Full Analysis Set (FAS): will include all participants with a documented vaccine administration, regardless of the occurrence of protocol deviations. The FAS is the primary safety population. The analysis of solicited and unsolicited AEs will be restricted to a subset of the FAS (ie, the Safety Subset).[a]
- Per-protocol Efficacy (PPE) population includes all randomized and vaccinated participants, excluding those with major protocol deviations (MPDs) expecting to impact the efficacy outcomes. Only data of the year (first year, second year) in which the MPD impacting efficacy occurs and the data of later years are excluded. The list of MPDs to be excluded from efficacy analyses will be specified in the Statistical Analysis Plan or major protocol violation criteria document, which will be finalized before database lock and unblinding. Participants with an RT-PCR-confirmed RSV-mediated ARI with onset within 14 days after vaccination, participants who discontinue within 14 days after vaccination, and participants who received vaccination within 14 days of the database cut-off date will be excluded from the PPE

[a] The specification of this subset for analysis of solicited and unsolicited AEs does not preclude the investigator from reporting an AE in any participant if the investigator considers the event to be of clinical relevance and/or related to the study vaccine.

population. This analysis set will be used for analyses over the whole study period (up to database cut-off).

For analyses focusing on the first year, the PPE population is similar but only excludes MPDs occurring during the first year.

For analyses focusing on the second year, participants with MPDs expecting to impact the efficacy outcomes and occurring during the second year will be additionally excluded from the PPE set defined above, as well as participants who discontinued prior to Day 365.

The PPE population is the primary efficacy population. Sensitivity efficacy analyses will be performed on the FAS.

- Per-protocol Immunogenicity (PPI) population: will include all randomized and vaccinated participants who are part of the Immuno Subset and for whom immunogenicity data are available. Samples taken after a participant experiences an MPD expected to impact the immunogenicity outcomes will be excluded from the PPI analysis. The list of MPDs to be excluded from the immunogenicity analysis will be specified in the Statistical Analysis Plan or major protocol violation criteria document, which will be finalized before database lock and unblinding.

In addition, for participants who experience an RT-PCR-confirmed RSV-mediated ARI, samples taken after the RSV infection will not be taken into account in the assessment of the immunogenicity.

- The PPI population is the primary immunogenicity population. For key tables, sensitivity immunogenicity analyses will be performed on the FAS, including participants who are part of the Immuno Subset for whom immunogenicity measures are available. Excluded samples may be taken into account as well in the sensitivity analysis.

9.4. Statistical Analyses

The Statistical Analysis Plan will be finalized prior to the first database lock (primary or interim analysis) and it will include a more technical and detailed description of the statistical analyses described in this section. This section is a summary of the planned statistical analyses of the most important endpoints including primary and secondary endpoints.

The analysis plan for the interim and primary analyses is provided in Section 9.5.1.

9.4.1. Efficacy Analysis

9.4.1.1. Primary Endpoint

The analysis of the primary endpoint will evaluate the first occurrence of RT-PCR-confirmed RSV-mediated LRTD (for the definition, see Section 8.2, Efficacy Assessments) with onset of at least 14 days after vaccination in the active vaccine group compared to the placebo group in the PPE population. Time at risk will also be taken into account. The null hypothesis of LL ≤20% for RT-PCR-confirmed RSV-mediated LRTD will be tested versus the alternative hypothesis of LL >20%. Additionally, the observed VE should be >50%.

Exact Poisson regression will be fitted with the event rate, defined as the number of cases (with onset at least 14 days after vaccination) over the follow-up time (off-set) as dependent variable and the vaccination group, being at increased risk of RSV disease, and age stratum (both variables as stratified; see Section 6.3) as independent variables. Each participant will be taken into account in the model with one observation indicating whether they had a case or not, the vaccination group, the stratification factors, and the follow-up time.

The follow-up time is defined as the time between 14 days post-vaccination and occurrence of the first event. For non-cases, it is the time between 14 days post-vaccination and the database cut-off date of the analysis. However, for participants who discontinued or completed the study before that date and before having an event, follow-up time is defined as the time between 14 days post-vaccination and the date of last contact.

For participants with an MPD impacting efficacy during the second year, only the first year data are taken into account: for cases during the first year, the follow-up time is defined as the time between 14 days post-vaccination and occurrence of the first event. For non-cases, it is the time between 14 days post-vaccination and the Day 365 visit. Thus, all participants will be included in the analysis according to their follow-up time.

The study is 92uccessfull if:

- The LL of the 95% 2-sided CI (potentially corrected as described in Section 9.4.1.3) around the VE (1-relative risk rate) calculated from the Exact Poisson regression model is above 20% and additionally a point-estimate of VE >50% is observed for Group 1

Sensitivity Analyses

As a sensitivity analysis, the above model will be repeated based on the FAS, not taking into account the restriction on the onset (at least 14 days). Additional sensitivity analyses will include:

- An exact binomial test, which does not take into account strata or follow-up time, based on the PPE population and the FAS. The VE (1-relative risk) and the corresponding corrected 2-sided CI based on the exact binomial will be calculated as well. The same alpha as in the primary analysis will be used for calculating the CI.
- If the primary analysis is performed in 2023, an exact Poisson model similar to the primary analysis will be performed including an interaction term between the treatment group and the year variables.

The analysis of the primary endpoint will be repeated, but participants with RSV infection and a co-infection with at least one other respiratory virus (ie, confirmed using the BioFire Filmarray Respiratory panel for nasal swabs, or the BioFire Filmarray PN panel for a sputum sample, when available) will not be considered as a case for that episode, as the etiology of symptoms will be indeterminate. Additionally, the analysis of the primary endpoint will be repeated, taking into account only the RT-PCR test results from the study-specified BioFire Filmarray Respiratory panel for nasal swabs and the BioFire Filmarray PN panel for a sputum sample, when available (ie, excluding hospital test results with a locally approved RT-PCR test for participants who were

hospitalized). Details on the strategy for local site and central testing of the nasal swabs and sputum samples are provided in Section 8.2.4. Another sensitivity analysis will focus on the programmed case definition of RT-PCR confirmed RSV-mediated LRTD, not taking into account the CEC adjudication.

9.4.1.2. Secondary Efficacy Endpoints

The following endpoints are considered confirmatory secondary endpoints:

1. First occurrence of any RT-PCR-confirmed RSV ARI, with onset at least 14 days after dosing of study vaccine
2. First occurrence of any RT-PCR-confirmed RSV-mediated LRTD during the second year with onset after study Day 365
3. First occurrence of any RT-PCR-confirmed RSV ARI during the second year with onset after study Day 365
4. First occurrence of predefined clinically relevant disease associated with RT-PCR-confirmed RSV-mediated ARI over the whole study, with onset at least 14 days after dosing of study vaccine

A similar Exact Poisson regression model as for the primary endpoint will be used for the analyses of the secondary endpoints focusing on VE (secondary endpoints #1, #2, #3 and #4). For those secondary endpoints that focus on VE during the second year (secondary endpoints #2 and #3) the follow-up time is defined as follows: For cases occurring after Day 365, the follow-up time is defined as the time between Day 365 and the occurrence of the event. For non-cases, the follow-up time is the time between Day 365 and the minimum of the database cut-off date of the analysis and the completion or discontinuation date. Participants who discontinue prior to the Month 12/Day 365 visit or have a major protocol deviation impacting efficacy during the first year are excluded from this analysis.

For secondary endpoint #1 the follow-up time is defined similarly as for the primary endpoint.

The following non-confirmatory secondary endpoint will also be evaluated:

- The AUC of the change from baseline in RiiQ Total Symptom score in participants with an RT-PCR-confirmed RSV-mediated ARI over the whole study

Descriptive statistics will be calculated restricting to participants with RT-PCR-confirmed RSV-mediated ARI during the whole study.

A Wilcoxon Rank Sum test will be performed to test the null hypothesis that the distribution in both groups is the same versus the alternative hypothesis that the distribution shifted towards a lower AUC in the change from baseline in RiiQ Total Symptom score (ie, more reduced symptom scores). This is a non-confirmatory endpoint and will not be taken into account in the testing strategy, no alpha correction will be applied.

9.4.1.3. Testing Strategy for Primary and Confirmatory Secondary Endpoints

The VE against the primary endpoint will be tested using the significance level α' (α_1 or α_2 or α_3 or α_4), as defined in Section 9.5.1. If the LL of the exact 2-sided CI (1-2×α') for the VE (1-relative risk rate) calculated from the Exact Poisson regression model, is above 20% and the observed VE is above 50%, VE is demonstrated for the primary endpoint. If VE is demonstrated for the primary endpoint, the above secondary endpoints will be tested according to the testing order displayed in Table 8. The first secondary efficacy endpoint (first occurrence of any RT-PCR-confirmed RSV ARI) is tested using the same significance level α' as used for the primary endpoint. The Exact 2-sided CI (1-2×α') for the VE (1-relative risk rate) will be calculated from the Exact Poisson regression model. If the LL of the CI is above 0%, VE will have been demonstrated for this endpoint and the next endpoint in the testing strategy can be tested. The following secondary endpoints (#2, #3, and #4) will be tested hierarchically at the end of the study at the full alpha (α 2.5%), as indicated in the table below.

Table 8: Secondary Endpoints: Overview of the Testing Order, Timing of the Testing in the Testing Strategy and the Corresponding Alpha

Secondary endpoint: first occurrence of	*Analysis timepoint in the testing strategy*	*Alpha at which the endpoint is tested*
1. Any RT-PCR confirmed RSV ARI	Time of primary analysis	Alpha used at the primary analysis
2. Any RT-PCR confirmed RSV LRTD during the second Year	End of the study	2.5%
3. Any RT-PCR confirmed RSV ARI during the second Year	End of the study	2.5%
4. Predefined clinically relevant disease associated with RT-PCR-confirmed RSV-mediated ARI	End of the study	2.5%

Second year starts at the D365 visit

9.4.1.4. Exploratory Endpoints

For the following exploratory endpoints, similar Exact Poisson regression models as for the primary and secondary endpoints will be used and exact 2-sided 95% Cis for VE (1-relative risk rate) will be calculated. For all exploratory endpoints related to influenza-mediated ARI and LRTD only the proportion of participants meeting the respective endpoints will be summarized.

- First occurrence of RT-PCR-confirmed RSV-mediated LRTD during the first year
- First occurrence of RT-PCR-confirmed RSV-mediated ARI during the first year
- First occurrence of RT-PCR-confirmed RSV-mediated LRTD by subtype (RSV A or RSV B)
- First occurrence of RT-PCR-confirmed RSV-mediated ARIs by subtype (RSV A or RSV B)
- First occurrence of complications associated with RT-PCR-confirmed RSV-mediated ARIs RSV-mediated LRTD, influenza-mediated ARIs and hMPV-mediated ARIs
- First occurrence of hospitalizations associated with RT-PCR-confirmed RSV-mediated ARIs RSV-mediated LRTD, influenza-mediated ARIs and hMPV-mediated ARIs

- First occurrence of pneumonia (radiological or X-ray confirmed) associated with RT-PCR-confirmed RSV-mediated ARIs RSV-mediated LRTD, influenza-mediated ARIs and hMPV-mediated ARIs
- First occurrence of emerging therapeutic interventions of interest associated with RT-PCR-confirmed RSV ARIs. The following are considered emerging therapeutic interventions of interest:

  New onset or increased dose (compared to baseline) of bronchodilator/nebulizer treatment

  New onset or increased dose (compared to baseline) of corticosteroid prescription

  New onset or increased dose (compared to baseline) of antibiotic prescription

  New onset or increased dose (compared to baseline) of antiviral prescription
- First occurrence of RT-PCR-confirmed hMPV-mediated ARI
- First occurrence of RT-PCR-confirmed hMPV-mediated LRTD

Unless specifically mentioned otherwise, the Ves will be calculated for the above endpoints occurring during the first year, and during the second year and over the whole study. The follow-up time is determined in a similar way as for the primary and secondary endpoints.

If the primary analysis occurs before the end of the study, the secondary endpoints #2, #3 (provided second year data are already available) and #4 might be analyzed descriptively at the time of the primary analysis, VE with 95% CI will be calculated.

RSV A and RSV B viral load will be analyzed descriptively. More details on exploratory endpoints will be provided in the Statistical Analysis Plan.

Exploratory endpoints related to disease severity in participants with RT-PCR-confirmed RSV-mediated ARIs and in participants with RT-PCR-confirmed RSV-mediated LRTDs are:

The AUC of the change from baseline in RiiQ Total Symptom score*

* Note: Reduction of symptom severity in participants with an RT-PCR-confirmed RSV-mediated ARI over the whole study is a non-confirmatory secondary endpoint.

The time to return to usual health (based on this question from the PGI questionnaire).

The CEC review will also assess, independently and based on CRF/eDiary data, the severity of the RT-PCR-confirmed RSV-mediated LRTDs. The CEC will use all information available for the participants, including but not limited to MRU, presence of clinically relevant disease, use of therapeutic interventions, and RiiQs, in order to make their adjudication.

The following endpoints will be calculated with similar Exact Poisson regression models as for the primary and secondary endpoints:

- First occurrence of an RT-PCR-confirmed RSV LRTDs assessed as at least mild, as at least moderate and as at least severe by the CEC during the considered year:

with onset at least 14 days after dosing of study vaccine and prior to study Day 365 (first year comparison)

with onset after study Day 365 (second year comparison)

with onset at least 14 days after dosing of study vaccine (whole study comparison)

For more details on these endpoints, see Section 8.1. These endpoints may be determined during the first year, during the second year, and potentially over the whole study. For the AUC, descriptive statistics will be calculated once restricting to participants with a RT-PCR-confirmed RSV-mediated ARI in the considered period and once restricting to participants with RT-PCR-confirmed RSV-mediated LRTD in the considered period. Additionally, these analyses will be done by subtype (RSV A or RSV B).

For the time to return to usual health, a Kaplan Meier curve will be fitted and a hazard ratio with corresponding 95% 2-sided CI will be calculated from a Cox proportional hazard model with group as dependent variable. These analyses will be restricted once to participants with a RT-PCR-confirmed RSV-mediated ARI in the considered period and once restricted to participants with RT-PCR-confirmed RSV-mediated LRTD in the considered period. These analyses will be done by subtype (RSV A or RSV B) as well.

9.4.2. Immunogenicity Analyses

No formal statistical testing of the immunogenicity data is planned. All immunogenicity analyses will be performed on the PPI set. Key tables may be repeated for the FAS (including the samples that are excluded from the PPI analysis).

Immunogenicity Subset

No formal hypothesis on immunogenicity will be tested. Descriptive statistics (such as geometric mean and 95% CI for ELISA and RSV neutralization assay, and median and quartiles for interferon gamma [IFN-γ] enzyme-linked immunospot [ELISpot]) will be calculated for continuous immunogenicity parameters at all available timepoints. For the humoral assays, geometric mean fold rises from baseline with 95% Cis may additionally be calculated. Baseline is considered as the last available assessment before vaccination. Graphical representations of immunogenicity parameters will be made as applicable.

The impact of baseline factors on the humoral and cellular responses will be explored graphically or via descriptive statistics.

Correlates of Protection

If VE is demonstrated, correlates of protection will be further explored in samples collected from all participants. Data from the current study will be used to confirm and refine the model found in study VAC18193RSV2001. Immunogenicity markers are considered correlates of protection if VE is explained through the effect of the vaccine on the immunogenicity markers. More details with appropriate methods will be provided in a separate analysis plan.

9.4.3. Safety Analyses

No formal statistical testing of safety data is planned. Safety data by vaccination group and based on the FAS will be analyzed descriptively. The analysis of solicited and unsolicited AEs will be restricted to a subset of the FAS (ie, the Safety Subset). For the analyses of AESIs, SAEs, and COVID-related AEs, the entire FAS population will be used.

The impact of baseline factors (such as being at increased risk for severe RSV disease) may be explored as well.

Adverse Events (Solicited and Unsolicited)

Solicited local (at injection site) and systemic AEs will be summarized descriptively. Solicited AEs shown in the tables and listings will be based on the overall assessment of the investigator. The overall frequencies by vaccine group as well as the frequencies according to severity and duration will be described for solicited AEs. The number and percentages of participants with at least one solicited local (at the injection site) or systemic AE will be presented. Frequencies of unsolicited AEs, separately for all and vaccination-related only, will be shown by system organ class and preferred term, while those of solicited AEs will be presented only by preferred term.

The verbatim terms used in the eCRF by investigators to identify AEs will be coded using the Medical Dictionary for Regulatory Activities (MedDRA). Reported AEs with onset during the active vaccination phase (ie, AEs occurring post vaccination up to 28 days post-vaccination) and SAEs and AESIs will be included in the analysis. For each AE, the number and percentage of participants who experience at least one occurrence of the given event will be summarized by group.

Summaries, listings, datasets, or participant narratives may be provided, as appropriate, for those participants who die, who discontinue the study (or vaccination, if applicable) due to an AE, or who experience a severe AE or AESI or SAE or COVID-19 infection.

ARIs recorded as (S)AEs in the eCRF will be excluded from any (S)AE analysis if the laboratory RT-PCR is subsequently found to be positive for RSV. ARIs arising from RSV infection will not be reported as (S)AEs in the Clinical Study Report, as they are endpoints of the study and will be tabulated separately.

Vital Signs

A tabulation of the distribution of body temperatures in the 7 days following vaccination will be provided per half-degree intervals. Emerging vital sign abnormalities will be summarized based on the planned visits. Clinically relevant abnormalities for systolic and diastolic blood pressure, heart rate, respiratory rate, and oxygen saturation collected during unscheduled visits will be documented as AEs.

9.4.4. Other Analyses

9.4.4.1. Patient-reported Outcomes

Changes from baseline in daily symptom severity reported by participants using the RiiQ Symptom Scale and changes from baseline functioning associated with ARI collected using the RiiQ Impact Scales will be descriptively analyzed. The AUC of the change from baseline in Total Symptom Score will be determined. For the analysis of this AUC, refer to Section 9.4.1.2. For other scores, AUC may be calculated as well.

The Patient Global Impression Scales from ARI symptom onset to ARI symptom resolution will be descriptively analyzed by group (active vaccine or placebo). AUCs may be calculated. For the analysis of the time to return to usual health refer to Section 9.4.1.4.

EQ-5D-5L data will be analyzed descriptively by dimension (mobility, self-care, usual activities, pain/discomfort, and anxiety/depression), single index value, and EQ-VAS, and summarized by study vaccination group. Focus will be on participants with an RSV-confirmed ARI. This will be further detailed in a Statistical Analysis Plan.

9.4.4.2. Medical Resource Utilization Analyses

MRU data will be descriptively summarized by vaccination group. A VE of emerging MRU associated with an RT-PCR-confirmed RSV mediated ARI and of hMPV and influenza-mediated respiratory infections may be calculated as well. This will be further detailed in the SAP.

9.5. Planned Analyses

9.5.1. Analysis Plan for the Interim and Primary Efficacy Analyses

The following describes the analysis plan for the interim analysis and the primary analysis. This takes into account participants enrolled in the Global cohort. Any local cohort enrolment beyond the Global cohort will be analyzed separately, except if individual participant enrollment started before the database cut-off of the primary analysis.

The interim analysis plan is as follows:

- Based on a database cut-off date of 15 September 2022, an IA by IDMC will be performed. The IDMC will evaluate in an unblinded fashion if the LL of the (100-2×α_1)% 2-sided CI around the VE (1-relative risk rate) is above 20% and additionally a point-estimate of VE >50% is observed for Group 1. If the success criteria are met, the sponsor will be notified. The database used for the IDMC analysis will be unblinded for the sponsor and the primary analysis will be performed by the sponsor at the same significance level (α_1). If the success criteria are not met, the participants will be followed further, and the same steps will be performed at the following subsequent database cut-offs:
- 24 October 2022 with an alpha level α_2
- 22 January 2023 with an alpha level α_3

- Provided no primary analysis occurred earlier, the primary analysis will be performed with database cut-off 30 April 2023 with alpha level α_4

Figure 3 displays the interim and primary analysis plan.

If VE is not demonstrated to be significantly >20% with a point estimate >50% at the time of the primary analysis, the study will be stopped.

The total alpha will be controlled at 2.5%. A Pocock-like alpha spending function as described by DeMets (DeMets 1994) with a target number of events of 76 used to spend the alpha over the different analyses. The different alphas to be used during every IA are shown above in Figure 3 and the calculations are explained below in Table 9.

Table 9: Alpha-spending at the Prespecified Analysis Timepoints

Alpha	*Description of how to obtain alpha*
α_1	Alpha obtained from Pocock-like alpha spending function with information fractions = [# RSV LRTD events by 15 September 2022 / 76[¥], 1]
α_2	Alpha obtained from Pocock-like alpha spending function with information fractions = [# RSV LRTD events by 15 September 2022 / 76[¥], # RSV LRTD events by 24 October 2022 / 76[¥], 1]
α_3	Alpha obtained from Pocock-like alpha spending function with information fractions = [# RSV LRTD events by 15 September 2022 / 76[¥], # RSV LRTD events by 24 October 2022 / 76[¥], # RSV LRTD events by 22 Jan 2023 / 76[¥], 1]
α_4	Alpha obtained from the remaining alpha to be spent (2.5% - total alpha spent during the interim analyses) and the actual information fractions observed at all analyses. That is, the information fraction, IF = [# RSV LRTD events by 15 September 2022 / # RSV LRTD events by 30 April 2023, # RSV LRTD events by 24 October 2022 / # RSV LRTD events by 30 April 2023, # RSV LRTD events by 22 January 2023 / # RSV LRTD events by 30 April 2023, 1]. The final alpha will be calculated using the user-defined alpha spending function approach (Jennison 2000).

¥ 76 target sample size for 90% power with 65% VE. Note that if at any of the interim analyses 76 or more RSV LRTD events have been observed, all remaining alpha will be spent and no further (interim) analyses for primary efficacy endpoint will be performed.

At the time of the primary analysis, unblinded safety and efficacy data will be analyzed. Immunogenicity data will be included if available.

Figure 3: Interim and Primary Analysis Plan

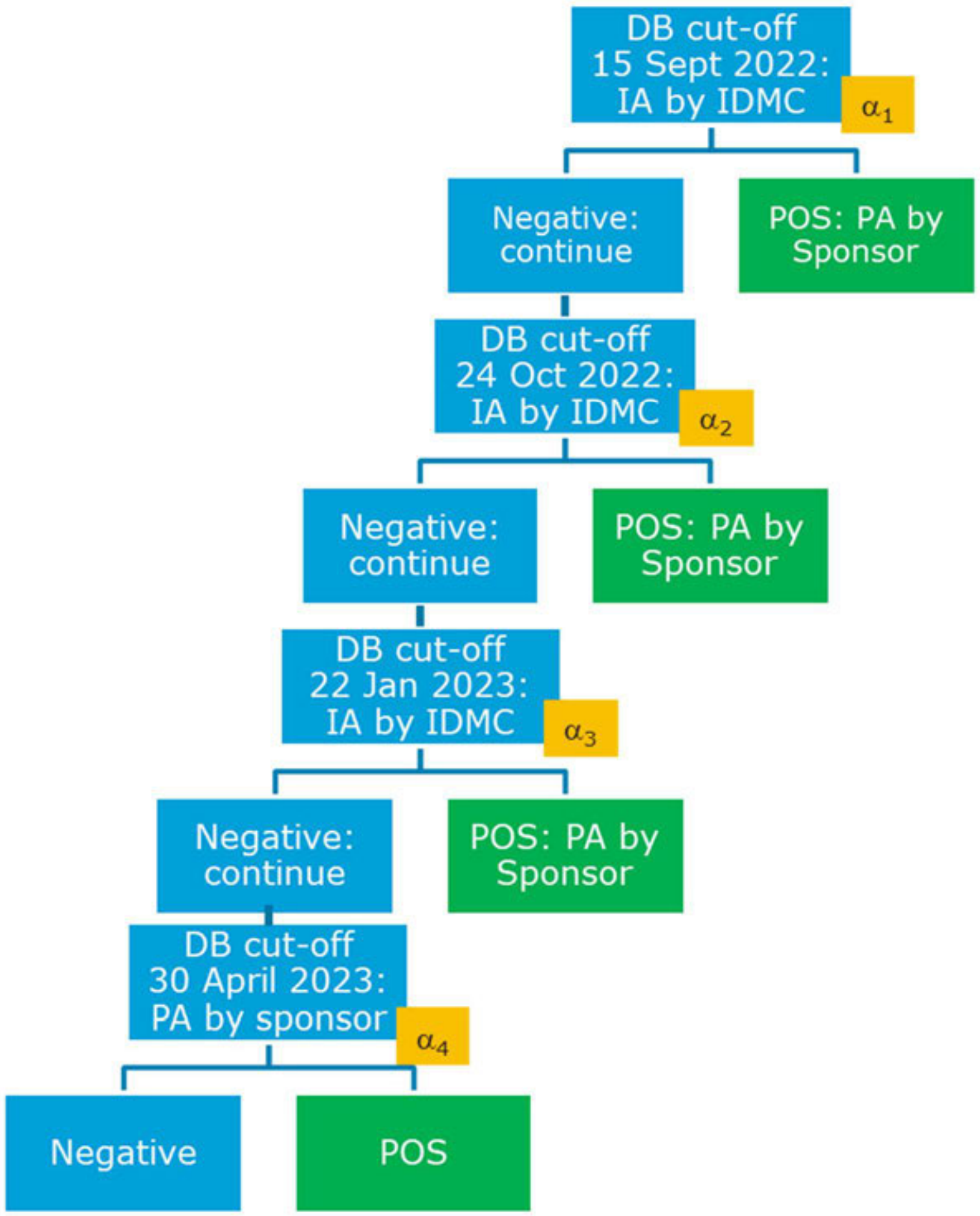

For the first interim analysis, ARIs with an onset up to 1 September 2022 will be included and a database cut-off of 15 September 2022 will be used. For the second IA, ARIs with an onset up to 10 October 2022 will be included and a database cut-off of 24 October 2022 will be used. For the third IA, ARIs with an onset up to 8 January 2023 will be included and a database cut-off of 22 January 2023 will be used. For the primary analysis, ARIs with an onset up to 15 April 2023 will be included and a database cut-off of 30 April 2023 will be used.
DB= database, IA= interim analysis, IDMC = independent data monitoring committee, PA=primary analysis, PPE=Per-protocol Efficacy, POS= positive

9.5.2. Additional Analyses

Provided the primary efficacy analysis has occurred earlier, the following exploratory analyses might be performed depending on program needs:

- **Exploratory analysis with database cut-off of 31 January 2023**: will include unblinded efficacy data, including secondary endpoints #2, #3 and #4. The latter will be considered an exploratory descriptive analysis. Formal inference of those endpoints as part of the testing strategy will only occur at study end. Safety data will be included when needed. Immunogenicity data will be included if available.
- **Exploratory analysis with database cut-off of 31 May 2023**: will include unblinded efficacy data, including secondary endpoints #2, #3 and #4. The latter will be considered an exploratory descriptive analysis. Formal inference of those endpoints as part of the testing strategy will only occur at study end. Safety data will be included when needed. Immunogenicity data will be included if available.

Note: Analyses will include all participants enrolled prior to the primary analysis and participants of local cohorts enrolled after the cut off of the primary analysis, provided that the local primary analysis has already been conducted.

9.5.3. End-of-Study Analysis

The end of study analysis will include unblinded efficacy, safety and immunogenicity data. For secondary endpoints #2, #3 and #4, formal inference as part of the testing strategy will occur during this analysis.

Note:

The timing of the end-of-study analysis is based on last participant last visit of the Global cohort but will include all participants of Global and local cohorts up to the cut-off of the end of study analysis (see Section 4.1, Number of Participants, for cohort definitions).

9.6. Committees

9.6.1. Independent Data Monitoring Committee

An IDMC will be established as noted under Committees Structure in Appendix 2: Regulatory, Ethical, and Study Oversight Considerations.

The study will be formally monitored by the IDMC. In general, the IDMC will monitor the safety data on a regular basis to ensure the continuing safety of the participants. Enrollment will not be paused during these safety reviews. The IDMC will review unblinded data. Conclusions from the IDMC reviews will be communicated to the sponsor.

In addition, the IDMC will also formally monitor the primary efficacy endpoint at the timepoints specified in Section 9.5, Planned Analyses. The IDMC will evaluate in an unblinded fashion if the success criteria have been met.

The IDMC will assess the null hypothesis of LL ≤20% for VE against RT-PCR-confirmed RSV-mediated LRTD versus the alternative hypothesis of LL >20%. For VE to be demonstrated for this primary endpoint additionally, the observed VE should be >50%. Refer to Section 9.1 for details on the hypothesis testing.

To maintain the blinding of the study to the clinical staff, investigators, and sponsor personnel, the independent statistical support group will perform the IDMC safety analyses and will present the results to the IDMC. A sponsor committee will be established for this study, which will be the point of contact between IDMC and sponsor. After the review, the IDMC will make recommendations regarding the continuation of the study.

The IDMC responsibilities, authorities, and procedures will be provided in its charter.

9.6.2. Clinical Event Adjudication Committee

Prior to database lock for all planned analyses (see Section 9.5), the CEC will review in a blinded manner all available data of all RSV-mediated ARIs and will confirm if the RT-PCR-confirmed RSV LRTD definition criteria were met. Further for all RT-PCR-confirmed RSV LRTD cases the CEC will assess their severity (mild, moderate, severe or unable to assess). Details on the review approach will be provided in the CEC Charter.

10. SUPPORTING DOCUMENTATION AND OPERATIONAL CONSIDERATIONS

10.1. Appendix 1: Abbreviations

Ad26	adenovirus serotype 26
ADCC	antibody dependent cell mediated cytotoxicity
ADCP	antibody dependent cellular phagocytosis
AE	adverse event
AESI	adverse event of special interest
ARI	acute respiratory infection
AUC	area under the curve
CEC	Clinical Event Adjudication Committee
CHF	congestive heart failure
CI	confidence interval
COPD	chronic obstructive pulmonary disease
COVID-19	coronavirus disease 2019
CVST	cerebral venous sinus thrombosis
CRF	case report form(s) (paper or electronic as appropriate for this study)
DNA	deoxyribonucleic acid
eCRF	electronic case report form
eDC	electronic data capture
ELISA	enzyme-linked immunosorbent assay
ELISpot	enzyme-linked immunospot (assay)
ePRO	electronic patient-reported outcome
EQ-5D-5L	EuroQoL, 5-Dimension, 5-Level (questionnaire)
EQ-VAS	EQ visual analogue scale
F protein	fusion protein
FAS	Full Analysis Set
FDA	(US) Food and Drug Administration
FOIA	Freedom of Information Act
GCP	Good Clinical Practice
GMT	geometric mean titer
HITT	heparin-induced thrombocytopenia and thrombosis
HIV	human immunodeficiency virus
hMPV	human metapneumovirus
HRQoL	health-related quality of life
IA	interim analysis
ICF	informed consent form
ICH	International Council for Harmonisation of Technical Requirements for Pharmaceuticals for Human Use
IDMC	Independent Data Monitoring Committee
IEC	Independent Ethics Committee
IF	information fraction
IFN-γ	interferon gamma
Ig	immunoglobin
IL	interleukin
IM	intramuscularly
IRB	Institutional Review Board
IWRS	interactive web response system
LL	lower limit
LRTD	lower respiratory tract disease
LRTI	lower respiratory tract infection
MAAE	medically-attended adverse event
mAbs	monoclonal antibodies
MedDRA	Medical Dictionary for Regulatory Activities
MPD	major protocol deviation
MRU	medical resource utilization
NH	Northern Hemisphere

PBMC	peripheral blood mononuclear cell
NSAIDs	non-steroidal anti-inflammatory drugs
PGI-C	Patient Global Impression of Change
PGI-H	Patient Global Impression of Health
PGI-S	Patient Global Impression of Severity
PPE	Per-protocol Efficacy
PPI	Per-protocol Immunogenicity
PQC	Product Quality Complaint
preF	pre-fusion conformation-stabilized F protein
PT	preferred term
RiiQ™	Respiratory Infection Intensity and Impact Questionnaire (Version 2)
RNA	ribonucleic acid
RSV	respiratory syncytial virus
RT-PCR	reverse transcriptase polymerase chain reaction
SAE	serious adverse event
SAM	synthetic absorptive matrix
SARS-CoV-2	severe acute respiratory syndrome coronavirus 2
SH	Southern Hemisphere
SUSAR	suspected unexpected serious adverse reaction
TNF-α	tumor necrosis factor alpha
TTS	thrombosis with thrombocytopenia syndrome
URTI	upper respiratory tract infection
US	United States
VE	vaccine efficacy
VITT	vaccine-induced immune thrombotic thrombocytopenia
VNA	virus neutralizing antibody
vp	viral particles

10.2. Appendix 2: Regulatory, Ethical, and Study Oversight Considerations

10.2.1. Regulatory and Ethical Considerations

Investigator Responsibilities

The investigator is responsible for ensuring that the study is performed in accordance with the protocol, the current International Council for Harmonisation (ICH) guidelines on Good Clinical Practice (GCP), and applicable regulatory and country/territory-specific requirements.

Good Clinical Practice is an international ethical and scientific quality standard for designing, conducting, recording, and reporting studies that involve the participation of human participants. Compliance with this standard provides public assurance that the rights, safety, and well-being of study participants are protected, consistent with the principles that originated in the Declaration of Helsinki, and that the study data are credible.

Protocol Clarification Communications

If text within a final approved protocol requires clarification (eg, current wording is unclear or ambiguous) that does not change any aspect of the current study conduct, a protocol clarification communication (PCC) may be prepared. The PCC Document will be communicated to the Investigational Site, Site Monitors, Local Trial Managers (LTMs), Clinical Trial Managers (CTMs), and/or Contract Research Organizations (CROs) who will ensure that the PCC explanations are followed by the investigators.

The PCC Document may be shared by the sites with Independent Ethics Committees/Institutional Review Boards (IECs/IRBs) per local regulations.

The PCC Documents must NOT be used in place of protocol amendments, but the content of the PCC Document must be included in any future protocol amendments.

Protocol Amendments

Neither the investigator nor the sponsor will modify this protocol without a formal amendment by the sponsor. All protocol amendments must be issued by the sponsor, and signed and dated by the investigator. Protocol amendments must not be implemented without prior IEC/IRB approval, or when the relevant competent authority has raised any grounds for non-acceptance, except when necessary to eliminate immediate hazards to the participants, in which case the amendment must be promptly submitted to the IEC/IRB and relevant competent authority. Documentation of amendment approval by the investigator and IEC/IRB must be provided to the sponsor. When the change(s) involve only logistic or administrative aspects of the study, the IEC/IRB (where required) only needs to be notified.

During the study, in situations where a departure from the protocol is unavoidable, the investigator or other physician in attendance will contact the appropriate sponsor representative listed in the Contact Information page(s), which will be provided as a separate document. Except in emergency situations, this contact must be made before implementing any departure from the protocol. In all cases, contact with the sponsor must be made as soon as possible to discuss the situation and agree

on an appropriate course of action. The data recorded in the eCRF and source documents will reflect any departure from the protocol, and the source documents will describe this departure and the circumstances requiring it.

Regulatory Approval/Notification

This protocol and any amendment(s) must be submitted to the appropriate regulatory authorities in each respective country/territory, if applicable. A study may not be initiated until all local regulatory requirements are met.

Required Prestudy Documentation

The following documents must be provided to the sponsor before shipment of study vaccine to the study site:

- Protocol and amendment(s), if any, signed and dated by the principal investigator
- A copy of the dated and signed (or sealed, where appropriate per local regulations), written IEC/IRB approval of the protocol, amendments, ICF, any recruiting materials, and if applicable, participant compensation programs. This approval must clearly identify the specific protocol by title and number and must be signed (or sealed, where appropriate per local regulations) by the chairman or authorized designee.
- Name and address of the IEC/IRB, including a current list of the IEC/IRB members and their function, with a statement that it is organized and operates according to GCP and the applicable laws and regulations. If accompanied by a letter of explanation, or equivalent, from the IEC/IRB, a general statement may be substituted for this list. If an investigator or a member of the study site personnel is a member of the IEC/IRB, documentation must be obtained to state that this person did not participate in the deliberations or in the vote/opinion of the study.
- Regulatory authority approval or notification, if applicable
- Signed and dated statement of investigator (eg, Form FDA 1572), if applicable
- Documentation of investigator qualifications (eg, curriculum vitae)
- Completed investigator financial disclosure form from the principal investigator, where required
- Signed and dated clinical trial agreement, which includes the financial agreement
- Any other documentation required by local regulations

The following documents must be provided to the sponsor before enrollment of the first participant:

- Completed investigator financial disclosure forms from all sub-investigators
- Documentation of sub-investigator qualifications (eg, curriculum vitae)
- Name and address of any local laboratory conducting tests for the study, and a dated copy of current laboratory normal ranges for these tests, if applicable

- Local laboratory documentation demonstrating competence and test reliability (eg, accreditation/license), if applicable

Independent Ethics Committee or Institutional Review Board

Before the start of the study, the investigator (or sponsor where required) will provide the IEC/IRB with current and complete copies of the following documents (as required by local regulations):

- Final protocol and, if applicable, amendments
- Sponsor-approved ICF (and any other written materials to be provided to the participants)
- Investigator Brochures (or equivalent information) and amendments/addenda
- Sponsor-approved participant recruiting materials
- Information on compensation for study-related injuries or payment to participants for participation in the study, if applicable
- Investigator's curriculum vitae or equivalent information (unless not required, as documented by the IEC/IRB)
- Information regarding funding, name of the sponsor, institutional affiliations, other potential conflicts of interest, and incentives for participants
- Any other documents that the IEC/IRB requests to fulfill its obligation

This study will be undertaken only after the IEC/IRB has given full approval of the final protocol, amendments (if any, excluding the ones that are purely administrative, with no consequences for participants, data or study conduct, unless required locally), the ICF, applicable recruiting materials, and participant compensation programs, and the sponsor has received a copy of this approval. This approval letter must be dated and must clearly identify the IEC/IRB and the documents being approved.

During the study the investigator (or sponsor where required) will send the following documents and updates to the IEC/IRB for their review and approval, where appropriate:

- Protocol amendments (excluding the ones that are purely administrative, with no consequences for participants, data or study conduct)
- Revision(s) to ICF and any other written materials to be provided to participants
- If applicable, new or revised participant recruiting materials approved by the sponsor
- Revisions to compensation for study-related injuries or payment to participants for participation in the study, if applicable
- New edition(s) of the Investigator Brochures and amendments/addenda
- Summaries of the status of the study at intervals stipulated in guidelines of the IEC/IRB (at least annually)
- Reports of AEs that are serious, unlisted/unexpected, and associated with the study vaccine

- New information that may adversely affect the safety of the participants or the conduct of the study
- Deviations from or changes to the protocol to eliminate immediate hazards to the participants
- Report of deaths of participants under the investigator's care
- Notification if a new investigator is responsible for the study at the site
- Development Safety Update Report and Line Listings, where applicable
- Any other requirements of the IEC/IRB

For all protocol amendments (excluding the ones that are purely administrative, with no consequences for participants, data or study conduct), the amendment and applicable ICF revisions must be submitted promptly to the IEC/IRB for review and approval before implementation of the change(s).

At least once a year, the IEC/IRB will be asked to review and reapprove this study, where required.

At the end of the study, the investigator (or sponsor where required) will notify the IEC/IRB about the study completion.

Country/Territory Selection

This study will only be conducted in those countries/territories where the intent is to launch or otherwise help ensure access to the developed product if the need for the product persists, unless explicitly addressed as a specific ethical consideration in Section 4.2.1, Study-Specific Ethical Design Considerations.

Other Ethical Considerations

For study-specific ethical design considerations, refer to Section 4.2.1.

10.2.2. Financial Disclosure

Refer to Required Prestudy Documentation (above) for details on financial disclosure.

10.2.3. Informed Consent Process

Each participant must give written consent according to the local requirements after the nature of the study has been fully explained. Informed consent for additional study procedures will be requested from participants in the Immuno Subset and/or the Safety Subset. The ICF(s) must be signed before performance of any study-related activity. The ICF(s) that is/are used must be approved by both the sponsor and by the reviewing IEC/IRB and be in a language that the participant can read and understand. The informed consent must be in accordance with principles that originated in the Declaration of Helsinki, current ICH and GCP guidelines, applicable regulatory requirements, and sponsor policy.

Informed consent may be obtained remotely.

Before enrollment in the study, the investigator or an authorized member of the study site personnel must explain to potential participants the aims, methods, reasonably anticipated benefits, and potential hazards of the study, and any discomfort participation in the study may entail. Participants will be informed that their participation is voluntary and that they may withdraw consent to participate at any time. They will be informed that choosing not to participate will not affect the care the participant will receive. Finally, they will be told that the investigator will maintain a participant identification register for the purposes of long-term follow up if needed and that their records may be accessed by health authorities and authorized sponsor personnel without violating the confidentiality of the participant, to the extent permitted by the applicable law(s) or regulations. By signing the ICF the participant is authorizing such access. It also denotes that the participant agrees to allow the study physician to recontact the participant for the purpose of obtaining consent for additional safety evaluations, if needed.

The participant will be given sufficient time to read the ICF and the opportunity to ask questions. After this explanation and before entry into the study, consent must be appropriately recorded by means of the participant's personally dated signature. After having obtained the consent, a copy of the ICF must be given to the participant.

Participants who are rescreened are required to sign a new ICF.

As described in Section 8, a caregiver may assist a participant who is unable to complete the eDiary, by reading the questions aloud and recording the responses in the eDiary on the participant's behalf (using the caregiver's unique identifier and PIN). For this purpose, a caregiver consent form has been developed. Consent must be obtained according to local requirements and must be obtained from the caregiver before the caregiver is allowed to complete the eDiary on behalf of the participant. After having obtained the caregiver's consent, a copy of the consent form must be given to the caregiver. Of note, the caregiver is not intended to be a Legally Authorized Representative who can provide informed consent for study participation on behalf of the participant.

10.2.4. Data Protection

Privacy of Personal Data

The collection and processing of personal data from participants enrolled in this study will be limited to those data that are necessary to fulfill the objectives of the study.

These data must be collected and processed with adequate precautions to ensure confidentiality and compliance with applicable data privacy protection laws and regulations. Appropriate technical and organizational measures to protect the personal data against unauthorized disclosures or access, accidental or unlawful destruction, or accidental loss or alteration must be put in place. Sponsor personnel whose responsibilities require access to personal data agree to keep the identity of participants confidential.

The informed consent obtained from the participants includes explicit consent for the processing of personal data and for the investigator/institution to allow direct access to their original medical

records (source data/documents) for study-related monitoring, audit, IEC/IRB review, and regulatory inspection. This consent also addresses the transfer of the data to other entities and to other countries/territories.

The participants have the right to request through the investigator access to their personal data and the right to request rectification of any data that are not correct or complete. Reasonable steps will be taken to respond to such a request, taking into consideration the nature of the request, the conditions of the study, and the applicable laws and regulations.

10.2.5. Long-Term Retention of Samples for Additional Future Research

Samples collected in this study may be stored for up to 15 years (or according to local regulations) for additional research. Samples will only be used to understand Ad26/protein preF RSV vaccine, to understand RSV and other respiratory pathogens, to understand differential vaccine responders, and to develop tests/assays related to Ad26/protein preF RSV vaccine and RSV and other respiratory pathogens. The research may begin at any time during the study or the post-study storage period.

Stored samples will be coded throughout the sample storage and analysis process and will not be labeled with personal identifiers. Participants may withdraw their consent for their samples to be stored for research (refer to Section 7.2.1, Withdrawal From the Use of Research Samples).

10.2.6. Committees Structure

10.2.6.1. Independent Data Monitoring Committee

An IDMC will be established. In general, the IDMC will monitor safety data on a regular basis throughout the study to ensure the continuing safety of the participants. In addition, the IDMC will also formally monitor the primary efficacy endpoints. The IDMC will evaluate in an unblinded fashion if the success criteria have been met.

This committee will consist of at least one medical expert in the relevant therapeutic area and at least one statistician; committee membership responsibilities, authorities, and procedures will be documented in its charter.

10.2.6.2. Clinical Event Adjudication Committee

Prior to database lock for all planned analyses (see Section 9.5), the CEC will review in a blinded manner all available data of all RSV-mediated ARIs and will confirm if the RT-PCR-confirmed RSV LRTD definition criteria were met. Further for all RT-PCR-confirmed RSV LRTD cases the CEC will assess their severity (mild, moderate or severe).

The CEC will consist of independent medical experts in the relevant therapeutic area. A Charter will be developed to describe the members, roles, and responsibilities of the CEC appointed to perform this review.

10.2.7. Publication Policy/Dissemination of Clinical Study Data

All information, including but not limited to information regarding Ad26/protein preF RSV vaccine or the sponsor's operations (eg, patent application, formulas, manufacturing processes, basic scientific data, prior clinical data, formulation information) supplied by the sponsor to the investigator and not previously published, and any data, including research data, generated as a result of this study, are considered confidential and remain the sole property of the sponsor. The investigator agrees to maintain this information in confidence and use this information only to accomplish this study and will not use it for other purposes without the sponsor's prior written consent.

The investigator understands that the information developed in the study will be used by the sponsor in connection with the continued development of Ad26/protein preF RSV vaccine, and thus may be disclosed as required to other clinical investigators or regulatory agencies. To permit the information derived from the clinical studies to be used, the investigator is obligated to provide the sponsor with all data obtained in the study.

The results of the study will be reported in a Clinical Study Report generated by the sponsor and will contain data from all study sites that participated in the study as per protocol. Recruitment performance or specific expertise related to the nature and the key assessment parameters of the study will be used to determine a coordinating investigator for the study. Results of analyses performed after the Clinical Study Report has been issued will be reported in a separate report and will not require a revision of the Clinical Study Report.

Study participant identifiers will not be used in publication of results. Any work created in connection with performance of the study and contained in the data that can benefit from copyright protection (except any publication by the investigator as provided for below) shall be the property of the sponsor as author and owner of copyright in such work.

Consistent with Good Publication Practices and International Committee of Medical Journal Editors (ICMJE) guidelines, the sponsor shall have the right to publish such primary (multicenter) data and information without approval from the investigator. The investigator has the right to publish study site-specific data after the primary data are published. If an investigator wishes to publish information from the study, a copy of the manuscript must be provided to the sponsor for review at least 60 days before submission for publication or presentation. Expedited reviews will be arranged for abstracts, poster presentations, or other materials. If requested by the sponsor in writing, the investigator will withhold such publication for up to an additional 60 days to allow for filing of a patent application. In the event that issues arise regarding scientific integrity or regulatory compliance, the sponsor will review these issues with the investigator. The sponsor will not mandate modifications to scientific content and does not have the right to suppress information. For multicenter study designs and sub-study approaches, secondary results generally should not be published before the primary endpoints of a study have been published. Similarly, investigators will recognize the integrity of a multicenter study by not submitting for publication data derived from the individual study site until the combined results from the completed study have been submitted for publication, within 18 months after the study end date, or the sponsor confirms there

will be no multicenter study publication. Authorship of publications resulting from this study will be based on the guidelines on authorship, such as those described in the ICMJE Recommendations for the Conduct, Reporting, Editing and Publication of Scholarly Work in Medical Journals, which state that the named authors must have made a significant contribution to the conception or design of the work; or the acquisition, analysis, or interpretation of the data for the work; and drafted the work or revised it critically for important intellectual content; and given final approval of the version to be published; and agreed to be accountable for all aspects of the work in ensuring that questions related to the accuracy or integrity of any part of the work are appropriately investigated and resolved.

Registration of Clinical Studies and Disclosure of Results

The sponsor will register and disclose the existence of and the results of clinical studies as required by law. The disclosure of the final study results will be performed after the end of study in order to ensure the statistical analyses are relevant.

10.2.8. Data Quality Assurance

Data Quality Assurance/Quality Control

Steps to be taken to ensure the accuracy and reliability of data include the selection of qualified investigators and appropriate study sites, review of protocol procedures with the investigator and study site personnel before the study, and periodic monitoring visits by the sponsor. Written instructions will be provided for collection, handling, storage, and shipment of samples.

Guidelines for eCRF completion will be provided and reviewed with study site personnel before the start of the study. The sponsor may review the eCRF for accuracy and completeness during on-site monitoring visits and after transmission to the sponsor; any discrepancies will be resolved with the investigator or designee, as appropriate. After upload of the data into the study database they will be verified for accuracy and consistency with the data sources.

10.2.9. Case Report Form Completion

Case report forms are prepared and provided by the sponsor for each participant in electronic format. All data relating to the study must be recorded in the eCRF. All eCRF entries, corrections, and alterations must be made by the investigator or authorized study site personnel. The investigator must verify that all data entries in the eCRF are accurate and correct.

The study data will be transcribed by study site personnel from the source documents onto an electronic eCRF, if applicable. Study-specific data will be transmitted in a secure manner to the sponsor.

Worksheets may be used for the capture of some data to facilitate completion of the eCRF. Any such worksheets will become part of the participant's source documents. Data must be entered into the eCRF in English. The eCRF must be completed as soon as possible after a participant visit and the forms must be available for review at the next scheduled monitoring visit.

If necessary, queries will be generated in the eDC tool. If corrections to an eCRF are needed after the initial entry into the eCRF, this can be done in either of the following ways:

- Investigator and study site personnel can make corrections in the eDC tool at their own initiative or as a response to an auto query (generated by the eDC tool).
- Sponsor or sponsor delegate can generate a query for resolution by the investigator and study site personnel.

10.2.10. Source Documents

At a minimum, source documents consistent in the type and level of detail with that commonly recorded at the study site as a basis for standard medical care must be available for the following: participant identification, eligibility, and study identification; study discussion and date of signed informed consent; dates of visits; results of safety and efficacy parameters as required by the protocol; record of all AEs and follow-up of AEs; concomitant medication; vaccination receipt/dispensing/return records; study vaccine administration information; and date of study completion and reason for early discontinuation of study vaccine or withdrawal from the study, if applicable.

The author of an entry in the source documents must be identifiable.

Information from the participant eDiary will be reviewed by the investigator. The participant's eDiary used to collect information regarding solicited signs and symptoms after vaccination will be considered source data.

Participant-completed scales and assessments designated by the sponsor (solicited AEs after vaccination [including body temperature], RiiQ, and ARI surveillance responses) will be recorded directly into an electronic device and will be considered source data.

Specific details required as source data for the study and source data collection methods will be reviewed with the investigator before the study and will be described in the monitoring guidelines (or other equivalent document).

An eSource system may be utilized, which contains data traditionally maintained in a hospital or clinic record to document medical care (eg, electronic source documents) as well as the clinical study-specific data fields as determined by the protocol. This data is electronically extracted for use by the sponsor. If eSource is utilized, references made to the eCRF in the protocol include the eSource system but information collected through eSource may not be limited to that found in the eCRF. Data in this system may be considered source documentation.

10.2.11. Monitoring

The sponsor will use a combination of monitoring techniques (central, remote, or on-site monitoring) to monitor this study.

The sponsor or its designee will perform on-site monitoring visits as frequently as necessary. The monitor will record dates of the visits in a study site visit log that will be kept at the study site. The

first post-initiation visit will be made as soon as possible after enrollment has begun. At these visits, the monitor may compare data entered into the eCRF with the source documents (eg, hospital/clinic/physician's office medical records); a sample may be reviewed. The nature and location of all source documents will be identified to ensure that all sources of original data required to complete the eCRF are known to the sponsor and study site personnel and are accessible for verification by the sponsor study site contact. If electronic records are maintained at the study site, the method of verification must be discussed with the study site personnel.

Direct access to source documents (medical records) must be allowed for the purpose of verifying that the recorded data are consistent with the original source data. Findings from this review will be discussed with the study site personnel. The sponsor expects that, during monitoring visits, the relevant study site personnel will be available, the source documents will be accessible, and a suitable environment will be provided for review of study-related documents. The monitor will meet with the investigator on a regular basis during the study to provide feedback on the study conduct.

In addition to on-site monitoring visits, remote contacts can occur. It is expected that during these remote contacts, study site personnel will be available to provide an update on the progress of the study at the site.

Central monitoring will take place for data identified by the sponsor as requiring central review.

10.2.12. On-Site Audits

Representatives of the sponsor's clinical quality assurance department may visit the study site at any time during or after completion of the study to conduct an audit of the study in compliance with regulatory guidelines and company policy. These audits will require access to all study records, including source documents, for inspection. Participant privacy must, however, be respected. The investigator and study site personnel are responsible for being present and available for consultation during routinely scheduled study site audit visits conducted by the sponsor or its designees.

Similar auditing procedures may also be conducted by agents of any regulatory body, either as part of a national GCP compliance program or to review the results of this study in support of a regulatory submission. The investigator must immediately notify the sponsor if the investigator has been contacted by a regulatory agency concerning an upcoming inspection.

10.2.13. Record Retention

In compliance with the ICH/GCP guidelines, the investigator/institution will maintain all eCRF and all source documents that support the data collected from each participant, as well as all study documents as specified in ICH/GCP Section 8, Essential Documents for the Conduct of a Clinical Trial, and all study documents as specified by the applicable regulatory requirement(s). The investigator/institution will take measures to prevent accidental or premature destruction of these documents.

Essential documents must be retained until at least 2 years after the last approval of a marketing application in an ICH region and until there are no pending or contemplated marketing applications in an ICH region or until at least 2 years have elapsed since the formal discontinuation of clinical development of the investigational product. These documents will be retained for a longer period if required by the applicable regulatory requirements or by an agreement with the sponsor. It is the responsibility of the sponsor to inform the investigator/ institution as to when these documents no longer need to be retained.

If the responsible investigator retires, relocates, or for other reasons withdraws from the responsibility of keeping the study records, custody must be transferred to a person who will accept the responsibility. The sponsor must be notified in writing of the name and address of the new custodian. Under no circumstance shall the investigator relocate or dispose of any study documents before having obtained written approval from the sponsor.

If it becomes necessary for the sponsor or the appropriate regulatory authority to review any documentation relating to this study, the investigator/institution must permit access to such reports.

10.2.14. Study and Site Start and Closure

First Act of Recruitment

The first site open is considered the first act of recruitment and it becomes the study start date.

Study/Site Termination

The sponsor reserves the right to close the study site or terminate the study at any time for any reason at the sole discretion of the sponsor. Study sites will be closed upon study completion. A study site is considered closed when all required documents and study supplies have been collected and a study site closure visit has been performed.

The investigator may initiate study site closure at any time, provided there is reasonable cause and sufficient notice is given in advance of the intended termination.

Reasons for the early closure of a study site by the sponsor or investigator may include but are not limited to:

- Failure of the investigator to comply with the protocol, the requirements of the IEC/IRB or local health authorities, the sponsor's procedures, or GCP guidelines
- Inadequate recruitment of participants by the investigator
- Discontinuation of further study vaccine development

10.3. Appendix 3: Adverse Events, Serious Adverse Events, Adverse Event of Special Interest, Product Quality Complaints, and Other Safety Reporting: Definitions and Procedures for Recording, Evaluating, Follow-up, and Reporting

10.3.1. Adverse Event Definitions and Classifications

Adverse Event

An AE is any untoward medical occurrence in a clinical study participant administered a pharmaceutical (investigational or non-investigational) product. An AE does not necessarily have a causal relationship with the intervention. An AE can therefore be any unfavorable and unintended sign (including an abnormal finding), symptom, or disease temporally associated with the use of a medicinal (investigational or non-investigational) product, whether or not related to that medicinal (investigational or non-investigational) product. (Definition per ICH)

This includes any occurrence that is new in onset or aggravated in severity or frequency from the baseline condition, or abnormal results of diagnostic procedures, including laboratory test abnormalities.

Note: For time period of sponsor's AE collection, see All Adverse Events under Section 8.5.1, Time Period and Frequency for Collecting Adverse Event and Serious Adverse Event Information.

Serious Adverse Event

An SAE based on ICH and EU Guidelines on Pharmacovigilance for Medicinal Products for Human Use is any untoward medical occurrence that at any dose:

- Results in death
- Is life-threatening
  (The participant was at risk of death at the time of the event. It does not refer to an event that hypothetically might have caused death if it were more severe.)
- Requires inpatient hospitalization or prolongation of existing hospitalization
- Results in persistent or significant disability/incapacity
- Is a congenital anomaly/birth defect
- Is a suspected transmission of any infectious agent via a medicinal product
- Is Medically Important*

*Medical and scientific judgment must be exercised in deciding whether expedited reporting is also appropriate in other situations, such as important medical events that may not be immediately life threatening or result in death or hospitalization but may jeopardize the participant or may require intervention to prevent one of the other outcomes listed in the definition above. These should usually be considered serious.

If a serious and unexpected AE occurs for which there is evidence suggesting a causal relationship between the study vaccine and the event (eg, death from anaphylaxis), the event must be reported

as a SUSAR by the sponsor to health authorities and by the investigator to the IRB/IEC according to regulatory and local requirements.

Unlisted (Unexpected) Adverse Event/Reference Safety Information

An AE is considered unlisted if the nature or severity is not consistent with the applicable product reference safety information. For Ad26.RSV.preF and RSV preF protein, expectedness of an AE will be determined by whether or not it is listed in the Investigator's Brochure.

10.3.2. Attribution Definitions

Assessment of Causality

The causal relationship to study vaccine is determined by the investigator. The following selection must be used to assess all AEs.

Related

There is a reasonable causal relationship between study vaccine administration and the AE.

Not Related

There is not a reasonable causal relationship between study vaccine administration and the AE.

The term "reasonable causal relationship" means there is evidence to support a causal relationship.

By definition, all solicited AEs at the injection site (local) will be considered related to the study vaccine administration.

10.3.3. Severity Criteria

All AEs will be coded for severity using a modified version of the FDA grading table, based on version of September 2007 (https://www.fda.gov/media/73679/download), included in Appendix 4: Toxicity Grading Scale. For AEs not identified in the grading table, the following guidelines will be applied:

**Grade 1**	Mild	Symptoms causing no or minimal interference with usual social and functional activities
**Grade 2**	Moderate	Symptoms causing greater than minimal interference with usual social and functional activities
**Grade 3**	Severe	Symptoms causing inability to perform usual social and functional activities and requires medical intervention
**Grade 4**	Potentially life-threatening	Symptoms causing inability to perform basic self-care functions, *or* medical or operative intervention indicated to prevent permanent impairment, persistent disability, *or* ER visit or hospitalization

The severity of solicited signs and symptoms will be graded in the eDiary by the participant based on the severity assessment provided in the eDiary and then verified by the investigator using the toxicity grading scale in Appendix 4: Toxicity Grading Scale. (Note: severity of the measured

events will be derived from the diameter [for erythema and swelling] and the temperature measurements [for fever]).

10.3.4. Special Reporting Situations

Safety events of interest on a sponsor study vaccine in an interventional study that may require expedited reporting or safety evaluation include, but are not limited to:

- Overdose of a sponsor study vaccine
- Suspected abuse/misuse of a sponsor study vaccine
- Accidental or occupational exposure to a sponsor study vaccine
- Medication error, intercepted medication error, or potential medication error involving a Johnson & Johnson medicinal product (with or without patient exposure to the Johnson & Johnson medicinal product, eg, product name confusion, product label confusion, intercepted prescribing or dispensing errors)

Special reporting situations must be recorded in the eCRF. Any special reporting situation that meets the criteria of an SAE must be recorded on the SAE page of the eCRF.

10.3.5. Procedures

All Adverse Events

AEs, regardless of seriousness, severity, or presumed relationship to study vaccine, must be recorded using medical terminology in the source document and the eCRF. Whenever possible, diagnoses should be given when signs and symptoms are due to a common etiology (eg, cough, runny nose, sneezing, sore throat, and head congestion should be reported as “upper respiratory infection”). Investigators must record in the eCRF their opinion concerning the relationship of the AE to the study vaccine. All measures required for AE management must be recorded in the source document and reported according to sponsor instructions.

The participant will be provided with a “wallet (study) card” and instructed to carry this card with them for the duration of the study indicating the following:

- Study number
- Statement, in the local language(s), that the participant is participating in a clinical study
- Investigator’s name and 24hour contact telephone number
- Local sponsor’s name and 24-hour contact telephone number (for medical personnel only)
- Site number
- Participant number
- Any other information that is required to do an emergency breaking of the blind

Serious Adverse Events

All SAEs that have not resolved by the end of the study, or that have not resolved upon the participant's discontinuation from the study, must be followed until any of the following occurs:

- The event resolves
- The event stabilizes
- The event returns to baseline, if a baseline value/status is available
- The event can be attributed to agents other than the study vaccine or to factors unrelated to study conduct
- It becomes unlikely that any additional information can be obtained (participant or healthcare practitioner refusal to provide additional information, lost to follow-up after demonstration of due diligence with follow-up efforts)

Any event requiring hospitalization (or prolongation of hospitalization) that occurs during participation in the study must be reported as an SAE, except hospitalizations for the following:

- Hospitalizations not intended to treat an acute illness or AE (eg, social reasons such as pending placement in long-term care facility)
- Surgery or procedure planned before entry into the study (must be documented in the eCRF). Note: Hospitalizations that were planned before the signing of the ICF, and where the underlying condition for which the hospitalization was planned has not worsened, will not be considered SAEs. Any AE that results in a prolongation of the originally planned hospitalization is to be reported as a new SAE.

The cause of death of a participant in a study, whether or not the event is expected or associated with the study vaccine, is considered an SAE.

Information regarding SAEs will be transmitted to the sponsor using an SAE reporting form and safety report form of the eCRF, which must be completed and reviewed by a physician from the study site, and transmitted in a secure manner to the sponsor within 24 hours. The initial and follow-up reports of an SAE should be transmitted in a secure manner electronically or by facsimile (fax). Telephone reporting should be the exception and the reporter should be asked to complete the appropriate form(s) first.

Adverse Events of Special Interest

AESIs, including potential AESIs, will be carefully monitored during the study by the sponsor and must be reported to the sponsor within 24 hours of awareness irrespective of seriousness (ie, serious and nonserious AEs) or causality assessment, following the procedure described above for SAEs and will require enhanced data collection.

10.3.6. Product Quality Complaint Handling

Definition

A Product Quality Complaint (PQC) is defined as any suspicion of a product defect related to manufacturing, labeling, or packaging, ie, any dissatisfaction relative to the identity, quality, durability, reliability, or performance of a distributed product, including its labeling, drug delivery system, or package integrity. A PQC may have an impact on the safety and efficacy of the product. In addition, it includes any technical complaints, defined as any complaint that indicates a potential quality issue during manufacturing, packaging, release testing, stability monitoring, dose preparation, storage or distribution of the product or the drug delivery system.

Procedures

All initial PQCs must be reported to the sponsor by the study site personnel within 24 hours after being made aware of the event.

A sample of the suspected product should be maintained under the correct storage conditions until a shipment request is received from the sponsor.

10.3.7. Contacting Sponsor Regarding Safety, Including Product Quality

The names (and corresponding telephone numbers) of the individuals who must be contacted regarding safety issues, PQC, or questions regarding the study are listed in the Contact Information page(s), which will be provided as a separate document.

10.4. Appendix 4: Toxicity Grading Scale

Adapted from the FDA Guidance document "Toxicity Grading Scale for Healthy Adult and Adolescent Volunteers Enrolled in Preventive Vaccine Clinical Trials" (September 2007)

A: Tables for Clinical Abnormalities

**Local Reaction to Injectable Product**	**Mild (Grade 1)**	**Moderate (Grade 2)**	**Severe (Grade 3)**	**Potentially Life-threatening (Grade 4)**
Pain/Tenderness[#]	Aware of symptoms but easily tolerated; Does not interfere with activity; Discomfort only to touch	Notable symptoms; Requires modification in activity or use of medications; Discomfort with movement	Incapacitating symptoms; Inability to do work, school, or usual activities; Use of narcotic pain reliever	Hospitalization; Pain/tenderness causing inability to perform basic self-care function
Erythema[#]	25-50 mm	51-100 mm	>100 mm	Hospitalization; Necrosis or exfoliative dermatitis
Swelling[#]	25-50 mm	51-100 mm	>100 mm	Hospitalization; Necrosis

[#] Revised by the sponsor.

**Vital Signs ***	**Mild (Grade 1)**	**Moderate (Grade 2)**	**Severe (Grade 3)**	**Potentially Life-threatening (Grade 4)**
Fever (°C) ** (°F) **	38.0-38.4 100.4-101.1	38.5-38.9 101.2-102.0	39.0-40.0 102.1-104.0	>40 >104.0
Tachycardia – beats per minute	101-115	116-130	>130	Hospitalization for arrhythmia[#]
Bradycardia – beats per minute***	50-54	45-49	<45	Hospitalization for arrhythmia[#]
Hypertension (systolic) – mm Hg	141-150	151-160[#]	>160[#]	Hospitalization for malignant hypertension[#]
Hypertension (diastolic) – mm Hg	91-95	96-100	>100	Hospitalization for malignant hypertension[#]
Hypotension (systolic) – mm Hg	85-89	80-84	<80	Hospitalization for hypotensive shock[#]
Respiratory Rate – breaths per minute	17-20	21-25	>25	Intubation

* Participant should be at rest for all vital sign measurements.
** For oral temperature: no recent hot or cold beverages or smoking.
***When resting heart rate is between 60 – 100 beats per minute. Use clinical judgment when characterizing bradycardia among some healthy participant populations, for example, conditioned athletes.
[#] Revised by the sponsor.

**Systemic (General)**	**Mild (Grade 1)**	**Moderate (Grade 2)**	**Severe (Grade 3)**	**Potentially Life-threatening (Grade 4)**
Vomiting#	No interference with activity or 1-2 episodes/24 hours	Some interference with activity or >2 episodes/24 hours	Prevents daily activity, requires outpatient IV hydration	Hospitalization; Hypotensive shock
Nausea#	Minimal symptoms; causes minimal or no interference with work, school, or self-care activities	Notable symptoms; Requires modification in activity or use of medications; Does NOT result in loss of work, school, or cancellation of social activities	Incapacitating symptoms; Requires bed rest and/or results in loss of work, school, or cancellation of social activities	Hospitalization; Inability to perform basic self-care functions
Diarrhea#	2-3 loose stools or <400 gms/24 hours	4-5 stools or 400-800 gms/ 24 hours	6 or more watery stools or >800 gms/24 hours or oral rehydration necessary	Hospitalization; Hypotensive shock *OR* IV fluid replacement indicated
Headache#	Minimal symptoms; causes minimal or no interference with work, school, or self-care activities	Notable symptoms; Requires modification in activity or use of medications; Does NOT result in loss of work, school, or cancellation of social activities	Incapacitating symptoms; Requires bed rest and/or results in loss of work, school, or cancellation of social activities; Use of narcotic pain reliever	Hospitalization; Inability to perform basic self-care functions
Fatigue#	Minimal symptoms; causes minimal or no interference with work, school, or self-care activities	Notable symptoms; Requires modification in activity or use of medications; Does NOT result in loss of work, school, or cancellation of social activities	Incapacitating symptoms; Requires bed rest and/or results in loss of work, school, or cancellation of social activities; Use of narcotic pain reliever	Hospitalization; Inability to perform basic self-care functions
Myalgia#	Minimal symptoms; causes minimal or no interference with work, school, or self-care activities	Notable symptoms; Requires modification in activity or use of medications; Does NOT result in loss of work, school, or cancellation of social activities	Incapacitating symptoms; Requires bed rest and/or results in loss of work, school, or cancellation of social activities; Use of narcotic pain reliever	Hospitalization; Inability to perform basic self-care functions

Revised by the sponsor.

**Systemic Illness**	**Mild (Grade 1)**	**Moderate (Grade 2)**	**Severe (Grade 3)**	**Potentially Life-threatening (Grade 4)**
Illness or clinical adverse event (as defined according to applicable regulations)	No interference with activity	Some interference with activity not requiring medical intervention	Prevents daily activity and requires medical intervention	Hospitalization#

Revised by the sponsor.

10.5. Appendix 5: Definition of Acute Respiratory Infection for Participants Aged ≥60 Years

Acute respiratory infection (ARI) is defined as the occurrence of at least one respiratory symptom that the participant reports is different or worse than the participant usually experiences:

- Respiratory symptom: an upper respiratory tract infection (URTI) symptom (ie, nasal congestion, sore throat) or a lower respiratory tract infection (LRTI) symptom (ie, cough, short of breath, coughing up phlegm [sputum], wheezing) from the RiiQ™v2 Symptom Scale)

Resolution of an ARI episode is defined as 2 consecutive days with no symptoms listed on the RiiQ™v2 Symptom Scale or, for participants who have RiiQ™v2 symptoms present at baseline (assessed pre-vaccination), 2 consecutive days where all symptoms on the RiiQ™v2 Symptom Scale have returned to same severity level as reported at baseline (or lower).

10.6. Appendix 6: Medically-attended ARI Form

Section 1: To be completed in all healthcare settings (e.g., family doctor, nurse practitioner, outpatient clinic, emergency department visits and hospitalizations).

**Participant ID (will be completed by study staff):**
**Date of visit:**
**Name and role of healthcare professional completing form:**
**Optional contact details:**

**DIAGNOSIS/DIAGNOSES**
Please list diagnosis or diagnoses made during clinical interaction here.

**MEDICATIONS**
Please list any new medications prescribed or changes in medication dosing.

**VITAL SIGNS**
**Temperature (ºC/ºF): ________________**
**Respiratory rate (breaths per minute): _________________**<br>**Does the measured respiratory rate support a diagnosis of lower respiratory tract disease (LRTD)?**<br>□ **Yes** □ **No**<br><br>*Below question only to be completed if patient has history of COPD or asthma.*<br>**Does the measured respiratory rate support a diagnosis of a COPD or asthma exacerbation?**<br>□ **COPD exacerbation** □ **asthma exacerbation** □ **No**
**Oxygen saturation (SpO_2): __________________**<br>**Does the measured oxygen saturation support a diagnosis of lower respiratory tract disease (LRTD)?**<br>□ **Yes** □ **No**<br><br>*Below question only to be completed if patient has history of COPD or asthma.*<br>**Does the measured oxygen saturation support a diagnosis of a COPD or asthma exacerbation?**<br>□ **COPD exacerbation** □ **asthma exacerbation** □ **No**
**WERE ADDITIONAL DIAGNOSTIC TESTS PERFORMED?** □ **Yes** □ **No**<br>*If 'yes' selected, please fill out remaining questions in this section.*
**Was a chest x-ray performed?** □ **Yes** □ **No**<br><br>**If yes, please indicate date performed: ___________________**<br>**If yes, do the results support a diagnosis of lower respiratory tract disease (LRTD)?** □ **Yes** □ **No**<br>**If LRTD diagnosis supported, further diagnosis by x-ray:** □ **Pneumonia** □ **Other LRTD**

**Was a peak flow measurement made?**<br><br>**If yes, please indicate date performed: ___________________**<br><br>**Peak flow (L/min): ______________**<br>**If yes, do the results support a diagnosis of LRTD?**<br><br>*Below question only to be completed if patient has history of COPD or asthma.*<br>**Do the results support a diagnosis of a COPD or asthma exacerbation?**<br>□ **COPD exacerbation** □ **asthma exacerbation** □ **No**	□ **Yes** □ **No**<br><br><br><br><br>□ **Yes** □ **No**
**Was spirometry performed?**<br><br>**If yes, please indicate date performed: ___________________**<br>**Specify results:**<br>*Only indicate results for measurements made.*<br><br>**FEV$_1$ (L): ________**<br><br>**FEV$_6$ (L): ________**<br><br>**FVC (L): _________**<br>**If yes, do the results support a diagnosis of LRTD?**<br><br>*Below question only to be completed if patient has history of COPD or asthma.*<br>**Do the results support a diagnosis of a COPD or asthma exacerbation?**<br>□ **COPD exacerbation** □ **asthma exacerbation** □ **No**	□ **Yes** □ **No**<br><br><br><br><br><br><br><br><br><br><br>□ **Yes** □ **No**
**Was another diagnostic method used?**<br><br>**If yes, please specify diagnostic method: ____**<br><br>**Date performed: ______________**<br><br>**Specify results: ____**<br>**Do the results support a diagnosis of LRTD?**	□ **Yes** □ **No**<br><br><br><br><br><br><br>□ **Yes** □ **No**
**WAS ANY MANAGEMENT PRESCRIBED OTHER THAN MEDICATION?**<br>*(including nebulizer treatments, IV fluids, etc.)*<br>*If 'yes' selected, please fill out remaining questions in this section.*	□ **Yes** □ **No**
**If yes, please specify:**	

Section 2: Additional questions only to be completed if patient seen in emergency department or is hospitalized.

WERE ADDITIONAL DIAGNOSTIC TESTS PERFORMED? □ **Yes** □ **No**
If 'yes' selected, please fill out remaining questions in this section.

Was a CT-scan performed? □ **Yes** □ **No**

If yes, please indicate date performed: ___________________
If yes, do the results support a diagnosis of LRTD? □ **Yes** □ **No**
If LRTD diagnosis supported, further diagnosis by CT: □ **Pneumonia** □ **Other LRTD**

Was an MRI performed? □ **Yes** □ **No**

If yes, please indicate date performed: ___________________
If yes, do the results support a diagnosis of LRTD? □ **Yes** □ **No**
If LRTD diagnosis supported, further diagnosis by MRI: □ **Pneumonia** □ **Other LRTD**

Was bronchoscopy performed? □ **Yes** □ **No**

If yes, please indicate date performed: ___________________
Specify results: ____
If yes, do the results support a diagnosis of LRTD? □ **Yes** □ **No**

Was an arterial blood gas measured? □ **Yes** □ **No**

If yes, please indicate date performed: ___________________

Specify results: pH: _________, **pCO_2 (mmHg):** _________, **pO_2 (mmHg):** _________, **HCO_3 (mEq/L):** _________,
O_2 saturation (%): _________
If yes, do the results support a diagnosis of LRTD? □ **Yes** □ **No**

Below question only to be completed if patient has history of COPD or asthma.
Do the results support a diagnosis of a COPD or asthma exacerbation?
□ **COPD exacerbation** □ **asthma exacerbation** □ **No**

Was another diagnostic method used? □ **Yes** □ **No**

If yes, please specify diagnostic method: ____

Date performed: ______________

Specify results: ____
Do the results support a diagnosis of LRTD? □ **Yes** □ **No**

SUPPLEMENTAL OXYGEN

Was supplemental oxygen administered? □ **Yes** □ **No**
If 'yes' selected, please fill out remaining questions in this section.

Type of supplemental oxygen administration:
□ **Invasive Mechanical Ventilation**
□ **Non-Invasive Mechanical Ventilation**
□ **Nasal Cannula**
□ **Nonrebreathing Face Mask with Reservoir and One-Way Valve**
□ **Venturi Mask**
□ **Simple Face Mask**
□ **Reservoir Cannulas**

□ **Other:** _______________________

If invasive mechanical ventilation, specify:
□ **Through endotracheal tube** □ **Through tracheostomy tube**

If non-invasive mechanical ventilation, specify:
□ **Continuous positive airway pressure** □ **Bilevel positive airway pressure**

Oxygen concentration and units: ________________________

Start date and time: _____________________

End date and time (if applicable): ___________________

Has supplemental oxygen administration returned to that level provided prior to the current respiratory illness?
□ **Yes** □ **No**

Below question only to be completed if patient has history of COPD or asthma.
If applicable, does requirement for oxygen supplementation or increased oxygen supplementation support a diagnosis of a COPD or asthma exacerbation?

□ **COPD exacerbation** □ **asthma exacerbation** □ **No**

**ADDITIONAL COMMENTS**

10.7. Appendix 7: ARI Surveillance Assessment

Since the last time you completed your eDiary, have you had any symptoms of a cold or respiratory infection such as nasal congestion (a stuffy or runny nose), a sore throat, a cough, coughing up phlegm (sputum), trouble breathing (short of breath), or whistling sounds when you breathe (wheezing)?

If you usually have some of these symptoms even when you do not have a cold, please only answer "yes" if you have more symptoms than usual or your symptoms are worse than usual.

Responses:

No cold symptoms *The participant will be referred to AE reminder screen (below)*

Yes, I had cold symptoms *The participant completes ARI assessment as follows:*

1. Date ARI symptoms started or became worse than usual
2. RiiQ™v2 Symptom Scale

The ePRO system will compare reported symptoms/severity to values reported during the vaccination visit.

If symptoms are the same or less severe than the participant reported during the vaccination visit, the symptoms indicate no ARI has occurred according to protocol definition. Therefore, the remainder of the ARI assessment is not collected. The participant will be referred to AE reminder screen (below).

If symptoms are worse than baseline, the remaining ARI assessments are presented in the following order:

3. RiiQ™v2 Impact on Daily Activities
4. EQ-5D-5L
5. PGI-H
6. PGI-S
7. PGI-C
   (Note that the PGI C will not be completed on ARI Day 1)
8. Return to Usual Health
   (Note that the Return to Usual Health question will not be completed on ARI Day 1)

The participant will then be referred to AE reminder screen (below). A final screen reminds participants to collect a nasal swab and to contact site to schedule a site visit.

Adverse Event Reminder Screen:

Thank you for completing your eDiary.

If you are having any severe symptoms, health issues, or other concerns about your health, please contact your study doctor to discuss these.

10.8. Appendix 8: Respiratory Infection Intensity and Impact Questionnaire (RiiQ™v2) – Symptom Scale and Impact on Daily Activity Scale

Please read each of the following questions and select the answer thinking about when you felt the worst in the past 24 hours.

1. <u>During the past 24 hours</u>, have you had the following symptoms?

	None	Mild	Moderate	Severe
a. Cough	□	□	□	□
b. Sore throat	□	□	□	□
c. Headache	□	□	□	□
d. Nasal congestion	□	□	□	□
e. Feeling feverish	□	□	□	□
f. Body aches and pains	□	□	□	□
g. Fatigue (tiredness)	□	□	□	□
h. Neck pain	□	□	□	□
i. Interrupted sleep	□	□	□	□
j. Wheezing	□	□	□	□
k. Coughing up phlegm (sputum)	□	□	□	□
l. Short of breath	□	□	□	□
m. Loss of appetite	□	□	□	□

2. During the past 24 hours, how able were you to:

	No Difficulty	Some Difficulty	Moderate Difficulty	Great Difficulty
a. Get out of bed	□	□	□	□
b. Leave your home	□	□	□	□
c. Prepare meals / get your own food	□	□	□	□
d. Perform usual activities	□	□	□	□
e. Concentrate on tasks	□	□	□	□
f. Take care of yourself	□	□	□	□
g. Go out of the room you are in	□	□	□	□

Questions 1 and 2 of the Respiratory Infection intensity and impact questionnaire (RiiQ™) ©RH Osborne (2006, 2018).
No part of the RiiQ™ may be copied or reproduced in any form without written permission from RH Osborne PhD:measuredsolutions@bigpond.com
(Variable recall Version. English. Administered under license by Janssen Pharmaceuticals).
(English – administration once daily)

10.9. Appendix 9 : EuroQoL, 5-Dimension, 5-Level Questionnaire (EQ-5D-5L)

Note: This appendix provides a representative example of the questionnaire that will be used in this study. The site should always use the most recently provided version of the questionnaire.

EQ-5D-5L

Health Questionnaire

English version for the UK

UK (English) © 2009 EuroQol Group. EQ 5D™ is a trade mark of the EuroQol Group

Under each heading, please check the ONE box that best describes your health TODAY.

MOBILITY

I have no problems in walking about ❑
I have slight problems in walking about ❑
I have moderate problems in walking about ❑
I have severe problems in walking about ❑
I am unable to walk about ❑

SELF-CARE

I have no problems washing or dressing myself ❑
I have slight problems washing or dressing myself ❑
I have moderate problems washing or dressing myself ❑
I have severe problems washing or dressing myself ❑
I am unable to wash or dress myself ❑

USUAL ACTIVITIES *(e.g. work, study, housework, family or leisure activities)*

I have no problems doing my usual activities ❑
I have slight problems doing my usual activities ❑
I have moderate problems doing my usual activities ❑
I have severe problems doing my usual activities ❑
I am unable to do my usual activities ❑

PAIN / DISCOMFORT

I have no pain or discomfort ❑
I have slight pain or discomfort ❑
I have moderate pain or discomfort ❑
I have severe pain or discomfort ❑
I have extreme pain or discomfort ❑

ANXIETY / DEPRESSION

I am not anxious or depressed ❑
I am slightly anxious or depressed ❑
I am moderately anxious or depressed ❑
I am severely anxious or depressed ❑
I am extremely anxious or depressed ❑

UK (English) © 2009 EuroQol Group. EQ 5D™ is a trade mark of the EuroQol Group

- We would like to know how good or bad your health is TODAY.
- This scale is numbered from 0 to 100.
- 100 means the best health you can imagine.
  0 means the worst health you can imagine.
- Mark an X on the scale to indicate how your health is TODAY.
- Now, please write the number you marked on the scale in the box below.

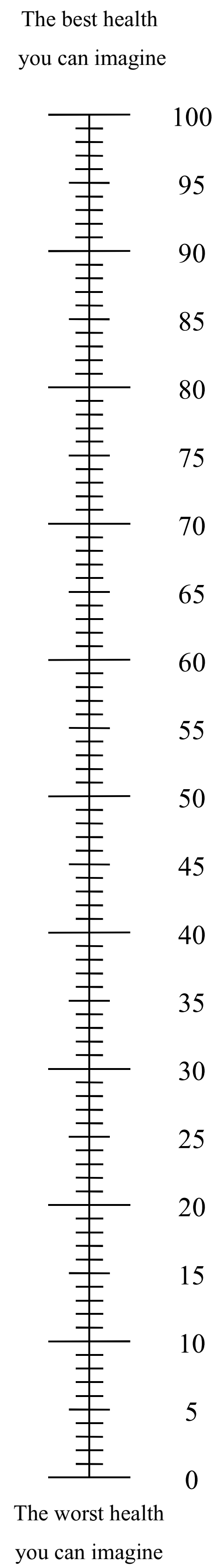

YOUR HEALTH TODAY

UK (English) © 2009 EuroQol Group. EQ 5D™ is a trade mark of the EuroQol Group

10.10. Appendix 10: Patient Global Impression Scales

Patient Global Impression of Health (PGI-H)

Overall, how would you rate your health today?

- ☐ Very good
- ☐ Good
- ☐ Fair
- ☐ Poor
- ☐ Very poor

Patient Global Impression of Severity (PGI-S)

Overall, how would you rate your respiratory illness today?

- ☐ None
- ☐ Mild
- ☐ Moderate
- ☐ Severe

Patient Global Impression of Change (PGI-C)

How was your respiratory illness today compared to when you first entered this study?

- ☐ Much better
- ☐ Somewhat better
- ☐ A little better
- ☐ About the same
- ☐ A little worse
- ☐ Somewhat worse
- ☐ Much worse

Return to Usual Health

Have you returned to your usual health today?

- ☐ Yes
- ☐ No

10.11. Appendix 11: Medical Resources Utilization Questionnaire

Participant ID:

Date (dd-mmm-yyyy):

During your ARI, did the interactions you had with healthcare providers for study-related visits and procedures affect the number of times you sought medical care outside of the study?

Yes No

If yes, what was the effect on the medical consultations that you sought outside of the study?

interactions with healthcare providers **increased** interactions with healthcare providers **decreased**

1. <u>Medical consultations</u>

Since onset of acute respiratory infection, how many times have you had medical consultations?

	No	Yes	Specify number of visits (include home visits, telephone consults, telemedicine consults, and in-person visits)	For each visit, please indicate:			
				if this was preplanned prior to ARI onset	if this was related to an ARI or its complications	a reason	if this was a home visit, an appointment by telephone, a telemedicine consult or an in-person visit
General Practitioner							
Nurse							
Internal Medicine							
Pulmonologist							
Respiratory Physiotherapy							
Other (Please specify)							

2. Hospital services

Since onset of acute respiratory infection, did you visit the hospital?

Yes:
No:

	No	Yes	Specify number of visits/ admissions	For each visit/admission, please indicate: if it was preplanned prior to ARI onset	if this was related to an ARI or its complications	the length of stay (days)	a reason	if supplemental oxygen was used	if mechanical ventilation was used
Emergency Department*									
Short term hospital admission (<24 hours admission)									
Hospitalization in general ward**									
Hospitalization in critical care**									

*Please count Emergency Department visits only if the visit did not result in a hospital admission.

** For single hospital admissions that had stays in different wards (ie, general ward and critical care), please list these admissions separately.

3. Institutional care admission(s) other than hospital

Please indicate if there has been any need for admission for care in a long-term facility, since onset of acute respiratory infection.

Yes:
No:

	No	Yes	Specify number of admissions	For each admission, please indicate: if this was related to an ARI or its complications	the length of stay (days)	a reason	if supplemental oxygen was used
Long term facilities							
Rehabilitation facility							

10.12. Appendix 12: Missed Working Days

Participant ID:

Date (dd-mmm-yyyy):

Missed working days

Do you normally work*? yes
no

** Either paid or volunteer work*

If yes, how many days from work* did you miss due to the ARI or its complications? days

10.13. Appendix 13: Guidance on Study Conduct During a Natural Disaster

GUIDANCE ON STUDY CONDUCT DURING THE COVID-19 PANDEMIC

It is recognized that the Coronavirus Disease 2019 (COVID-19) pandemic may have an impact on the conduct of this clinical study due to, for example, isolation or quarantine of participants and study-site personnel; travel restrictions/limited access to public places, including hospitals; study site personnel being reassigned to critical tasks.

The sponsor is providing options for study related participant management in the event of disruption to the conduct of the study. This guidance does not supersede any local or government requirements or the clinical judgement of the investigator to protect the health and well-being of participants and site staff. If, at any time, a participant's travel to the study site is considered to be dangerous, study participation may be interrupted, and study follow-up conducted. If it becomes necessary to discontinue participation in the study, the procedures outlined in the protocol for discontinuing study intervention will be followed.

If, as a result of the COVID-19 pandemic, scheduled visits cannot be conducted in person at the study site, they will be performed to the extent possible remotely/virtually or delayed until such time that on-site visits can be resumed. At each contact, participants will be interviewed to collect safety data. Key immunogenicity endpoint assessments should be performed if required and as feasible. Participants will also be questioned regarding general health status to fulfill any physical examination requirement.

Every effort should be made to adhere to protocol-specified assessments for participants on study intervention, including follow up. Modifications to protocol-required assessments may be permitted using the examples contained in this appendix, after consultation with the participant, investigator, and the sponsor. Missed assessments/visits will be captured in the clinical trial management system for protocol deviations. Discontinuations of study interventions and withdrawal from the study should be documented with the prefix "COVID-19-related" in the case report form CRF.

If a participant has tested positive for COVID-19, the investigator should contact the sponsor's responsible medical officer to discuss plans for administration of study intervention, performing study assessments, and follow-up. Modifications made to the study conduct as a result of the COVID-19 pandemic should be summarized in the clinical study report.

Guidance Specific to this Protocol

- Depending on the ongoing incidence of COVID-19 infection and subject to availability of SARS-CoV-2 point-of-care testing, participants may receive COVID-19 screening at home or clinical visits. Informed consent may be obtained remotely.
- When site visits are not possible due to local/national guidelines, sites should collect the assessments via telephone or telemedicine contact or home-based visits, if the participant allows. The actual visit date and the type of visit (ie, telephone/ telemedicine or home-based

visit) should be captured in the eCRF according to the eCRF completion guidelines. Procedures that require an on-site visit should be excluded.

- For participants with an ARI episode: nasal swabs and a sputum sample (when available) still need to meet the case definition for RSV-mediated LRTD. The ARI Day 1-2 nasal swab is a self-swab collected at home by the participant, and the ARI Day 3-5 nasal swab and sputum sample (when available) may also need to be collected at home, when site visits may not be possible.
- In the setting that on-site visits are not possible, a courier service will need to be arranged to transport the samples to the site in a timely manner.
- Questionnaires to be completed at the Day 365 visit, and additionally for participants with an ARI episode on ARI Days 3-5 and 29, will be completed by the participant in the eDiary or via a telephone or telemedicine interview with the participant, as applicable.

10.14. Appendix 14: Thrombotic Events to be Reported as Potential AESIs

At the time of protocol writing, the list of thrombotic events to be reported to the sponsor as potential AESIs is provided below. Further guidance may become available on thrombotic events of interest.

- MedDRA preferred terms (PTs) for large vessel thrombosis and embolism

  Aortic embolus, aortic thrombosis, aseptic cavernous sinus thrombosis, brain stem embolism, brain stem thrombosis, carotid arterial embolus, carotid artery thrombosis, cavernous sinus thrombosis, cerebral artery thrombosis, cerebral venous sinus thrombosis, cerebral venous thrombosis, superior sagittal sinus thrombosis, transverse sinus thrombosis, mesenteric artery embolism, mesenteric artery thrombosis, mesenteric vein thrombosis, splenic artery thrombosis, splenic embolism, splenic thrombosis, thrombosis mesenteric vessel, visceral venous thrombosis, hepatic artery embolism, hepatic artery thrombosis, hepatic vein embolism, hepatic vein thrombosis, portal vein embolism, portal vein thrombosis, portosplenomesenteric venous thrombosis, splenic vein thrombosis, spontaneous heparin-induced thrombocytopenia syndrome, femoral artery embolism, iliac artery embolism, jugular vein embolism, jugular vein thrombosis, subclavian artery embolism, subclavian vein thrombosis, obstetrical pulmonary embolism, pulmonary artery thrombosis, pulmonary thrombosis, pulmonary venous thrombosis, renal artery thrombosis, renal embolism, renal vein embolism, renal vein thrombosis, brachiocephalic vein thrombosis, vena cava embolism, vena cava thrombosis, truncus coeliacus thrombosis

- MedDRA PTs for more common thrombotic events

  Axillary vein thrombosis, deep vein thrombosis, pulmonary embolism, MedDRA PTs for acute myocardial infarction*, MedDRA PTs for stroke*

Source: Shimabukuro T. CDC COVID-19 Vaccine Task Force. Thrombosis with thrombocytopenia syndrome (TTS) following Janssen COVID-19 vaccine. Advisory Committee on Immunization Practices (ACIP). April 23, 2021. https://www.cdc.gov/vaccines/acip/meetings/slides-2021-04-23.html.

*Vaccine Adverse Event Reporting System (VAERS) Standard Operating Procedures for COVID-19 (as of 29 January 2021) https://www.cdc.gov/vaccinesafety/pdf/VAERS-v2-SOP.pdf

10.15. Appendix 15: Protocol Amendment History

The Protocol Amendment Summary of Changes Table for the current amendment is located directly before the Table of Contents (TOC).

Amendment 4 (8 April 2022)

Overall Rationale for the Amendment:

The available immunogenicity data generated in ongoing clinical studies with the Ad26/protein preF RSV vaccine suggest that immune responses after a single vaccination are durable beyond one year, and that a revaccination at Month 12 is likely not necessary for efficacy. Therefore, the second dose at Month 12 has been removed from the protocol. Changes have been made to the planned analyses including the interim analyses plan. The changes made to this clinical protocol as part of Protocol Amendment 4 are listed below, including the rationale of each change and a list of all applicable sections.

Section Number and Name	Description of Change	Brief Rationale
1.1Synopsis;<br>Figure 1: Schematic Overview of the Study for all Participants ;<br>1.3.1Schedule of Activities – Assessments for All Participants ;<br>2.1Study Rationale;<br>3OBJECTIVES AND ENDPOINTS;<br>4.1Overall Design;<br>Table 2: Study Design: VAC18193RSV3001;<br>5.1Inclusion Criteria;<br>5.2Exclusion Criteria;<br>5.5Criteria for Temporarily Delaying Administration of Study Vaccine;<br>6.3Measures to Minimize Bias: Randomization and Blinding;<br>6.8Concomitant Therapy<br>8 STUDY ASSESSMENTS AND PROCEDURES	All references to revaccination have been removed from the design and study procedures etc, including the safety follow-up (calls, visits)	Immunogenicity data from ongoing clinical studies with the Ad26/protein preF RSV vaccine suggest that the immune responses after a single vaccination are durable beyond one year, and that a revaccination at Month 12 is likely not necessary.

Section Number and Name	Description of Change	Brief Rationale
7 DISCONTINUATION OF STUDY VACCINATION AND PARTICIPANT DISCONTINUATION/WITHDRAWAL<br>7.1Discontinuation of Study Vaccination;<br>8.5.5Pregnancy	Discontinuation of study vaccination Section 7.1 is not applicable because participants will not receive a second vaccination<br>A discussion about study vaccination discontinuation in a pregnant participant was deleted.	Participants will receive only 1 dose of active vaccine/placebo
1.1Synopsis;<br>2.1Study Rationale;<br>3OBJECTIVES AND ENDPOINTS;<br>4.1Overall Design;<br>8.1ARI Assessments and Procedures;<br>8.4Safety Assessments;<br>8.5.1Time Period and Frequency for Collecting Adverse Event, Serious Adverse Event, and Adverse Event of Special Interest Information	The timing of safety assessments has been revised in several sections from "end of first RSV season" or "end of second RSV season" to time from vaccination.	To align the safety follow-up process with the removal of the second vaccination
5.1Inclusion Criteria;<br>Inclusion criterion 5	Inclusion criterion # 5 was modified to delete the note discussing the Month 12 revaccination.	Participants will receive only 1 dose of active vaccine/placebo.
5.2Exclusion Criteria;<br>Exclusion criterion 16	Exclusion criterion #16 was modified to remove reference to the full course of vaccination	Participants will receive only 1 dose of active vaccine/placebo.
5.2Exclusion Criteria;<br>Exclusion criterion 19	Exclusion criterion #19 was modified to remove reference to a subsequent vaccination	Participants will receive only 1 dose of active vaccine/placebo.
5.2Exclusion Criteria;<br>Exclusion criterion 20	Exclusion criterion #20 was modified to remove reference to "each" vaccination.	Participants will receive only 1 dose of active vaccine/placebo.
1.1Synopsis;<br>4.1Overall Design	Sample size was updated to clarify the number of participants enrolled in specific Asian countries/territories under local protocols	To clarify the number of participants for which safety data will be available
1.1Synopsis;<br>6.3Measures to Minimize Bias: Randomization and Blinding;<br>9.4.1Efficacy Analysis;<br>9.4.2Immunogenicity Analyses;<br>9.5Planned Analyses	The planned analyses and randomization sections were updated to reflect the lack of a second vaccination.<br>Revisions to planned analyses, including the interim analyses were made.	Statistical analyses were updated to reflect revision to objectives/endpoints/hypothesis, to align with health authority feedback and program needs.

Section Number and Name	Description of Change	Brief Rationale
Figure 3: Interim and Primary Analysis Plan		
1.1Synopsis;<br>1.2Schema;<br>2.1Study Rationale;<br>4.1Overall Design;<br>8.1 ARI Assessments and Procedures;<br>9STATISTICAL CONSIDERATIONS	The study duration was increased to 24 months from date of vaccination (Day 1).	To allow a full 2-year follow-up of all participants
1.3.2Schedule of Activities – Assessments for Participants with an ARI Episode;<br>4.1Overall Design;<br>8.1ARI Assessments and Procedures	Information from a protocol clarification communication (PCC) was included on the blood sample collection and Day 3-5 ARI visit.<br>PCC text:<br>To reduce the burden on the participant and to avoid collection of blood samples (ie, serology blood sample, whole blood PAXgene sample and whole blood sample with PROT1 stabilizer), not related to an RSV/hMPV/influenza related ARI, participants and investigators can choose the preferred option during each Day 3-5 visit between:<br>1) Collecting blood samples before the nasal swab samples are tested or in the setting where a sputum sample is collected independent of the RT-PCR results;<br>2) Collecting blood samples after the nasal swab RT-PCR results are available, according to nasal swab test results, and for ARIs where there is no sputum sample collection. If this is the preferred option by the participant and investigator, the participant will need to wait at the site for the time needed to test the swab samples (estimated time: 2 hours). In this scenario, if the swab samples are negative for RSV, hMPV and influenza AND if no sputum sample can be collected, the blood samples collection at the ARI Day	To reduce the burden on the participant during an ARI

Section Number and Name	Description of Change	Brief Rationale
	3-5 timepoint can be omitted. If the swab samples are positive for RSV, hMPV or influenza OR if a sputum sample is collected, the blood samples collection has to be performed.<br><br>Whenever a sputum sample is collected during the ARI Day 3-5 visit, the blood sample must still be collected as the RT-PCR test on the sputum sample will be performed at the central lab and not on site.<br><br>Also, note that a split ARI Day 3-5 visit is not allowed.	
1.1Synopsis;<br><br>4.1Overall Design;<br><br>8.2.4Diagnosis of RSV and Other Respiratory Infections;<br><br>Figure 2: Schematic Overview of the ARI Assessments and Procedures	RT-PCR test results related to the detection of respiratory pathogens other than RSV, hMPV and influenza can be communicated to participants	Clarification that RT-PCR results related to the detection of other respiratory pathogens can be communicated to participants
1.3.1Schedule of Activities – Assessments for All Participants;<br><br>8STUDY ASSESSMENTS AND PROCEDURES	Total blood volumes have been reduced because the second vaccination will not be administered.	Blood draws associated with the second vaccination will not be collected.
2.3.1Risks of Study Participation	The risks related to Ad26/protein preF RSV vaccine have been updated	Based on ongoing clinical trial data, the risks to the active vaccine have been updated.
Throughout protocol	Wording revisions to references and procedures related to a second vaccination and to references related to RSV season.<br><br>Minor typographical corrections.	Clarification
Throughout protocol	Language was updated from “should” to “must” in applicable sections to reflect updated template language	Update to protocol template language

Amendment 3 (10 November 2021)

Overall Rationale for the Amendment: This amendment is made to align the protocol following comments from CBER that all cases meeting the programmed case definition of RT-PCR confirmed RSV lower respiratory tract disease (LRTD) should be reviewed by the Clinical Event Adjudication Committee (CEC). Additionally, to increase the probability of having a primary analysis at the end of the first RSV season, an interim analysis by the IDMC has been added if the number of observed RSV cases is ≥24 to

<62. If the interim analysis by the IDMC has positive results, then the study will be unblinded and the primary analysis will be performed by the sponsor. The changes made to this clinical protocol as part of Protocol Amendment 3 are listed below, including the rationale of each change and a list of all applicable sections.

**Section Number and Name**	**Description of Change**	**Brief Rationale**
1.1 Synopsis; 4.1 Overall Design; 8.2.1 Case Definition for RT-PCR-confirmed RSV-mediated LRTD; 9.4.1.4 Exploratory Endpoints; 9.6.2 Clinical Event Adjudication Committee (new section); 10.2.6.2 Clinical Event Adjudication Committee (new section)	The Clinical Event Adjudication Committee (CEC) will review all available data of all RSV-mediated acute respiratory infections (ARIs) and will confirm if the RT-PCR-confirmed RSV-mediated LRTD definition criteria were met. Further for all RT-PCR-confirmed RSV LRTD cases the CEC will assess their severity (mild, moderate or severe).	To use the outcome of the adjudication performed by the CEC on each protocol-defined RSV-positive ARI for primary and secondary endpoints related to LRTD and to have an independent expert clinical assessment on the severity of each RSV-positive LRTD case for the exploratory related endpoint.
1.1 Synopsis; 2.1 Study Rationale; 3 OBJECTIVES AND ENDPOINTS; 9.1 Statistical Hypotheses; 9.4.1.1 Primary Endpoint; 9.4.1.2 Secondary Efficacy Endpoints; 9.4.1.3 Testing Strategy for Primary and Confirmatory Secondary Endpoints; 9.4.1.4 Exploratory Endpoints; 9.5.1 Analysis Plan for the Interim and Primary Analyses; 9.6.1 Independent Data Monitoring Committee; 10.2.6.1 Independent Data Monitoring Committee	An interim analysis (IA) at the end of the RSV season was added. An IA will be performed by the IDMC if the number of primary endpoint events observed is ≥24 and <62. If the interim analysis by the IDMC has positive results, then the study will be unblinded and the primary analysis will be performed by the sponsor. If ≥62 primary endpoint events are observed, then the primary analysis (PA) will be performed by the sponsor. The testing strategy was updated accordingly. There will be at most one IA by the IDMC.	To increase the probability of having a primary analysis at the end of the first NH RSV season, in the event of an atypical RSV season.
6.3 Measures to Minimize Bias: Randomization and Blinding; 9.5.2 Year 2 Analysis (new section); 9.5.3 End-of-Study Analysis	For a NH + SH study, a Year 2 analysis is added prior to the final analysis. If the PA occurs prior to or at the end of the second NH RSV season, the Year 2 analysis will be performed at the end of the second NH RSV season. The second SH RSV season will be considered supportive. If the PA occurs only at the end of the second SH RSV season, then the Year 2 analysis will coincide with the final analysis.	To increase the likelihood to have key confirmatory secondary endpoints available at the end of the second NH RSV season.
1.1 Synopsis; 3 OBJECTIVES AND ENDPOINTS; 9.4.1.4 Exploratory Endpoints	An objective was added to explore the relative VE against RT-PCR confirmed RSV-mediated LRTD during the second RSV season and over the whole study between the group with active vaccination at Day 1 and Day 365 and the group with active vaccination at Day 1.	To comply with regulatory authority recommendations.

**Section Number and Name**	**Description of Change**	**Brief Rationale**
2.1 Study Rationale; 6.3 Measures to Minimize Bias: Randomization and Blinding; 9.2.2 Immunogenicity; 9.2.3 Safety	Removal of reference to NH only scenario.	The study will be recruited in the NH + SH.
1.3.1 Schedule of Activities – Assessments for All Participants; 9.5.1 Analysis Plan for the Interim and Primary Analyses, Footnote to Figure 3	The end of RSV season dates were shifted by 2 weeks earlier.	There are indications of shifting seasonality.
1.1 Synopsis; 1.3.2 Schedule of Activities – Assessments for Participants with an ARI Episode; 4.1 Overall Design; 8 STUDY ASSESSMENTS AND PROCEDURES; 8.1 ARI Assessments and Procedures	Additional instructions were provided with regard to ARI Day 3-5 sample collection in case of first positivity for SARS-CoV-2 infection during ARI Day 1-2 and subsequent self-isolation. For these cases, 2 swab kits will be provided to the participant.	Because of local regulations, participants whose standard-of-care testing is positive for SARS-CoV-2 at ARI Day 1-2 will not come to the site for the ARI Day 3-5 visit. Therefore, additional kit material is provided to self-collect the ARI Day 3-5 swab.
1.1 Synopsis; 8.1 ARI Assessments and Procedures; 8.2 Efficacy Assessments; 8.2.4 Diagnosis of RSV and Other Respiratory Infections	Clarification on central testing, in case BioFire Filmarray Respiratory panel unavailability in the country or at the site.	Due to logistic reasons, it may be that the BioFire Filmarray Respiratory panel will not be available in some countries or sites. This change introduces the possibility to perform these tests centrally, in this circumstance.
2.3.1 Risks of Study Participation; 8.5.5 Pregnancy	In case of pregnancy, postnatal sequelae in the infant will not be followed-up.<br>Note: Follow up info regarding the outcome of the pregnancy of the study participant themselves or of the partner of the participant will be requested (upon consent).	According to the current Janssen SOP 'Receipt, Follow-up, and Reporting of Janssen Medicinal Product Individual Case Safety Reports', follow-up of postnatal sequelae in the infant is not a requirement.<br>The sponsor protocol template is planned to be modified to reflect this change.
5.1 Inclusion Criteria, Criterion #5	Footnote for Inclusion Criterion 5 was updated to allow participants with values for vital signs measurements ≤Grade 3 (other than temperature) to be enrolled in the study, if the values are deemed not clinically significant by the investigator.	To clarify that participants with a Grade 3 vital sign abnormality at screening may also be eligible for the study.
5.2 Exclusion Criteria, Criterion #11	Footnote for Exclusion Criterion 11 was updated to allow participants who participate in the observational phase of interventional studies to be enrolled in the study, if approved by the sponsor or its delegate.	To clarify that participants who participate in the observational phase of an interventional study at screening may also be eligible for the study.

Section Number and Name	Description of Change	Brief Rationale
1.3.1 Schedule of Activities – Assessments for All Participants	Update of the relevant medical history to be reported including thrombotic events and/or thrombocytopenia.	To facilitate the assessment of adverse events of special interest (AESIs) reported during the study.
8.5.1 Time Period and Frequency for Collecting Adverse Event, Serious Adverse Event, and Adverse Event of Special Interest Information; 8.5.6.1 Thrombosis with Thrombocytopenia Syndrome	To remove the word "Symptomatic" from "Symptomatic Thrombocytopenia".	The definition of thrombocytopenia was broadened to include all cases of thrombocytopenia the investigators observe (including participant's routine blood test or blood test performed concomitantly to any AE/SAE/ hospitalization, or any other) in the period of 6 months after vaccination; these should be reported as AESIs.
1.3.1 Schedule of Activities – Assessments for All Participants – footnote z; 8.5.6.1 Thrombosis with Thrombocytopenia Syndrome	Additional language referring to laboratory diagnostic tests for the assessment of potential AESIs is added.	To facilitate and simplify the local site management of potential AESIs.
5 STUDY POPULATION; 1.3.1 Schedule of Activities – Assessments for All Participants	To clarify that in case of a split visit, efforts should be made that the visits occur preferably within 3 to 5 days but no later than 14 days apart.	To ensure that the health conditions of the potential participant are unchanged during the split visit window.
1.3.1 Schedule of Activities – Assessments for All Participants; 8 STUDY ASSESSMENTS AND PROCEDURES – Visit Windows.	Clarification that efforts should be made so that revaccination occurs not earlier than mid-June 2022 for the NH countries, and not earlier than November 2022 for the SH countries, and not later than the expected peak of the RSV season in both NH and SH countries, respectively. In addition, for each participant there should be a minimum of 6 months between the first and the second vaccination.	To increase the likelihood that participants are revaccinated prior to or around the start of the season during the second year given the unpredictability of the RSV season.
Throughout the protocol	Minor clarifications, grammatical, formatting, or spelling changes were made.	Minor errors were noted.

Amendment 2 (19 July 2021)

Overall Rationale for the Amendment: This protocol has been created to align the information and guidance for investigators on signs and symptoms and on medical management of thrombosis with thrombocytopenia syndrome (TTS) events across the VAC18193 program.

Section Number and Name	Description of Change	Brief Rationale
1.1 Synopsis<br>1.3.1 Schedule of Activities – Assessments for All Participants<br>4.1 Overall Design<br>6.7 Overdose<br>6.8 Concomitant Therapy<br>7.1 Discontinuation of Study Vaccination<br>8.4 Safety Assessments<br>8.5.6 Adverse Events of Special Interest<br>9.2.3 Safety<br>10.1 Appendix 1: Abbreviations<br>10.14 Appendix 14: Thrombotic Events to be Reported as Potential AESIs<br>11 REFERENCES	To rename the immediately reportable events (IREs) to (potential) adverse events of special interest (AESIs) and to update the case definition including potential cases and symptomatic thrombocytopenia.	To align the wording on information and guidance for investigators on signs and symptoms and on medical management of TTS across the RSV vaccine (VAC18193) program.
1.1 Synopsis<br>1.3.1 Schedule of Activities – Assessments for All Participants<br>2.2 Background<br>2.3.3 Benefit-Risk Assessment of Study Participation<br>5.2 Exclusion Criteria<br>8.4 Safety Assessments<br>8.5 Adverse Events, Serious Adverse Events, Adverse Events of Special Interest, and Other Safety Reporting<br>8.5.1 Time Period and Frequency for Collecting Adverse Event, Serious Adverse Event, and Adverse Event of Special Interest Information<br>8.5.2 Method of Detecting Adverse Events, Serious Adverse Events, and Adverse Events of Special Interest<br>8.5.3 Follow-up of Adverse Events, Serious Adverse Events, and Adverse Events of Special Interest<br>8.5.6 Adverse Events of Special Interest<br>8.5.6.1 Thrombosis with Thrombocytopenia Syndrome<br>10.1 Appendix 1: Abbreviations<br>10.2.6 Committees Structure<br>10.3 Appendix 3: Adverse Events, Serious Adverse Events, Adverse Event of Special Interest, Product Quality Complaints, and Other Safety Reporting: Definitions and	To add AdVac safety wording and to update and wording on information and guidance for investigators on signs and symptoms and on medical management of thrombosis with thrombocytopenia syndrome (TTS).	

Section Number and Name	Description of Change	Brief Rationale
Procedures for Recording, Evaluating, Follow-up, and Reporting<br>10.3.5 Procedures<br>10.14 Appendix 14<br>11 REFERENCES		
1.1 Synopsis<br>1.3.2 Schedule of Activities – Assessments for Participants with an ARI Episode<br>4.1 Overall Design<br>8.1 ARI Assessments and Procedures<br>8.2 Efficacy Assessments<br>8.2.4 Diagnosis of RSV and Other Respiratory Infections<br>8.6 Medical Resource Utilization and Health Economics	To change the BioFire Filmarray RP2.1-EZ panel to the BioFire Filmarray Respiratory panel.	To generalize the type of BioFire Filmarray that is used to test for RSV, hMPV, and influenza.
1.1 Synopsis<br>1.3.2 Schedule of Activities – Assessments for Participants with an ARI Episode<br>4.1 Overall Design<br>8.1 ARI Assessments and Procedures<br>9.4.1.1 Primary Endpoint	To change "Participants with a RT-PCR-confirmed…" to "Participants with an ARI and a RT-PCR positive for…"	To clarify the testing of participants who have an ARI and have a RT-PCR that is positive for RSV, hMPV or influenza.
1.1 Synopsis<br>3 OBJECTIVES AND ENDPOINTS<br>9.4.1.4 Exploratory Endpoints	Minor changes to the planned analyses.	To align with the statistical Analysis plan (SAP).
1.1 Synopsis<br>1.3.2 Schedule of Activities – Assessments for Participants with an ARI Episode<br>4.1 Overall Design<br>8.2.4 Diagnosis of RSV and Other Respiratory Infections	To update footnote b in the Schedule of Activities – assessments for participants with an ARI Episode and to provide more guidance on the disclosure of RT-PCR results to the participants in the study.	To clarify that the RT-PCR results for RSV, hMPV, and influenza are not shared with the participant.
1.1 Synopsis<br>1.3.2 Schedule of Activities – Assessments for Participants with an ARI Episode<br>4.1 Overall Design<br>8.1 ARI Assessments and Procedures<br>8.2.4 Diagnosis of RSV and Other Respiratory Infections	Participants who have a positive SARS-CoV-2 RT-PCR test as part of the study procedures should be referred to their own healthcare provider for a locally approved diagnostic test and further management, in accordance with local/site specific guidelines.	To clarify the study procedures for participants who have a positive SARS-CoV-2 RT-PCR test.
1.1 Synopsis<br>8.3 Immunogenicity Assessments	Added further details of assays that will be used for assessment of RSV cross-neutralization.	To clarify assays in scope for the corresponding exploratory endpoint.
1.1 Synopsis<br>8.3 Immunogenicity Assessments	Blood samples from all participants for immunogenicity are collected for the analysis of correlates of risk of RSV disease and correlates of protection.	To specify the purpose of the immunogenicity samples.

Section Number and Name	Description of Change	Brief Rationale
Throughout the protocol	Minor grammatical, formatting, or spelling changes were made.	Minor errors were noted.

Amendment 1 (3 May 2021)

Overall Rationale for the Amendment: Several changes are made to follow recommendations from regulatory authorities (eg, update of the primary hypothesis of vaccine efficacy against RT-PCR- confirmed RSV mediated lower respiratory tract disease (LRTD), stratification and minimization factors for the randomization, secondary endpoints testing strategy).

Section Number and Name	Description of Change	Brief Rationale
1.1 Synopsis;<br>1.3.2 Schedule of Activities– Assessments for Participants with an ARI Episode;<br>4.1 Overall Design;<br>8.1 ARI Assessments and Procedures;<br>8.2 Efficacy Assessments	Sputum testing will be performed at the central laboratory; in those cases (when sputum is collected and sent to the central laboratory), the ARI episode will be followed up independent of the RT-PCR results from the nasal swabs.	To ensure that all sputum samples collected during ARI episodes will be tested in a CLIA-certified laboratory.
1.1 Synopsis;<br>1.3.1 Schedule of Activities – Assessments for All Participants;<br>3 OBJECTIVES AND ENDPOINTS;<br>4.1 Overall Design;<br>6.7 Overdose;<br>6.8 Concomitant Therapy;<br>7.1 Discontinuation of Study Vaccination;<br>8.4 Safety Assessments;<br>8.5.6 Immediately Reportable Events;<br>9.4.3 Safety Analyses;<br>Appendix 14: Immediately Reportable Events	The Schedule of Activities and the text were updated to include wording about collection of immediately reportable events (IREs).	The reporting of IREs was implemented to ensure complete information will be available for all thrombotic events allowing evaluation of those events.
1.1 Synopsis;<br>1.3.1 Schedule of Activities – Assessments for All Participants;<br>8.4 Safety Assessments;<br>8.5.1 Time Period and Frequency for Collecting Adverse Event and Serious Adverse Event Information;<br>9.4.3 Safety Analyses	The Schedule of Activities and the text were updated to include wording about collection of all events of COVID-19 infection.	The reporting of any severity COVID 19 cases was implemented to ensure complete information will be available.

Section Number and Name	Description of Change	Brief Rationale
1.1 Synopsis;<br>1.3.2 Schedule of Activities– Assessments for Participants with an ARI Episode;<br>4.1 Overall Design;<br>8.1 ARI Assessments and Procedures; Appendix 9	Full RiiQ (Appendix 9) collection was substituted by the RiiQ Symptom Scale and the RiiQ Impact on Daily Activities Scale (Appendix 8). Appendix 9 was deleted.	To comply with regulatory authorities recommendations and to have a more specific and simplified PRO collection during ARI episode.
1.1 Synopsis;<br>3 OBJECTIVES AND ENDPOINTS, Secondary Endpoints	The secondary endpoint comparing the area under the curve of the change from baseline in the RiiQ Total Symptom score in participants with an RT-PCR-confirmed RSV-mediated ARI over the whole study was moved to be a non-confirmatory secondary endpoint.	To comply with regulatory authority recommendations.
1.1 Synopsis;<br>4.1 Overall Design;<br>8.1 ARI Assessments and Procedures	Footnote was added to clarify the process to confirm a potential ARI episode.	To clarify the process to confirm a potential ARI episode.
1.1 Synopsis;<br>4.1 Overall Design;<br>8.1 ARI Assessments and Procedures	Specified that during an ARI episode, in the event of concomitant positivity to RSV (or hMPV or influenza) and SARS-Cov-2, the participants should keep on filling in the ARI questionnaire during the entire ARI episode.	The process to stop answer ARI related questionnaires in the eDiary by the participant during an ARI episode was clarified.
1.1 Synopsis;<br>4.1 Overall Design;<br>9.2 Sample Size Determination	Specified that the sponsor is monitoring the RSV incidence across the countries involved. If the collected information combined with ongoing datascience and modeling activities indicate that the incidence assumptions can be increased, the sponsor can decrease the sample size during the recruitment of the study.	To allow sample size decrease based on ongoing datascience and modeling activities.
1.1 Synopsis;<br>4.2 Scientific Rationale for Study Design;<br>8.7 Participant Medical Information Prior to, During and After the Study (Real-world Data)	Wording was updated to clarify that, even though consent is optional, real-world data should be considered as part of the study data.	To align all sections of the protocol with the exploratory study endpoint "Utilization of tokenization and matching procedures for exploratory analysis of participant's medical data prior to, during, and following participation in the study (real-world data). Analysis will be performed to relate real-world data to vaccine immune responses, efficacy and duration of protection, and AEs".
1.1 Synopsis;<br>8.1 ARI Assessments and Procedures	If it is not possible to perform the swab testing at the site, the swab sample needs to be tested centrally and the ARI episode should be followed up until resolution of the ARI episode.	To ensure that all available samples will be tested and used for the study-related analyses, the central laboratory will function as back-up.
1.1 Synopsis;<br>8.2.2 Case Definition for RT-PCR-confirmed RSV-mediated ARI	The ARI case definition was clarified.	To comply with regulatory authority recommendations and to ensure compliance on data collection.

Section Number and Name	Description of Change	Brief Rationale
1.1 Synopsis;<br>8.2.3 Predefined Clinically Relevant Disease Associated with RT-PCR-confirmed RSV-mediated ARI	Wording was updated to clarify the definition of decreased oxygen saturation.	To avoid inconsistencies due to the use of oxygen saturation collected with devices/techniques characterized by different specifications/accuracies.
1.1 Synopsis;<br>8.3 Immunogenicity Assessments	A sentence was added specifying that the analysis is to be performed on Immuno Subset only.	To align Section 8.3 with the secondary objective "in the Immuno Subset and in subgroups of the Immuno Subset (including but not limited to participants at increased risk of severe RSV disease), to evaluate the immunogenicity of active study vaccine when compared to placebo".
1.1 Synopsis;<br>3 OBJECTIVES AND ENDPOINTS;<br>9.1 Statistical Hypotheses;<br>9.4.1.1 Primary Endpoint	The null hypothesis of LL ≤20% for RT-PCR-confirmed RSV-mediated LRTD will be tested versus the alternative hypothesis of LL >20%. The success criterion was updated to align with the updated hypotheses and additionally requires a point estimate for the vaccine efficacy (VE) of the primary endpoint >50%.	To comply with regulatory authority recommendations.
1.1 Synopsis;<br>9.2 Sample Size Determination	The sample size assumptions and power were updated to align with the new primary hypothesis.	To comply with regulatory authority recommendations.
1.3.1 Schedule of Activities – Assessments for All Participants	Table was updated to report height, weight, and smoking status.<br><br>Footnote j was updated to also include reporting of Type 2 diabetes and chronic kidney diseases (CKD) in Relevant Medical History.	To collect data to assess the potential impact of BMI, smoking status and underling relevant medical conditions on the ARI reporting.
1.3.1 Schedule of Activities – Assessments for All Participants;<br>8.4.1 Physical Examinations	Text was added to specify that at each clinical visit an abbreviated, symptom-directed physical examination will be performed if deemed necessary by the investigator.	To ensure a complete safety follow up of the participant.
2.3.1 Risks of Study Participation;<br>2.3.2 Benefits of Study Participation	Text was updated.	The risk section was updated with currently available data and focusing on Ad26/protein preF RSV vaccine.
5.2 Exclusion Criteria	In the exclusion criterion 4 the words "diabetes mellitus" were replaced by the words "type 1 diabetes".	To specify that type 1 diabetes is not an exclusion criterion.
	Per exclusion criterion 4, prior use of chronic or recurrent systemic corticosteroids is prohibited.	Text was updated to reduce the risk to enroll participants using systemic corticosteroids impacting immune response at the time of enrolment.

Section Number and Name	Description of Change	Brief Rationale
	Exclusion criterion 5 was updated and a new exclusion criterion 19 was added to cover all potential types of SARS-CoV-2 vaccines a participant could receive before or during the study.	Text was updated to provide guidance on the enrollment of participants who received SARS-CoV-2 vaccines.
	A note on monoclonal antibodies use was added to exclusion criterion 10.	Text was updated to clarify the exclusion criterion related to monoclonal antibodies administration.
	Exclusion criterion 13 was updated and criterion 20 was added to clarify the exclusion criteria related to participants who have received an Ad26-vectored vaccine.	Text was updated to o provide guidance on the enrolment of participants who received another Ad26-based vaccine.
	Exclusion criterion 14 was deleted.	The criterion was redundant as disallowed therapy before enrollment is already covered by exclusion criteria 4 and 10.
6.3 Measures to Minimize Bias: Randomization and Blinding	The text was updated to consider regions and subset as minimization criteria instead of stratification factors.	To comply with regulatory authority recommendations.
6.6 Continued Access to Study Vaccine After the End of the Study	New section.	Section was added to clarify the access to the vaccine after the end of the study.
6.8 Concomitant Therapy	Text was updated.	Section was updated for alignment with the amended exclusion criteria.
1.1 Synopsis;<br>4.1 Overall Design;<br>8.1 ARI Assessments and Procedures;<br>8.2 Efficacy Assessments;<br>9.4.1.1 Primary Endpoint	Text was updated specifying that local RT-PCR testing will be performed using the BioFire® Filmarray RP2.1-EZ panel.	To comply with regulatory authority recommendations.
	Sentence was added to clarify that central RT-PCR testing can function as a back-up option in case a local RT-PCR test cannot be performed.	To avoid missing any important information on RT- PCR testing during ARI episode.
8.1 ARI Assessments and Procedures	Specified that if ARI is ongoing at Day 29, ARI questionnaires will be triggered until the end of the ARI episode.	The process to stop answer ARI related questionnaires in the eDiary by the participant during an ARI episode was clarified.
8.1.3 Patient Global Impression Scores<br>Appendix 10: Patient Global Impression Scales	The response categories for the PGI-S were updated.	To comply with regulatory authority recommendations.
8.2.4 Diagnosis of RSV and Other Respiratory Infections	New section 8.2.4 was added.	To clarify the distribution and the purposes of site and central RT-PCR testing.
8.5.5 Pregnancy	Text was updated.	Text was updated to ensure complete safety follow up of the participants.

Section Number and Name	Description of Change	Brief Rationale
9.4.1.4 Exploratory Endpoints	The Clinical Evaluation Committee (CEC) review will assess, independently and based on CRF/eDiary data, the location of the ARI (upper or lower respiratory tract infection) and the ARI severity. Exploratory endpoints were added to explore the efficacy of active study vaccine in the prevention of an RT-PCR confirmed RSV lower respiratory infection as assessed by the CEC compared to placebo.	To comply with regulatory authority recommendations, a CEC review was added to independently assess the location of the RSV ARIs and their severity.
10.2.1 Regulatory and Ethical Considerations	New section on Protocol Clarification Communications was added.	To align with the most recent Janssen protocol template.
10.2.6 Committees Structure	The sentence "The IDMC will also formally monitor the efficacy endpoints at the timepoints specified in Section 9.5, Planned Analyses" was deleted.	To align with analysis section.
Throughout the protocol	The number of RT-PCR-confirmed RSV-mediated LRTD events was changed from 50 to 52.	The number was changed to align with the updated success criteria.
Throughout the protocol	Minor grammatical, formatting, or spelling changes were made. Parenthetical referencing was applied.	

11. REFERENCES

Adenoviral Vaccine Safety Database - Report V7.0. Janssen Vaccines & Prevention B.V. (July 2022).

American Society of Hematology (2021). COVID-19 resources. Thrombosis with Thrombocytopenia Syndrome (also termed Vaccine-induced Thrombotic Thrombocytopenia). Updated 6 May 2021. Available at: https://www.hematology.org/covid-19/vaccine-induced-immune-thrombotic-thrombocytopenia. Accessed 27 April 2021.

Belongia EA (2018), King JP, Kieke BA, et al. Clinical features, severity, and incidence of RSV illness during 12 consecutive seasons in a community cohort of adults ≥60 years old. Open Forum Infect Dis. 2018;5(12):ofy316. Published 2018 Nov 27. doi:10.1093/ofid/ofy316.

Brighton Collaboration (2021). Interim Case Definition of Thrombosis with Thrombocytopenia Syndrome (TTS). 21 April 2021. Available at: https://brightoncollaboration.us/thrombosis-with-thrombocytopenia-syndrome-interim-case-definition/. Accessed 29 April 2021.

British Society for Haematology (2021). Guidance produced from the Expert Haematology Panel (EHP) focussed on Covid-19 Vaccine induced Thrombosis and Thrombocytopenia (VITT). Available at: https://b-s-h.org.uk/media/19530/guidance-version-13-on-mngmt-of-thrombosis-with-thrombocytopenia-occurring-after-c-19-vaccine 20210407.pdf. Version 1.3; 7 April 2021. Accessed 27 April 2021.

Centers for Disease Control and Prevention (CDC) (2020). People at high risk for severe RSV infection - Respiratory Syncytial Virus Infection (RSV) (2020). Available at: https://www.cdc.gov/rsv/high-risk/index.html. Accessed 18 Feb 2021.

Centers for Disease Control and Prevention (CDC) (2021). Cases of cerebral venous sinus thrombosis with thrombocytopenia after receipt of the Johnson & Johnson COVID-19 Vaccine. 13 April 2021. Available at: https://emergency.cdc.gov/han/2021/han00442.asp. Accessed 27 April 2021.

Demets DL (1994), Lan KK. Interim analysis: The alpha spending function approach. Stat Med. 1994:13;1341-52

Falsey A (2005), Hennessey P, Formica M, Cox C, Walsh E. Respiratory syncytial virus infection in elderly and high-risk adults. N Engl J Med. 2005:352;1749-1759.

Falsey A (2000), Walsh E. Respiratory syncytial virus infection in adults. Clin Microbiol Rev. 2000:13;371-384.

Fleming D (2015), Taylor R, Lustig R, et al. Modelling estimates of the burden of respiratory syncytial virus infection in adults and the elderly in the United Kingdom. BMC Infect Dis. 2015;15:443.

Gidudu J (2012), Walco G, Taddio A, et al. The Brighton Immunization Site Pain Working Group. Immunization site pain: Case definition and guidelines for collection, analysis, and presentation of immunization safety data. Vaccine. 2012:30;4558-4577.

Hardelid P (2013), Pebody R, Andrews N. Mortality caused by influenza and respiratory syncytial virus by age group in England and Wales 1999-2010. Influenza Other Respir Viruses. 2013;7(1):35-45.

Htar MTT (2020), Yerramalla MS, Moïsi JC, and Swerdlow DL. The burden of respiratory syncytial virus in adults: a systematic review and meta-analysis. Epidemiol Infect. 2020;148:e48.

Jansen A (2007), Sanders E, Hoes A, et al. Influenza- and respiratory syncytial virus-associated mortality and hospitalisations. Eur Respir J. 2007;30(6):1158-1166.

Jennison C (2000) and Turnbull BW. Group Sequential Methods with Applications to Clinical Trials. Boca Raton: Chapman & Hall; 2000. Chapter 7, Flexible Monitoring: The Error Spending Approach.

Kohl K (2007), Walop W, Gidudu J, et al. The Brighton Collaboration Local Reaction Working Group for Swelling at or Near Injection Site. Swelling at or near injection site: Case definition and guidelines for collection, analysis and presentation of immunization safety data. Vaccine. 2007:31;5858-5874.

Marcy S (2004), Kohl K, Dagan R, et al. The Brighton Collaboration Fever Working Group. Fever as an adverse event following immunization: case definition and guidelines of data collection, analysis, and presentation. Vaccine. 2004:22;551-556.

Osborne R (2011), Norquist J, Elsworth G, et al. Development and validation of the Influenza Intensity and Impact Questionnaire (FluiiQ™). Value in Health. 2011;14:687-699.

Thompson W (2003), Shay D, Weintraub E, et al. Mortality associated with influenza and respiratory syncytial virus in the United States. JAMA. 2003:289;179-186.

U.S. Department of Health and Human Services, Office for Human Research Protections. March 2016. OHRP Expedited Review Categories (1998). Available at: http://www.hhs.gov/ohrp/regulations-and-policy/guidance/categories-of-research-expedited-review-procedure-1998/index.html. Accessed 26 January 2021.

US Food and Drug Administration. Conditions for IRB Use of Expedited Review. Federal Register: November 9, 1998 (Volume 63, Number 216). Available at: https://www.hhs.gov/ohrp/news/federal-register-notices/federal-register-11-09-1998-vol-63-no-216/index.html.

van Asten L (2012), van den Wijngaard C, van Pelt W, et al. Mortality attributable to 9 common infections: significant effect of influenza A, respiratory syncytial virus, influenza B, norovirus, and parainfluenza in elderly persons. J Infect Dis. 2012;206(5):628-639.

van Essen G (2014), Beran J, Devaster J, et al. Influenza symptoms and their impact on elderly adults: randomized trial of AS03-adjuvanted or non-adjuvanted inactivated trivalent season influenza vaccines. Influenza Other Respir Viruses. 2014;8:452-462.

INVESTIGATOR AGREEMENT

I have read this protocol and agree that it contains all necessary details for carrying out this study. I will conduct the study as outlined herein and will complete the study within the time designated.

I will provide copies of the protocol and all pertinent information to all individuals responsible to me who assist in the conduct of this study. I will discuss this material with them to ensure that they are fully informed regarding the study intervention, the conduct of the study, and the obligations of confidentiality.

Coordinating Investigator (where required):

Name (typed or printed): ______________________________

Institution and Address: ______________________________

____

____

____

Signature: ______________________________ Date: ______________

(Day Month Year)

Principal (Site) Investigator:

Name (typed or printed): ______________________________

Institution and Address: ______________________________

____

____

____

Telephone Number: ______________________________

Signature: ______________________________ Date: ______________

(Day Month Year)

Sponsor's Responsible Medical Officer:

Name (typed or printed): PPD

Institution: Janssen Vaccines & Prevention B.V.

Signature: electronic signature appended at the end of the protocol Date: ______________

(Day Month Year)

Note: If the address or telephone number of the investigator changes during the course of the study, written notification will be provided by the investigator to the sponsor, and a protocol amendment will not be required.

Signature

User	Date	Reason
PPD	12-Oct-2022 11:08:28 (GMT)	Document Approval
