# Supplementary material for: A randomised, double-blind, multicentre, placebo-controlled phase 3 efficacy, safety, and immunogenicity study of an Ad26.RSV.preF/RSV prefusion F-protein vaccine for the prevention of respiratory syncytial virus-mediated lower respiratory tract disease in adults aged 60 years and older
Source: eBioMedicine. 2026 Nov 4;133:106513. doi: 10.1016/j.ebiom.2026.106513 (PMC13760371; doi:10.1016/j.ebiom.2026.106513)
Supplement: Supplementary file 2 [file mmc2.pdf]

Janssen Vaccines & Prevention B.V.*

Statistical Analysis Plan

A Randomized, Double-blind, Placebo-controlled Phase 3 Efficacy Study of an Ad26.RSV.preF-based Vaccine in the Prevention of Lower Respiratory Tract Disease Caused by RSV in Adults Aged 60 Years and Older

Protocol VAC18193RSV3001; Phase 3
EVERGREEN
AMENDMENT 3

VAC18193 (JNJ-64400141/JNJ-64213175)

* Janssen Vaccines & Prevention B.V. is a Janssen pharmaceutical company of Johnson & Johnson and is hereafter referred to as the sponsor of the study. The sponsor is identified on the Contact Information page that accompanies the protocol

Status: Approved
Date: 5 June 2023
Prepared by: Janssen Vaccines & Prevention B.V
Document No.: EDMS-RIM-395835, 4.0

Compliance: The study described in this report was performed according to the principles of Good Clinical Practice (GCP).

Confidentiality Statement
The information provided herein contains Company trade secrets, commercial or financial information that the Company customarily holds close and treats as confidential. The information is being provided under the assurance that the recipient will maintain the confidentiality of the information under applicable statutes, regulations, rules, protective orders or otherwise.

TABLE OF CONTENTS

TABLE OF CONTENTS 2

LIST OF IN-TEXT TABLES AND FIGURES 4

AMENDMENT HISTORY 5

1. INTRODUCTION 7
1.1. Objectives and Endpoints 7
1.2. Study Design 7

2. STATISTICAL HYPOTHESES 8

3. SAMPLE SIZE DETERMINATION 9

4. POPULATIONS (ANALYSIS SETS) FOR ANALYSIS 9

5. PLANNED ANALYSIS 10
5.1. Analysis Plan for the Interim and Primary Analysis 10
5.2. Additional Analyses 12
5.3. End-of-Study Analysis 12

6. STATISTICAL ANALYSES 13
6.1. General Considerations 13
6.1.1. Study Phases 13
6.1.2. Phase Definitions 13
6.1.3. Immunogenicity Visit Windows 15
6.2. Participant Dispositions 16
6.3. Primary Endpoint Analysis 17
6.3.1. Definition of Endpoint 17
6.3.2. Level of Significance 21
6.3.3. Analysis Methods 21
6.4. Secondary Endpoints Analysis 24
6.4.1. Key Confirmatory Secondary Endpoints 24
6.4.1.1. Definition of Endpoints 25
6.4.1.1.1. Case Definition for RT-PCR-confirmed RSV-mediated ARI 25
6.4.1.1.2. Predefined Clinically Relevant Disease Associated with RT-PCR-confirmed RSV-mediated ARI 25
6.4.1.2. Level of Significance 26
6.4.1.2.1. Analysis Methods 27
6.4.2. Supportive (Non-Confirmatory) Secondary Endpoint 27
6.4.2.1. Analysis Methods 27
6.5. Exploratory Endpoints Analysis 28
6.5.1. Analysis Methods 30
6.6. Other Exploratory Analysis 31
6.6.1. ARI Duration 31
6.6.2. ARI Symptoms 31
6.6.3. ARI Complications 31
6.6.4. Clinically Relevant Disease 32
6.6.5. Vital Signs During the ARI episodes 32
6.6.6. RSV Viral Load. 32
6.6.7. Patient Reported outcomes 33
6.6.7.1. Respiratory Infection Intensity and Impact Questionnaire (RiiQ™v2) 33
6.6.7.1.1. Analysis Methods 34
6.6.7.2. Patient Global Impression Scores 34
6.6.7.2.1. Analysis Methods 35
6.6.7.3. EuroQoL, 5-Dimension, 5-Level Questionnaire 36

6.6.7.3.1. Analysis Methods 37
6.6.7.4. Days missed from work 38
6.6.8. Medical Resources Use Questionnaire 38
6.6.9. Approximate ARI Surveillance Compliance 38
6.6.10. ARI Missing Data 40
6.7. Safety Analyses 41
6.7.1. Adverse Events 41
6.7.1.1.1. Definitions 41
6.7.1.1.2. Analysis of Adverse Events 42
6.7.1.1.3. Phase Allocation of Adverse Events 43
6.7.1.1.4. Missing Data 45
6.7.2. Vital Signs 45
6.8. Other Analyses 45
6.8.1. Immunogenicity 45
6.8.1.1. Parameters 45
6.8.1.2. Handling of Missing and/or Unquantifiable Immune Response Data 46
6.8.1.3. Immune Response Analysis 46
6.8.1.4. Immunogenicity Against the Insert: 46
6.8.1.4.1. Humoral assays 46
6.8.1.4.2. Cellular assays 47
6.8.1.5. Immunogenicity Against the Vector 48
6.8.2. Definition of Subgroups 48
6.9. Interim Analyses 49
6.9.1. Data Monitoring Committee (DMC) 49
6.10. Additional Analyses 50
6.10.1. Efficacy Analyses 50
6.10.2. Safety Analyses 51
6.10.3. Demographics 53
6.11. Discontinuation of the study 53

7. SUPPORTING DOCUMENTATION 54
7.1. Appendix 1 List of Abbreviations 54
7.2. Appendix 2 Changes to Protocol-Planned Analyses 55
7.3. Appendix 3 Demographics and Baseline Characteristics 56
7.4. Appendix 4 Protocol Deviations 57
7.5. Appendix 5 Prior and Concomitant Medications 58
7.6. Appendix 6 Guidance for Industry: Toxicity Grading Scale for Healthy Adult and Adolescent Volunteers Enrolled in Preventive Vaccine Clinical Trials 60
7.7. Appendix 7 Respiratory Viruses and Bacteria Used to Define Coinfections 61
7.8. Appendix 8 Clinically Relevant Complication AE terms 63
7.8.1. Update MEDRA 24.1 to MEDRA 25.1 78
7.9. Appendix 9 Area Under the Curve Calculation 79
7.10. Appendix 10 Therapeutic Interventions of Interest 80
7.11. Appendix 11 Binomial Exact Test 84
7.12. Appendix 12 EQ-5D Valuation index 85
7.13. Appendix 13 Chronic Kidney Disease [CKD] + Diabetes Terms 86
7.14. Appendix 14 List of preferred terms for VTE (broad) 89

8. REFERENCES 92

LIST OF IN-TEXT TABLES AND FIGURES

TABLES

Table 1: SAP Version History Summary 5
Table 2: Study Design 8
Table 3: Analysis Sets 9
Table 4: Phase Definitions 13
Table 5: Interval Definition for potential AESI analysis 14
Table 6: Immunogenicity timepoints 15
Table 7: Immunogenicity timepoints during an ARI 16
Table 8: Adjudicator's choices and selection for the primary endpoint 20
Table 9: Alpha spending at the Prespecified Analysis Timepoints 21
Table 10: Secondary Endpoints: Overview of the Testing Order, Timing of the Testing in the Testing Strategy and the Corresponding Alpha 26
Table 11: Example for 'Return to Usual Health' question 35
Table 12: Case Definitions 50
Table 13: Grades for erythema and swelling based on diameter 51
Table 14: Grades for pyrexia based on temperatures 52
Table 15: Adverse Events of (clinical) interest 52
Table 16: Demographic Variables 56

FIGURES

Figure 1: Interim and Primary Analysis Plan 11

AMENDMENT HISTORY

Table 1: SAP Version History Summary

SAP Version	Approval Date	Change	Rationale
1.0	6 July 2021	Not Applicable	Initial release
2.0	19 November 2021	The amendment is made to align with changes in the Protocol amendment 3	Amendment of the CTP
3.0	23 September 2022	The amendment of the SAP is made to align with the protocol amendment 4. Additionally, the following were added:<br>• Clarification on the way potential AESI will be analyzed.<br>• Clarification on the primary analysis definition and the options provided to the Clinical Evaluation Committee<br>• Clarification on the timing of the end-of-study analysis and the population used<br>• Clarification on how to handle missing RSV ARI end dates close to the database lock.<br>• The method used for local nasal swab testing for China sites.<br>• New exploratory endpoints related to clinically relevant diseases and EQ-5D<br>Moreover:<br>• appendix 8 was updated, where AE terms were removed<br>• three ATC terms were added in appendix 10	Amendment of the CTP

4.0	05/06/2023	• Information regarding the discontinuation of the study was added in section 6.11<br><br>• Additional analyses were added in section 6.10.<br><br>• Small updates were made for aligning the definition of the end of the first year and the start of the second year between safety and efficacy. Day 365 is part of the second year. And second year starts at the study Day 365 visit, or relative day 365, in case Day 365 visit not available but there are ARI surveillance questions available after the relative day 365.<br><br>• Imputation was added for humoral assays that have both LLOQ and LOD reported.<br><br>• New PTs are added due to update of MEDRA 25.1	The main rationales for this update are:<br>• To include information regarding the discontinuation of the study<br>• to clarify the season 2 start date<br>• to include new safety analyses after FDA request.

1. INTRODUCTION

This is the Statistical Analysis Plan (SAP) applicable for the VAC18193RSV3001 study. This SAP is applicable for all analyses described in the clinical trial protocol (CTP) under Section 9.4 and the IDMC safety updates. Note that the correlate of protection and the real-world data (tokenization) analysis will be described in separate SAPs. Exploratory assessment of pre-exposure with Ad26.COV2.S Vaccine on the Immune Response to Ad26/protein preF RSV is also described in separate SAP.

This document contains all information needed for performing a full efficacy, safety, and immunogenicity analysis. The tables, listings, and figures (TLF) required for each analysis will be described in separate data presentation specifications (DPS) documents.

1.1. Objectives and Endpoints

Please refer to Section 3 in the CTP.

1.2. Study Design

This is a multicenter, randomized, double-blind, placebo-controlled Phase 3 confirmatory efficacy study in male and female participants aged 60 years and older. The primary objective of the study is to further establish the efficacy of the active Ad26.RSV.preF-based study vaccine in the prevention of RT-PCR-confirmed RSV-mediated LRTD.

Up to approximately 27,500 participants (with up to 23,000 in the Global cohort, up to approximately 4,500 participants in specific Asian countries under local protocols) will be enrolled and randomized in parallel in a 2:1 ratio to 1 of 2 groups to receive active study vaccine or placebo.

Note that some additional local cohort enrollment, beyond the global cohort enrollment may be allowed if required by local health authorities for the purpose of local health authority consideration.

- The Global cohort is defined as all participants (including the Safety subset and the Immuno subset) who are recruited up to the point that global enrollment is stopped.
- Local cohorts are defined as participants from the considered country/region and under local protocols.

For analyses purposes, data from the Global cohort and the local cohorts may be aggregated.

The study design is depicted in the table below.

Table 2: Study Design

Group	N[a]	Day 1
1	15,340	Ad26/protein preF RSV vaccine (1×10^{11} vp/150 µg)
2	7,670	Placebo

[a] The study will be discontinued if the primary analysis is performed by the sponsor and the outcome is negative; however, if at this timepoint, some participants have not completed 6 months of follow-up after their previous vaccination, the study will continue until this follow-up period is complete.
N=number of participants, vp=viral particles

The study includes one dose of study vaccine on Day 1.

At least 1,050 participants will be assigned to the Safety Subset of the global cohort, for which additional safety assessment will be collected.

Moreover, at least 360 participants are planned to be assigned to the Immuno Subset. For participants in the Immuno Subset blood will be collected for analysis of humoral and cellular immune responses.

For more details, please refer to Section 4.1 in the CTP.

2. STATISTICAL HYPOTHESES

The study is designed to test the primary hypothesis of VE against RT-PCR confirmed RSV-mediated LRTD in the PPE population. The hypotheses are:

- **Null hypothesis:** the VE against RT-PCR-confirmed RSV-mediated LRTD of the active study vaccine vs placebo is ≤20% .
- **Alternative hypothesis:** the VE against RT-PCR-confirmed RSV-mediated LRTD of active study vaccine vs placebo is >20%.

The study is successful if:

- The lower limit of the 95% 2-sided confidence interval (CI, potentially corrected as described in Section 6.3.2) around the VE (1-relative risk rate) calculated from the Exact Poisson regression model is above 20% and additionally a point-estimate of VE >50% is observed for the active study vaccine.

If VE for the primary endpoint is demonstrated, the following secondary endpoints will be tested:

1. First occurrence of any RT-PCR-confirmed RSV ARI, with onset at least 14 days after dosing of study vaccine
2. First occurrence of any RT-PCR-confirmed RSV-mediated LRTD during the second year, with onset after study Day 365
3. First occurrence of any RT-PCR-confirmed RSV ARI during the second year, with onset after study Day 365

4. First occurrence of predefined clinically relevant disease associated with RT-PCR-confirmed RSV-mediated ARI over the whole study, with onset at least 14 days after dosing of study vaccine

The following null and alternative hypothesis will be used for those secondary endpoints:

– The **null hypothesis** is that the VE for the considered secondary endpoint is ≤0%.

– The **alternative hypothesis** is that the VE for the considered secondary endpoint is >0%.

3. SAMPLE SIZE DETERMINATION

Please refer to Section 9.2 in the CTP.

4. POPULATIONS (ANALYSIS SETS) FOR ANALYSIS

For vaccine studies, study intervention assignment will follow the as treated principle.

Table 3: Analysis Sets

Analysis Sets	Description
**All Screened**	The analysis set includes all participants screened for the study, regardless if they were screening failures or they got enrolled in the study.<br><br>Note: Rescreened participants are counted only one time.
**Full Analysis Set (FAS)**	Full Analysis Set (FAS): will include all participants with a documented vaccine administration, regardless of the occurrence of protocol deviations. The FAS is the primary safety population for all cohort. The analysis of solicited and unsolicited AEs will be restricted to a subset of the FAS (i.e., the Safety Subset).
**Per Protocol Efficacy Analysis Set (PPE)**	Per-protocol Efficacy (PPE) population includes all randomized and vaccinated participants, excluding those with major protocol deviations (MPDs) expecting to impact the efficacy outcomes. Only data of the year (first year, second year) in which the MPD impacting efficacy occurs and the data of later years are excluded. The list of MPDs to be excluded from efficacy analyses will be specified in the major protocol violation criteria document, which will be finalized before database lock and unblinding. Participants with an RT-PCR-confirmed RSV-mediated ARI with onset within 14 days after vaccination, participants who discontinue within 14 days after vaccination, and participants who received vaccination within 14 days before the database cut-off date will be excluded from the PPE population. This analysis set will be used for analyses over the whole study period (up to database cut-off).<br><br>For analyses focusing on the first year, the PPE population is similar but only excludes MPDs occurring during the first year.<br><br>For analyses focusing on the second year, participants with MPDs expecting to impact the efficacy outcomes

Table 3: Analysis Sets

Analysis Sets	Description
	and occurring during the second year will be **additionally** excluded from the PPE set defined above, as well as participants who discontinued prior to Day 365.<br><br>The PPE population is the primary efficacy population. Sensitivity efficacy analyses will be performed on the FAS, restricted to cohorts where efficacy data are collected.
**Per Protocol Immunogenicity Analysis Set (PPI)**	Per-protocol Immunogenicity (PPI) population: will include all randomized and vaccinated participants who are part of the Immuno Subset and for whom immunogenicity data are available. Samples taken after a participant experiences a major protocol deviation (MPD) expected to impact the immunogenicity outcomes will be excluded from the PPI analysis.<br><br>The list of major protocol deviations that would lead to elimination from the immunogenicity analysis will be specified in the major protocol violation criteria document, which will be finalized before database lock and unblinding.<br><br>In addition, for participants who experience an RT-PCR-confirmed RSV-mediated ARI, samples taken after the RSV infection will not be taken into account in the assessment of the immunogenicity.<br>See Section 6.1.3 for visit windows.<br><br>The PPI population is the primary immunogenicity population. For key tables, sensitivity immunogenicity analyses will be performed on the FAS, including participants who are part of the Immuno Subset for whom immunogenicity measures are available. Excluded samples may be taken into account as well in the sensitivity analysis

5. PLANNED ANALYSIS

5.1. Analysis Plan for the Interim and Primary Analysis

Figure 1 displays the interim and primary analysis plan.

Figure 1: Interim and Primary Analysis Plan

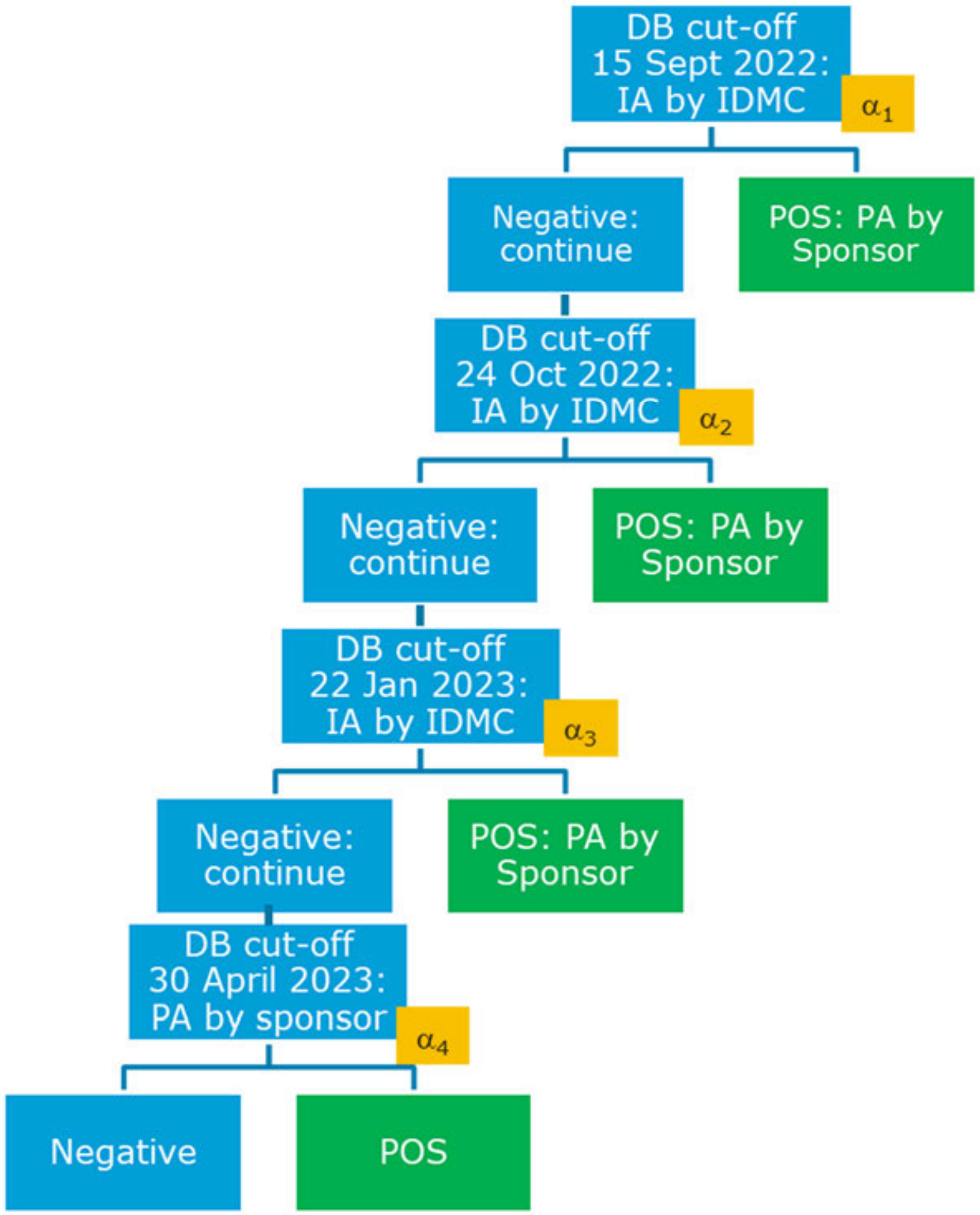

For the first interim analysis, ARIs with an onset up to 1 September 2022 will be included and a database cut-off of 15 September 2022 will be used. For the second IA, ARIs with an onset up to 10 October 2022 will be included and a database cut-off of 24 October 2022 will be used. For the third IA, ARIs with an onset up to 8 January 2023 will be included and a database cut-off of 22 January 2023 will be used. For the primary analysis, ARIs with an onset up to 15 April 2023 will be included and a database cut-off of 30 April 2023 will be used.
DB= database, IA= interim analysis, IDMC = independent data monitoring committee, PA=primary analysis, PPE=Per-protocol Efficacy, POS= positive

Please refer to Section 9.5.1 in the CTP for more details on the timing of the Interim and Primary analysis. Information regarding additional country-specific enrollment, beyond the global cohort enrollment, can be found in the country specific amendments.

In case the analysis is conducted prior to the completion of the study recruitment, all participants recruited in the global and local cohorts up to the database cut off will be used in the efficacy analysis, regardless the number of events already observed in the different local cohorts. Moreover, only participants vaccinated >14 days prior to the database cut off will be taken into account for the analysis of the primary efficacy endpoint, including the counting of LRTD cases. For the sensitivity analysis based on the FAS all participants will be taken into account.

Note: Participants with an RT-PCR-confirmed RSV-mediated ARI with onset within 14 days after vaccination, participants who discontinue within 14 days after vaccination and participants that have not reached 14 days (inclusive) after vaccination, will be excluded from the PPE population and thus from the counting of the number of events.

Local study teams will be blinded to study vaccination until the primary analysis as specified per local requirements. The local cohorts' primary analysis cannot be conducted prior to the primary analysis of the global cohort.

5.2. Additional Analyses

Provided the primary efficacy analysis has occurred earlier, the following exploratory analyses might be performed depending on program needs:

1. **Exploratory analysis with database cut-off of 31 January 2023**: will include unblinded efficacy data, including secondary endpoints #2, #3 and #4. The latter will be considered an exploratory descriptive analysis. Formal inference of those endpoints as part of the testing strategy will only occur at study end. Safety data will be included when needed. Immunogenicity data will be included if available.
2. **Exploratory analysis with database cut-off of 31 May 2023**: will include unblinded efficacy data, including secondary endpoints #2, #3 and #4. The latter will be considered an exploratory descriptive analysis. Formal inference of those endpoints as part of the testing strategy will only occur at study end. Safety data will be included when needed. Immunogenicity data will be included if available.

5.3. End-of-Study Analysis

The end of study analysis will include unblinded efficacy, safety and immunogenicity data. For secondary endpoints #2, #3 and #4, formal inference as part of the testing strategy will occur during this analysis.

Note: The timing of the end-of-study analysis is based on last subject last visit of the global cohort but will include all participants of global and local cohorts enrolled up to the cut-off of the final analysis.

6. STATISTICAL ANALYSES

6.1. General Considerations

6.1.1. Study Phases

A baseline (or reference) value for safety (except from vital signs) and immunogenicity will be defined as the value of the last available assessment prior to vaccination on Day 1. If no immunogenicity assessment was performed pre-vaccination, the (first) post-vaccination assessment on Day 1 will be used as the baseline value for the immunogenicity analysis, if available.

A baseline (or reference) value for all efficacy analysis, except from vital signs for the whole study will be defined as the value of the available assessment at Day 1 visit, if Day 1 visit is not available the Day 1 unscheduled visit closest to the day of vaccination will be used. In case two or more assessments are available at the same visit or at the same day of the selected baseline visit the worst will be used (per question).

For vital signs, the vital sign assessment collected at the pre dose timepoint will be defined as baseline (or reference).

For all efficacy analysis 'Month 12' will be defined similar to Day 1. 'Month 12' will be defined as the value of the available assessment at 'Month 12' visit, if 'Month 12' visit is not available the 'Month 12' unscheduled visit closest to the relative day 365 will be used. In case two or more assessments are available at the same visit or at the same day of the selected 'Month 12' visit the worst will be used (per question).

The safety analysis will present all results by phase. Immunogenicity results will be presented per scheduled time point as appropriate. Listings will be shown per phase/period and time point. Efficacy results will be presented across the first year, second year, whole study or per timepoint, where appropriate.

Study day or relative day is defined as follows:

Study Day = visit date – date of Day 1 + 1; if visit date >= date of Day 1 (date of vaccination).

Study Day = visit date – date of Day 1; if visit date < date of Day 1 (date of vaccination).

6.1.2. Phase Definitions

The phases in the study will be constructed as follows:

Table 4: Phase Definitions

Phase	Phase #	Period	Period #	Interval	
				**From**	**To**
Screening	1			Date and time of signing the informed consent form	One minute prior to start of post dose period
Regimen	2	Post-Dose	1	Date and time of vaccination	Minimum of:

Table 4: Phase Definitions

Phase	Phase #	Period	Period #	Interval From	Interval To
					a) 23:59 at the date of last contact (for early study discontinuation)<br>b) 23:59 at the database cut-off date in case of interim analysis<br>c) Maximum (28 days after vaccination at 23:59, scheduled visit 4 weeks after vaccination at 23:59)
First Year Follow up	3			One minute after Post-Dose period end	Minimum of:<br>a) 23:59 at the date of last contact (for early study discontinuation)<br>b) 23:59 at the database cut-off date in case of interim analysis<br>c) One minute prior to Second Year Follow up
Second Year Follow up	4			00:00 at the date of the Month 12 visit *	Minimum of:<br>a) 23:59 at the date of last contact (for early discontinuation or participants that completed the study)<br>b) 23:59 at the database cut-off date for analyses conducted before the final analysis.

The combination of 'Post-Dose' period and 'First Year Follow up' period will be referred as 'First Year'. The combination of the 'Regimen', 'First Year Follow up' and 'Second Year Follow up' periods will be referred as 'Whole Study' phase.

*If Month 12 visit not available, and there are ARI surveillance questions available after the relative day 365, then the relative day 365 (vaccination date + 365) will be used as the start of the Second Year Follow up.

'Month 12' and 'Day 365' visit are used interchangeably in the rest of the document.

For the potential adverse events of special interest (AESI) analysis the following intervals will also be used:

Table 5: Interval Definition for potential AESI analysis

Dose	Interval	Interval From	Interval To
Post-vaccination	0 - 28 days post-dose	Date time of vaccination	Min of:<br>• 23:59 at date of last contact (for discontinuations)<br>• 23:59 at the database cut-off date in case of interim analysis<br>• Maximum (date of vaccination + 28 days at 23:59, date of scheduled visit 4 weeks after vaccination at 23:59)
	29 - 56 days post-dose*	One minute after the end of the interval '0 - 28 days post-dose'	Min of:<br>• 23:59 at date of last contact (for discontinuations)

			• 23:59 at the database cut-off date in case of interim analysis<br>• Date of vaccination + 56 days, at 23:59
	57 days - 6 months post-dose	One minute after the end of the interval '29 - 56 days post-dose'	Min of:<br>• 23:59 at date of last contact (for discontinuations)<br>• 23:59 at the database cut-off date in case of interim analysis<br>• Maximum (Date of vaccination + 169 days at 23:59 , date of scheduled visit 169 days post vaccination at 23:59)
	> 6 months post-dose	One minute after the end of the interval '0 - 6 months post-dose'	Min of:<br>• 23:59 at the date of last contact (for discontinuations/ completions)<br>• 23:59 at the date of DB cut-off for interim analyses

The combination of the '0 - 28 days post-dose' interval and the '29 - 56 days post-dose' interval will be referred as '0 - 56 days post-dose'. The combination of the '0 - 28 days post-dose' ,'29 - 56 days post-dose' and '57 days - 6 months post-dose' intervals will be referred as '0-6 months post-dose'.

*In case the '0 - 28 days post-dose' interval end later than 56 days post vaccination then the '29 - 56 days post-dose' interval is not required.
For example, if a participant had Day 29 visit at relative day 57, then the following intervals will be created:

- 0 - 28 days post-dose, lasting from 0 to relative day 57,
- 57 days - 6 months post-dose, lasting from 58 onwards.

The same logic will apply for all intervals that end later after the next interval.

6.1.3. Immunogenicity Visit Windows

For immunogenicity summaries and tabulations per time point, assessments will be allocated to an analysis visit based on the planned visit as captured in the CRF. Visits that are out of the protocol-defined visit windows (see table below) will not be included in the immunogenicity summaries and tabulations per timepoint. However, they may be included in sensitivity analyses.

Table 6: Immunogenicity timepoints

Analysis timepoint	Reference day	Target day (counted from the reference day)	Window
Baseline	Day of vaccination	1	(-inf, 1]
Day 15	Day of vaccination	15	[15, 18]
Day 85	Day of vaccination	85	[78, 92]
Day 169	Day of vaccination	169	[155, 183]
Day 365/Month 12	Day of vaccination	365	[358, 379]
Day 533	Day of vaccination	533	[519, 547]

Immunogenicity samples taken during an ARI will be allocated to an analysis timepoint based on the relative day during the ARI and the below windowing. The relative day during the ARI is determined as ARI visit date – ARI start date +1. ARI immunogenicity timepoints will only be part of the correlate of protection analysis and predictive immune marker assessment where possible. Immunogenicity analysis related to correlate of protection will be described in a separate SAP.

Table 7: Immunogenicity timepoints during an ARI

Analysis timepoint	Reference day	Target day (counted from the reference day)	Window
ARI Day 3-5	Start date of ARI	4	[1,7]

6.2. Participant Dispositions

Participant information will be shown for the full analysis set, safety subset, PPE, PPI as well based on the risk level of the participant.

The number of participants in the following disposition categories will be summarized throughout the study by intervention group and overall:

- participants screened
- participants in the FAS
- participants in the PPI
- participants in the PPE
- participants vaccinated and not randomized
- participants randomized and not vaccinated
- participants not randomized and not vaccinated
- participants randomized and vaccinated
- participants who discontinued study
- participants who discontinued vaccination (for participants that discontinued before amendment 4 was approved)
- reasons for termination (with special interest on the COVID-19 related reasons)

Also, the number of participants and percentage per phase will be tabulated.

The time that participants are followed in the study will be summarized descriptively, for the FAS and PPE. The time that participants are followed in the study based on the FAS is defined as time between vaccination and the minimum between study completion, discontinuation or database cut off and based on the PPE is defined as time between vaccination and the minimum between study completion, discontinuation or database cut off or Day 365 planned visit (or relative day 365 if no planned Day 365 is present but there are ARI surveillance questions available after the relative day

365), in case the participant had a major protocol deviation impacting efficacy during the second year.

6.3. Primary Endpoint Analysis

The primary endpoint of the study is the first occurrence of RT-PCR-confirmed RSV-mediated LRTD, with onset at least 14 days after dosing with study vaccine.

The PPE will be used to analyze the primary efficacy endpoint.

6.3.1. Definition of Endpoint

A participant will be considered to have RT-PCR-confirmed RSV-mediated LRTD if the following criteria are met:

- New onset or worsening from baseline of 3 or more of the below symptoms as captured in the RiiQ at the same assessment time point:
  - Cough
  - Short of breath
  - Coughing up phlegm (sputum)
  - Wheezing

  AND

- Confirmation of RSV by RT-PCR in one or more of the nasal swabs, or in the sputum sample (when available)

Prior to database lock for all planned analyses, the CEC will review all available data of all RT-PCR confirmed RSV-mediated ARIs and will confirm if the above criteria are met. If the CEC confirms that the criteria for RT-PCR-confirmed RSV-mediated LRTD are met, the cases will be counted as a primary endpoint in the statistical analysis. This will be done in two steps:

Step 1:
The set of RT-PCR confirmed RSV-mediated ARIs for which the CEC will review all available data will be determined programmatically as follows:

ARI episode definition
An ARI episode is defined as an episode reported by the participant and confirmed by the site, which is captured on the 'Acute Respiratory Infection' form of the CRF, meaning that all ARIs with a confirmed start (CESTDTC) and end (CEENDTC) date on this form will be considered an ARI episode. ARIs initiated by error and their respective symptoms will be removed from the analysis.

Note: In the cases that an RSV ARI is ongoing during the DB cut off, the database cutoff date will be used as an interim ARI end date for the purposes of the analysis. An ARI with no end date will be considered as an RSV ARI, if an RSV positive result is available within 15 days after the ARI onset.

ARI episode duration

The duration of an ARI episode is defined based on the ARI start and ARI end date reported in the CRF: *end date of ARI- start date of ARI +1 (CEENDTC- CESTDTC+1).*

RSV RT-PCR Confirmation of the Considered ARI Episode

Confirmation of RSV infection by RT-PCR for the primary endpoint will be done in nasal swabs or sputum samples.

For samples collected outside of China, a sample is considered RSV positive if:

- Confirmation of RSV infection by RT-PCR in nasal swabs is obtained at the site laboratory with the BioFire Filmarray RP2.1-EZ panel, which will be provided to all sites (both US and non-US, except China sites). Both the home swab and the site swab will be analyzed at the site. The confirmation of RSV infection from the nasal swab at central laboratory using BioFire will not be taken into account for the primary endpoint, unless in the unlikely event where at least one nasal swab from an ARI episode cannot be tested at the site (eg, in the setting of instrument malfunction or shortage of testing reagents at the site).

- Confirmation of RSV infection by RT-PCR in sputum samples (when collected) is obtained in the central laboratory with the Biofire Filmarray PN panel.

- For participants hospitalized with symptoms of an ARI, confirmation of RSV infection using a locally approved RT-PCR test obtained at the hospital laboratory can also be used. These results will be used in the primary analysis only if approved by an independent expert with expertise in diagnostic testing. The approval of the RT-PCR results by the expert will be based on the specifications of the test performed, the qualifications of the testing laboratory and the procedures followed in the laboratory.

For samples collected in China, a sample is considered RSV positive if:

- Confirmation of RSV infection by RT-PCR in nasal swabs is obtained at the site laboratory with the Cepheid GeneXpert Flu-RSV XPRESS, which will be provided to all China sites. Both the home swab and the site swab will be analyzed at the site. The confirmation of RSV infection from the nasal swab at central laboratory using Cepheid GeneXpert Flu-RSV XPRESS will not be taken into account for the primary endpoint, unless in the unlikely event where at least one nasal swab from an ARI episode cannot be tested at the site (eg, in the setting of instrument malfunction or shortage of testing reagents at the site).

- Confirmation of RSV infection by RT-PCR in sputum samples (when collected) is obtained in the central laboratory with the Biofire Filmarray PN panel.

- For participants hospitalized with symptoms of an ARI, confirmation of RSV infection using a locally approved RT-PCR test obtained at the hospital laboratory can also be used. These results will be used in the primary analysis only if approved by an independent expert with expertise in diagnostic testing. The approval of the RT-PCR results by the expert will

be based on the specifications of the test performed, the qualifications of the testing laboratory and the procedures followed in the laboratory.

One positive sample (defined as any sample with a value above the limit of detection) is sufficient.

Any nasal swab and/or sputum sample taken within 7 days after start of an ARI episode or during the ARI episode will be allocated to that ARI. In case of 2 consecutive ARI episodes, where the second ARI starts within 7 days after the onset of the first episode and the ARI Day 3-5 sample of the first episode is taken while the second episode is ongoing, then the nasal swab and/or the sputum sample will be attributed to both ARIs.

Step 2:

For the set of RT-PCR confirmed RSV-mediated ARIs, the CEC will determine if the protocol defined criteria for LRTD (as specified in the beginning of this section) are met, using the following framework.

Counting of symptoms during an ARI episode to determine if LRTD criteria are met

To meet the primary endpoint, new onset or worsening from baseline in 3 or more LRTD symptoms (cough, short of breath, coughing up phlegm, wheezing) collected at the same assessment timepoint are required.

Symptoms present during an ARI episode but not at the baseline assessment will be considered as symptoms with a new onset. Symptoms present at the baseline assessment that become worse (higher severity) during the ARI episode will be considered as worsening symptoms. Missing baseline assessments will not be imputed, in case of missing baseline symptoms the participant will be considered to have no baseline symptoms at all. The baseline is defined in Section 6.1.1. Only assessments occurring during the ARI episode as defined above will be taken into account. Any assessment collected outside this period will not be used for counting symptoms.

Counting of the number of symptoms with new onset or worsening will be done per assessment timepoint. If a participant completed the RiiQ more than once per day, the case definitions cannot be met by combining symptoms from different assessments on that day, the required number of symptoms must be attained at one assessment.

Note: Only symptoms collected between the start of the ARI (CESTDTC) and the end of the ARI (CEENDTC) will be used for determining if the LRTD criteria are met.

Examples:

If a participant had an ARI lasting 4 days, with the following new onset or worsening of symptoms compared to baseline:

ARI Day 1: new onset of cough
ARI Day 2: new onset of wheezing and worsening of short of breath
ARI Day 3: new onset of cough and wheezing
ARI Day 4: worsening of short of breath

This participant does not meet the conditions of an LRTD.
If another participant had an ARI lasting 5 days, with the following new onset or worsening of symptoms compared to baseline:

ARI Day 1: new onset of cough
ARI Day 2: new onset of wheezing and worsening of short of breath and new onset of cough
ARI Day 3: new onset of cough and wheezing
ARI Day 4: new onset of cough
ARI Day 5: new onset of cough

This participant meets the condition of an LRTD at ARI Day 2.

Finally, if a participant completed by accident two RiiQ assessments at the same day, but at different timepoints with the following symptoms:

ARI Day 1: new onset of cough
ARI Day 2 (1st assessment): new onset of wheezing and worsening of short of breath
ARI Day 2 (2nd assessment): new onset of cough
ARI Day 3: new onset of cough and wheezing
ARI Day 4: new onset of cough
ARI Day 5: new onset of cough

This participant does not meet the conditions of an LRTD, since symptoms of two assessments cannot be combined.

RT-PCR confirmed RSV-mediated ARIs confirmed by the CEC as RSV-mediated LRTDs will be counted as primary endpoint in the statistical analysis, as per table below.

Table 8: Adjudicator's choices and selection for the primary endpoint

Option in the Adjudication System	Option in the Database	Included as Primary Endpoint
RSV positive ARI meets the CTP-defined criteria for RT-PCR confirmed RSV-mediated LRTD (in accordance with the programmed case definition)	RSV MEDIATED LRTD BOTH PROGRAMMED AND BY CEC	Yes
RSV positive ARI meets the CTP-defined criteria for RT-PCR confirmed RSV-mediated LRTD, but not classified as RT-PCR confirmed RSV-mediated LRTD by the medical assessment of CEC	RSV MEDIATED LRTD PROGRAMMED BUT NOT BY CEC	No
RSV positive ARI does not meet CTP-defined criteria for RT-PCR confirmed RSV-mediated LRTD but classified as RT-PCR confirmed RSV-mediated LRTD by the medical assessment of CEC	RSV MEDIATED LRTD BY CEC BUT NOT PROGRAMMED	Yes
RSV positive ARI not meeting CTP-defined criteria for RT-PCR confirmed RSV-mediated LRTD nor is classified as RT-PCR confirmed RSV-mediated LRTD by the medical assessment of CEC	NO RSV MEDIATED LRTD	No

For the analyses, unless explicitly specified, 'RSV-mediated LRTDs' is always referring to the ones assessed as such by the CEC.

6.3.2. Level of Significance

The total alpha will be controlled at 2.5%. A Pocock-like alpha spending function as described by DeMets (DeMets 1994) with a target number of events of 76 will be used to spend the alpha over the different analyses. The different alphas to be used during every IA are shown in Figure 1 and the calculations are explained below in Table 9

Table 9: Alpha spending at the Prespecified Analysis Timepoints

Alpha	Description of how to obtain alpha
α_1	Alpha obtained from Pocock-like alpha spending function with information fractions = [# RSV LRTD events by 15 September 2022 / 76[¥], 1]
α_2	Alpha obtained from Pocock-like alpha spending function with information fractions = [# RSV LRTD events by 15 September 2022 / 76[¥], # RSV LRTD events by 24 October 2022 / 76[¥], 1]
α_3	Alpha obtained from Pocock-like alpha spending function with information fractions = [# RSV LRTD events by 15 September 2022 / 76[¥], # RSV LRTD events by 24 October 2022 / 76[¥], # RSV LRTD events by 22 Jan 2023 / 76[¥], 1]
α_4	Alpha obtained from the remaining alpha to be spent (2.5% - total alpha spent during the interim analyses) and the actual information fractions observed at all analyses. That is, the information fraction, IF = [# RSV LRTD events by 15 September 2022 / # RSV LRTD events by 30 April 2023, # RSV LRTD events by 24 October 2022 / # RSV LRTD events by 30 April 2023, # RSV LRTD events by 22 January 2023 / # RSV LRTD events by 30 April 2023, 1]. The final alpha will be calculated using the user-defined alpha spending function approach (Jennison 2000).

More details related to the significance level the secondary efficacy endpoints will be tested can be found in Section 6.4.1.2. Refer to Section 2 for more details on the success criterion.

6.3.3. Analysis Methods

The analysis of the primary endpoint will evaluate the first occurrence of RT-PCR-confirmed RSV-mediated LRTD with onset of at least 14 days after the vaccination in the active vaccine group compared to the placebo group in the PPE population. Time at risk will also be taken into account. The null hypothesis of LL ≤20% for RT-PCR-confirmed RSV-mediated LRTD will be tested versus the alternative hypothesis of LL >20%. Additionally, the observed VE should be >50%.

<u>**Model:**</u>
Exact Poisson regression will be fitted with the event rate, defined as the number of events (with onset at least 14 days after vaccination) over the follow-up time (off-set) as dependent variable and the vaccination group, being at increased risk of RSV disease and age strata (both variables as stratified) as independent variables.

The model is defined with the ***proc genmod*** function in SAS, with an ***offset*** statement and a ***log*** (natural logarithm) link. The ***exact*** statement with the ***estimate = both*** option and an ***alpha = 2****

significance level from Section 6.3.2 is used to calculate the relative risk ratio and its confidence interval. The Vaccine Efficacy is then calculated as *1 – Relative Risk Rate*.

In case the algorithm does not converge, age and risk level will be removed from the model.

Each participant will be taken into account in the model with one observation indicating whether they had an event or not, the vaccination group, the stratification factors and the follow up time.

Follow up time:

The follow-up time is defined as the time between 14 days post vaccination (vaccination day + 14 days) and occurrence of the first event; for participants with no events, it is the time between 14 days post vaccination and the minimum of the database cut-off date of the analysis. However, for participants who discontinued or completed the study before having an event, follow-up time is defined as the time between 14 days post vaccination and the date of last contact (day of discontinuation or completion).

For participants with an MPD impacting efficacy during the second year (during the second year follow up period), only the first year data are taken into account: for cases during the first year, the follow - up time is defined as the time between 14 days post-vaccination and occurrence of the first event . For non-cases, it is the time between 14 days post - vaccination and the day before Day 365 visit (or relative day 365, in case Day 365 not available but there are ARI surveillance questions available after the relative day 365). Thus, all participants will be included in the analysis according to their follow-up time.

Note: Occurrence is always referring to the start of the ARI as reported in the CRF (CESTDTC).

As **sensitivity analyses** the above Poisson model will be repeated similar to the primary analysis:

- based on the FAS (which also does not take into account the restriction on the onset [at least 14 days] and will count follow up from vaccination on Day 1 onwards).

- based on the PPE set but excluding coinfections with other respiratory viruses, based on the Biofire - Filmarray assay (Section 7.7) or the Cepheid GeneXpert (for swabs in China sites). This should be detected in one of the scheduled nasal or sputum samples linked to the respective ARI but should not necessarily be detected in the same sample as the one that resulted in RSV confirmation.

- based on the PPE set but excluding coinfections with other respiratory viruses or bacteria, based on the Biofire - Filmarray assay (Section 7.7) or the Cepheid GeneXpert (for swabs in China sites). This should be detected in one of the scheduled nasal or sputum samples linked to the respective ARI but should not necessarily be detected in the same sample as the one that resulted in RSV confirmation.

- based on the PPE set with age and increased risk status as collected (instead of as stratified) in case there are many discrepancies between as collected and as stratified.

- based on the PPE set but excluding RSV positive tests from local hospital assays of hospitalized participants.
- based on the PPE set, using the only central RSV results by Biofire Filmarray PN panel, Cepheid GeneXpert and BioFire Filmarray RP2.1-EZ and the approved local RT-PCR test from hospitalization cases. This sensitivity analysis will only be performed, if there are positive RSV cases based on the local site results of the Cepheid GeneXpert (China sites) or BioFire Filmarray RP2.1-EZ (other sites), that are negative based on the central confirmation.
- based on the PPE, using a robust Poisson regression similarly defined as the primary endpoint including the minimization factors region (North America, Europe and Southern Hemisphere if applicable), subset (safety, immuno, both or neither) as additional covariates. The model will be fitted in SAS similarly to the primary analysis with a ***repeated*** statement instead of an ***exact*** and ***type=unstr***. This model will only be performed if the algorithm converges.
- based on the programmed case definition of RT-PCR confirmed RSV-mediated LRTD, in case there are discrepancies with the CEC adjudication.
- an exact binomial test (See Section 7.11), which does not take into account strata or follow-up time, based on the PPE population and the FAS. The VE (=1-relative risk) and the corresponding corrected 2-sided CI based on the exact binomial will be calculated as well.
- based on the PPE set, but instead of focusing on the first occurrence of RT-PCR confirmed RSV-mediated LRTD, the number of events will be used as dependent variable and the offset will be defined as the time between 14 days post vaccination and the minimum of (the database cut-off date , study discontinuation, study completion). For participants with an MPD impacting efficacy during the second year (during the second year follow up period), only the first year data are taken into account, and the follow up with be defined as the time between 14 days post vaccination and the minimum of (the database cut-off date , Day 365 visit -1 or relative day 365 -1, if Day 365 visit not available but there are ARI surveillance questions available after the relative day 365).
- A sensitivity analysis using multiple imputations will be performed to evaluate the impact of missing RT-PCR results on the primary endpoint. For ARIs where no RT-PCR result can be linked to the considered ARI episode the RT-PCR result (positive or negative) will be imputed using the period the ARI occurred, the geographic location (Europe, Canada, the RSV census regions for USA sites [West, North East, South, Midwest], Latin America [Brazil and Chile], South Africa, Thailand and Oceania [Australia and New Zealand]), the age, the risk level (CDC definition) of the participants and the severity of the episode *(LRTD or not LRTD)* as parameters in the model. The ARI periods will be defined based on the distribution of the cases observed during the study. Moreover, the geographic location grouping might be adjusted to avoid model fitting issues. Based on the model fit parameters might be dropped.

  If one or more RT-PCR test results are available, no imputation will be performed. Moreover, the primary analysis model already accounts for dropouts, since participants follow up time is taken into consideration.

- A rerandomization test will be performed to assess the impact of the minimization algorithm.

If the primary analysis is performed in 2023 the following sensitivity analyses will also be conducted.

- New case will be defined for the second year using Day 365 as baseline to define new onset or worsening of symptoms. The cases of the first year will be defined using Day 1 as baseline. In case the Day 365 visit not available the Day 1 data will be used as baseline for both years. The same Poisson model as the primary endpoint based on the PPE will be performed.

- An exact Poisson model similar to the primary analysis will be performed including an interaction term between the treatment group and the year variables.

The proportion of participants meeting the primary endpoint and the sensitivity analyses, will also be summarized with the corresponding VE and will be depicted with forest plots. The same alpha as in the primary analysis will be used for calculating the CIs.

6.4. Secondary Endpoints Analysis

The PPE will be used to analyze all secondary efficacy endpoints.

6.4.1. Key Confirmatory Secondary Endpoints

The following secondary endpoint will be formally analyzed:

1. First occurrence of any RT-PCR-confirmed RSV ARI, with onset at least 14 days after dosing of study vaccine
2. First occurrence of any RT-PCR-confirmed RSV-mediated LRTD during the second year, with onset after study Day 365
3. First occurrence of any RT-PCR-confirmed RSV ARI during the second year, with onset after study Day 365
4. First occurrence of predefined clinically relevant disease associated with RT-PCR-confirmed RSV-mediated ARI over the whole study, with onset at least 14 days after dosing of study vaccine

Formal inference for secondary endpoint 1 (RSV ARI) as part of the testing strategy will occur at the primary analysis, for secondary endpoints 2, 3 and 4 the formal inference as part of the testing strategy will only occur at study end (Table 10). Exploratory descriptive analyses of these endpoints might also occur at other planned analysis timepoints.

Note: Occurrence is always referring to the start of the ARI as reported in the CRF (CESTDTC). Second year starts at the study Day 365 visit, or relative day 365, in case Day 365 visit not available but there are ARI surveillance questions available after the relative day 365.

6.4.1.1. Definition of Endpoints

6.4.1.1.1. Case Definition for RT-PCR-confirmed RSV-mediated ARI

A participant will be considered to have RT-PCR-confirmed RSV-mediated ARI if the following criteria are met:

ARI episode reported by participant and confirmed by the site. See '*ARI episode definition'* in Section 6.3.1.

Confirmation of RSV by RT-PCR in one or more of the nasal swabs, or in the sputum sample, when available. See '*RSV RT-PCR Confirmation of the Considered ARI Episode'* in Section 6.3.1.

6.4.1.1.2. Predefined Clinically Relevant Disease Associated with RT-PCR-confirmed RSV-mediated ARI

A participant will be considered to have clinically relevant disease with specific parameters associated with an RT-PCR-confirmed RSV-mediated ARI if the following criteria are met:

The participant has an RT-PCR-confirmed RSV-mediated ARI as defined in Section 6.4.1.1.1.

Any of the following is associated with the ARI:

- **Hospitalization** (as collected on eCRF AE pages (linked via acute respiratory infection form or complications linked to ARI form), medical encounters [Intensive Care Unit and Hospital inpatient department] form and MRU pages [Emerging Hospitalization admissions (Emerging emergency department visits are not taken into account)]

- **Emergency department visit** (as assessed on the eCRF medical encounters form and MRU pages [emerging MRU to be summarized])

- At least one of the following **complications**: asthma, COPD, respiratory distress, bronchitis, bronchial hyperreactivity, CHF, cardiac arrhythmia, renal impairment or the presence of X-ray or radiological confirmed , respiratory arrest and/or failure, pulmonary embolism, pleural effusion, atelectasis, acute coronary events, acute cerebrovascular events, altered mental status, seizure, syncope, systemic inflammatory response syndrome (SIRS), new neurological deficit, asthenia, dehydration or metabolic disturbances.

  See Section 7.8 for the complication terms. These terms will be reported in the AE or the CE domain.

  Asthma and chronic obstructive pulmonary disease (COPD) exacerbation supporting LRTD reported in the medically attended ARI form will also be counted, as well as X-ray or radiological confirmed pneumonia from the diagnostic tests form.

- **Decreased oxygen saturation**:
  Based on the vital signs form collected at the site: defined as oxygen saturation of <92% for participants with a baseline oxygen saturation of ≥92% or missing at randomization; for participants with baseline oxygen saturation <92%, decreased oxygen saturation is defined as a ≥3% decrease in their oxygen saturation from baseline
  Based on eCRF pages on measurements during a medically attended ARI (eq. hospitalizations): defined as oxygen saturation of <92% supporting LRTD

- **Tachypnea** (at least emerging Grade 2, see respiratory rate at Section 7.6)
- Need of **supplemental oxygen** (as indicated on the eCRF medical encounters form, the eCRF oxygen supplementation form and from emerging MRUs [both '*Supplemental oxygen used?*' and '*Mechanical ventilation used?*' questions to be used])
- **Hypotension** (emerging Grade 3, see Section 7.6)
- **Pulmonary function test** results supporting diagnosis of LRTD (as captured under the eCRF pages on measurements during a medically attended ARI, where peak expiratory flow, spirometry FVC, FEV1 or FEV6 support diagnosis of LRTD)
- **Arterial blood gas** results supporting diagnosis of LRTD (as captured under the eCRF pages on measurements during a medically attended ARI, where arterial blood gas pH, pO_2, pCO_2, HCO_3 supports diagnosis of LRTD)

6.4.1.2. Level of Significance

The VE against the primary endpoint will be tested using the significance level α' (α_1 or α_2 or α_3 or α_4), as defined in Section 6.3.2. If the LL of the exact 2-sided CI (1-2×α') for the VE (1-relative risk rate) calculated from the Exact Poisson regression model, is above 20% and the observed VE is above 50%, VE is demonstrated for the primary endpoint. If VE is demonstrated for the primary endpoint, the above secondary endpoints will be tested according to the testing order displayed in Table 10. The first secondary efficacy endpoint (first occurrence of any RT-PCR-confirmed RSV ARI) is tested using the same significance level α' as used for the primary endpoint. Exact 2-sided CIs (1-2×α') for the VE (1-relative risk rate) will be calculated from these Exact Poisson regression models. If the LL of the CI is above 0%, VE will have been demonstrated for this endpoint and the next endpoint in the testing strategy can be tested. The following secondary endpoints (#2, #3 and #4) will be tested hierarchically at the end of the study at the full alpha (α=2.5%), as indicated in Table 10 below.

Table 10: Secondary Endpoints: Overview of the Testing Order, Timing of the Testing in the Testing Strategy and the Corresponding Alpha

Secondary endpoint: first occurrence of	***Analysis timepoint in the testing strategy***	***Alpha at which the endpoint is tested***
1. Any RT-PCR confirmed RSV ARI	Time of primary analysis	Alpha used at the primary analysis
2. Any RT-PCR confirmed RSV LRTD during the second Year	End of the study	2.5%
3. Any RT-PCR confirmed RSV ARI during the second Year	End of the study	2.5%
4. Predefined clinically relevant disease associated with RT-PCR-confirmed RSV-mediated ARI	End of the study	2.5%

Second year starts at the D365 visit

6.4.1.2.1. Analysis Methods

A similar Exact Poisson regression model as for the primary endpoint will be used for the analyses of the secondary endpoints focusing on VE.

For secondary endpoints focusing on VE during the second year, the follow up time will be defined as indicated below:

For participants with events occurring from the Day 365 onwards, the follow-up time is defined as the time between Day 365 study visit and the occurrence of the event. For participants with no events, the follow-up time is the time between Day 365 study visit and the minimum of the database cut-off date of the analysis and the completion or discontinuation date. Participants who discontinue prior to the Month 12/Day 365 visit and participants that have an MPD impacting efficacy during the first or second year are excluded from this analysis. For those secondary endpoints, each participant will be taken into account in the model with one observation indicating whether they had an event or not, the vaccination group, the stratification factors and the follow up time. Events with onset from the study Day 365 onwards will be considered in the model.

For the first occurrence of any RT-PCR-confirmed RSV ARI and first occurrence of predefined clinically relevant disease endpoints, refer to Section 6.3.3.

Note: Occurrence is always referring to the start of the ARI as reported in the CRF (CESTDTC). Second year starts at the study Day 365 visit, or relative day 365, in case Day 365 visit not available but there are ARI surveillance questions available after the relative day 365.

The proportion of participants meeting the different secondary endpoints, will also be summarized with the corresponding VE and will be depicted with forest plots.

6.4.2. Supportive (Non-Confirmatory) Secondary Endpoint

The AUC of the change from baseline in RiiQ Total Symptom score (see Section 6.6.7.1) in participants with an RT-PCR-confirmed RSV-mediated ARI over the whole study.

The AUC is calculated from the first symptoms reported after the start of the ARI (CESTDTC) until the end of the ARI (CEENDTC). See Section 7.9 for details on the calculations.

6.4.2.1. Analysis Methods

The AUC of the change from baseline in RiiQ Total Symptoms score from the ARI episode corresponding with the first occurrence of any RT-PCR-confirmed RSV-mediated ARI over the whole study, with onset at least 14 days after vaccination will be summarized.

Descriptive statistics will be calculated restricting to participants with RT-PCR confirmed RSV-mediated ARI during the whole study. A Wilcoxon Rank Sum test will be performed to test the null hypothesis that the distribution in both groups is the same versus the alternative hypothesis that the distribution shifted towards a lower AUC in the change from baseline in RiiQ Total Symptom score (ie, more reduced symptom scores) in the considered active group. This is a non-confirmatory endpoint and will not be taken into account in the testing strategy, no alpha correction will be applied.

6.5. Exploratory Endpoints Analysis

The PPE will be used to analyze all exploratory efficacy analysis. For the definition of RT-PCR confirmed RSV- mediated LRTD and ARI please refer to Section 6.3.1.

Moreover, RT-PCR confirmed Flu and hMPV- mediated LRTD and ARI are defined in the same way as the RSV- mediated LRTD and ARI, Section 6.3.1, but instead of an RSV confirmation a Flu or hMPV confirmation is required, respectively. The CEC will not evaluate the RT-PCR confirmed Flu and hMPV- mediated ARIs, therefore the LRTD definition will be based on the programmed definition. The following exploratory endpoints, will be analyzed:

- First occurrence of RT-PCR-confirmed RSV-mediated LRTD during the first year, with onset between 14 days post vaccination and Day 365.
- First occurrence of RT-PCR-confirmed RSV-mediated ARI during the first year, with onset between 14 days post vaccination and Day 365.
- First occurrence of RT-PCR-confirmed RSV-mediated LRTD by subtype (RSV A or RSV B), based on the DDL assay

  Note: This is defined as an RT-PCR-confirmed RSV-mediated LRTD (see Section 6.3.1) in combination with the considered RSV subtype (RSV A or RSV B respectively) obtained from DDL in at least one of the samples taken up to 7 days after the onset of the ARI episode or during the ARI episode. Both nasal and sputum swabs will be taken into consideration.

- First occurrence of RT-PCR-confirmed RSV-mediated ARIs by subtype (RSV A or RSV B), based on the DDL assay

  Note: This is defined as an RT-PCR-confirmed RSV-mediated ARI (see Section 6.4.1.1.1) in combination with the considered RSV subtype (RSV A or RSV B respectively) obtained from DDL in at least one of the samples taken up to 7 days after the onset of the ARI episode or during the ARI episode. Both nasal and sputum swabs will be taken into consideration.

First occurrence of complications associated with RT-PCR-confirmed RSV-mediated ARIs, RT-PCR-confirmed RSV-mediated LRTDs, influenza-mediated ARIs and hMPV-mediated ARIs. For the list of complications see corresponding bullet in Section 6.4.1.1.2.

- First occurrence of hospitalizations associated with RT-PCR-confirmed RSV-mediated ARIs, RSV-mediated LRTD, influenza-mediated ARIs and hMPV-mediated ARIs. See Section 6.4.1.1.2 for hospitalization definition.
- First occurrence of pneumonia (radiological or X-ray confirmed, as assessed in the CRF diagnostic tests form or reported as a complication) associated with RT-PCR-confirmed RSV-mediated ARIs, RSV-mediated LRTD, influenza-mediated ARIs and hMPV-mediated ARIs
- First occurrence of emerging therapeutic interventions of interest associated with RT-PCR-confirmed RSV-mediated ARIs, RSV-mediated LRTD, influenza-mediated ARIs and hMPV-mediated ARIs. The following are considered emerging therapeutic interventions of interest (Section 7.10) :
  - New onset or increased dose (compared to baseline) of bronchodilator/nebulizer treatment

- New onset or increased dose (compared to baseline) of corticosteroid prescription
- New onset or increased dose (compared to baseline) of antibiotic prescription
- New onset or increased dose (compared to baseline) of antiviral prescription

Note: These will be taken from the concomitant medication pages of the eCRF. The concomitant medications listed in Section 7.10 are considered as therapeutic interventions of interest. The principal investigator will indicate whether there has been new onset of increase in dose compared to baseline in the CRF. Those associated with an ARI or complication of an ARI will be displayed.

- First occurrence of RT-PCR confirmed hMPV-mediated ARI
- First occurrence of RT-PCR-confirmed hMPV-mediated LRTD
- First occurrence of RT-PCR confirmed influenza-mediated ARI and influenza-mediated LRTD
- First occurrence of an RT-PCR-confirmed RSV LRTD assessed as at least mild, as at least moderate and as severe by the CEC during the considered year:
  - with onset at least 14 days after dosing of study vaccine and prior to study Day 365 (first year comparison)
  - with onset from the study Day 365 onwards (second year comparison)
  - with onset at least 14 days after dosing of study vaccine (whole study comparison)

Note: Occurrence is always referring to the time to the start of the ARI as reported in the CRF (CESTDTC). The start of the associated ARI will also be used for calculating the time to first occurrence of emerging therapeutic interventions, pneumonia, complications and hospitalizations.

- First occurrence of RT-PCR-confirmed, RSV-mediated LRTD during the second year and over the whole study (if the primary analysis is conducted before the end of the study):
  - with onset from the study Day 365 visit onwards (second year comparison)
  - with onset 14 days after the first dose of study vaccine (whole study comparison)
- First occurrence of RT-PCR-confirmed, RSV-mediated ARI over the whole study (if the primary analysis is conducted before the end of the study), with onset at least 14 days after the first dose of study vaccine
- First occurrence of predefined clinically relevant disease associated with RT-PCR-confirmed RSV-mediated ARI and LRTD

Note: If the primary analysis occurs in 2022 exploratory endpoints related to the whole study (data collected up to Data base cut off) will be analyzed, and the first- and second-year endpoints will not be analyzed at that timepoint.

If the primary analysis occurs in 2023 all exploratory endpoints will be analyzed up to the date of the data base cut off.

The inclusion of Flu and hMPV endpoints in the primary or additional analyses is depending on the cleaning status of those cases at the time of analyses. They will be included in the end of study analysis.

6.5.1. Analysis Methods

The VEs will be calculated for the above endpoints, occurring during the first year, during the second year and over the whole study, except for all endpoints related to RT-PCR confirmed influenza-mediated ARI and LRTD. For all exploratory endpoints related to influenza-mediated ARI and LRTD only the proportion of participants meeting the respective endpoints, will be summarized.

The PPE set will be used for the analysis of these exploratory endpoints.

For endpoints focusing on the first year or during the whole study, events with onset at least 14 days after the vaccination will be considered. And for endpoints focusing on the second year, event with onset from the study Day 365 onwards will be considered.

A similar Exact Poisson regression model as for the primary and secondary endpoints will be used and exact 2-sided 95% CIs for VE (1-relative risk rate) will be calculated. For endpoints focusing on the whole study or the second year, the follow-up time is determined in a similar way as for the primary and secondary endpoints, respectively. For endpoints focusing on the first year, the follow up time is defined as the time between 14 days post vaccination (vaccination day + 14 days) and occurrence of the first event during the first year; for participants with no events, it is the time between 14 days post vaccination and the minimum of the database cut-off date of the analysis and a day prior to study Day 365 (or relative day 365, if the Day 365 visit not available but there are ARI surveillance questions available after the relative day 365). However, for participants who discontinued the study before having an event, follow-up time is defined as the time between 14 days post vaccination and the minimum of the database cut-off date, a day prior to study Day 365 (or relative day 365, if the Day 365 visit not available but there are ARI surveillance questions available after the relative day 365) and date of last contact (day of discontinuation).

The proportion of participants meeting the different exploratory endpoints, will also be summarized with the corresponding VE and will be depicted with forest plots.

The proportion of participants with emerging therapeutic interventions of interest by subcategory (bronchodilator/nebulizer treatment, corticosteroid prescription, antibiotic prescription, antiviral prescription) associated with RT-PCR-confirmed RSV-mediated ARI, LRTD and no LRTDs will also be summarized per group, category and risk level, for the first, second year and over the whole study. In case a participant had multiple RT-PCR confirmed RSV-mediated ARIs or LRTDs during the reported period, then the therapeutic interventions corresponding with the first occurrence of RT-PCR confirmed RSV-mediated ARI or LRTD will be used, respectively.

The proportion of participants with therapeutic interventions of interest by subcategory at the days of vaccination will also be summarized per group, category and risk level.

6.6. Other Exploratory Analysis

If the primary analysis occurs in 2022 the exploratory endpoints related to the whole study (data collected up to Data base cut off) will be analyzed, and the first- and second-year endpoints will not be analyzed at that timepoint.

If the primary analysis occurs in 2023 all exploratory endpoints will be analyzed up to the date of the data base cut off.

6.6.1. ARI Duration

For participants with an RT-PCR confirmed RSV-mediated ARI and for those with a RT-PCR-confirmed RSV-mediated LRTD, the ARI duration will also be summarized with Kaplan Meier curves. The duration is defined as: end date of the ARI episode – start date of the ARI episode + 1 (see section 6.3.1). Hazard ratios will be calculated, with a Cox proportional hazard model with ARI duration as dependent variable and group as independent variable for the whole study. In case that the ARI was stopped by the PI before the two consecutive days back to baseline for the RiiQ assessment (indicated by a flag in the DB), the record will be considered to be censored.

Similar to the ARI duration, the **time to first occurrence** for a RT-PCR confirmed RSV-mediated ARI, LRTD, ARI by subtype and LRTD by subtype will be summarized with Kaplan Meier curves. Hazard ratios will be calculated, with a Cox proportional hazard model with time to first occurrence as dependent variable and group as independent variable for the first year, second year and the whole study. Participants with no events will be considered in the analysis as censored records.

Time to first occurrence, will be calculated for:

- first and second year separately, (determination of the time to first occurrence is similar to 'follow up time' as described in Section 6.4.1.2.1 and 6.5.16.3.3)
- the entire study (determination of the time to first occurrence is similar to 'follow up time' as described in Section 6.3.3 and 6.4.1.2.1)

In case a participant had multiple RT-PCR confirmed RSV-mediated ARIs or LRTDs during the reported period, then the first occurrence of RT-PCR confirmed RSV-mediated ARI or LRTD will be used, respectively.

6.6.2. ARI Symptoms

For participants with RT-PCR-confirmed RSV-mediated ARIs, influenza-mediated ARIs and hMPV-mediated ARIs the combination of the symptoms (cough, short of breath, coughing up phlegm ,wheezing, fever and fatigue) will be summarized per group and risk level for each year separately and over the whole study.

6.6.3. ARI Complications

All complications associated with any RT-PCR confirmed RSV-mediated ARI, LRTD, and non LRTD will be summarized per group and per risk level, during the first and the second year separately, and over the whole study. A summary table including the number of participants with

any complication, any complication per worst grade reported, with fatal outcome and reported as SAE will be created. All complications and those reported as SAEs will also be summarized by System Organ Class and Preferred Term. In case a participant had multiple RT-PCR confirmed RSV-mediated ARIs or LRTDs during the reported period, then the first occurrence of RT-PCR confirmed RSV-mediated ARI or LRTD will be used, respectively.

6.6.4. Clinically Relevant Disease

The proportion of participants with clinically relevant disease (See Section 6.4.1) by subcategory associated with RT-PCR-confirmed RSV-mediated ARI, LRTD and non LRTD will also be summarized per group, category and risk level for the first and second year separately and over the whole study. In case a participant had multiple RT-PCR confirmed RSV-mediated ARIs or LRTDs during the reported period, then the first occurrence of RT-PCR confirmed RSV-mediated ARI or LRTD will be used, respectively.

6.6.5. Vital Signs During the ARI episodes

The following will be summarized for the first and the second year separately and over the whole study.

Emerging vital sign abnormalities collected at ARI Day 3-5 for participants with an RT-PCR confirmed RSV-mediated ARI and/or LRTD will be summarized per vaccine group. An abnormality will be considered as emerging in a particular period if it is worse than the baseline value. The baseline value for efficacy will be used to define abnormalities (See Section 6.1.1). Please refer to Section 7.6 for the vital sign grades. If the baseline is missing, the abnormality is always considered emerging.

Moreover, for the determination of oxygen saturation collected by the vital signs page during the ARI Day 3-5, the number of subjects (and percentages) :

- with baseline $SpO_2 \geq 92\%$ and $SpO_2 < 92\%$ during the ARI episode,
- with baseline $SpO_2 < 92$ and that have a $\geq 3\%$ decrease during ARI episode
- and those with a missing baseline and $SpO_2 < 92\%$ during the ARI episode

will be tabulated per group, for participants with a RT-PCR confirmed RSV-mediated ARI and for those with a RT-PCR-confirmed RSV-mediated LRTDs.

In case a participant had multiple RT-PCR confirmed RSV-mediated ARIs or LRTDs during the reported period, then the first occurrence of RT-PCR confirmed RSV-mediated ARI or LRTD will be used, respectively.

6.6.6. RSV Viral Load.

The following will be summarized for the first and the second year separately and over the whole study.

For RSV A and RSV B viral load based on nasal swabs, descriptive statistics for ARI Day 1-2, ARI Day 3-5 and for the maximum viral load observed between the 2 visits, will be calculated for participants with a RT-PCR confirmed RSV-mediated ARI of the considered subtype and for those

with a RT-PCR-confirmed RSV-mediated LRTD of the considered subtype. The difference and 95% CI of the maximum log10 viral load between the 2 groups will also be calculated. Means and standard errors will also be plotted.
For RSV A and RSV B viral load based on sputum will be listed.

In case a participant had multiple RT-PCR confirmed RSV-mediated ARIs or LRTDs during the reported period, then the first occurrence of RT-PCR confirmed RSV-mediated ARI or LRTD will be used, respectively.

For analysis purposes, for both nasal and sputum swabs, the qRT-PCR viral load will be imputed with the midpoint on the log scale between the limit of detection (LOD) and LLOQ of the RSV qRT-PCR assay when the result is 'target detected' (TD) but non-quantifiable.

1. For the RSV-A qRT-PCR assay, the LOD is 620 copies/mL and the LLOQ is 1000 copies/mL, a result that is TD will be imputed with 2.90 $\log_{10}$ copies/mL.
2. For the RSV-B qRT-PCR assay, the LOD is 80 copies/mL and the LLOQ is 250 copies/mL, a result that is TD will be imputed with 2.15 $\log_{10}$ copies/mL.

When the result is 'target not detected' (TND) (meaning below the LOD), for both RSV A and RSV B the value of TND will be imputed with the respective LODs.

RNA sequencing data, if available, will be explored with heatmaps.

6.6.7. Patient Reported outcomes

6.6.7.1. Respiratory Infection Intensity and Impact Questionnaire (RiiQ™v2)

The RiiQ consists of 4 scales that are scored separately. In the setting of the present study, only the RiiQ Symptom Scale (Question 1) and the RiiQ Impact on Daily Activities Scale (Question 2) will be used.

- **RiiQ Symptom Scale (Question 1).** Each symptom is rated on the following scale: 0=None, 1=Mild, 2=Moderate, and 3=Severe. Based on this questionnaire, the following will be calculated:
  - **RiiQ Total Symptom score** is the mean of all scores (based on 13 symptoms).
  - **RiiQ Lower Respiratory Symptom score** is the mean of the four lower respiratory scores (cough, short of breath, coughing up phlegm (sputum) and wheezing).
  - The **AUC** (Section 7.9) **for the change from baseline for the RiiQ Total Symptom score and the RiiQ Lower Respiratory Symptom score** during the ARI will be calculated.

– **RiiQ Impact Scale Question 2:**
  - **RiiQ Total Impact on Daily Activity score (Question 2)** consists of 7 activities. Ability to perform each activity item is rated on the following scale: 0=No difficulty, 1=Some difficulty, 2=Moderate Difficulty, and 3=Great difficulty. The total score is calculated as the mean of all 7 items (range 0-3).

- Moreover, the **AUC for the change from baseline for the RiiQ Total Impact on Daily Activity score** during the ARI will be calculated.

Note 1: Total scores will be calculated based on the number of assessments completed by the participant and in cases where more than 50% of the items needed to calculate the score is not collected, then the value for that score will be set to missing. For example, if a participant has responded to only 11 out of the 13 RiiQ symptom scale questions the 'RiiQ Total Symptom score' will be the mean of the 11 available questions. If the participant has only completed 6 or less of the questions, then the 'RiiQ Total Symptom score' will be set to missing.

6.6.7.1.1. Analysis Methods

RiiQ scores will be analyzed based on the PPE set, with a major focus on participants with RT-PCR-confirmed RSV-mediated ARI and LRTD, for the first, second year and the whole study. Part of the analysis might also be performed for participants with RT-PCR-confirmed hMPV-mediated and influenza-mediated ARI.

For the **total scores**, number of observations, mean, standard error, median, first and third quartile will be tabulated for the change from baseline for all RiiQ scores per group and means with standard errors may be graphically presented per group and time point for changes from baseline for different scores.

For the **total scores**, descriptive statistics at the start and end of each year as well as the change between the two timepoints will also be calculated for all participants based on the PPE set, for participants with RT-PCR-confirmed RSV ARI and those with RT-PCR-confirmed RSV LRTD, based on the PPE set.

For the **AUCs** number of observations, median, first and third quartile (q1, q3), minimum and maximum will be calculated and graphically presented with boxplots per group.

In case two or more assessments are reported at the same day then the worst total score will be used. The same applies for the AUC calculations. In case a participant had multiple RT-PCR confirmed RSV-mediated ARIs or LRTDs during the reported period, then the AUC corresponding to the first occurrence of RT-PCR confirmed RSV-mediated ARI or LRTD will be used, respectively.

6.6.7.2. Patient Global Impression Scores

- **Patient Global Impression of Health (PGI-H).** Participants report their overall impression of their health status today on the following scale: 0=Very good, 1=Good, 2=Fair, 3=Poor, 4=Very poor
- **Patient Global Impression of Severity (PGI-S).** Participants rate the severity of their respiratory illness on the following scale: 0=none 1=mild, 2=moderate, 3=severe.
- **Patient Global Impression of Change (PGI-C).** Participants rate the amount of change in their health each day during an ARI episode on the following scale: -3= much better, -2=somewhat better, -1=A little better, 0=About the same, 1=A little worse, 2=somewhat worse, 3= much worse

The **AUC** (Section 7.9) **for the change from baseline for the PGI-H and the AUC of the actual values of the PGI-C and PGI-S** during the ARI will be calculated

- **Return to Usual Health.** Participants are being asked whether they have returned to their usual health after developing symptoms suggesting an ARI. The response is binary, yes/no.

  Number of days a participant took to return to its usual health: This is counted based on the Return to Usual Health question.

  - In case a participant indicated he returned to its usual health (based on the 'Return to usual health' question) on the last day he completed this questionnaire during the considered episode, this is calculated as: *the first day of a series where the participant answered consecutively 'yes' to the 'Return to usual health question' (with no intermittent 'no' answers) - the ARI start date based on the CRF+1* . For the Kaplan-Meier curve this is considered an event.
  - In case a participant indicated he did not return to its usual health (based on the 'Return to usual health' question) on the last day he completed this questionnaire during the considered episode, this is calculated as: *the date of the last reply- the ARI start date based on the CRF + 1*. For the Kaplan-Meier curve this record will be censored.
  - In case a participant never responded to any 'Return to usual health' question during an ARI episode then the number of days will be considered missing.

For example, if 2 participants completed the 'Return to Usual Health' question only for 8 days as indicated in Table 11. For participant 1 the number of days to return to usual health is 5 (*15/11/2021- 11/11/2021+1*) and this will be considered an event. For participant 2, the number of days to return to usual health is 8 (*18/11/2021- 11/11/2021 + 1*) and this record will be censored.

Table 11: Example for 'Return to Usual Health' question

	ARI Day 1 Onset (11/11/2021)	ARI Day 2 (12/11/2021)	ARI Day 3 (13/11/2021)	ARI Day 4 (14/11/2021)	ARI Day 5 (15/11/2021)	ARI Day 6 (16/11/2021)	ARI Day 7 (17/11/2021)	ARI Day 8 (18/11/2021)
Participant 1	No	No	Yes	No	Yes	Yes	Yes	Yes
Participant 2	No	No	Yes	Yes	Yes	No	No	No

Note: in case a participant has two or more different responses at the same day, then the 'No' answer will be taken into account for calculating the number of days a participant took to return to his usual health.

6.6.7.2.1. Analysis Methods

PGI scores will be analyzed based on the PPE set, with a major focus in participants with RT-PCR-confirmed RSV ARI and LRTD, for the first and second year and the over whole study. Part of the analysis might also be performed for participants with RT-PCR-confirmed hMPV-mediated and influenza-mediated ARI.

For the **PGI-H** collected during an ARI, number of observations, mean, standard error, median, first and third quartile, minimum and maximum of the change from baseline will be tabulated and means with standard errors may be graphically presented per group and time point for the change from baseline.

For the PGI-H, descriptive statistics start and end of each year, as well as the change between the two timepoints will also be calculated for all participants based on the PPE set and for participants with RT-PCR-confirmed RSV ARI, based on the PPE set.

For the **PGI-C** and **PGI-S** scores collected during an ARI, number of observations, mean, standard error, median, first and third quartile, minimum and maximum will be tabulated and means with standard errors may be graphically presented per group and time point.

For the **AUCs** number of observations, median, first and third quartile (q1, q3), minimum and maximum will be calculated and graphically presented with boxplots per group.

The number of participants and days it took them to **return to their usual health** will be summarized and graphically presented with Kaplan Meier curves. Hazard ratios will also be calculated, with a Cox proportional hazard model with time to return to usual health as dependent variable and group as independent variable. Return to usual health will also be summarized by RSV subtype.

In case two or more assessments are reported at the same day then the worst total score will be used. The same applies for the AUC calculations.

In case a participant had multiple RT-PCR confirmed RSV-mediated ARIs or LRTDs during the reported period, then the PGI score or AUC corresponding to the first occurrence of RT-PCR confirmed RSV-mediated ARI or LRTD will be used, respectively.

6.6.7.3. EuroQoL, 5-Dimension, 5-Level Questionnaire

The EQ-5D-5L questionnaire is a standardized instrument for use as a measure of health outcome, primarily designed for self-completion by the participant. It consists of the **EQ-5D-5L descriptive system** and **EQ visual analogue scale** (VAS).

The **AUC for the change from baseline for EQ visual analogue scale** during the ARI will also be calculated.

The descriptive system includes the following dimensions: mobility, self-care, usual activities, pain/discomfort, and anxiety/depression. Each of these 5 dimensions is divided into 5 levels of perceived problems (1=no problem, 2=slight problems, 3=moderate problems, 4=severe problems; 5=extreme problems) (https://euroqol.org/eq-5d-instruments/).

Based on the descriptive system the **EQ-5D Valuation index** is calculated. This index summarizes the information of the 5 dimensions of the descriptive system.

- Assign the level code 1, 2, 3, 4 and 5 to each level of the 5 dimensions (see CTP Appendix 9)
- Create a health state for each patient-time point combination. A health state is a combination of 5 level codes: one level code for each dimension. E.g. health state 12543

indicates 'no problems walking, slight problems washing or dressing myself, unable to do my usual activities, severe pain or discomfort, moderately anxious or depressed'.

- Assign an index value to each health state as defined in Respiratory where EQ-5D health state is converted to a single summary index by applying a formula that essentially attaches weights to each of the levels in each dimension. The algorithm is based on the valuation of EQ-5D health states using the UK TTO (=time tradeoff method) based value set (see Section 7.12).

6.6.7.3.1. Analysis Methods

EQ-5D-5L scores will be analyzed based on the PPE set, with a major focus in participants with RT-PCR-confirmed RSV ARI and LRTD, RT-PCR-confirmed RSV non- LRTD, for the first, second year and over the whole study. Part of the analysis might also be performed for participants with RT-PCR-confirmed hMPV-mediated and influenza-mediated ARI.

The **EQ-5D-5L dimension** responses collected during an ARI, will be summarized descriptively per category, level and day relative to the start of the ARI for the two groups. Stacked bar plots will be used to visualize the EQ-5D-5L dimension.

For the **EQ visual analogue scale** collected during the ARI, number of observations, mean, standard error, median, first and third quartile, minimum and maximum will be tabulated and means with standard errors may be graphically presented per group and time point for the change from baseline.

For the **EQ-5D Valuation index** (See Section 7.12) collected during the ARI, number of observations, mean, standard error, median, first and third quartile, minimum and maximum will be tabulated and means with standard errors may be graphically presented per group and time point for the change from baseline.

For all three measures, descriptive statistics at the start and end of each year as well as the change between the two timepoints will also be calculated for all participants, for participants with RT-PCR-confirmed RSV ARI and for participants with RT-PCR-confirmed RSV LRTD, based on the PPE set.

For the **AUC** number of observations, median, first and third quartile (q1, q3), minimum and maximum will be calculated and graphically presented with boxplots per group.

Additionally, the vaccine efficacy of the first occurrence of RT-PCR-confirmed RSV-mediated LRTD will be calculated, based on the four quartiles of the EQ visual analogue scale collected at baseline for the PPE participants.

In case two or more assessments are reported at the same day then the worst total score will be used. The same applies for the AUC calculations.

For EQ VAS, worse is defined as the lowest number of the ones reported.

6.6.7.4. Days missed from work

The number of days missed from work will be summarized descriptively for participants that normally work with a RT-PCR confirmed RSV ARI, LRTD, non LRTD and Influenza and hMPV-mediated ARI for the first and second year, separately and over the whole study.

6.6.8. Medical Resources Use Questionnaire

Medical resource utilization questionnaire data are collected at ARI Day 29. The questionnaire contains 4 categories of medical resources and each of them has several subcategories (See Attachment 10.11 in CTP).

MRUs scores will be analyzed based on the PPE set, with a major focus on participants with RT-PCR-confirmed RSV-mediated ARI, LRTD, non LRTD and with influenza-mediated ARI and hMPV-mediated ARI for the first and second year separately, and over the whole study.

An emerging use of medical resources is defined as a medical resource visit that was related to an ARI or a complication of an ARI (meaning the 'Specify number of visits to ARI or its complications' question in the MRU is equal or larger to one and at least one of the visits should be set as related to an ARI or its complications).

Emerging medical resources utilized will be summarized per category, subcategory and per group. The number of visits and the length of stay will also be summarized per group, as well as the type of contact (home visit, appointment by telephone etc.). For hospital services, length of stay will also be calculated combined for 'Short term hospital admission', 'Hospitalization in general ward' and 'Hospitalization in critical Care'.

The proportion of participants in the PPE with emerging medical resource utilizations associated with an RT-PCR-confirmed RSV ARI, RSV-mediated LRTD, influenza-mediated ARIs and hMPV-mediated ARIs will also be summarized with the corresponding VE and will be depicted with forest plots. A similar Exact Poisson regression model as for the primary and secondary endpoints will be used and exact 2-sided 95% CIs for VE (1-relative risk rate) will be calculated. The follow-up time is determined in a similar way as for the primary and secondary endpoints.

Moreover, percentage of participants reporting that their interactions with healthcare providers for study -related visits and procedures affected the number of times they sought medical care outside of the study will be summarized. The type of the effect (increase or decrease of interactions with healthcare providers) will also be summarized.

In case a participant had multiple RT-PCR-confirmed ARIs or LRTDs during the reported period, then the MRU corresponding to the first occurrence of the RT-PCR-confirmed ARIs or LRTDs will be used, respectively.

6.6.9. Approximate ARI Surveillance Compliance

Participants will receive an ARI surveillance question twice per week questioning whether they have experienced any cold or respiratory infection symptoms. The responsiveness of the

participants to this ARI surveillance question will be summarized for both groups for the first year, second year, over the whole study and per risk level.

The approximate number of ARI surveillance questions that each participant is expected to have responded per year will be calculated as follows:

1. First the number of weeks between the start and the end of the year. The start of the first year is defined as the day of vaccination. The end of the first year is defined as the minimum of (the database cut-off date, a day prior to the study Day 365 [or relative day 365, if the Day 365 visit not available but there are ARI surveillance questions available after the relative day 365], date of study discontinuation). The start of the second year is defined as the Month 12 visit (see phases definition in section 6.1.2) and the end of the second year is defined as the minimum of (the database cut-off date, date of study discontinuation/completion).
2. Then:
   - for ARI that are not closed (not censored) by the PI, the number of weeks that the participant experienced an ARI episode between start and end of the year, will be calculated (= duration of the ARIs in weeks, see Section 6.3.1 for the duration definition).
   - For ARI that are closed (censored) by the PI, because the PI assessed that symptoms have permanently worsened, the number of weeks that the participant experienced an ARI episode between start and end of the year, will be calculated (= duration of the ARIs in weeks, see Section 6.3.1 for the duration definition).
   - for ARIs that are closed by the PI because they were not RSV, hMPV or Flu positive and no sputum was collected, then 1.4 weeks (10 days) will be used. Per CTP, these participants will return to ARI surveillance with twice weekly reminders 10 days after the day of onset of this ARI.

   Notes:
     - ARIs ending prior to the day of vaccination will not be taken into account.
     - In case an ARI starts in the first year and ends in the second year, the duration will be split between the two years. The duration from the start of the ARI until the end of the first year will be attributed to the first year, and the duration between the start of the second year and the end of ARI episode will be attributed to the second year. For example, if the ARI started 10 Aug 2022 and ended 23 Aug 2022, and first year ended 14 Aug 2022 and second year started 15 Aug 2022, the overall ARI duration will be 14 days, of which 5 days will be attributed to the first year and 9 days will be attributed to the second year.
     - For ARIs that are closed by the PI because they were not RSV, hMPV or Flu positive and no sputum was collected, min[(ARI start date +10 days – 1), last phase date] will be considered as their ‘temporary’ ARI end date,

instead of the end date reported in the CRF. Therefore, in case the ARI starts in the first year and the 'temporary' end date falls in the second year, the 10 days duration will be split between the two years, similar to an ARI not closed by the PI. For example, if an ARI closed by the PI started on the 10 Aug 2022, the first year ended 15 Aug 2022 and the second year started 16 Aug 2022, then the duration between the 10 August 2022 and the 15 August 2022 (= 6 days) will be attributed to the first year and the duration between the 16 August 2022 and 19 August 2022 (10 August 2022 + 10 days - 1) (4 days) will be attributed to the second year.

- In case a RSV+ ARI is ongoing at the time of the DB cut off, the date of the DB cut off will be considered as the 'temporary' end date of the ARI for the calculation of the ARI duration.
- In case an ARI episode starts prior to the day of vaccination, only the ARI duration after the vaccination will be used.

3. And finally, the difference of the two (1-2) will be multiplied by 2, since for each week a participant is part of the ARI surveillance journey, we expect to receive 2 questionnaires.

For estimating the percentage of compliance per subjects the number of ARI surveillance questions answered after vaccination, until the end of the year and outside RSV-episodes will be divided by the number obtained in step 3 and will be multiplied by 100.

In case multiple ARI surveillance questions have been answered at the same day, only one will be used.

The approximate number of ARI surveillance questions that each participant is expected to have responded over the whole study will be defined similar as per year, where in the first step the number of weeks between day of vaccination and the minimum of (the database cut-off date, date of study discontinuation, date of completion, end of first year in case of MPD impacting efficacy during the second year).

6.6.10. ARI Missing Data

The reason and number of days with ARI missing data will be analyzed based on the PPE set, with a major focus on any RT-PCR confirmed RSV, Influenza and hMPV-mediated ARI for the first year, second year, over the whole study and per risk level.

During the day of vaccination, at month 12, month 24 and during the ARI episodes the site will collect the reason for missingness in case there are missing ePro assessments. These reasons will be tabulated for all participants per assessment and per group, per timepoint. The reason of missingness for the different assessments will similarly be tabulated, per year. More than one reason might be reported during one ARI episode.

Moreover, based on the RiiQ assessments descriptive statistics of the number of days with missing assessments during the first 7 days of an ARI episode, the first 14 days of an ARI episode and during the full ARI episode will be summarized per group and depicted with stacked bar plots.
The start of the ARI (CESTDTC) will be used for this calculation.
Finally, the percentage of compliance based on the RiiQ assessments will be calculated as follows:

- Compliance over the whole episode is defined as: Nr of days with RiiQ assessments/duration of episode x 100
- Compliance during the first seven days is defined as: Nr of days with RiiQ assessments between day 1 and day 7/ min (duration episode, 7 days) x 100
- Compliance during the first 14 days is defined as: Nr of days with RiiQ assessments between day 1 and day 14/ min (duration episode, 14 days) x 100

For example: For a subject with an ARI episode duration of 21 days with missing RiiQ assessment on day 4, 8 and 19, the compliance over the whole episode is 18/21x100, the compliance during the 1st 7 days is 6/7x100, the compliance over the 1st 14 days is 12/14x100.

6.7. Safety Analyses

Safety analyses will be performed on the FAS. The analysis of solicited and unsolicited AEs will be restricted to the Safety Subset, whereas for the analysis of serious AEs (SAEs), Covid cases, potential adverse events of special interest (AESI) and potential AESI qualified for assessment the entire FAS population will be used. Solicited and unsolicited AEs accidently captured for participants outside the Safety Subset of the main cohort, will be listed.

Continuous variables will be summarized using the following statistics, as appropriate: number of observations, median, minimum and maximum. Frequencies and percentages will be generated for categorical variables. No formal comparisons between groups will be provided.

Safety data will be analyzed by vaccine regimens, by risk level and by age category (<75 years and ≥ 75 years) for the first and second year and over the whore study. Safety data may also be analyzed per country. For unsolicited AE, denominator for the percentages is the number of participants in the considered population and phase (or interval) for a certain regimen (incidence per 100 participants/phase) risk level and age category. For solicited AEs, the denominator for the percentages is the number of participants with data assessed by the PI in the considered population and phase for a certain regimen (incidence per 100 participants/phase), risk level and age category.

6.7.1. Adverse Events

6.7.1.1.1. Definitions

Solicited AEs shown in the tables are extracted from the investigator assessment pages (CE) of the CRF. For unsolicited AEs, only the AEs within the 28-day period following vaccination will be presented in the safety tables except for SAE, Covid cases and potential adverse events of special interest, which will be captured and tabulated in the outputs covering the whole study period. Unsolicited non-serious adverse events collected outside the 28-day period following a vaccination will be presented through listings.

Solicited administration site symptoms will be by definition considered as related to the study vaccine.

The severity of the AEs will be classified as grade 1 to 4. Solicited events of grade 0, not reported in the CE domain, will therefore not be reported in the AE analysis

Any ARI recorded as an (S)AE in the eCRF will be excluded from any AE analysis if it is a confirmed RSV infection by RT-PCR (nasal swabs and sputum sample, when available) based on the laboratory or if it is connected with a hospitalized confirmed RSV infection based on an FDA-approved RT-PCR test at the local laboratory. RT-PCR confirmed RSV-mediated ARIs are part of the efficacy evaluation and will be summarized separately from the safety.

For AESI analyses, the following subcategories are defined:

- Potential AESIs as identified by the investigator in the database
- Potential AESIs selected programmatically

  Those include all reported AEs that are identified by the selection rule:

  - SMQ (Standardised MedDRA Queries) = "EMBOLIC AND THROMBOTIC EVENTS (SMQ)"
    or
  - (SUB_SMQ1 = "HAEMATOPOIETIC THROMBOCYTOPENIA (SMQ)" and SCOPE in ("BROAD", "NARROW")) or HLT (higher level term)="Thrombocytopenias"

- Potential AESIs qualified for assessment
  Potential AESIs (programmed/CRF) that have risk levels assessed by one of the following three criteria are considered 'qualified for assessment':

  - Brighton Collaboration Level (Level 1-5)
  - CDC Tier (non-tier 1/ 2, tier 1, tier 2)
  - PRAC criteria (confirmed, possible, probable, unlikely, criteria not met)

6.7.1.1.2. Analysis of Adverse Events

Number and percentage of participants with at least one particular AE (unsolicited/solicited) will be tabulated. Unsolicited AEs will be summarized by System Organ Class and Preferred Term. Solicited AEs will be summarized by class (administration site, systemic) and preferred term. AESI analyses will be summarized by Interest Category and Preferred Term.

For solicited AEs following tables will be provided: summary, by worst severity grade, at least grade 3, related (systemic only), time to onset (in days) and duration (in days). Note: Duration is defined as number of days from the start of the event until resolution of the event. The time to first onset is defined as (date of first onset – reference date + 1). The reference date is the start date of the post dose period.

For unsolicited AEs following tables will be provided: summary table (including SAE, fatal outcome and vaccination discontinuation), all events, at least grade 3, permanent stop of vaccine and related. For SAEs following tables will be provided: all events, all related events, events with fatal outcome and related events with fatal outcome.

Moreover, tables with Covid AEs, potential AESI as identified by the investigator, potential AESIs selected programmatically and potential AESI qualified for assessment will be created.

Potential AESIs selected programmatically will be tabulated by categories: 'Embolic and thrombotic events (SMQ)' and 'Haematopoietic Thrombocytopenia (SMQ) (broad) or HLT = Thrombocytopenias'. Potential AESI determined programmatically, related to study vaccine (investigator assessment), will be tabulated similarly.

Potential AESIs as identified by the investigator, will be tabulated by categories: 'Embolic and thrombotic events (SMQ)' and 'Haematopoietic Thrombocytopenia (SMQ) (broad) or HLT = Thrombocytopenias', and 'Other'. Potential AESI as identified by the investigator, related to study vaccine (investigator assessment), will be tabulated similarly.

Potential AESIs qualified for assessment will be tabulated by categories: 'Embolic and thrombotic events (SMQ)' and 'Haematopoietic Thrombocytopenia (SMQ) (broad) or HLT=Thrombocytopenias'.

All potential AESI analyses will be presented by phase as well as by time interval. The definition of the different time intervals can be found in Section 6.1.2.

For potential AESIs analyses, attribution to the intervals will be done similarly to the unsolicited AEs as described in Section 6.7.1.1.3. For Step 2 of phase allocation of adverse events, the '0 - 28 days post-dose' interval should be treated similar to 'active' periods and the rest as 'non- active' periods.

Listings and/or participant narratives will be provided as appropriate, for those participants who die, discontinue study vaccinations due to an AE, experience a severe or serious AE or a COVID-19 infection or potential AESIs.

6.7.1.1.3. Phase Allocation of Adverse Events

As the analysis of solicited events will be based on the overall assessment of the investigator, which is documented in the CE domain, the ADAM (Analysis Data Model) dataset will be based on the CE domain. Solicited events are allocated to the phases as described below, however they are always allocated to the respective post-dose period and will never be attributed to the screening phase. Time of day is not considered while attributing solicited AEs to phases.

For unsolicited AEs, the steps below are followed as well.

Step 1: Allocation of events to the periods:

Adverse events in the SDTM database are allocated to periods based on their start date/time. If the start date/time of an event falls between (or on) the start and stop date/time of a period, the AE is attributed to that period (emergent principle).

- In case of partial start or stop dates (i.e. time and/or day and/or month and/or year missing), the events are allocated to the periods using the available partial information on start and end date; no imputation will be done. If, for instance, the AE start date only month and year are available, these data are compared to the month and year information of the periods. This rule may lead to multiplication of the event as a consequence of its assignment to multiple periods.

- In case of a completely missing end date, the date is imputed by the cut-off date of the analysis for participants still ongoing in the study, and by the end date of the last period for participants who discontinued or completed the trial. In case of a completely missing start date, the event is allocated to the active treatment phase (post dose period), except if the end date of the AE falls before the start of the active treatment phase (post dose period).

Step 2: Combination of events:

Overlapping/consecutive events are defined as events of the same participant with the same preferred term which have at least 1 day overlap or for which the start date of an event is 1 day after the end date of the preceding event. Overlapping/consecutive events may be combined into one AE or not, according to the following rules:

1) If overlapping/consecutive events start in one of the following phases/periods - Screening or Follow-up (defined as non-active periods) - followed by an AE in - Post-dose period (defined as active period) - they are allocated to their respective phases/periods and are considered as separate events.

2) In case overlapping/consecutive events start within a single period, they are considered as one and the same AE. The individual events which contribute to this AE are retained as individual records in the ADaM database but are assigned the same onset, period, and total duration. All related attributes to the AE/phase/period should also be consistent with the new event.

3) In case overlapping/consecutive events start in both an active period followed by a consecutive non-active period, they are allocated to the active period only and are considered as one and the same AE. The individual events which contribute to this AE are retained as individual records in the ADaM database but are assigned the same onset, treatment period, and total duration. All related attributes to the AE/phase/period should also be consistent with the new event.

4) In case an active period is followed by another active period, and the overlapping/consecutive events start in both periods, they are allocated to their respective period and are considered as separate AEs. In case overlapping/consecutive events start in non-consecutive periods (regardless

of active or non-active), they are allocated to their respective period and are considered as separate AEs.

5) In case a non-active period is followed by another non-active period, and the overlapping/consecutive events start in both periods, they are allocated to the first period and they are considered as one and the same AE. The individual events which contribute to this AE are retained as individual records in the ADaM database but are assigned the same onset, period, and total duration. All related attributes to the AE/phase/period should also be consistent with the new event.

Remarks:
1. Events can only be combined into one and the same AE if their start and stop dates are known.
2. In case the completely missing end date is imputed (for period allocation), this date is also considered as a complete date.
3. Time is not considered when determining overlap of events.

6.7.1.1.4. Missing Data

Missing data (grade, relationship) will not be imputed. Participants who do not report an event will be considered as participants without an event. An AE with a missing severity or relationship will be considered as an AE reported but will be considered as not reported for the severity or relationship. For example, an AE with missing severity will be considered as an AE reported for the analysis of any grade but will be considered as not reported for the analysis of at least grade 3. The analysis of solicited AEs will include the safety data as documented by the investigator.

6.7.2. Vital Signs

Emerging vital sign abnormalities will be tabulated based on the abnormality gradings in Section 7.6. An abnormality will be considered as emerging if it is worse than the baseline value. If the baseline is missing, the abnormality is always considered emerging.

6.8. Other Analyses

6.8.1. Immunogenicity

The analysis of immunogenicity will use the PPI set. Key immunogenicity assay results will also be analyzed for the subgroups defined in Section 6.8.2.

Immunogenicity analysis will focus on the immuno subset. Immunogenicity analysis related to correlate of protection will be described in a separate SAP.

6.8.1.1. Parameters

Humoral and cellular immune responses against the insert are measured in the immunosubset. The measured humoral immune responses include titers of neutralizing antibodies and binding antibody titers (ELISA). The measured cell-mediated immune responses include RSV specific INFγ ELISpot responses and intracellular cytokine staining (ICS) if available. Immunogenicity against the vector will be explored using an adenovirus neutralization assay.

6.8.1.2. Handling of Missing and/or Unquantifiable Immune Response Data

Missing immune response data will not be imputed.

Values below the lower limit of quantification (LLOQ) will be treated differently according to the assay:

- For all humoral assays:
  - If the assay has only LLOQ: Values below LLOQ will be imputed to LLOQ/2, except for the calculation of the geometric mean of the increase from baseline, where values below LLOQ will be imputed to LLOQ.
  - If the assay has LLOQ and LOD: Values below LOD will be imputed to LOD/2, except for the calculation of the geometric mean of the increase from baseline, where values will be imputed to LOD. Values between LOD and LLOQ will be imputed to the midpoint between LOD and LLOQ for all analysis.
- For ELISpot assays: the LLOQ will be used if available and validated. For descriptive statistics or graphs on actual values, values below the LLOQ will be imputed to a value of LLOQ/2.
- For ICS assays: the LLOQ will be used if available and validated. In case no validated LLOQ is available then a provisional cut-off will be provided before DBL (only for total cytokine response), in the database. For the individual cytokine combinations of INFγ, TNFa and IL2, if available, negative values will be imputed with 0. For descriptive statistics or graphs on actual values, values below the LLOQ will be imputed to a value of LLOQ/2.

Data above the ULOQ will always be imputed with the ULOQ.
The ULOQ and LLOQ values per assay will be available in the database.

6.8.1.3. Immune Response Analysis

No formal hypothesis on immunogenicity will be tested.

6.8.1.4. Immunogenicity Against the Insert:

6.8.1.4.1. Humoral assays

For **VNA** and **ELISA** assays following results will be calculated: N, geometric mean[§] and corresponding 95% CI of the actual values and fold increases from baseline will be tabulated and graphically presented. [§]*calculate the mean and corresponding 95%CI of the $\log_2$ transformed values, back-transform this mean [i.e. 2^mean] and CI [i.e. 2^CI].*

Actual values and fold changes from baseline are tabulated and shown as dot plots with dots for participant values, and the corresponding geometric mean and 95% CI per time point for each assay. In addition, Geometric Mean Titers (GMT) plots over time, combining the regimens in one graph (without individual participant dots) will also be created.

The percentage of participants with a 2-fold and a 4-fold increase from baseline might also be tabulated for some assays.

Reverse distribution curves of the actual values are provided for selected time points.

In the graphs, original values will be displayed on the $\log_2$ scale.

Scatterplots with the VNA versus ELISA (fold rises) will be provided for the most important time points. A scatterplot will also be created for the actual values comparing the different ELISA responses. In these scatterplots the actual values will be shown, even if they are below the LLOQ, but the LLOQ cut-off will be visualized in the graph per assay if some values are below LLOQ. The impact of baseline factors on the humoral and cellular responses will be explored graphically or via descriptive statistics.

Other exploratory parameters may be analyzed at the discretion of the sponsor.

6.8.1.4.2. Cellular assays

For **ELISpot** following results will be calculated: N, median and quartiles will be tabulated and graphically presented.

Tables with the corresponding descriptive statistics will be provided.

Actual values are shown as box plots with dots for participant values, and the corresponding median and interquartile range per time point for each assay. In addition, box plots over time, combining the regimens in one graph (without individual participant dots) will also be created. For the graphs, original values will be displayed on the $\log_{10}$ scale.

For **ICS** and **PBMC secreted cytokines** (if available) possible analyses may include:

Total Cytokine response: the % of subsets expressing at least IFNγ, TNFa or IL2 will be calculated for CD4 and CD8.

Tables with the corresponding descriptive statistics will be provided.

Actual values are shown as box plots with dots for participant values, and the corresponding median and interquartile range per time point for each assay.

In addition, box plots over time, combining the regimens in one graph (without individual participant dots) will also be created.

The distribution of the different cytokine combinations (the proportion of a specific cytokine combination of the CD4 or CD8 T-cells secreting at least one cytokine) will be represented graphically using bar charts or other graph forms. Tables with the corresponding descriptive statistics will be provided.

For the graphs, original values will be displayed on the $\log_{10}$ scale.

Scatterplot with humoral and cellular assays may be provided for the most important time points.

Technical details for the calculation of the ICS values to be used in the graphs will be part of the DPS.

6.8.1.5. Immunogenicity Against the Vector

For **Ad26-specific VNA** following statistics will be calculated: N, geometric mean. [§]*calculate the mean and corresponding 95%CI of the log_{10} transformed values, back-transform this mean [i.e. 10^mean] and CI [i.e. 10^CI].* and corresponding 95% CI of the actual values.

Geometric mean and 95% CI per time point will be calculated.

Participant profiles of the assays against the insert will be created, highlighting participants with pre-existing immunity at the day of vaccination (Day 1) against the vectors.

Scatterplots with the Adeno assays versus the assays against the inserts will be provided for the most important time points. In these scatterplots the actual values will be shown, even if they are below the LLOQ.

For the graphs, Ad26-specific VNA values will be displayed on the log_{10} scale.

6.8.2. Definition of Subgroups

The following subgroups will be investigated for the primary endpoint and for key immunogenicity assays:

- Age categories
  - (60-64 years, 65-74 years, 75-84 years, ≥85 years),
  - <65 years and ≥65 years
  - <75 years and ≥75 years
  - This is based on the age as collected in the CRF (not as stratified)
- Risk Level (CDC definition)
  - (increased risk/ non-increased risk)
  - This is based on the risk level as collected in the CRF (not as stratified)
- Risk Level, (CDC definition + Chronic Kidney Disease [CKD] + Diabetes)
  - (increased risk/ non-increased risk)
  - based on CDC definitions and the medical history terms (See Section 7.13).
- Sex
- Region (North America, Europe, South America, Asia if applicable, Australia/New Zealand, Africa)
- Race
- Smoking status

- Note: participants using only nicotine patches or gums will not be considered as smokers for the subgroup analysis.

- BMI
  - <18.5 kg/m2, 18.5-<25 kg/m2, 25-<30 kg/m2, >=30 kg/m2
- COVID-19 vaccination status at baseline (used for efficacy subgroup analysis)
  - Janssen Covid-19 vaccine
  - AstraZeneca COVID-19 vaccine
  - Other COVID-19 vaccination or no vaccination

– Participants can be part of only one of the three categories, based on the following rules: If a participants received at least one dose of Janssen Covid-19 vaccine, will be counted in the ‘Janssen Covid-19 vaccine’ category. If a participants received at least one dose of AstraZeneca Covid-19 vaccine and no doses of Janssen Covid-19 vaccine, will be counted in the ‘AstraZeneca Covid-19 vaccine’ category. All other participants will be counted in the last category (‘Other COVID-19 vaccination or no vaccination’).

- HIV status, based on medical history
- Based on baseline EQ-5D-5L VAS quartiles (see Section 6.6.7.3)

Demographic characteristics, study disposition, protocol deviations, safety, immunogenicity, and efficacy might also be analyzed by risk level and by age category (<75 years and ≥ 75 years).

Additional exploratory subgroup analysis, for example by age category may be performed.

6.9. Interim Analyses

6.9.1. Data Monitoring Committee (DMC)

An Independent DMC (IDMC) will be installed to monitor the safety of participants in the ongoing VAC18193 Phase 3 studies.

The IDMC will receive monthly safety updates, starting 6 weeks post first participant dosed until the day that the last participant dosed in the safety subset reached his/her 28- day post dose visit, and updates every 2 to 3 months after that until the end of the study. These reviews would be based on a snapshot of the database which might not have been completely cleaned. Data will be cleaned on an ongoing basis. The IDMC will review unblinded data; the data package to be reviewed (summary data) will display the real vaccine identity. The study team will transfer the blinded data to the statistical support group (SSG), and the IWRS vendor or Secure Data vendor will securely transfer the unblinded randomization data to the SSG. In principle there will be no meeting, unless this is requested by one of the IDMC members or by the Sponsor.

In addition, the IDMC will also formally monitor the efficacy endpoints at the timepoints specified in Section 9.5, Planned Analyses of the CTP. The IDMC will evaluate in an unblinded fashion if the success criteria have been met (See Section 2).

The IDMC will assess the null hypothesis of LL ≤20% for RT-PCR-confirmed RSV-mediated LRTD versus the alternative hypothesis of LL >20%. For vaccine efficacy to be demonstrated additionally, the observed VE should be >50%. Refer to Section 2 for details on the hypothesis testing.

Conclusions from the IDMC reviews will be communicated to the sponsor.

The IDMC data package (summary data) will follow the same statistical methods described in this SAP. The IDMC safety data package will consist of the following tabulations: participant disposition and demographics, SAEs, related SAEs, AEs with fatal outcome, related AEs with fatal outcome, solicited and unsolicited grade 3 AEs, related AEs, AEs leading to discontinuation. Potential AESI qualified for assessment and potential AESI will be listed. Other safety summaries might be requested as well.

The IDMC efficacy evaluation package will contain one table summarizing the vaccine efficacy and corresponding confidence interval.

A separate IDMC DPS document will be provided to describe the specifications of the individual tables to be generated should a safety issue arise.

Data packages will be distributed by the SSG to the IDMC members via a secure electronic environment. A separate data package might be made available to the study team where the summary data are presented for the pooled groups (blinded).

More details regarding the IDMC roles, responsibilities and way of working are included in the IDMC Charter (Section 5). This SAP will be used to support any IDMC analyses.

6.10. Additional Analyses

6.10.1. Efficacy Analyses

First occurrence of RT-PCR-confirmed RSV-mediated ARI, with onset between 14 days post vaccination according to the following two case definitions:

Table 12: Case Definitions

Case Definition #2	Case Definition #3
≥2 symptoms of LRTI<br><br>(new onset or worsening)	≥2 symptoms of LRTI,<br>*OR*<br>≥1 symptom of LRTI<br>*combined with*<br>≥1 systemic symptom (fatigue or feeling feverish)<br><br>(new onset or worsening)
RT-PCR confirmation of RSV	

LRTI = lower respiratory tract infection

These case definitions are defined similarly to the programmed definition. See section 6.3.1 for more details.

The VEs will be calculated for the above endpoints, occurring during the first year, during the second year and over the whole study, similar to all other efficacy endpoints (See Section 6.5.1). The PPE set will be used for the analysis of these exploratory endpoints.

6.10.2. Safety Analyses

SAE analyses will also be presented by time interval. The definition of the different time intervals can be found in Section 6.1.2.

AEs of interest and AEs of (Clinical) interest are also defined and will be tabulated.

Additionally, the following analyses will be added per FDA request:

- immediate reactions will be tabulated by worst severity grade
- the number of events in addition to the number of participants, already presented, will be tabulated for unsolicited AEs, SAEs and AEs of (special) interest.

Immediate reactions:

All solicited AE tables are based on the overall assessment by principal investigator, extracted from the investigator assessment pages (which spans the evaluation period since vaccination) of the CRF.

In response to FDA comments, immediate reactions should also be tabulated. Therefore, on top of the solicited AE tables based on the overall assessment by the principal investigator, a table by worst severity grade will be created focusing on the 15- or 30-minute on-site assessment post vaccination. In China the on-site assessment was planned at 30 minutes post-vaccination, in all other countries it was planned at 15 minutes post vaccination.

Note that for Erythema and Swelling the grades for immediate reactions are only captured in the FA domain with a diameter. The grades should be derived from the diameter as indicated below, grade 4 is not determined as this is not based on the diameter.

Table 13: Grades for erythema and swelling based on diameter

Diameter	Grade
25-50 mm	Grade 1
>50-100 mm	Grade 2
>100 mm	Grade 3

The 15- or 30-minute on-site assessment for pyrexia is only captured in the vital signs domain, grades should be based on the temperature captured as indicated in the table below:

Table 14: Grades for pyrexia based on temperatures

Temperature (°C)	Temperature (°F)	Grade
38.0-38.4°C	100.4-101.1°F	Grade 1
38.5-38.9°C	101.2-102.0°F	Grade 2
39.0-40.0°C	102.1-104.0°F	Grade 3
>40.0°C	>104.0°F	Grade 4

Counting the Number of Events

When counting the number of events, overlapping/consecutive events that are combined into one AE according to section 6.7.1.1.3 are counted as one event. The number of events will only be shown for subset of tables.

Adverse Events of Interest and Adverse Events of Clinical Interest

The below table provides the AEs of (clinical) interest and their selection criterium.

Table 15: Adverse Events of (clinical) interest

AE of (Clinical) Interest	AEI or AECI	Selection Criteria (SMQ, HTL, PT)
Myocarditis	AEI	HLT=”Noninfectious myocarditis”
Pericarditis	AEI	HLT=”Noninfectious pericarditis”
Capillary leak syndrome	AEI	PT=“Capillary leak syndrome” or PT=“Capillary permeability increased”
Guillain-Barre syndrome	AEI	SMQ="Guillain-Barre Syndrome (SMQ)" and scope=“NARROW”
Immune-mediated conditions	AEI	SMQ=“Immune-Mediated/Autoimmune Disorders (SMQ)” and scope=“NARROW”
Facial nerve disorders	AEI	PT in ("Bell’s palsy”, “Crocodile tears syndrome”, “Facial nerve disorder”, “Facial paralysis”, “Facial paresis”, “Facial spasm”, “Oculofacial paralysis”, “VIIth nerve injury”) (note : all PT are part of HLT “Facial cranial nerve disorders”)
VTE (narrow)	AECI	PT in (‘Pulmonary Embolism’ ‘Deep Vein Thrombosis’)
VTE (broad)	AECI	See Appendix 14 List of preferred terms for VTE (broad)
Arterial Thrombotic Events	AECI	based on sub_SMQ1 “Embolic and thrombotic events, arterial [SMQ]”
Pneumonia	AECI	PT=’Pneumonia’
Thrombocytopenia	AECI	SUB_SMQ1=“Haematopoietic thrombocytopenia (SMQ)” and scope=”NARROW” or HLT=“Thrombocytopenias”

AEI=AE of Interest, AECI=AE of Clinical Interest

Finally, for events with a completely missing start date, the event is allocated per SAP rule to the active treatment phase (post dose period), except if the end date of the AE falls before the start of the active treatment phase (post dose period). If this happens for AEs of (clinical) interest, a sensitivity analysis might be performed, allocating the event to only the follow up phases.

6.10.3. Demographics

Age will also be summarized based on the following age categories 60-69 years, 70-79 years and above 80 years old.

6.11. Discontinuation of the study

On the 29th of March 2023 the EVERGREEN study was permanently terminated. All participants are to be followed up for 6 months post vaccinations for safety collection. ARIs will not be collected after the 29th of March 2023 or followed up.

Due to the early termination of the study, the Sponsor decided not to perform the complete planned analysis as described in the protocol and SAP. The final analysis will contain the following:

- Efficacy analysis for secondary endpoints and for a limited selection of the exploratory endpoints.
- Safety analysis presented only per period (as defined in Table 4).
- Immunogenicity analysis of all completed assays in the database at the time of the analysis.

Moreover, no local specific cohort analysis will be conducted.

The following efficacy endpoints will be analyzed per year and over the whole study:

- First occurrence of any RT-PCR-confirmed RSV-mediated LRTD and ARI
- First occurrence of any RT-PCR-confirmed RSV-mediated LRTD and ARI by subtype
- First occurrence of any clinically relevant diseases associated with RT-PCR-confirmed RSV-mediated LRTD and ARI
- AUC for participants with any RT-PCR-confirmed RSV-mediated LRTD and ARI
- Viral loads

Note: In the cases that an RSV ARI is ongoing during the time of the discontinuation and does not have an end date, the 29th of March 2023 will be used as an ARI end date. An ARI with no end date will be considered as an RSV ARI, if an RSV positive result is available within 15 days after the ARI onset.

7. SUPPORTING DOCUMENTATION

7.1. Appendix 1 List of Abbreviations

ADaM	Analysis Data Model
AE	adverse event
AESI	adverse event of special interest
ATC	anatomic and therapeutic class
BCC	Brighton Collaboration Case Definition
BMI	body mass index
CD4	cluster of differentiation 4
CD8	cluster of differentiation 8
CDC	Center for Disease Control and Prevention
CEC	Clinical Event Adjudication Committee
CI	confidence interval
CRF	case report form
CTP	clinical trial protocol
IDMC	Independent Data Monitoring Committee
ECG	electrocardiogram
ELISA	enzyme-linked immunosorbent assay
ELISpot	enzyme-linked immunospot
FAS	full analysis set
FDA	Food and Drug Administration
FEV1	forced expiratory volume in 1 second
FEV6	forced expiratory volume in 6 second
FVC	forced vital capacity
GMC	geometric mean antibody concentration
GMT	Geometric Mean Titre
HR	hazard ratio
HLT	higher level term
ICF	informed consent form
ICS	intracellular cytokine staining
IFNg	interferon gamma
IL2	interleukin 2
IRR	incidence rate ratio
ITT	intent-to-treat
LLOQ	lower limit of quantification
LOD	lower limit of detection
NA	not applicable
PBMC	peripheral blood mononuclear cells
PPE	per protocol efficacy analysis set
PPI	per protocol immunogenicity analysis set
PRAC	Pharmacovigilance Risk Assessment Committee
SAE	serious adverse event
SAP	Statistical Analysis Plan
SD	standard deviation
SDTM	Study Data Tabulation Model
SE	standard error
SFU	spot forming units
SMQ	Standardised MedDRA Queries
TNFa	tumor necrosis factor alpha
ULOQ	upper limit of quantification
VE	vaccine efficacy
VNA	virus neutralizing antibody
VTE	Venousthromboembolism
WHO	World Health Organization

7.2. Appendix 2 Changes to Protocol-Planned Analyses

In the section 6.4.1.2, α_4 was added in the following sentence: The VE against the primary endpoint will be tested using the significance level α' (α_1 or α_2 or α_3 or α_4), as defined in Section 6.3.2.

The following exploratory endpoint was added:

- First occurrence of predefined clinically relevant disease associated with RT-PCR-confirmed RSV-mediated ARI and LRTD

This was already a secondary endpoint for the end of study analysis.

The following exploratory analysis was added:

- First occurrence of RT-PCR-confirmed RSV-mediated LRTD will be calculated, based on the four quartiles of the EQ visual analogue scale collected at baseline for the PPE participants

7.3. Appendix 3 Demographics and Baseline Characteristics

The following demographic and baseline characteristics will be summarized.

Table 16 presents a list of the demographic variables that will be summarized by vaccine regimen and overall, for the for the full analysis set and by vaccine regimen for safety subset, PPE, PPI and for participants with an RT-PCR confirmed RSV-mediated ARI as well based on the risk level of the participant. Demographics will also be summarized by region using the FAS analysis set. Demographics may also be summarized by age category.

Table 16: Demographic Variables

**Continuous Variables:**	**Summary Type**
Age (years)	Descriptive statistics (N, median , mean, standard deviation, minimum and maximum).
Weight (kg)	
Height (cm)	
Body Mass Index (BMI) (kg/m^2)	
**Categorical Variables**	
Age categories (60-64 years, 65-74 years, 75-84 years, ≥85 years)	Frequency distribution with the number and percentage of participants in each category.
Sex (male, female, intersex)	
Race[a] (American Indian or Alaska Native, Asian, Black or African American, Native Hawaiian or other Pacific Islander, White, Multiple)	
Ethnicity (Hispanic or Latino, not Hispanic or Latino)	
Risk Level (CDC definition), as collected	
COPD (for increased risk)	
CHF (for increased risk)	
Asthma (for increased risk)	
other chronic heart disease (for increased risk)	
other chronic lung disease (for increased risk)	
Risk Level (CDC definition + CKD + Diabetes)	
BMI (underweight <18.5 kg/m2, normal or healthy weight 18.5-<25 kg/m2, overweight 25-<30 kg/m2, obese >=30 kg/m2)	
Region (Northern America, Europe , South America, Asia, Australia/New Zealand, Africa)	
Country	
Smoking status	
HIV status	
COVID-19 vaccination status at baseline .<br>Participants receiving at least one dose of:<br>• AstraZeneca COVID-19 vaccine<br>• Janssen Covid-19 vaccine<br>• Moderna COVID-19 vaccine<br>• Pfizer COVID-19 vaccine<br>• Other COVID-19 vaccine<br>• No COVID-19 vaccination<br>– (participants might be counted in multiple categories)	

[a] If multiple race categories are indicated, the Race is recorded as 'Multiple'

7.4. Appendix 4 Protocol Deviations

Major protocol deviations will be summarized per year, per vaccine group, increased level subgroup and overall.

In general, a list of major protocol deviations may have the potential to impact participants' rights, safety or well-being, or the integrity and/or result of the clinical study. Participants with major protocol deviations will be identified prior to database lock and the participants with major protocol deviations will be summarized by category. In addition, minor and major protocol deviations related to COVID-19 will be tabulated.

7.5. Appendix 5 Prior and Concomitant Medications

The analysis of concomitant therapies will be done using the WHO drug coded terms. Concomitant medications associated with SAE will be collected through the study, as well as concomitant medications associated with ARI episodes and with complications of ARI will be recorded for all participants during all ARI episodes for the duration of the two study years.

Based on their start and stop date, concomitant therapies will be reported in each applicable phase.

If a concomitant therapy record misses components of its start and/or stop dates (time, day and/or month and/or year):

- In case of partial start or stop dates, the concomitant therapy records will be allocated to periods using the available partial information, without imputations. If, for example, only month and year are available, these will be compared to the month and the year of the periods, and the concomitant therapy record will be allocated to the period(s) where these date parts match. This rule may lead to assignment to multiple periods. The same rule applies for identifying whether a concomitant therapy was administered during 8 days following a vaccination. If for example, the vaccination was administered on the 30 December 2017 and the concomitant therapy start date is January 2018, then the concomitant therapy will be assumed to have started within 8 days of the vaccination.
- In case of a completely missing start date, the concomitant therapy will be considered as having started before the study.
- In case of a completely missing end date, the concomitant therapy will be considered as ongoing at the end of the study.

For the use of analgesics/antipyretics (definition below) which are taken on the day of vaccination, an exception is made in case the time is before vaccination. In this case, the concomitant medication is also allocated to the post-dose period.

There will be special attention to:

- analgesics/antipyretics such as acetaminophen, non-steroidal anti-inflammatory drugs (NSAIDs) and aspirin, that started being administered during 8 days following the vaccination (00:00 of day of vaccination + 7 days), based on the safety subset. The following codes will be used for this: N02A (OPIOIDS) and N02B (OTHER ANALGESICS AND ANTIPYRETICS), M01A (ANTIINFLAMMATORY AND ANTIRHEUMATIC PRODUCTS, NON-STEROIDS) and M01B (ANTIINFLAMMATORY/ANTIRHEUMATIC AGENTS IN COMBINATION) (ATC/DD Index). The classes will be added in a footnote in all related tables and listings
- all concomitant medications for the safety subset
- concomitant medications related to SAEs, based on the FAS
- emerging therapeutic interventions of interest associated with RT-PCR confirmed RSV-mediated ARI episodes and with complications of RT-PCR confirmed RSV-mediated ARI based on the PPE set.

- new concomitant medications that are not part of the therapeutic interventions of interest associated with RT-PCR confirmed RSV-mediated ARI episodes and with complications of RT-PCR confirmed RSV-mediated ARI based on the PPE set.

New concomitant medications and emerging therapeutic interventions are defined as medications not available at baseline or medication with an increased dosage, compared to baseline and are indicated by the principal investigator.

7.6. Appendix 6 Guidance for Industry: Toxicity Grading Scale for Healthy Adult and Adolescent Volunteers Enrolled in Preventive Vaccine Clinical Trials

Adapted from the FDA Guidance document "Toxicity Grading Scale for Healthy Adult and Adolescent Volunteers Enrolled in Preventive Vaccine Clinical Trials" (September 2007)

**Vital Signs ***	**Mild (Grade 1)**	**Moderate (Grade 2)**	**Severe (Grade 3)**	**Potentially Life-threatening (Grade 4)**
Fever (°C) **<br>(°F)**	38.0-38.4<br>100.4-101.1	38.5-38.9<br>101.2-102.0	39.0-40.0<br>102.1-104.0	>40<br>>104.0
Tachycardia - beats per minute	101-115	116-130	>130	Hospitalization for arrhythmia[#]
Bradycardia - beats per minute***	50-54	45-49	<45	Hospitalization for arrhythmia[#]
Hypertension (systolic) - mm Hg	141-150	151-160[#]	>160[#]	Hospitalization for malignant hypertension[#]
Hypertension (diastolic) - mm Hg	91-95	96-100	>100	Hospitalization for malignant hypertension[#]
Hypotension (systolic) - mm Hg	85-89	80-84	<80	Hospitalization for hypotensive shock[#]
Respiratory Rate - breaths per minute	17-20	21-25	>25	Intubation

* Participant should be at rest for all vital sign measurements.
** For oral temperature: no recent hot or cold beverages or smoking.
*** When resting heart rate is between 60 - 100 beats per minute. Use clinical judgment when characterizing bradycardia among some healthy participant populations, for example, conditioned athletes.
[#] Revised by the sponsor.

For the vital signs' analysis in Section 6.6.5 only values will be used to assign abnormalities, no clinical interpretations will be used. Therefore, grade 3 and 4 will be combined because grade 4 always requires clinical interpretation.

7.7. Appendix 7 Respiratory Viruses and Bacteria Used to Define Coinfections

The following respiratory viruses will be evaluated by the BioFire® Filmarray RP2.1-EZ on the nasal swab:

- Adenovirus
- Coronavirus 229E
- Coronavirus HKU1
- Coronavirus NL63
- Coronavirus OC43
- Coronavirus SARS-CoV-2
- Human Metapneumovirus
- Rhinovirus/Enterovirus
- Influenza A, including subtypes H1, H3 and H1-2009
- Influenza B
- Parainfluenza Virus
- Respiratory Syncytial Virus

The following respiratory bacteria will be evaluated by the BioFire® Filmarray RP2.1-EZ on the nasal swab:

- Bordetella parapertussis
- Bordetella pertussis
- Chlamydia pneumoniae
- Mycoplasma pneumoniae

The following based on the BioFire Filmarray PN assay from the sputum sample:

- Adenovirus
- Coronavirus
- Human Metapneumovirus
- Human Rhinovirus/Enterovirus
- Influenza A
- Influenza B
- Parainfluenza Virus
- Respiratory Syncytial Virus

The following respiratory bacteria will be evaluated by the BioFire Filmarray PN assay from the sputum sample:

- Acinetobacter calcoaceticus-baumannii complex
- Enterobacter cloacae complex
- Escherichia coli
- Haemophilus influenzae
- Klebsiella aerogenes
- Klebsiella oxytoca

- Klebsiella pneumoniae group
- Moraxella catarrhalis
- Proteus spp.
- Pseudomonas aeruginosa
- Serratia marcescens
- Staphylococcus aureus
- Streptococcus agalactiae
- Streptococcus pneumoniae
- Streptococcus pyogenes
- Chlamydia pneumoniae
- Legionella pneumophila
- Mycoplasma pneumoniae

For samples collected in China, the following respiratory viruses will be evaluated by the Cepheid GeneXpert on the nasal swab:

- Influenza A
- Influenza B
- Respiratory Syncytial Virus

7.8. Appendix 8 Clinically Relevant Complication AE terms

**Asthma & COPD & bronchial hyperreactivity (Preferred terms)**
Acute post asthmatic amyotrophy
Asthma late onset
Obstructive airways disorder
Asthma
Bronchospasm
Asthma exercise induced
Status asthmaticus
Asthma-chronic obstructive pulmonary disease overlap syndrome
Asthmatic crisis
Wheezing
Bronchial hyperreactivity
Bronchial obstruction
Bronchostenosis
Obliterative bronchiolitis
Bronchiolitis obliterans syndrome
Bronchitis chronic
Childhood asthma
Chronic obstructive pulmonary disease
Cough variant asthma
Infective exacerbation of chronic obstructive airways disease
Occupational asthma
Reactive airways dysfunction syndrome
Reversible airways obstruction
Unilateral bronchospasm
Asthma
Bronchitis chronic
Obstructive airways disorder

**Respiratory Distress (Preferred Terms)**
Respiratory Distress

**Bronchitis (Preferred Terms)**
Bronchitis
Bronchitis bacterial
Tracheobronchitis
Bronchitis viral
Allergic bronchitis
Pertussis
Bronchitis chemical
Bronchitis fungal
Bronchitis haemophilus
Bronchitis moraxella
Bronchitis mycoplasmal
Bronchitis pneumococcal
Coxsackie bronchitis
Fibrinous bronchitis
Enterobacter tracheobronchitis
Eosinophilic bronchitis
Herpes simplex bronchitis
Tracheobronchitis mycoplasmal
Noninfective bronchitis
Parainfluenzae viral bronchitis
Parainfluenzae viral laryngotracheobronchitis
Pseudomonas bronchitis
Respiratory syncytial virus bronchitis
Sinobronchitis

**Bronchitis (Preferred Terms)**
Streptococcal bronchitis
Tracheobronchitis bacterial
Tracheobronchitis viral

**CHF Exacerbation (Preferred Term)**
Cardiac failure congestive

**Cardiac Arrhythmia (Preferred Terms)**
Chronotropic incompetence
Bezold-Jarisch reflex
Cardiac arrest
Cardiac death
Cardiac telemetry abnormal
Cardio-respiratory arrest
Electrocardiogram abnormal
Electrocardiogram ambulatory abnormal
Heart rate abnormal
Palpitations
Respiratory sinus arrhythmia magnitude abnormal
Respiratory sinus arrhythmia magnitude decreased
Respiratory sinus arrhythmia magnitude increased
Bradyarrhythmia
Ventricular asystole
Accessory cardiac pathway
Adams-Stokes syndrome
Agonal rhythm
Atrial conduction time prolongation
Atrioventricular block
Atrioventricular block complete
Atrioventricular block first degree
Atrioventricular block second degree
Atrioventricular conduction time shortened
Atrioventricular dissociation
Atrioventricular node dysfunction
Bifascicular block
BRASH syndrome
Brugada syndrome
Bundle branch block
Bundle branch block bilateral
Bundle branch block left
Bundle branch block right
Conduction disorder
Defect conduction intraventricular
Electrocardiogram delta waves abnormal
Lenegre's disease
Long QT syndrome
Paroxysmal atrioventricular block
Sinoatrial block
Trifascicular block
Ventricular dyssynchrony
Wolff-Parkinson-White syndrome
Nodal arrhythmia
Sinus arrest
Sinus arrhythmia
Arrhythmia
Heart rate irregular

Cardiac Arrhythmia (Preferred Terms)
Holiday heart syndrome
Pacemaker generated arrhythmia
Pacemaker syndrome
Paroxysmal arrhythmia
Reperfusion arrhythmia
Withdrawal arrhythmia
Atrial fibrillation
Atrial flutter
Atrial parasystole
Congenital supraventricular tachycardia
Frederick's syndrome
Junctional ectopic tachycardia
Supraventricular extrasystoles
Supraventricular tachyarrhythmia
Anomalous atrioventricular excitation
Cardiac fibrillation
Cardiac flutter
Extrasystoles
Tachyarrhythmia
Cardiac fibrillation
Parasystole
Rhythm idioventricular
Ventricular arrhythmia
Ventricular extrasystoles
Ventricular fibrillation
Ventricular flutter
Ventricular parasystole
Ventricular pre-excitation
Andersen-Tawil syndrome
Arrhythmia neonatal
Arrhythmogenic right ventricular dysplasia
Atrioventricular node dispersion
Brugada syndrome
Foetal arrhythmia
Foetal heart rate disorder
Foetal tachyarrhythmia
Heart block congenital
Junctional ectopic tachycardia
Long QT syndrome congenital
Lown-Ganong-Levine syndrome
Neonatal bradyarrhythmia
Neonatal tachyarrhythmia
Wolff-Parkinson-White syndrome congenital
Baseline foetal heart rate variability disorder
Cardiac arrest neonatal
Cardio-respiratory arrest neonatal
Foetal cardiac arrest
Foetal heart rate acceleration abnormality
Foetal heart rate deceleration abnormality

Renal Impairment (Preferred Terms)
Atypical haemolytic uraemic syndrome
Anuria
Renal failure
Cardiorenal syndrome

Chronic kidney disease
End stage renal disease
Renal impairment
Crush syndrome
Diabetic end stage renal disease
Hepatorenal failure
Foetal renal impairment
Prerenal failure
Haemolytic uraemic syndrome
Hepatorenal syndrome
Nail-patella syndrome
Renal injury
Neonatal anuria
Pancreatorenal syndrome
Postoperative renal failure
Postrenal failure
Renal failure neonatal
Renal impairment neonatal
Scleroderma renal crisis
Subacute kidney injury

**X-ray or Radiologic Pneumonia (Preferred Terms)**
Eosinophilic pneumonia
Eosinophilic pneumonia acute
Eosinophilic pneumonia chronic
Pneumonitis
Pneumonitis chemical
Idiopathic interstitial pneumonia
Pneumonia
Atypical pneumonia
COVID-19 pneumonia
Embolic pneumonia
Enterobacter pneumonia
Haemorrhagic pneumonia
Miliary pneumonia
Paracancerous pneumonia
Pneumocystis jirovecii pneumonia
Pneumonia
Pneumonia acinetobacter
Pneumonia adenoviral
Pneumonia anthrax
Pneumonia bacterial
Pneumonia blastomyces
Pneumonia bordetella
Pneumonia chlamydial
Pneumonia cryptococcal
Pneumonia cytomegaloviral
Pneumonia escherichia
Pneumonia fungal
Pneumonia haemophilus
Pneumonia helminthic
Pneumonia herpes viral
Pneumonia influenzal
Pneumonia klebsiella
Pneumonia legionella
Pneumonia measles
Pneumonia moraxella
Pneumonia mycoplasmal
Pneumonia necrotising

Pneumonia parainfluenzae viral
Pneumonia pneumococcal
Pneumonia proteus
Pneumonia pseudomonal
Pneumonia respiratory syncytial viral
Pneumonia salmonella
Pneumonia serratia
Pneumonia staphylococcal
Pneumonia streptococcal
Pneumonia toxoplasmal
Pneumonia tularaemia
Pneumonia viral
Pneumonic plague
Post procedural pneumonia
Pulmonary mucormycosis
Pulmonary nocardiosis
Pulmonary paracoccidioidomycosis
Pulmonary sepsis
Pulmonary sporotrichosis
Pulmonary syphilis
Pulmonary trichosporonosis
Pulmonary tuberculosis
Pneumococcal infection
Pneumovirus test positive
Pulmonary tuberculoma

**Respiratory Arrest or Failure (Preferred Terms)**
Acute respiratory distress syndrome
Acute respiratory failure
Agonal respiration
Apnoea
Apnoeic attack
Bradypnoea
Cardio-respiratory distress
Central-alveolar hypoventilation
Chronic respiratory failure
Hypopnoea
Hypoventilation
Hypoventilation neonatal
Infantile apnoea
Lung hypoinflation
Neonatal respiratory arrest
Neonatal respiratory depression
Neonatal respiratory distress
Neonatal respiratory distress syndrome
Neonatal respiratory failure
Postoperative respiratory distress
Postoperative respiratory failure
Respiratory arrest
Respiratory depression
Respiratory depth decreased
Respiratory distress
Respiratory failure
Respiratory paralysis
Adaptive servo-ventilation
Alveolar oxygen partial pressure abnormal
Alveolar oxygen partial pressure decreased
Alveolar-arterial oxygen gradient increased

Respiratory Arrest or Failure (Preferred Terms)
Anoxia
Asphyxia
Automatic positive airway pressure
Bilevel positive airway pressure
Blood gases abnormal
Capnogram abnormal
Carbon dioxide abnormal
Carbon dioxide increased
Cardiopulmonary failure
Cardio-respiratory arrest
Cardio-respiratory arrest neonatal
Cheyne-Stokes respiration
Chronic respiratory disease
Continuous positive airway pressure
Cyanosis central
Dependence on oxygen therapy
Dependence on respirator
Dyspnoea at rest
Endotracheal intubation
End-tidal CO2 abnormal
End-tidal CO2 decreased
Hyperbaric oxygen therapy
Hypercapnia
Hypercapnic coma
Hypoxia
Intermittent positive pressure breathing
Irregular breathing
Life support
Lung assist device therapy
Mechanical ventilation
Mechanical ventilation complication
Neonatal anoxia
Neonatal asphyxia
Neonatal hypoxia
Neonatal respiratory acidosis
Neonatal respiratory distress syndrome prophylaxis
Neonatal tachypnoea
Oxygen saturation abnormal
Oxygen saturation decreased
Oxygen therapy
PaO2/FiO2 ratio decreased
PCO2 abnormal
PCO2 decreased
PCO2 increased
PO2 abnormal
PO2 decreased
Positive end-expiratory pressure
Positive expiratory pressure therapy
Respiration abnormal
Respiratory acidosis
Respiratory disorder
Respiratory disorder neonatal
Respiratory fatigue
Respiratory gas exchange disorder
Use of accessory respiratory muscles
Venous oxygen partial pressure abnormal

Respiratory Arrest or Failure (Preferred Terms)
Venous oxygen partial pressure decreased
Venous oxygen saturation abnormal
Venous oxygen saturation decreased
Ventilation perfusion mismatch
Ventilation/perfusion scan abnormal
Wean from ventilator
Weaning failure
Acute respiratory failure
Agonal respiration
Apnoea
Apnoeic attack
Bradypnoea
Central-alveolar hypoventilation
Hypopnoea
Hypoventilation
Hypoventilation neonatal
Infantile apnoea
Lung hypoinflation
Neonatal respiratory arrest
Neonatal respiratory depression
Neonatal respiratory failure
Postoperative respiratory failure
Respiratory arrest
Respiratory depression
Respiratory depth decreased
Respiratory failure
Respiratory paralysis
Adaptive servo-ventilation
Alveolar oxygen partial pressure abnormal
Alveolar oxygen partial pressure decreased
Anoxia
Apnoea test abnormal
Asphyxia
Automatic positive airway pressure
Bilevel positive airway pressure
Blood gases abnormal
Capnogram abnormal
Carbon dioxide abnormal
Carbon dioxide increased
Cardiopulmonary failure
Cardio-respiratory arrest
Cardio-respiratory arrest neonatal
Cardio-respiratory distress
Cheyne-Stokes respiration
Continuous positive airway pressure
Cyanosis
Cyanosis central
Dependence on oxygen therapy
Dependence on respirator
Dyspnoea
End-tidal CO2 abnormal
End-tidal CO2 decreased
Hyperbaric oxygen therapy
Hypercapnia
Hypercapnic coma
Hypoxia

**Respiratory Arrest or Failure (Preferred Terms)**
Intermittent positive pressure breathing
Irregular breathing
Life support
Lung assist device therapy
Mechanical ventilation
Mechanical ventilation complication
Neonatal anoxia
Neonatal asphyxia
Neonatal dyspnoea
Neonatal hypoxia
Neonatal respiratory acidosis
Neonatal respiratory distress syndrome prophylaxis
Oxygen saturation abnormal
Oxygen saturation decreased
Oxygen therapy
PaO2/FiO2 ratio decreased
PCO2 abnormal
PCO2 increased
PO2 abnormal
PO2 decreased
Positive end-expiratory pressure
Respiration abnormal
Respiratory acidosis
Respiratory disorder
Respiratory disorder neonatal
Respiratory distress
Respiratory gas exchange disorder
Venous oxygen partial pressure abnormal
Venous oxygen partial pressure decreased
Venous oxygen saturation abnormal
Venous oxygen saturation decreased
Ventilation perfusion mismatch
Ventilation/perfusion scan abnormal
Wean from ventilator
Weaning failure

**Pulmonary Embolism (Preferred Terms)**
Pulmonary embolism
Pulmonary infarction
Post procedural pulmonary embolism
Pulmonary artery thrombosis
Pulmonary microemboli
Pulmonary oil microembolism
Pulmonary thrombosis
Pulmonary tumour thrombotic microangiopathy
Pulmonary venous thrombosis
Septic pulmonary embolism

**Pleural Effusion (Preferred Terms)**
Pleural effusion
Haemothorax
Eosinophilic pleural effusion
Traumatic haemothorax
Hepatic hydrothorax
Hydrothorax

Procedural pneumothorax
Neonatal pneumothorax
Paraneoplastic pleural effusion
Pleuroperitoneal communication

**Atelectasis (Preferred Terms)**
Atelectasis
Atelectasis neonatal

Acute Coronary Events (Preferred Terms)
Acute myocardial infarction
Acute coronary syndrome
Myocardial ischaemia
Myocardial infarction
Coronary artery dissection
Coronary artery aneurysm
Angina pectoris
Angina unstable
Prinzmetal angina
Anginal equivalent
Congenital coronary artery malformation
Coronary artery insufficiency
Arteriosclerosis coronary artery
Arteriospasm coronary
Arteritis coronary
Coronary artery disease
Cardiac perfusion defect
Microvascular coronary artery disease
Chronic coronary syndrome
Coronary artery stenosis
Haemorrhage coronary artery
Coronary artery compression
Coronary artery dilatation
Coronary artery embolism
Coronary artery occlusion
Coronary artery perforation
Coronary artery reocclusion
Coronary artery restenosis
Coronary artery thrombosis
Coronary bypass stenosis
Coronary bypass thrombosis
Coronary no-reflow phenomenon
Coronary ostial stenosis
Coronary sinus dilatation
Coronary steal syndrome
Coronary vascular graft occlusion
Coronary vascular graft stenosis
Diabetic coronary microangiopathy
Subendocardial ischaemia
Papillary muscle infarction
Kounis syndrome
Myocardial reperfusion injury
Myocardial stunning
Periprocedural myocardial infarction
Post procedural myocardial infarction
Postinfarction angina
Silent myocardial infarction
Subclavian coronary steal syndrome
Wellens' syndrome

Altered Mental Status (SOC)
Psychiatric disorders

Acute Cerebrovascular Events (Preferred Terms)
Cerebrovascular accident
Subdural haematoma

Acute Cerebrovascular Events (Preferred Terms)
Superior sagittal sinus thrombosis
Cerebrovascular disorder
Spinal artery embolism
Amaurosis fugax
Amyloid related imaging abnormalities
Amyloid related imaging abnormality-oedema/effusion
Amyloid related imaging abnormality-microhaemorrhages and haemosiderin deposits
Intracranial aneurysm
Brain hypoxia
Cerebral artery embolism
Cerebral artery occlusion
Cerebral artery perforation
Cerebral artery stenosis
Cerebral artery thrombosis
Aseptic cavernous sinus thrombosis
Cerebral infarction
Haemorrhagic transformation stroke
Subarachnoid haemorrhage
Cerebral arteriosclerosis
Basal ganglia haemorrhage
Basal ganglia haematoma
Basal ganglia infarction
Basal ganglia stroke
Basilar artery aneurysm
Basilar artery occlusion
Basilar artery perforation
Basilar artery stenosis
Vertebrobasilar insufficiency
Basilar artery thrombosis
Benedikt's syndrome
Reversible cerebral vasoconstriction syndrome
Vascular encephalopathy
Haemorrhage intracranial
Blood brain barrier defect
Cerebral congestion
Brain stem haemorrhage
Brain stem embolism
Brain stem haematoma
Brain stem infarction
Brain stem ischaemia
Brain stem microhaemorrhage
Brain stem stroke
Brain stem thrombosis
Capsular warning syndrome
Embolic stroke
Carotid aneurysm rupture
Carotid arterial embolus
Carotid arteriosclerosis
Carotid artery aneurysm
Carotid artery disease
Carotid artery dissection
Carotid artery dolichoectasia
Carotid artery insufficiency
Carotid artery occlusion
Carotid artery perforation
Carotid artery restenosis

**Acute Cerebrovascular Events (Preferred Terms)**
Carotid artery stenosis
Carotid artery thrombosis
Cavernous sinus thrombosis
Central nervous system haemorrhage
Central nervous system vasculitis
Cerebellar artery occlusion
Cerebellar artery thrombosis
Cerebellar atherosclerosis
Cerebellar haemorrhage
Cerebellar embolism
Cerebellar haematoma
Cerebellar infarction
Cerebellar ischaemia
Cerebellar microhaemorrhage
Cerebellar stroke
Cerebral amyloid angiopathy
Cerebral aneurysm perforation
Cerebral aneurysm ruptured syphilitic
Cerebral vasoconstriction
Cerebral arteriovenous malformation haemorrhagic
Cerebral arteritis
Intraoperative cerebral artery occlusion
Cerebral artery restenosis
Cerebral haemorrhage
Cerebral capillary telangiectasia
Cerebral circulatory failure
Cerebral gas embolism
Cerebral haematoma
Cerebral haemorrhage foetal
Cerebral haemorrhage neonatal
Traumatic intracranial haemorrhage
Cerebral haemosiderin deposition
Cerebral hyperperfusion syndrome
Cerebral hypoperfusion
Cerebral infarction foetal
Cerebral ischaemia
Cerebral microangiopathy
Cerebral microhaemorrhage
Cerebral microembolism
Cerebral microinfarction
Cerebral reperfusion injury
Cerebral septic infarct
Cerebral small vessel ischaemic disease
Cerebral thrombosis
Cerebral vascular occlusion
Cerebral vasodilatation
Cerebral venous sinus thrombosis
Cerebral venous thrombosis
Cerebrovascular arteriovenous malformation
Foetal cerebrovascular disorder
Cerebrovascular insufficiency
Cerebrovascular pseudoaneurysm
Cerebrovascular stenosis
Charcot-Bouchard microaneurysms
Chronic cerebrospinal venous insufficiency

Acute Cerebrovascular Events (Preferred Terms)
Claude's syndrome
Spinal vessel congenital anomaly
Giant cell arteritis
Delayed ischaemic neurological deficit
Dural arteriovenous fistula
Embolic cerebellar infarction
Embolic cerebral infarction
Epidural haemorrhage
Extradural haematoma
Jugular vein haemorrhage
Extra-axial haemorrhage
Extraischaemic cerebral haematoma
Haemorrhagic stroke
Intraventricular haemorrhage neonatal
Spinal cord haemorrhage
Subdural haemorrhage
Haemorrhagic cerebral infarction
Hypertensive cerebrovascular disease
Inner ear infarction
Transient ischaemic attack
Internal capsule infarction
Internal carotid artery deformity
Intracranial artery dissection
Intracranial haematoma
Ruptured cerebral aneurysm
Intracranial tumour haemorrhage
Intraventricular haemorrhage
Ischaemic cerebral infarction
Ischaemic stroke
Lacunar infarction
Lacunar stroke
Lateral medullary syndrome
Lenticulostriatal vasculopathy
Meningorrhagia
Migrainous infarction
Moyamoya disease
Pituitary infarction
Subarachnoid haemorrhage neonatal
Subdural haemorrhage neonatal
Precerebral artery occlusion
Vertebral artery occlusion
Perinatal stroke
Periventricular haemorrhage neonatal
PHACES syndrome
Pituitary apoplexy
Pituitary haemorrhage
Post cardiac arrest syndrome
Post procedural stroke
Post stroke depression
Precerebral arteriosclerosis
Precerebral artery embolism
Precerebral artery thrombosis
Pseudostroke
Putamen haemorrhage
Susac's syndrome
Reversible ischaemic neurological deficit

Acute Cerebrovascular Events (Preferred Terms)
Sneddon's syndrome
Spinal artery thrombosis
Spinal cord haematoma
Spinal cord infarction
Spinal epidural haemorrhage
Spinal epidural haematoma
Spinal stroke
Spinal subarachnoid haemorrhage
Spinal subdural haemorrhage
Spinal subdural haematoma
Spinal vascular disorder
Spontaneous internal carotid artery recanalisation
Stroke in evolution
Subarachnoid haematoma
Subclavian steal syndrome
Superficial siderosis of central nervous system
Weber's syndrome
Thalamic infarction
Thalamus haemorrhage
Thrombotic cerebral infarction
Thrombotic stroke
Transient aphasia
Transverse sinus stenosis
Transverse sinus thrombosis
Traumatic intracranial haematoma
Vascular cognitive impairment
Vein of Galen aneurysmal malformation
Vertebral artery aneurysm
Vertebral artery arteriosclerosis
Vertebral artery dissection
Vertebral artery perforation
Vertebral artery stenosis
Vertebral artery thrombosis
Vertebrobasilar dolichoectasia
Vertebrobasilar stroke
Wyburn Mason's syndrome

Seizure (Preferred Terms)
Acquired epileptic aphasia
Atonic seizures
Atypical benign partial epilepsy
Automatism epileptic
Autonomic seizure
Baltic myoclonic epilepsy
Benign rolandic epilepsy
Change in seizure presentation
Clonic convulsion
Congenital bilateral perisylvian syndrome
Convulsion in childhood
Convulsions local
Convulsive threshold lowered
Dreamy state
Early infantile epileptic encephalopathy with burst-suppression
Epilepsy
Epilepsy surgery
Epilepsy with myoclonic-atonic seizures

Seizure (Preferred Terms)
Epileptic aura
Epileptic psychosis
Faciobrachial dystonic seizure
Febrile convulsion
Febrile infection-related epilepsy syndrome
Focal dyscognitive seizures
Frontal lobe epilepsy
Gelastic seizure
Generalised onset non-motor seizure
Generalised tonic-clonic seizure
Hemiconvulsion-hemiplegia-epilepsy syndrome
Hemimegalencephaly
Hyperglycaemic seizure
Hypocalcaemic seizure
Hypoglycaemic seizure
Hyponatraemic seizure
Idiopathic generalised epilepsy
Infantile spasms
Jeavons syndrome
Juvenile absence epilepsy
Juvenile myoclonic epilepsy
Lafora's myoclonic epilepsy
Lennox-Gastaut syndrome
Migraine-triggered seizure
Myoclonic epilepsy
Myoclonic epilepsy and ragged-red fibres
Neonatal epileptic seizure
Neonatal seizure
Parietal lobe epilepsy
Partial seizures
Partial seizures with secondary generalisation
Petit mal epilepsy
Polymicrogyria
Post stroke epilepsy
Post stroke seizure
Postictal headache
Postictal paralysis
Postictal psychosis
Postictal state
Seizure
Seizure anoxic
Seizure cluster
Seizure like phenomena
Severe myoclonic epilepsy of infancy
Simple partial seizures
Status epilepticus
Sudden unexplained death in epilepsy
Temporal lobe epilepsy
Tonic clonic movements
Tonic convulsion
Tonic posturing
Topectomy
Transient epileptic amnesia
Aura
Drop attacks
Narcolepsy
Preictal state
Seizure prophylaxis
Tongue biting
Altered state of consciousness

**Seizure (Preferred Terms)**
Depressed level of consciousness
Loss of consciousness
Clonus
Hypotonia

**Syncope (Preferred Terms)**
Syncope

**Systemic Inflammatory Response (Preferred Terms)**
Systemic inflammatory response syndrome

**New Neurological Deficit (SOC)**
Nervous system disorders

**Asthenia (Preferred Terms)**
Asthenia
Cachexia
Cancer fatigue
Fatigue
Chronic fatigue syndrome
Decreased activity
Malaise
Lethargy
Listless
Sluggishness

**Dehydration (Preferred Terms)**
Dehydration
Dry eye
Dry mouth
Dry skin
Dry throat
Fluid balance negative
Lip dry
Mucosal dryness
Nasal dryness
Tongue dry

**Metabolic Disturbances (SOC)**
Metabolism and nutrition disorders

7.8.1. Update MEDRA 24.1 to MEDRA 25.1

Due to updates to the MEDRA version, the following PTs are added

**Cardiac Arrhythmia (Preferred Term)**
Atrial standstill

**X-ray or Radiologic Pneumonia (Preferred Terms)**
Pulmonary blastomycosis

**Acute Cerebrovasular Events (Preferred Term)**
Vertebrobasilar artery dissection

7.9. Appendix 9 Area Under the Curve Calculation

In the calculation of the AUC, not only the date, but also the timing (the real hours, minutes and seconds as captured in the database should be used, but the AUC result should be reported in hours), of the assessment, is taken into account.

$$\boldsymbol{AUC\ of\ total\ score = \sum_{i=2}^{T} \frac{[TS_{t_i} + TS_{t_{(i-1)}}]}{2} [t_i - t_{(i-1)}]} \qquad \textbf{(1)}$$

where

$t_i = (actual)\ timepoint\ i$

$t_{i-1} = (actual)\ timepoint\ (i-1)$

$T = last\ timepoint$

$t_1 = first\ timepoint$

$TS_{t_i} = Total\ score\ at\ \ (actual)\ timepoint\ i$

$TS_{t_{(i-1)}} = Total\ score\ at\ \ (actual)\ timepoint\ (i-1)$

7.10. Appendix 10 Therapeutic Interventions of Interest

Corticosteroid's prescription

ATC codes	ATC Text
A01AC	Corticosteroids for local oral treatment
A07EA	Corticosteroids acting locally
A11ED	Vitamin B-complex with anabolic steroids
A14A	Anabolic steroids
C05AA	Corticosteroids
D01AC	imidazoles/triazoles in combination with corticosteroids (subset of terms with this ATC code)
D07	CORTICOSTEROIDS, DERMATOLOGICAL PREPARATIONS
D07A	CORTICOSTEROIDS, PLAIN
D07AA	Corticosteroids, weak (group I)
D07AB	Corticosteroids, moderately potent (group II)
D07AC	Corticosteroids, potent (group III)
D07AD	Corticosteroids, very potent (group IV)
D07B	CORTICOSTEROIDS, COMBINATIONS WITH ANTISEPTICS
D07BA	Corticosteroids, weak, combinations with antiseptics
D07BB	Corticosteroids, moderately potent, combinations with antiseptics
D07BC	Corticosteroids, potent, combinations with antiseptics
D07BD	Corticosteroids, very potent, combinations with antiseptics
D07C	CORTICOSTEROIDS, COMBINATIONS WITH ANTIBIOTICS
D07CA	Corticosteroids, weak, combinations with antibiotics
D07CB	Corticosteroids, moderately potent, combinations with antibiotics
D07CC	Corticosteroids, potent, combinations with antibiotics
D07CD	Corticosteroids, very potent, combinations with antibiotics
D07X	CORTICOSTEROIDS, OTHER COMBINATIONS
D07XA	Corticosteroids, weak, other combinations
D07XB	Corticosteroids, moderately potent, other combinations
D07XC	Corticosteroids, potent, other combinations
D07XD	Corticosteroids, very potent, other combinations
D10AA	Corticosteroids, combinations for treatment of acne
G01B	ANTIINFECTIVES/ANTISEPTICS IN COMBINATION WITH CORTICOSTEROIDS
G01BA	Antibiotics and corticosteroids
G01BC	Quinoline derivatives and corticosteroids
G01BD	Antiseptics and corticosteroids
G01BE	Sulfonamides and corticosteroids
G01BF	Imidazole derivatives and corticosteroids
H02	CORTICOSTEROIDS FOR SYSTEMIC USE
H02A	CORTICOSTEROIDS FOR SYSTEMIC USE, PLAIN
H02B	CORTICOSTEROIDS FOR SYSTEMIC USE, COMBINATIONS
H02BX	Corticosteroids for systemic use, combinations
M01BA	Antiinflammatory/antirheumatic agents in combination with corticosteroids
N02CB	Corticosteroid derivatives
R01AD	Corticosteroids
R03AK	Adrenergics in combination with corticosteroids or other drugs, excl. anticholinergics
R03AL	Adrenergics in combination with anticholinergics incl. triple combinations with corticosteroids
R03BA	Corticosteroids for inhalation
S01BA	Corticosteroids, plain
S01BB	Corticosteroids and mydriatics in combination
S01CA	Corticosteroids and antiinfectives in combination
S01CB	Corticosteroids/antiinfectives/mydriatics in combination
S02B	CORTICOSTEROIDS
S02BA	Corticosteroids
S02C	CORTICOSTEROIDS AND ANTIINFECTIVES IN COMBINATION
S02CA	Corticosteroids and antiinfectives in combination
S03B	CORTICOSTEROIDS
S03BA	Corticosteroids

ATC codes	ATC Text
S03C	CORTICOSTEROIDS AND ANTIINFECTIVES IN COMBINATION
S03CA	Corticosteroids and antiinfectives in combination
H02AB	Glucocorticoids

Bronchodilator/nebulizer treatment

ATC Code	ATC Text
R03	Drugs for Obstructive Airway Diseases
R03A	Adrenergics Inhalents
R03AA	Alpha-and beta-adrenoreceptor agonists
R03AB	Non Selective beta-adrenoreceptor agonists
R03AC	Selective beta-2-adrenoreceptor agonists
R03AH	Combinations of adrenergics
R03AK	Adrenergics and other drugs for obstructive airway diseases
RO3AL	Adrenergics in combination with corticosteroids or other drugs excl. anticholinergics
R03B	Other Drugs for Obstructive Airways Diseases, Inhalents
R03BA	Glucocorticoids
R03BB	Anticholinergics
R03BC	Antiallergic agents excl. corticosteroids
R03BX	Other Drugs for Obstructive Airways Diseases, Inhalents
R03C	Adrenergics for systemic use
R03CA	Alpha-and beta-adrenoreceptor agonists
R03CB	Non Selective beta-adrenoreceptor agonists
R03CC	Selective beta-2-adrenoreceptor agonists
R03CK	Adrenergics and other drugs for obstructive airway diseases
R03D	Other Systemic Drugs for Obstructive Airways Disease
R03DA	Xanthines
R03DB	Xanthines and Adrenergics
R03DC	Leukotriene receptor agonists
R03DX	Other Systemic Drugs for Obstructive Airways Disease
C01CX	Other cardiac stimulants
R01AX	Other nasal preparations

Antibiotic prescription

ATC Code	ATC Text
A01AB	Antiinfectives and antiseptics for local oral treatment
A02BD	Combinations for eradication of Helicobacter pylori
A07A	Intestinal antiinfectives
C05AB	Antibiotics
D01AA	Antibiotics
D06	Antibiotics and chemotherapeutics for dermatological use
D07C	Corticosteroids, combinations with antibiotics
D09AA	Ointment dressings with antiinfectives
D10AF	Antiinfectives for treatment of acne
G01	Gynecological antiinfectives and antiseptics
J01	Antibacterials for systemic use
J01A	Tetracyclines
J01AA	Tetracyclines
J01B	Amphenicols
J01BA	Amphenicols
J01C	Beta-Lactam Antibacterials, penicillins
J01CA	Penicillins with extended spectrum
J01CE	Beta-lactamase sensitive penicillins
J01CF	Beta-lactamase resistant penicillins
J01CG	Beta-lactamase inhibitors

ATC Code	ATC Text
J01CR	Combinations of penicillins, incl. beta-lactamase inhibitors
J01D	Other Beta-Lactam Antibacterials
J01DB	First generation cephalosporins
J01DC	Second generation cephalosporins
J01DD	Third generation cephalosporins
J01DE	Fourth generation cephalosporins
J01DF	Monobactams
J01DH	Carbapenems
J01DI	Other cephalosporins and penems
J01E	Sulfonamides and Trimethroprim
J01EA	Trimethoprim and derivatives
J01EB	Short acting sulfonamides
J01EC	Intermediate-acting sulfonamides
J01ED	Long-acting sulfonamides
J01EE	Combinations of sulfonamides and trimethoprim, incl derivatives
J01F	Macrolides, Lincosamides and Streptogramins
J01FA	Macrolides
J01FG	Streptogramins
J01G	Aminoglycoside Antibacterials
J01GA	Streptomycins
J01GB	Other aminoglycosides
J01M	Qunilone Antibacterials
J01MA	Fluoroquinolones
J01MB	Other quinolones
J01R	Combinations of antibacterials
J01RA	Combinations of antibacterials
J01X	Other Antibacterials
J01XA	Glycopeptide antibacterials
J01XB	Polymyxins
J01XC	Steroid antibacterials
J01XD	Imidazole derivatives
J01XE	Nitrofuran derivatives
J01XX	Other Antibacterials
J0IFF	Lincosamides
J02AA	Antibiotics
J04	Antimycobacterials
J07A	Bacterial vaccines
J07C	Bacterial and viral vaccines, combined
L01D	Cytotoxic antibiotics and related substances
R02AB	Antibiotics
S01A	Antiinfectives
S01AA	Antibiotics
S01AB	Sulfonamides
S02A	Antiinfectives
S02AA	Antiinfectives
S02C	Corticosteroids and antiinfectives in combination
S02CA	Corticosteroids and antiinfectives in combination
S03	Antiinfectives
S03AA	Antiinfectives
S03C	Corticosteroids and antiinfectives in combination
S03CA	Corticosteroids and antiinfectives in combination

Antiviral prescription

ATC Codes	ATC Text
D06BB	Antivirals
J05	Antivirals for systemic use

ATC Codes	ATC Text
J05A	Direct acting anti-virals
J05AA	Thiosemicarbazones
J05AB	Nucleosides and nucoeotides exc. Reverse transcriptase inhibitors
J05AC	Cyclic amines
J05AD	Phosphonic acid derivatives
J05AE	Protease inhibitors
J05AF	Nucleosides and nucoeotides reverse transcriptase inhibitors
J05AG	Non-nucleoside reverse transcriptase inhibitors
J05AH	Neuraminidase inhibitors
J05AP	Antivirals for treatment of HCV infections
J05AR	Antivirals for treatment of HIV infections
J05AX	Other antivirals
S01AD	Antivirals

7.11. Appendix 11 Binomial Exact Test

$$\textbf{Vaccine Efficacy} = 1 - \text{Relative Risk} = 1 - \frac{\#\text{ of Events}_{\text{VX}} / \#\text{ of Subjects}_{\text{VX}}}{\#\text{ of Events}_{\text{P}} / \#\text{ of Subjects}_{\text{P}}}$$

$$= 1 - \frac{1}{r} * \frac{\#\text{ of Events}_{\text{VX}}}{\#\text{ of Events}_{\text{p}}} = 1 - \frac{1}{r} * \frac{\#\text{ of Events}_{\text{VX}} / \#\text{ of Events}_{\text{T}}}{(\#\text{ of Events}_{\text{T}} - \#\text{ of Events}_{\text{VX}}) / \#\text{ of Events}_{\text{T}}}$$

$$= 1 - \frac{1}{r} * \frac{\#\text{ of Events}_{\text{VX}} / \#\text{ of Events}_{\text{T}}}{1 - \#\text{ of Events}_{\text{VX}} / \#\text{ of Events}_{\text{T}}} = 1 - \frac{p}{r * (1 - p)}$$

Where,

$$\boldsymbol{r} = \frac{\#\text{ of Subjects}_{\text{VX}}}{\#\text{ of Subjects}_{\text{p}}}$$

$$\boldsymbol{p} = \#\text{ of Events}_{\text{VX}} / \#\text{ of Events}_{\text{T}}$$

$\#\ \boldsymbol{of\ Events_{VX}}$ = number of events in the active group
$\#\ \boldsymbol{of\ Events_{P}}$ = number of events in the Placebo group
$\#\ \boldsymbol{of\ Events_{T}}$ = number of events, regardless the group

Therefore, there is a monotonic link between VE, the vaccine efficacy, and p, the proportion of subjects in the vaccine group among the total cases in the two groups.

Note that, conditional on the total number of events, $\#\ \boldsymbol{of\ Events_{VX}}$ is binomially distributed ($\#\ \boldsymbol{of\ Events_{VX}, n}$) with n the expected proportion of events in the vaccine group under the true vaccine efficacy.

The CI for vaccine efficacy can then be derived from the exact CI from p (Dragalin, Fedorov and Cheuvart, 2002)[0].

7.12. Appendix 12 EQ-5D Valuation index

The validation index will be defined based on the following equation:

$$EQ - 5DValidation\ index =$$

$$= 0 * MO1 - 0.096 * MO2 - 0.122 * MO3 - 0.237 * M04 - 0.322 * MO5$$

$$+ 0 * SC1 - 0.089 * SC2 - 0.107 * SC3 - 0.220 * SC4 - 0.261 * SC5$$

$$+ 0 * UA1 - 0.068 * UA2 - 0.101 * UA3 - 0.255 * UA4 - 0.255 * UA5$$

$$+ 0 * PD1 - 0.060 * PD2 - 0.098 * PD3 - 0.318 * PD4 - 0.414 * PD5$$

$$+ 0 * AD1 - 0.057 * AD2 - 0.123 * AD3 - 0.299 * AD4 - 0.321 * AD5$$

$$Where\ M0x, SCx, UAx, PDx, ADx = \begin{cases} 0\ if\ participant\ did\ not\ selected \\ 1\ if\ participant\ selected\ it \end{cases}, with\ x=1,2,3,4,5$$

For example, if a participant's health state is 12543, which indicates 'no problems walking, slight problems washing or dressing myself, unable to do my usual activities, severe pain or discomfort, moderately anxious or depressed, the participants index score will be:

$$EQ - 5DValidation\ index =$$

$$= 0 * 1 - 0.096 * 0 - 0.122 * 0 - 0.237 * 0 - 0.322 * 0$$

$$+ 0 * 0 - 0.089 * 1 - 0.107 * 0 - 0.220 * 0 - 0.261 * 0$$

$$+ 0 * 0 - 0.068 * 0 - 0.101 * 0 - 0.255 * 0 - 0.255 * 1$$

$$+ 0 * 0 - 0.060 * 0 - 0.098 * 0 - 0.318 * 1 - 0.414 * 0$$

$$+ 0 * 0 - 0.057 * 0 - 0.123 * 1 - 0.299 * 0 - 0.321 * 0 =$$

$$-0.785$$

If a participant has no problems with a health state equal to 11111 then the index score would be 0.

The above equation is based on Table 2 Model 1 from 'United States Valuation of EQ-5D-5L Health States Using an International'[0] paper.

7.13. Appendix 13 Chronic Kidney Disease [CKD] + Diabetes Terms

**Chronic Kidney Disease**
Artificial kidney device user
Chronic kidney disease
Chronic kidney disease-mineral and bone disorder
Diabetic end stage renal disease
Dialysis
Dialysis device insertion
End stage renal disease
Glomerulonephritis chronic
Haemodialysis
Haemofiltration
Hepatorenal failure
Hyperparathyroidism secondary
Kidney fibrosis
Metabolic nephropathy
Nephrogenic systemic fibrosis
Nephrosclerosis
Oedema due to renal disease
Peritoneal dialysis
Renal and liver transplant
Renal and pancreas transplant
Renal failure
Renal replacement therapy
Renal rickets
Renal transplant
Acquired cystic kidney disease
Acquired hypocalciuric hypercalcaemia
Acquired perforating dermatosis
Acute phosphate nephropathy
Albuminuria
Autoimmune nephritis
C1q nephropathy
C3 glomerulopathy
Chronic allograft nephropathy
Diabetic nephropathy
Dialysis amyloidosis
Dialysis disequilibrium syndrome
Dialysis hypotension
Dialysis induced hypertension
Dialysis membrane reaction
Dialysis related complication
Diffuse mesangial sclerosis
Extensive interdialytic weight gain
Fibrillary glomerulonephritis
Focal segmental glomerulosclerosis
Glomerular filtration rate abnormal
Glomerular filtration rate decreased
Glomerulonephritis
Glomerulonephritis membranoproliferative
Glomerulonephritis membranous
Glomerulonephritis minimal lesion
Glomerulonephritis proliferative
Glomerulonephritis rapidly progressive
Glomerulonephropathy
Glomerulosclerosis
Goodpasture's syndrome
Haemodialysis complication
Haemolytic uraemic syndrome
Hepatitis virus-associated nephropathy

**Chronic Kidney Disease**
HIV associated nephropathy
Hypercalcaemic nephropathy
Hypertensive nephropathy
IgA nephropathy
IgM nephropathy
Immune-mediated nephritis
Immune-mediated renal disorder
Immunotactoid glomerulonephritis
Inadequate haemodialysis
Inadequate peritoneal dialysis
Intercapillary glomerulosclerosis
Intradialytic parenteral nutrition
Inulin renal clearance decreased
Ischaemic nephropathy
Kidney small
Lupus nephritis
Membranous-like glomerulopathy with masked IgG-kappa deposits
Mesangioproliferative glomerulonephritis
Metabolic acidosis
Microalbuminuria
Nephritic syndrome
Nephropathy
Nephropathy toxic
Nephrotic syndrome
Neutrophil gelatinase-associated lipocalin increased
Obstructive nephropathy
Paraneoplastic glomerulonephritis
Paraneoplastic nephrotic syndrome
Peritoneal dialysate leakage
Peritoneal dialysis complication
Pigment nephropathy
Polyomavirus-associated nephropathy
Potassium wasting nephropathy
Protein urine present
Proteinuria
Red blood cells urine positive
Reflux nephropathy
Renal amyloidosis
Renal atrophy
Renal papillary necrosis
Renal tubular atrophy
Sickle cell nephropathy
Tubulointerstitial nephritis
Ultrafiltration failure
Urate nephropathy
Urea renal clearance decreased
Urine albumin/creatinine ratio abnormal
Urine albumin/creatinine ratio increased
Urine protein/creatinine ratio abnormal
Urine protein/creatinine ratio increased

**Diabetes**
Acquired lipoatrophic diabetes
Diabetes complicating pregnancy
Diabetes mellitus
Diabetes mellitus inadequate control
Diabetes with hyperosmolarity
Diabetic arteritis
Diabetic coma
Diabetic coronary microangiopathy

**Diabetes**
Diabetic hepatopathy
Diabetic hyperglycaemic coma
Diabetic hyperosmolar coma
Diabetic ketoacidosis
Diabetic ketoacidotic hyperglycaemic coma
Diabetic ketosis
Diabetic metabolic decompensation
Diabetic wound
Euglycaemic diabetic ketoacidosis
Fructosamine increased
Fulminant type 1 diabetes mellitus
Gestational diabetes
Glycosuria
Glycosylated haemoglobin abnormal
Glycosylated haemoglobin increased
Hyperglycaemic hyperosmolar nonketotic syndrome
Hyperglycaemic seizure
Hyperglycaemic unconsciousness
Insulin resistance
Insulin resistant diabetes
Insulin-requiring type 2 diabetes mellitus
Ketosis-prone diabetes mellitus
Latent autoimmune diabetes in adults
Monogenic diabetes
Neonatal diabetes mellitus
New onset diabetes after transplantation
Pancreatogenous diabetes
Steroid diabetes
Type 1 diabetes mellitus
Type 2 diabetes mellitus
Type 3 diabetes mellitus
Anti-GAD antibody positive
Anti-IA2 antibody positive
Anti-insulin antibody increased
Anti-insulin antibody positive
Anti-insulin receptor antibody increased
Anti-insulin receptor antibody positive
Anti-islet cell antibody positive
Anti-zinc transporter 8 antibody positive
Blood insulin abnormal
Blood insulin decreased
Hypoinsulinaemia
Impaired insulin secretion
Increased insulin requirement
Insulin autoimmune syndrome
Insulin therapy
Insulin tolerance test abnormal
Metabolic syndrome
Wolfram syndrome

7.14. Appendix 14 List of preferred terms for VTE (broad)

Prefered Term	PTCODE	Level	Scope	Category
Aseptic cavernous sinus thrombosis	10084527	PT	Narrow	A
Axillary vein thrombosis	10003880	PT	Narrow	A
Brachiocephalic vein occlusion	10076837	PT	Narrow	A
Brachiocephalic vein thrombosis	10063363	PT	Narrow	A
Budd-Chiari syndrome	10006537	PT	Narrow	A
Catheterisation venous	10052698	PT	Narrow	A
Cavernous sinus thrombosis	10007830	PT	Narrow	A
Central venous catheterisation	10053377	PT	Narrow	A
Cerebral venous sinus thrombosis	10083037	PT	Narrow	A
Cerebral venous thrombosis	10008138	PT	Narrow	A
Compression garment application	10079209	PT	Narrow	A
Deep vein thrombosis	10051055	PT	Narrow	A
Deep vein thrombosis postoperative	10066881	PT	Narrow	A
Embolism venous	10014522	PT	Narrow	A
Hepatic vein embolism	10078810	PT	Narrow	A
Hepatic vein occlusion	10058991	PT	Narrow	A
Hepatic vein thrombosis	10019713	PT	Narrow	A
Homans' sign positive	10051031	PT	Narrow	A
Iliac vein occlusion	10058992	PT	Narrow	A
Inferior vena cava syndrome	10070911	PT	Narrow	A
Inferior vena caval occlusion	10058987	PT	Narrow	A
Jugular vein embolism	10081850	PT	Narrow	A
Jugular vein occlusion	10076835	PT	Narrow	A
Jugular vein thrombosis	10023237	PT	Narrow	A
Mahler sign	10075428	PT	Narrow	A
May-Thurner syndrome	10069727	PT	Narrow	A
Mesenteric vein thrombosis	10027402	PT	Narrow	A
Mesenteric venous occlusion	10027403	PT	Narrow	A
Obstetrical pulmonary embolism	10029925	PT	Narrow	A
Obstructive shock	10073708	PT	Narrow	A
Ophthalmic vein thrombosis	10074349	PT	Narrow	A
Ovarian vein thrombosis	10072059	PT	Narrow	A
Paget-Schroetter syndrome	10050216	PT	Narrow	A
Pelvic venous thrombosis	10034272	PT	Narrow	A
Penile vein thrombosis	10034324	PT	Narrow	A
Peripheral vein occlusion	10083103	PT	Narrow	A
Peripheral vein thrombus extension	10082853	PT	Narrow	A
Phlebectomy	10048874	PT	Narrow	A
Portal vein cavernous transformation	10073979	PT	Narrow	A
Portal vein embolism	10082030	PT	Narrow	A
Portal vein occlusion	10058989	PT	Narrow	A
Portal vein thrombosis	10036206	PT	Narrow	A

Portosplenomesenteric venous thrombosis	10077623 PT	Narrow	A
Post procedural pulmonary embolism	10063909 PT	Narrow	A
Post thrombotic syndrome	10048591 PT	Narrow	A
Postoperative thrombosis	10050902 PT	Narrow	A
Postpartum venous thrombosis	10036300 PT	Narrow	A
Pulmonary embolism	10037377 PT	Narrow	A
Pulmonary infarction	10037410 PT	Narrow	A
Pulmonary microemboli	10037421 PT	Narrow	A
Pulmonary thrombosis	10037437 PT	Narrow	A
Pulmonary vein occlusion	10068690 PT	Narrow	A
Pulmonary veno-occlusive disease	10037458 PT	Narrow	A
Pulmonary venous thrombosis	10037459 PT	Narrow	A
Renal vein embolism	10038547 PT	Narrow	A
Renal vein occlusion	10056293 PT	Narrow	A
Renal vein thrombosis	10038548 PT	Narrow	A
Retinal vein occlusion	10038907 PT	Narrow	A
Retinal vein thrombosis	10038908 PT	Narrow	A
SI QIII TIII pattern	10068479 PT	Narrow	A
Splenic vein occlusion	10068122 PT	Narrow	A
Splenic vein thrombosis	10041659 PT	Narrow	A
Subclavian vein occlusion	10079164 PT	Narrow	A
Subclavian vein thrombosis	10049446 PT	Narrow	A
Superficial vein thrombosis	10086210 PT	Narrow	A
Superior sagittal sinus thrombosis	10042567 PT	Narrow	A
Superior vena cava occlusion	10058988 PT	Narrow	A
Superior vena cava syndrome	10042569 PT	Narrow	A
Thrombophlebitis	10043570 PT	Narrow	A
Thrombophlebitis migrans	10043581 PT	Narrow	A
Thrombophlebitis neonatal	10043586 PT	Narrow	A
Thrombosed varicose vein	10043605 PT	Narrow	A
Thrombosis corpora cavernosa	10067270 PT	Narrow	A
Transverse sinus thrombosis	10044457 PT	Narrow	A
Vena cava embolism	10047193 PT	Narrow	A
Vena cava filter insertion	10048932 PT	Narrow	A
Vena cava filter removal	10074397 PT	Narrow	A
Vena cava thrombosis	10047195 PT	Narrow	A
Venogram abnormal	10047209 PT	Narrow	A
Venoocclusive disease	10062173 PT	Narrow	A
Venoocclusive liver disease	10047216 PT	Narrow	A
Venous angioplasty	10077826 PT	Narrow	A
Venous occlusion	10058990 PT	Narrow	A
Venous operation	10062175 PT	Narrow	A
Venous recanalisation	10068605 PT	Narrow	A
Venous repair	10052964 PT	Narrow	A

Venous stent insertion	10063389 PT	Narrow	A
Venous thrombosis	10047249 PT	Narrow	A
Venous thrombosis in pregnancy	10067030 PT	Narrow	A
Venous thrombosis limb	10061408 PT	Narrow	A
Venous thrombosis neonatal	10064602 PT	Narrow	A
Visceral venous thrombosis	10077829 PT	Narrow	A

8. REFERENCES

Dragalin V, Fedorov V, Cheuvart B (2002) Statistical approaches to establishing vaccine safety. Statistics in Medicine, 21, 877-893: https://onlinelibrary.wiley.com/doi/epdf/10.1002/sim.1039

A. Simon Pickard, P. H. (August 2019). United States Valuation of EQ-5D-5L Health States Using an International. *elsevier* , Pages 931-941.
