# Supplementary material for: A randomised, double-blind, multicentre, placebo-controlled phase 3 efficacy, safety, and immunogenicity study of an Ad26.RSV.preF/RSV prefusion F-protein vaccine for the prevention of respiratory syncytial virus-mediated lower respiratory tract disease in adults aged 60 years and older
Source: eBioMedicine. 2026 Nov 4;133:106513. doi: 10.1016/j.ebiom.2026.106513 (PMC13760371; doi:10.1016/j.ebiom.2026.106513)
Supplement: Supplementary file 3 [file mmc3.pdf]

Independent Data Monitoring Committee Charter

VAC18193 (JNJ-64400141/JNJ-64213175)

Protocol Number(s): VAC18193RSV3001, VAC18193RSV3002, VAC18193RSV3003, VAC18193RSV3004, VAC18193RSV3005, VAC18193RSV3006

IDMC Charter covering safety reviews of the phase 3 efficacy study (VAC18193RSV3001) and the supportive phase 3 studies
Amendment 3

Amendment 3

**Status:**	Approved
**Date:**	27/07/2022
**EDMS number:**	EDMS-RIM-429851

Confidentiality Statement

The information provided herein contains Company trade secrets, commercial or financial information that the Company customarily holds close and treats as confidential. The information is being provided under the assurance that the recipient will maintain the confidentiality of the information under applicable statutes, regulations, rules, protective orders or otherwise.

TABLE OF CONTENTS

TABLE OF CONTENTS 2

LIST OF IN-TEXT TABLES 3

AMENDMENT HISTORY 4

ABBREVIATIONS 6

1. PURPOSE OF THE INDEPENDENT DATA MONITORING COMMITTEE CHARTER 7

2. BACKGROUND 7

3. PURPOSE OF THE INDEPENDENT DATA MONITORING COMMITTEE 11

4. ORGANIZATIONS 11
4.1. Independent Data Monitoring Committee 11
4.1.1. Independent Data Monitoring Committee Members 11
4.1.2. Financial and Non-financial Disclosure and Conflict of Interest 11
4.1.3. Duration of Independent Data Monitoring Committee Membership 11
4.2. Statistical Support Group 12
4.3. Sponsor Committee 12
4.4. Study Team 12

5. ROLES AND RESPONSIBILITIES 12
5.1. Roles and Responsibilities of Independent Data Monitoring Committee Members 12
5.1.1. Independent Data Monitoring Committee Members 12
5.1.2. Independent Data Monitoring Committee Chairperson 13
5.2. Roles and Responsibilities of the Statistical Support Group 14
5.2.1. Statistical Support Group Statistician 14
5.2.2. Statistical Support Group 15
5.3. Roles and Responsibilities of the Sponsor Committee 16
5.4. Roles and Responsibilities of the Study Team(s) 16

6. INDEPENDENT DATA MONITORING COMMITTEE MEETINGS 17
6.1. Initial Meeting 17
6.2. Scheduled Meetings 18
6.3. Unscheduled Meetings 19
6.4. Meeting Report/Minutes 19

7. STATISTICAL GUIDELINE FOR PROTOCOL SPECIFIED INTERIM ANALYSIS 19
7.1. Pre-planned Interim Analysis 19
7.2. Protection of Study Integrity and Control of Operational Bias 23

8. ANALYSIS DATA REVIEWS AND COMMUNICATIONS 23
8.1. IDMC Statistical Analysis Plan 23
8.2. Data Reviews 23
8.3. Communication between the Independent Data Monitoring Committee and the Sponsor 25
8.3.1. Possible Independent Data Monitoring Committee Recommendations 25

9. PROJECTED TIMETABLE FOR INDEPENDENT DATA MONITORING COMMITTEE MEETINGS AND DATA PACKAGES 26

10. DOCUMENTATION 26

11. DATA AND COMMUNICATION FLOWCHART 28

LIST OF IN-TEXT TABLES

TABLES

Table 1: Summary 9
Table 2 Alpha-spending at the Prespecified Analysis Timepoints 21

ATTACHMENTS

Attachment 1: Independent Data Monitoring Committee Members 29
Attachment 2: Sponsor Committee Members 30
Attachment 3: Statistical Supporting Group 31
Attachment 4: Independent Data Monitoring Committee Meeting Report 32
Attachment 5: Checklist for Quality Review of Prepared Tables/Listings 34
Attachment 6: Checklist of Documents Prepared for Independent Data Monitoring Committee Members 35
Attachment 7: Plan for Communication of Independent Data Monitoring Committee (IDMC) Recommendations and Sponsor Actions VAC18193RSV3001/2/3/4/5/6 36
Attachment 8: Brief summary of the studies in scope for the IDMC (VAC18193RSV3001/2/3/4/5/6) 37

AMENDMENT HISTORY

IDMC Charter History	
**Document Type and File Name**	**Issue Date**
Initial IDMC Charter	07 July 2021
Amended Initial IDMC Charter, Amendment 1	10 November-2021
Amended Initial IDMC Charter, Amendment 2	03 March 2022
Amended Initial IDMC Charter, Amendment 3	27 July 2022

Amendment 3 (27 July 2022):

The amendment is made to update the VAC18193RSV3001 design and interim analysis plan, based on the protocol amendment 4 for the global cohort and based on amendment 5 for the China cohort. Moreover, the frequency of the safety updates was updated, based on the new design.

Additional changes, include the update on the population for the VAC18193RSV3003 and VAC18193RSV3004 studies.

Amendment 2 (14/03/2022):

The amendment is made for the following reasons:

- to add the new IDMC chair. The IDMC will now consist of 3 members (instead of 2 as described in the previous amendment).
- to align with updates in the VAC18193RSV3001 protocol amendment 3. An interim analysis by the IDMC has been added in the protocol, if the number of observed RSV cases is ≥24 to <62. If the interim analysis by the IDMC has positive results, then the study will be unblinded and the primary analysis will be performed by the sponsor.
- to include additional information on the China cohort of the VAC18193RSV3001 study.
- to align with updates in the VAC18193RSV3005 protocol, where the sample size was updated to 375 participants in each group (compared to the original sample size that was 440 per group).

Additional changes, include the need of at least two members for an IDMC meeting, in comparison to previous version where presence of all members was required.

Amendment 1 (10-November-2021):

Per initial IDMC charter, the study team should inform the IDMC members of any related SAEs within 24h of receipt. In order to allow the study team to collect the needed information, this is in agreement with the IDMC updated to 'The study team should inform the IDMC members of any related SAEs within at most 5 business days.

- This affects Section 5.4

Additionally, the IDMC chairperson has left the IDMC. The team is looking for a replacement. The contact details are removed from Attachment 1 and the language in Section 4.1 and Section 6 is updated to reflect there are 2 IDMC members now. Once the replacement is found the language will be updated again in the next amendment.

ABBREVIATIONS

Ad26	adenovirus serotype 26
AE	adverse event
CRF	Case Report Form
CRO	Contract Research Organization
IDMC	Independent Data Monitoring Committee
FOIA	Freedom of Information Act
IB	Investigator's Brochure
IVRS/IWRS	interactive voice/web response system
LRTD	lower respiratory tract disease
LRTI	lower respiratory tract infection
PA	Primary Analysis
preF	pre-fusion conformation-stabilized F protein
RSV	respiratory syncytial virus
RT-PCR	reverse transcriptase polymerase chain reaction
SAE	serious adverse event
SAS	Statistical Analysis System
SSG	Statistical Support Group
VE	Vaccine efficacy

1. PURPOSE OF THE INDEPENDENT DATA MONITORING COMMITTEE CHARTER

This document provides the plan for monitoring the safety of participants in the studies VAC18193RSV3001 (efficacy study), VAC18193RSV3002 (Coadministration study with Fluarix), VAC18193RSV3003 (End of Shelf life study), VAC18193RSV3004 (Clinical Bridging Study), VAC18193RSV3005 (Coadministration study with Fluzone High Dose) and VAC18193RSV3006 (Immunobridging study in adults between 18 and 60 years). It details the roles and responsibilities of the Independent Data Monitoring Committee (IDMC) and identifies the members of the committee. It also outlines what data will be provided to the IDMC, the process for disseminating study data, and the communication plan.

2. BACKGROUND

The respiratory syncytial virus (RSV) vaccine that will be investigated in the phase 3 studies under consideration, the Ad26/protein preF RSV vaccine (VAC18193), is a combination of 2 vaccine components, administered as a single intramuscular (IM) injection:

- Ad26.RSV.preF (JNJ-64400141), a replication-incompetent adenovirus serotype 26 (Ad26) containing a deoxyribonucleic acid (DNA) transgene that encodes the pre-fusion conformation-stabilized F protein (pre-F) derived from the RSV A2 strain.
- RSV preF protein (JNJ-64213175), a recombinant pre-F protein derived from the RSV A2 strain.

The 2 vaccine components are mixed immediately prior to administration and administered as a single intramuscular injection.

Due to the earlier availability of the clinical trial material for the Ad26.RSV.preF component of the vaccine, clinical studies were initiated first with Ad26.RSV.preF to investigate the safety and immunogenicity of this component. Following the establishment of a favorable safety and tolerability profile, and evidence that Ad26.RSV.preF induced both a humoral and cellular immune response, the ability of Ad26.RSV.preF to protect against RSV infection was shown in the human challenge study VAC19183RSV2002. Post intranasal RSV challenge, participants vaccinated with Ad26.RSV.preF shed less RSV RNA and infectious virus as well as reported fewer and milder symptoms compared to placebo.

VAC18193RSV2003 was a co-administration study conducted in 180 participants ≥60 years to assess the safety and immunogenicity of a seasonal quadrivalent influenza vaccine and Ad26.RSV.preF. Co-administration of a seasonal influenza vaccine (Fluarix®) with Ad26.RSV.preF did not impact the measured immune responses to seasonal influenza vaccine nor to Ad26.RSV.preF. There were no safety concerns

Once the clinical trial material for the RSV preF protein became available, the safety and immunogenicity of Ad26.RSV.preF mixed with RSV preF protein was studied in study VAC18193RSV1004. In this study, the added value in immunogenicity of the RSV preF protein

when combined in a single immunization with Ad26.RSV.preF has been shown. From the primary analysis, a significant increase in virus neutralizing antibodies (VNA) A2 titers was observed in the groups combining Ad26.RSV.preF and RSV preF protein compared to Ad26.RSV.preF 1×10^{11} vp alone. No relevant differences between the mixture groups and Ad26.RSV.preF alone were noted for other immunogenicity assays available at the time of primary analysis. All regimens had acceptable safety and tolerability profiles, which were similar across groups. Based on these data, the mixture of Ad26.RSV.preF 1×10^{11} vp and 150 µg of the RSV preF protein was selected for the proof of concept study VAC18193RSV2001.

In study VAC18193RSV2001 5,782 participants of at least 65 years old were randomized and vaccinated (2,891 in each group). The participants were followed for signs of RSV infection during the RSV season. From the primary analysis performed at the end of the RSV season, vaccine efficacy (VE) was demonstrated for all 3 primary endpoints, ranging from 80% (94.211% CI: 52.2, 92.9) for the most severe endpoint (Case Definition 1: RT-PCR confirmation of RSV in combination with ≥3 symptoms of LRTI) to 69.8% (94.211% CI: 43.7, 84.7) for the least severe endpoint (Case Definition 3: RT-PCR confirmation of RSV in combination with ≥2 symptoms of LRTI or ≥1 symptom of LRTI combined with ≥1 systemic symptom), demonstrating the value of the Ad26/protein preF RSV vaccine approach for the prevention of RSV disease in older adults. All identified RSV infections met Case Definition 3 which means that the VE for Case Definition 3 equals the VE for any symptomatic RSV infection. The results showed a lower incidence and higher VE with increasing severity of case definitions, which is consistent with observations for other vaccines (influenza, meningococcal, varicella). The robustness of the results was further supported by sensitivity and subgroup analyses.

The proposed indication for the Ad26/protein preF RSV vaccine (VAC18193) is for the active immunization for the prevention of lower respiratory tract disease (LRTD) caused by RSV in adults aged 18 years or older. Being an investigational new vaccine, the vaccine is not approved by any regulatory authority and so not marketed yet.

Study Design:

Table 1 provides a brief description of the studies covered under this charter. For a more extensive summary refer to Attachment 8. All details can be found in the respective protocols.

Table 1: Summary

Study	Description	N_{Active}	N_{Pbo}	Study duration	Assessments	Population
VAC18193RSV3001	Phase 3 efficacy study	15,340*<br><br>subsets:<br>>700<br>>240	7,670*<br><br>>350<br>>120	~24 months	Efficacy<br>SAEs<br><br>Safety/reactogenicity<br>Immunogenicity	≥60 years including participants at increased risk of RSV disease<br><br>Safety and immuno subsets include 50% increased risk participants and 50% of participants ≥ 75 years
VAC18193RSV3001	China cohort	2,400<br><br>subsets:<br>>116	1,200<br><br>>58	~19 months	Efficacy<br>SAEs<br>Safety/reactogenicity<br><br>Immunogenicity	≥60 years including participants at increased risk of RSV disease<br><br>Safety and immuno subsets include 50% increased risk participants and 50% of participants ≥ 75 years
VAC18193RSV3002	Influenza vaccine co-administration (Fluarix)	400 coad<br>400 sequential administration	-	~7 months	Safety/reactogenicity<br>Immunogenicity	≥60 years including participants at increased risk of RSV disease
VAC18193RSV3003	End of Shelf Life study	750 (250 near release material, 250 around the intermediate shelf-life, 250 near the presumed end of shelf life)	-	~6 months	Safety/reactogenicity<br>Immunogenicity	60-<75 years including participants at increased risk of RSV disease
VAC18193RSV3004	Clinical bridging study	240	-	~6 months	Safety/reactogenicity<br>Immunogenicity	60-<75 years including participants at increased risk of RSV disease
VAC18193RSV3005	Influenza vaccine co-administration (Fluzone HD)	375 coad<br>375 sequential administration	-	~7 months	Safety/reactogenicity<br>Immunogenicity	≥65 years including participants at increased risk of RSV disease
VAC18193RSV3006	Safety and immunogenicity bridging study	315 older adults<br>315 adults HR<br>315 adults non-HR	28 older adults<br>70 adults HR	~6 months	Safety/reactogenicity<br>Immunogenicity	Older adults: ≥60 years including participants at increased risk of RSV disease

			70 adults non-HR			Adults HR: 18-59 years of age, half of them at increased risk of RSV disease

N_{Active}: number of participants that receives Ad26/protein preF RSV vaccine, N_{Pbo}: number of participants that receives Placebo vaccine only, HR= at increased risk for severe RSV disease.
*: Additional country-specific enrollment, beyond the global cohort enrollment, may be allowed if required by local health authorities for the purpose of local regulatory approval consideration.

3. PURPOSE OF THE INDEPENDENT DATA MONITORING COMMITTEE

The primary purpose of this IDMC is to ensure the safety of the participants in these studies by monitoring safety data collected in the clinical program. The IDMC will make recommendations concerning the conduct of the study including changes to the informed consent.

In addition, the IDMC will also formally monitor the primary efficacy endpoint for the VAC18193RSV3001 study at the timepoints specified in the clinical protocol. The IDMC will evaluate in an unblinded fashion if the efficacy success criteria have been met.

Details of specific committee member roles will be further described in Section 5 below.

4. ORGANIZATIONS

4.1. Independent Data Monitoring Committee

4.1.1. Independent Data Monitoring Committee Members

The IDMC consists of at least 1 medical member with experience in respiratory disease and a statistician knowledgeable about statistical methods for clinical studies. IDMC members are not investigators or otherwise associated with the study.

The names of the IDMC members and the consultants to the IDMC, affiliations, and the contact information are listed in Attachment 1.

4.1.2. Financial and Non-financial Disclosure and Conflict of Interest

The IDMC members must disclose financial and non-financial/intellectual involvement in the product being developed or service on IDMCs of the same, related or competing product. Each IDMC member must evaluate his or her own potential conflicts of interest and complete the assessment of conflict of interest form. Members of the IDMC will be responsible for advising the Sponsor of any changes in financial and non-financial/intellectual interests in pharmaceutical and/or biotechnology companies, including consultancies. Any IDMC member who develops potential or significant conflicts of interest during the course of the study that could impact his/her objectivity may resign/could be asked to resign from the IDMC.

The conflict of interest assessment and confidentiality agreement of any consultants to the IDMC is the responsibility of the IDMC Chairperson, working in consultation with the Sponsor as needed (see TV-FRM-03591).

4.1.3. Duration of Independent Data Monitoring Committee Membership

The IDMC membership will cover the period from IDMC kick-off meeting before first participant enrolled in the first study until the last participant's last study visit in the last study. If a member leaves the IDMC, the Sponsor may seek a replacement.

4.2. Statistical Support Group

A Statistical Support Group (SSG) independent of the Study Team/Sponsor will support the IDMC. A SSG typically constitutes at least a Statistician (SSG Statistician), a Project Manager, and a Programmer (one person may serve more than one role). The SSG Statistician is a person who is capable of performing the duties as described in Section 5.2.1. The roles and responsibilities of the SSG are detailed in Section 5.2.

The statistics supporting group is composed of personnel from DataMap (VAC18193RSV3001) and 4Clinics (VAC18193RSV3002/3/4/5/6). Their names, roles in the project, and the contact information are included in Attachment 3.

4.3. Sponsor Committee

The Sponsor Committee is composed of the Global Medical Safety (GMS) Therapeutic Area Safety Head, at least a senior level Clinician and a senior level Statistician. One of them will serve as the Chairperson. The names of the Sponsor Committee members, their roles, and contact information are included in Attachment 2.

The Chairperson will serve as the point of contact between the IDMC and Sponsor. The roles and responsibilities of the Sponsor Committee are detailed in Section 5.3. The Sponsor Committee members must be independent of the study conduct.

4.4. Study Team

The Study Team is composed of a group of people including Clinician(s), Statistician(s), and others who are responsible for the design, conduct, and analyses of the study. The team members may be people external to the Sponsor in case the contract service is used. The roles and responsibilities of the Study Team are detailed in Section 5.4.

5. ROLES AND RESPONSIBILITIES

5.1. Roles and Responsibilities of Independent Data Monitoring Committee Members

5.1.1. Independent Data Monitoring Committee Members

Each member is responsible for maintaining strict confidentiality of the study data. Members will not share any study data or information about the study(ies) with any individual external to the IDMC. The IDMC members may contact the SSG Statistician directly with questions regarding the operational details associated with the data analyses and summary tables. The IDMC Chairperson must be copied in all correspondence from IDMC members to the SSG Statistician.

Each member will review the data package thoroughly prior to each IDMC meeting. A member who believes he or she may have a potential intellectual or financial conflict of interest during the course of review of the data must inform the Chairperson of the IDMC. In such cases, the IDMC meeting minutes must document the disclosure of the potential conflict of interest and the outcome

of the discussion, e.g., abstention of member from formulating recommendations, no action needed.

The IDMC members will perform the following major functions:

1. The IDMC will be responsible to monitor safety data at regular timepoints for study VAC18193RSV3001 (see Section 8.2) or upon Sponsor request for the other studies to ensure the continuing safety of the participants.
2. The IDMC will review summarized safety data (solicited and unsolicited AEs, SAEs and any other data applicable for the study) as appropriate.
3. The IDMC should always be prepared to review any individual event thought to be of major significance by the Study Team. In addition, safety letters for the specific study agent will be reviewed.
4. The IDMC should address the Study Team's concern provided that no unblinded information/conclusion will be implied by answering these questions.
5. The IDMC is responsible for alerting the Sponsor if they have safety concerns. In addition, they are responsible for making recommendations to the Sponsor concerning the conduct of the study including changes to the inform consent.
6. The IDMC (with the help of the SSG) is responsible for performing the interim analyses of the VAC18193RSV3001 study (as described in the CTP, Section 9.5.1) as well as formulating recommendation for the Sponsor Committee.
7. The IDMC is responsible for reviewing and agreeing to the sections of the SAPs applicable to the IDMC.

5.1.2. Independent Data Monitoring Committee Chairperson

In addition to the roles and responsibilities detailed in Section 5.1.1, the IDMC Chairperson has the following responsibilities. If the IDMC chairperson is unable to attend an IDMC meeting, then the second medical member of the IDMC will act as an interim IDMC chair.

1. Setting the meeting agenda along with the SSG Statistician and/or other member of the SSG
2. Leading and guiding discussions in both the Open and Closed sessions of the IDMC meetings
3. Making executive decisions, if necessary
4. Informing IDMC members of the completion of their responsibilities
5. Collecting feedback from IDMC members
6. Seeking consensus among the IDMC members
7. Ensuring that requests from IDMC members for additional information are communicated to the SSG Statistician and/or the Sponsor Committee Chairperson
8. Reviewing and finalizing the draft meeting minutes provided by the SSG
9. Ensuring that all points of view are adequately summarized in the minutes

10. Reporting IDMC safety issues and related recommendations to the Sponsor Committee Chairperson
11. Seek logistical input from the Sponsor Committee Chairperson prior to finalization of IDMC recommendations if necessary; ensuring that all recommendations from the IDMC are clearly formulated
12. Sign the IDMC meeting minutes and the IDMC Meeting Report summarizing the conclusions and recommendations of the IDMC from each meeting
13. Act as the contact between the IDMC and the Sponsor Committee Chairperson by discussing the issues and representing the views of the IDMC with the Sponsor Committee Chairperson without jeopardizing the integrity of the data
14. Ensuring that the IDMC recommendations related to the interim efficacy analysis results for the VAC18193RSV3001 study are provided to the Sponsor Committee Chairperson at the same day of the meeting.
15. Ensuring that the IDMC recommendations regarding safety updates are provided to the Sponsor Committee Chairperson within 1 day of each meeting
16. Informing the Sponsor Committee Chairperson of the need for additional IDMC meetings and identified issues, proposed meeting date(s), and specifications for data review

5.2. Roles and Responsibilities of the Statistical Support Group

5.2.1. Statistical Support Group Statistician

The SSG Statistician has the following major responsibilities:

1. Reviewing the SAP and DPS including mockup tables, listings, and graphs
2. Overseeing the SSG programming staff to ensure that the tables are programmed as intended based on the mock-ups as per the DPS, conducting a quality control review of all tables and listings (programmed and hand-tabulated) using the SSG organization's procedures for QC review prior to sending each data package to the IDMC members, a checklist (Attachment 5) that documents this review must be signed by the SSG Statistician
3. Obtaining the treatment assignment data from the IWRS vendor (Signant Health for VAC18193RSV3001, 4G for VAC18193RSV3002/3/4/5/6) or SDOffice.
4. Working with the SSG programming staff to maintain an archive of electronic copies of the datasets and the SAS programs used to generate IDMC reports
5. Providing selected summary tables and listings with test data and dummy treatment assignments to the Study Statistician for review prior to production of the first data package and for other packages as needed.
6. Serving as a liaison between the IDMC members and the Sponsor Committee when the need arises for additional information

5.2.2. Statistical Support Group

The SSG has the following major responsibilities:

1. Requesting transfers of the clinical datasets from the Sponsor or Data management CRO, as applicable and randomization code from the IWRS vendor (Signant Health for VAC18193RSV3001, 4G for VAC18193RSV3002/3/4/5/6) or SDOffice.
2. Programming and validating analyses
3. Merging the randomization data and producing programmed tables for IDMC review
4. For the VAC18193RSV3001 study , the SSG is responsible of merging the randomization data with the ADAMs that are provided by the study team, producing the primary efficacy table and presenting it to the IDMC members at the pre-specified interim analysis timepoints.
5. Sending the data package to IDMC members
6. Serving as a minute-taker to appropriately document the Closed sessions of the IDMC meetings
7. Coordinating scheduling IDMC meetings and notifying IDMC meeting attendees and the Sponsor's Study Statistician and Sponsor Committee Chairperson of the meeting dates
8. Ensuring that the appropriate documents/data packages (e.g., protocol, Investigator's Brochure (IB), Informed Consent Form (ICF), and any amendments to these documents) are compiled and sent to the IDMC members in accordance with the established timeframes and obtaining acknowledgement that the IDMC members received the documents/data packages. A checklist to facilitate this process must be utilized and is provided in Attachment 6.
9. Requesting the personal working documents from the IDMC members and destroying them in a secure manner following consultation with Sponsor to verify no hold notices exist for the drug. In case the IDMC package is delivered electronically to the IDMC members and the members did not add personal notes to these documents, the IDMC members should provide written confirmation that they deleted this from their servers.
10. Performing a quality review on documents received from the IDMC (e.g., evaluation of forms, meeting minutes)
11. Collecting all personal working documents from the IDMC members. The checklist in Attachment 6 should be used to determine if all documents/data packages that were sent to the IDMC members have been returned. See Section 10 for information on what constitutes a personal working document and what should be done with the documents once they are collected from the IDMC members. In case only electronic documents were used (so no paper documents), the SSG should request confirmation from the IDMC members that the IDMC members have deleted the electronic documents received from their servers.
12. Archiving the data packages, meeting minutes, evaluation forms, and any other documents that were needed during the course of the IDMC activities (e.g., additional requests for information) in a secured file. This archived documentation must be sent to the Study Team at the end of the trial according to the Sponsor's specifications. Prior to sending the documentation to the Sponsor Committee, the SSG Statistician should ensure that the electronic files are readable/useable. After receiving the archived documentation, the Study

Team must verify that all of the appropriate documentation has been returned and that all of the electronic files are readable/useable

5.3. Roles and Responsibilities of the Sponsor Committee

The Sponsor Committee is responsible for communicating the IDMC recommendations within the Sponsor and identifying appropriate actions based on the recommendations of the IDMC. In addition, the Sponsor Committee is responsible for:

1. Agreeing with the constitution of the IDMC
2. Agreeing to the IDMC charter and the section of the SAPs applicable to the IDMC and approving the IDMC charter
3. Addressing Study Team concerns with the IDMC
4. Communicating the IDMC recommendations to Study Team(s)
5. Requesting additional information, even unblinded summary data, from the IDMC if the IDMC recommends stopping or making any major unplanned modification to the study. However, the unblinded information will not be shared with the Study Team until the study is completed
6. Communicating the action(s) taken to the IDMC

5.4. Roles and Responsibilities of the Study Team(s)

The Study Team has the following major responsibilities:

1. Providing the SSG/IDMC with relevant information regarding the drug and conduct of clinical trials
2. Providing the SSG with the appropriate database in accordance with the established timeframes, by requesting transfers of the clinical datasets and randomization code from IWRS to the SSG
3. For the VAC18193RSV3001 study, at the time of interim analysis the team (statistical programming and statistician) will provide the SSG with fully qc-ed blinded ADAMs and a draft code to run the primary analysis model
4. Informing IDMC members of any related SAEs within at most 5 business days.
5. Requesting CIOMS from Global Medical Safety (GMS) if asked by the IDMC
6. Preparing (study statistician) and approving the SAP
7. Preparing (study statistician/study programmer) and approving the IDMC DPS including mockup tables, listings, and graphs
8. Ensuring the SAP and IDMC DPS is agreed by the IDMC members, the Sponsor Committee, the SSG and the Study Team
9. Before each IDMC meeting, provide a list of concerns (if any) to the Sponsor Committee. If in agreement, the Sponsor Committee will provide the list to the IDMC.
10. Serving as a minute-taker (study statistician) to appropriately document the open sessions of the IDMC meetings

11. Coordinating (study statistician) IDMC meetings and notifying IDMC meeting attendees and the Sponsor's Study Statistician and Sponsor Committee Chairperson of the meeting dates
12. Communicating the Sponsor decision/action to appropriate external parties, e.g., global regulatory authorities, IRB and the study investigators as necessary

6. INDEPENDENT DATA MONITORING COMMITTEE MEETINGS

A IDMC meeting may be either a teleconference (preferred) or a face-to-face meeting, unless the type of meeting is specified. The IDMC meeting may include an open session. Sponsor Study Team members may present pertinent study information to the IDMC members during the open session. Investigators or experts serving as ad hoc advisors may be requested to attend an open session of the meeting. The closed session will be limited to the IDMC members, consultants to the IDMC if needed, and designated staff from the SSG for presentation of the unblinded data. An executive session can be called with IDMC members only. Sponsor and SSG members are excluded from the executive session. A joint session may be held with all representatives from the IDMC, Sponsor, and SSG after the closed/executive session is completed.

All IDMC meetings require attendance of at least two IDMC members , all members attending the meeting have to consent on the recommendation. For the efficacy evaluation of the VAC18193RSV3001 study, at least one medic and one statistician need to attend the meeting. The Chairperson (or interim Chairperson) has the authority to cancel or change the meeting if he feels more members should attend for the decision.

For safety updates that the IDMC receives in regular time intervals, review is required from all IDMC members and the IDMC recommendation needs to be reviewed and signed by the IDMC chair.

6.1. Initial Meeting

All IDMC members will be required to attend the initial organizational meeting. The purposes of the meeting are as follows:

1. Present relevant background safety data for the study vaccine
2. Review the study protocols
3. Review the IDMC charter, including roles and responsibilities and the communication plan
4. Discuss whether the IDMC will be unblinded only to group level summaries by treatment or to individual treatment assignment. The Sponsor recommends that the IDMC review unblinded data. If the IDMC desires to remain blinded, a rationale must be provided.
5. Review table and listing mockups (typically provided in the IDMC DPS)
6. Select tentative timing of IDMC meetings, especially the first scheduled IDMC meeting

The Sponsor will set up the organizational meeting. The attendees will include the IDMC members, the SSG Statistician, and any other representatives from the SSG and the Sponsor's Study Team.

6.2. Scheduled Meetings

For trial VAC18193RSV3001, the IDMC members will receive at regular timepoints (for frequency, see Section 8.2) an electronic package of safety data to review. In principle there will be no meeting unless this is requested by one of the IDMC members or by the Sponsor. All members need to confirm that they received and reviewed the safety package and indicate whether a meeting is needed or not. **Even if there is no meeting required, the IDMC chair must provide the IDMC recommendations to the SSG.**

In addition, the IDMC will perform the 3 efficacy interim analyses, at the timepoints specified in Section 9.5.1 of the VAC18193RSV3001 CTP. The IDMC will evaluate in an unblinded fashion if the success criteria have been met. The IDMC will assess the null hypothesis of LL ≤20% for VE against RT-PCR-confirmed RSV-mediated LRTD versus the alternative hypothesis of LL >20%. For VE to be demonstrated for this primary endpoint additionally, the observed VE should be >50%. Refer to Section 9.1 of the VAC18193RSV3001 CTP for details on the hypothesis testing.

Moreover, for the local China cohort of the VAC18193RSV3001 trial the IDMC will convene in case one of the prespecified pausing rules, specific to China has been met. The occurrence of any of the following events will lead to pausing of enrollment in the China cohort until the IDMC can hold a meeting to discuss the event(s) and possible follow-up actions, including study suspension, adaptation, or discontinuation of further vaccination:

- The discovery of an unexpected, serious, excessively frequent, or unacceptable risk to the participants enrolled in the study, OR
- Death of any participant, considered related to any study vaccine or if the causal relationship to study vaccine cannot be excluded, OR
- One or more participants experience anaphylaxis or a systemic allergic reaction that requires medical intervention clearly not attributable to other causes than vaccination with any study vaccine, OR
- More than 10% of the participants within 72 hours from vaccination experience a systemic allergic reaction that require medical intervention, OR
- 15% or more of the participants in the China cohort had an AE of a single type of Grade 3 or higher during the 28-day follow-up period after vaccination

For the other trials (VAC18193RSV3002/3/4/5/6), there are no planned safety reviews. If safety issues arise from a study, the Study Team might escalate this to the IDMC and an ad hoc safety review might be requested.

If data review meetings occur, they will occur via teleconference after the IDMC members have reviewed the data package. The SSG will set up these meetings (the study statistician might assist in scheduling the meeting) and all IDMC members, the SSG Statistician, and other SSG representatives will attend the meetings. The purpose of these meetings is to discuss the data and formulate a recommendation to the Sponsor.

6.3. Unscheduled Meetings

The IDMC or Sponsor has the option to call an unscheduled meeting at any time. The attendees for this meeting are the IDMC members and the SSG Statistician. In rare situations, such as a grave safety concern or other special circumstance, the IDMC may request that a Sponsor representative attend a portion of an unscheduled meeting.

The IDMC members should not disclose to the Sponsor the reasons behind the perceived need for unscheduled meetings or additional data requests.

6.4. Meeting Report/Minutes

IDMC meeting minutes summarizing the conclusions and recommendations of the IDMC will be drafted after each open and closed meeting, separately. A Study Team representative (open part) and a SSG representative (closed part) are responsible for preparing draft minutes of each IDMC scheduled or unscheduled meeting. This individual will ensure that the date of the meeting, a summary of the discussions, the committee recommendations, the rationale for the recommendations, and the list of attendees are included in these minutes. The Study Team (open part) and SSG (closed part) will send the draft minutes to the IDMC Chairperson who will review and modify the minutes. The IDMC Chairperson will then sign and date the finalized minutes and return them to the SSG for confidential distribution to IDMC members. The IDMC Chairperson will sign the IDMC meeting minutes. The IDMC meeting minutes should include important considerations that led to the IDMC recommendations.

The IDMC meeting report containing open session summaries, and the conclusions or recommendations without reference to unblinded data will be sent to the Sponsor within 1 working day after the meeting. A template of a meeting report is provided in Attachment 4 as a guideline. If the IDMC meeting minutes for open sessions are not included in the open session summaries mentioned above, they will be sent to the Sponsor within 5 working days after the meeting. The IDMC meeting minutes for closed sessions, however, will not be sent to the Sponsor until after the completion of the primary analysis of the studies and database lock of the relevant clinical database(s)/unblinding of the trial. All meeting minutes from each scheduled or unscheduled meeting will be archived, along with a copy of the corresponding data package, in a secure location until after database lock of the final analysis.

7. STATISTICAL GUIDELINE FOR PROTOCOL SPECIFIED INTERIM ANALYSIS

7.1. Pre-planned Interim Analysis

At most three interim analyses (IA) will be conducted prior to the primary analysis for the VAC18193RSV3001 study. The outcome of the IA can be any of the following:

- "superiority": the success criteria have been met and the sponsor can be unblinded to the results,

- "continue": the success criteria have not been met and the study should continue as planned,

The study is designed to test the primary hypothesis of VE against RT-PCR-confirmed RSV-mediated LRTD in the PPE population.

- The hypothesis for the primary endpoint is:
  - Null hypothesis: the VE against RT-PCR-confirmed RSV-mediated LRTD of Group 1 vs placebo is ≤20%.
  - Alternative hypothesis: the VE against RT-PCR-confirmed RSV-mediated LRTD of Group 1 vs placebo is >20%.

The study is successful if:

- The LL of the 95% 2-sided confidence interval (CI, corrected as described below) around the VE (1-relative risk rate) calculated from the Exact Poisson regression model is above 20% and additionally a point-estimate of VE >50% is observed for Group 1

The interim analysis plan is as follows:

- Based on a database cut-off date of 15 September 2022, an IA by IDMC will be performed. The IDMC will evaluate in an unblinded fashion if the LL of the (100-2×α_1)% 2-sided CI around the VE (1-relative risk rate) is above 20% and additionally a point-estimate of VE >50% is observed for Group 1. If the success criteria are met, the sponsor will be notified. The database used for the IDMC analysis will be unblinded for the sponsor and the primary analysis will be performed by the sponsor at the same significance level (α_1). If the success criteria are not met, the participants will be followed further, and the same steps will be performed at the following subsequent database cut-offs:
- 24 October 2022 with an alpha level α_2
- 22 January 2023 with an alpha level α_3
- Provided no primary analysis occurred earlier, the primary analysis will be performed with database cut-off 30 April 2023 with alpha level α_4

  If VE is not demonstrated to be significantly >20% with a point estimate >50% at the time of the primary analysis, the study will be stopped.

Figure 1 displays the interim and primary analysis plan.

The total alpha will be controlled at 2.5%. A Pocock-like alpha spending function as described by DeMets (DeMets 1994) with a target number of events of 76 used to spend the alpha over the different analyses. The different alphas to be used during every IA are shown above in Figure 1 and the calculations are explained below in Table 2.

Table 2 Alpha-spending at the Prespecified Analysis Timepoints

Alpha	*Description of how to obtain alpha*
α_1	Alpha obtained from Pocock-like alpha spending function with information fractions = [# RSV LRTD events by 15 September 2022 / 76[¥], 1]
α_2	Alpha obtained from Pocock-like alpha spending function with information fractions = [# RSV LRTD events by 15 September 2022 / 76[¥], # RSV LRTD events by 24 October 2022 / 76[¥], 1]
α_3	Alpha obtained from Pocock-like alpha spending function with information fractions = [# RSV LRTD events by 15 September 2022 / 76[¥], # RSV LRTD events by 24 October 2022 / 76[¥], # RSV LRTD events by 22 Jan 2023 / 76[¥], 1]
α_4	Alpha obtained from the remaining alpha to be spent (2.5% - total alpha spent during the interim analyses) and the actual information fractions observed at all analyses. That is, the information fraction, IF = [# RSV LRTD events by 15 September 2022 / # RSV LRTD events by 30 April 2023, # RSV LRTD events by 24 October 2022 / # RSV LRTD events by 30 April 2023, # RSV LRTD events by 22 January 2023 / # RSV LRTD events by 30 April 2023, 1]. The final alpha will be calculated using the user-defined alpha spending function approach (Jennison 2000).

[¥] 76 = target sample size for 90% power with 65% VE. Note that if at any of the interim analyses 76 or more RSV LRTD events have been observed, all remaining alpha will be spent and no further (interim) analyses for primary efficacy endpoint will be performed.

Figure 1: Interim and Primary Analysis Plan

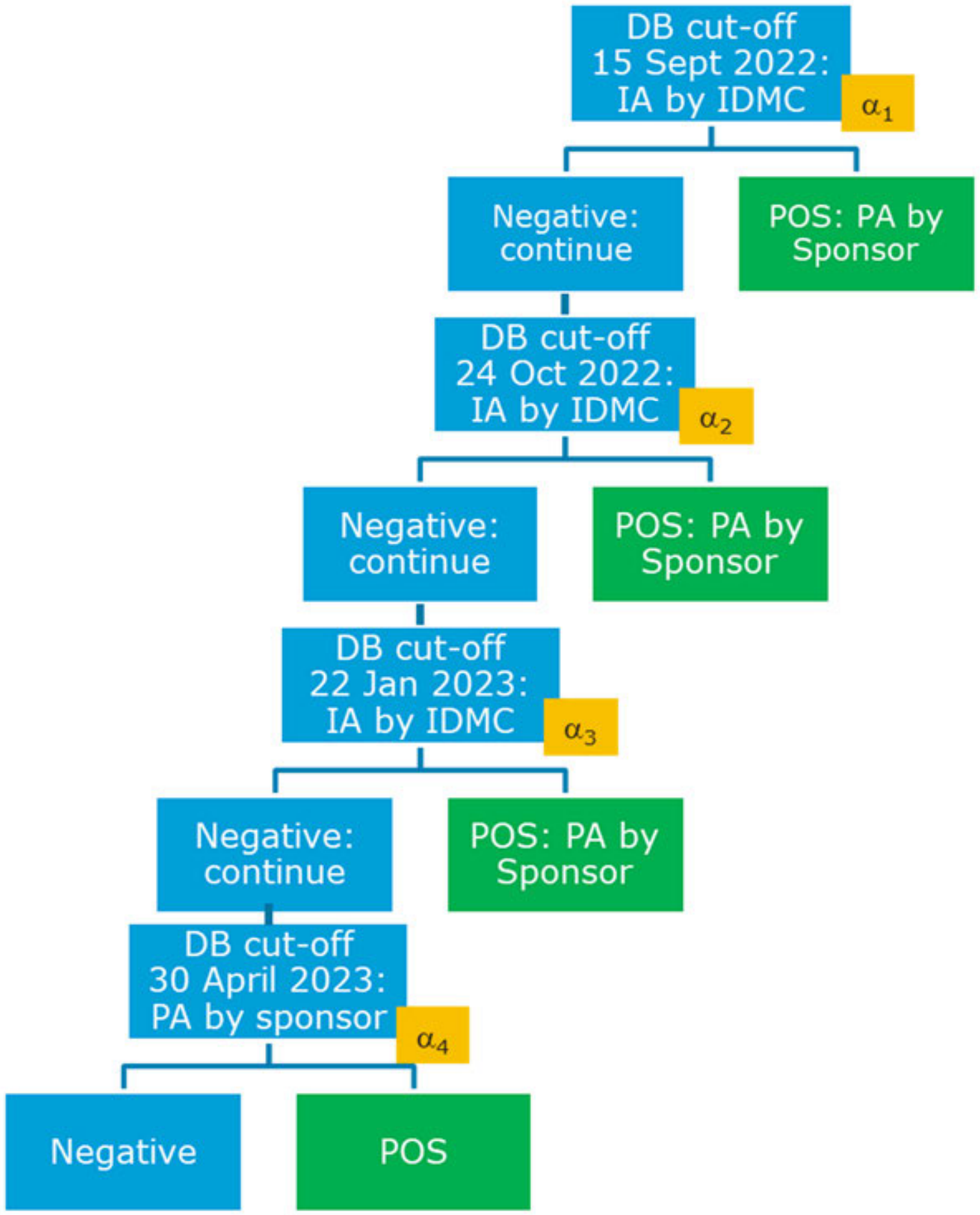

For the first interim analysis, ARIs with an onset up to 1 September 2022 will be included and a database cut-off of 15 September 2022 will be used. For the second IA, ARIs with an onset up to 10 October 2022 will be included and a database cut-off of 24 October 2022 will be used. For the third IA, ARIs with an onset up to 8 January 2023 will be included and a database cut-off of 22 January 2023 will be used. For the primary analysis, ARIs with an onset up to 15 April 2023 will be included and a database cut-off of 30 April 2023 will be used.

DB= database, IA= interim analysis, IDMC = independent data monitoring committee, PA=primary analysis, PPE=Per-protocol Efficacy, POS= positive

7.2. Protection of Study Integrity and Control of Operational Bias

The integrity of the data is ensured as follows:

- Counting the number of events to decide if an interim analysis or primary analysis will be performed will be done on blinded data.
- Interim analyses performed before the primary analysis will be done by the IDMC. The sponsor will remain blinded to the actual results of any IA and will only receive the outcome (superiority, continue). Unblinding of the sponsor occurs at the time of the primary analysis.

8. ANALYSIS DATA REVIEWS AND COMMUNICATIONS

8.1. IDMC Statistical Analysis Plan

For each of the studies, the Study Statistician will draft the SAP including a section on the analysis plan for the review of safety data by the IDMC. The section(s) of the SAPs applicable to the IDMC must be agreed to by the IDMC members, the Study Team, and the Sponsor Committee. The SAPs provide the details of the statistical methods and analyses to be applied to the data. The section of the SAPs applicable to the IDMC also describes in detail the scope of the data, the data cut-off criteria for each IDMC review, the contents to be included in the data package, and whether and how the treatment groups will be blinded in the data package.

8.2. Data Reviews

1. Type of data review and frequency of each type of review:
    - VAC18193RSV3001: The IDMC will receive monthly safety updates, starting 6 weeks after the first participant receives vaccination until the day that the last participant in the safety subset reached his/her 28- day post dose visit, and updates every 2 to 3 months until the end of the study. In principle there will be no meeting, unless this is requested by one of the IDMC members or by the Sponsor.
    - VAC18193RSV3001: In addition, interim analyses for efficacy will be performed by the IDMC as outline in section 7.1.
    - VAC18193RSV3001 China cohort: In addition to the monthly safety updates, if a pausing rule is met for the China cohort, the team will request an ad hoc safety review by the IDMC.
    - Other studies: There are no planned reviews. If safety issues arise in one of the studies, the team might request an ad hoc safety review by the IDMC. In such a case, a meeting will be scheduled only if this is requested by the Study Team or the IDMC.
    - Note: once the primary analysis for each study is performed by the Sponsor, the Sponsor will share the primary analysis results with the IDMC.

2. Data handling:

    - For the safety updates, no special data cleaning procedures will apply. Data will be cleaned on an ongoing basis. The Study Team will transfer the blinded data to the SSG and the IWRS vendor (Signant Health for VAC18193RSV3001, 4G for VAC18193RSV3002/3/4/5/6) or SDOffice will transfer the randomization code to the SSG a few of days prior to the planned safety review.

    - For the efficacy interim analyses performed by the IDMC, database cut-off dates are specified in section 7.1. Data used for the different interim analyses should be cleaned. Two days (end of business) prior to the IDMC meeting, the Study Team will transfer the blinded ADAM datasets to the SSG and the IWRS vendor (Bracket) or SDOffice will transfer the randomization code to the SSG. Outputs are expected within 1 working day of receiving the data.

3. Unblinding: Summary tables for the IDMC will be displayed with real treatment identity. A separate data package might be made available to the Study Team where the summary data are presented for the pooled groups (blinded).
4. Distribution of data package: Data packages will be distributed by the SSG to the IDMC members via a secure electronic environment. All data and output should be held in strict confidence. The data package will be sent to the IDMC members no later than 1 business day prior to the scheduled meeting.

IDMC data packages should begin with a protocol synopsis of the study under consideration, a listing of new amendments to the considered protocol (if any), a reminder of previous recommendations of the IDMC, an explanation of complex medical or statistical issues that may underlie the interpretation of the data if any, and a copy of the informed consent form so that the IDMC knows of the adverse reactions about which the participants are aware. The data package should include the tables and figures as specified in the IDMC DPS.

In the event that the IDMC requires additional safety information beyond the standard summary tables and listings that were provided, the request will be made to the SSG Statistician. The SSG will provide all analytical support for this effort and will forward the results to each of the IDMC members. If the SSG Statistician does not have the required information, he/she will request that the Study Statistician obtain the information. The information request should be worded in such a way that the Study Statistician does not know the intent of the data inquiry, if possible. In the event that the Study Statistician is not able to appropriately address the request, the Study Clinician may provide additional information. Additional data requests should be limited to those that are considered essential by the IDMC.

The IDMC must not divulge the contents of the unblinded data to members of the Study Team or to any members of the Sponsor Committee unless specifically requested by the Sponsor Committee Chairperson, until the study has completed.

8.3. Communication between the Independent Data Monitoring Committee and the Sponsor

8.3.1. Possible Independent Data Monitoring Committee Recommendations

The possible recommendations the IDMC members can make are:

1. To continue the study(ies) unmodified until next scheduled meeting
2. To continue the study(ies) unmodified and plan an additional meeting
3. To continue the study(ies) unmodified, and request additional expert review/analyses
4. To continue study(ies) and amend protocol(s)
5. After IDMC review of VAC18193RSV3001 efficacy Interim Analysis: To start the primary analysis, due to superiority
6. After IDMC review of VAC18193RSV3001 efficacy Interim Analysis: To continue with the study as described in the Protocol
7. To set up a meeting with the Sponsor Committee to discuss concerns of safety and/or efficacy within the clinical study(ies)
8. To stop the study(ies)

Options 5 and 6 are specific to the interim analysis of the VAC18193RSV3001 study. In the recommendation it should be clearly specified for which study the recommendation applies. And in case a pausing rule in China cohort of the VAC18193RSV3001 study is met, the recommendation should specify if the recommendation is applicable only for that cohort or the global study.

The IDMC recommendations, which are sent to the Sponsor Committee Chairperson, will include IDMC conclusions and recommendations without reference to unblinded data. It should be clearly indicated which studies were reviewed and for which studies the recommendations are applicable. For study VAC18193RSV3001, there are planned reviews, but there will only be a meeting if requested by the IDMC members or sponsor. **Even if there is no meeting required, the IDMC chair must provide the IDMC recommendations to the SSG. The recommendation from a meeting regarding safety is expected within 1 days of the meeting, and the recommendation from a meeting regarding efficacy is expected at the same day as the meeting.**

The IDMC should provide rationale for their recommendation if the recommendation is anything other than continuing the study without modification. The IDMC Chairperson should call the Sponsor Committee Chairperson to communicate the IDMC recommendation and should also send an evaluation form to the Sponsor Committee Chairperson conveying the same information.

The plan for communicating IDMC recommendations and sponsor actions is included in Attachment 7.

Concerns about Participants' Safety

If the IDMC determines that there is a safety risk for participants in the trial, they will note their concerns on the IDMC meeting report, and the Chairperson will alert the Sponsor Committee Chairperson via a phone call. The Sponsor Committee Chairperson will immediately notify the GMS Therapeutic Area Head of the issues. The GMS Therapeutic Area Head will call an urgent meeting to discuss the safety issues. The meeting attendees should be the members of the Sponsor Committee, members from the Safety Management Team (SMT) who are not involved in the study directly, and, at a minimum, the IDMC Chairperson or delegate. The Sponsor Committee Chairperson or delegate is responsible for recording the minutes of this meeting and getting approval for the final minutes from the meeting attendees. Once the Sponsor has the appropriate information from the IDMC, they will make a decision regarding the possible modification of the study design or termination of the trial.

The intention to modify or stop the study by the Sponsor may then be made in consultation with the regulatory agencies. The final decision will be conveyed to the Study Team, the IDMC, and the investigators along with the rationale for any modifications to the trial. The Study Team will implement the required changes.

9. PROJECTED TIMETABLE FOR INDEPENDENT DATA MONITORING COMMITTEE MEETINGS AND DATA PACKAGES

Initial Meeting

The organizational kick-off meeting will be scheduled approximately 1 month prior to the enrollment of the first participant in the trials under consideration.

First Participant Enrolled

The first participant is expected to be enrolled by 22 July 2021.

First set of data package

For VAC18193RSV3001, the first data package including a safety summary of grade 3 adverse events and SAE data should be available approximately 6 weeks after the enrollment of the first participant in this study.

For the other studies, there are no scheduled reviews.

10. DOCUMENTATION

Original documents (meeting agendas, meeting minutes, safety reports/tables/line listings, IDMC members' faxed IDMC meeting reports, QC checklists, document checklists, IDMC members' faxed signature pages, correspondence, etc.) will be managed by the SSG Statistician.

For each of the studies: After evaluation of the last safety data and study completion, the SSG Statistician will be responsible for transferring all final documents to the Study Team for filing in the central file (as outlined in Section 6.4).

Personal working documents (copies of safety reports/tables/line listings, safety reports/tables/line listings with handwritten notes, notes taken during a meeting, original signature IDMC meeting reports, correspondence, etc.) reviewed or created by a IDMC member to support or back-up decisions captured in the minutes are also participant to the Sponsor's current record retention schedule or policy.

IDMC members should retain all the personal working documents during the study and return them to the SSG Statistician at the end of their participation. Alternatively, the personal working documents may be returned to the SSG Statistician during the trial if they are no longer needed. In case only electronic documents were used (so no paper documents), the SSG should request confirmation from the IDMC members that the IDMC members have deleted the electronic documents received from their servers. When the IDMC's participation has ended, the SSG Statistician will then need to contact the Study Team to inquire whether there are any hold notices on VAC18193. The Study Team will review their current retention procedures including possible company hold notices. If there are no active hold notices on VAC18193, the Study Team will inform the SSG Statistician that the working documents, with the exception of the original signed IDMC meeting reports, should be destroyed as soon as possible in a secure manner. If there is a hold notice on VAC18193, the SSG Statistician will forward the documents to the Study Team upon request. In either situation, the original signature IDMC meeting reports must be returned to the Study Team.

The Study Team will compile and maintain the following documents:

- Copy of the IDMC charter (and all amendments to the charter) and associated attachments and addenda, SAP for each study, IDMC DPS
- Copy of the IB
- Protocols and protocol amendments for the clinical studies pertinent to the IDMC
- Curriculum vitae for each IDMC member and conflict of interest form
- Curriculum vitae for staff of the SSG
- Copies of all correspondence between the Study Team and the IDMC

The SSG will compile and maintain the following documents:

- Copies of the data package provided to the IDMC members
- Minutes of each IDMC meeting, including conclusions or recommendations concerning the conduct or evaluation of the study(ies) and any important considerations that led to the conclusions/recommendations
- IDMC reports provided to the Sponsor Committee Chairperson containing conclusions or recommendations without reference to unblinded data
- Copies of all correspondence between the SSG and the IDMC

Upon completion of the study(ies) and closure of the relevant clinical database(s), the documents will be forwarded to the Study Team for archiving.

11. DATA AND COMMUNICATION FLOWCHART

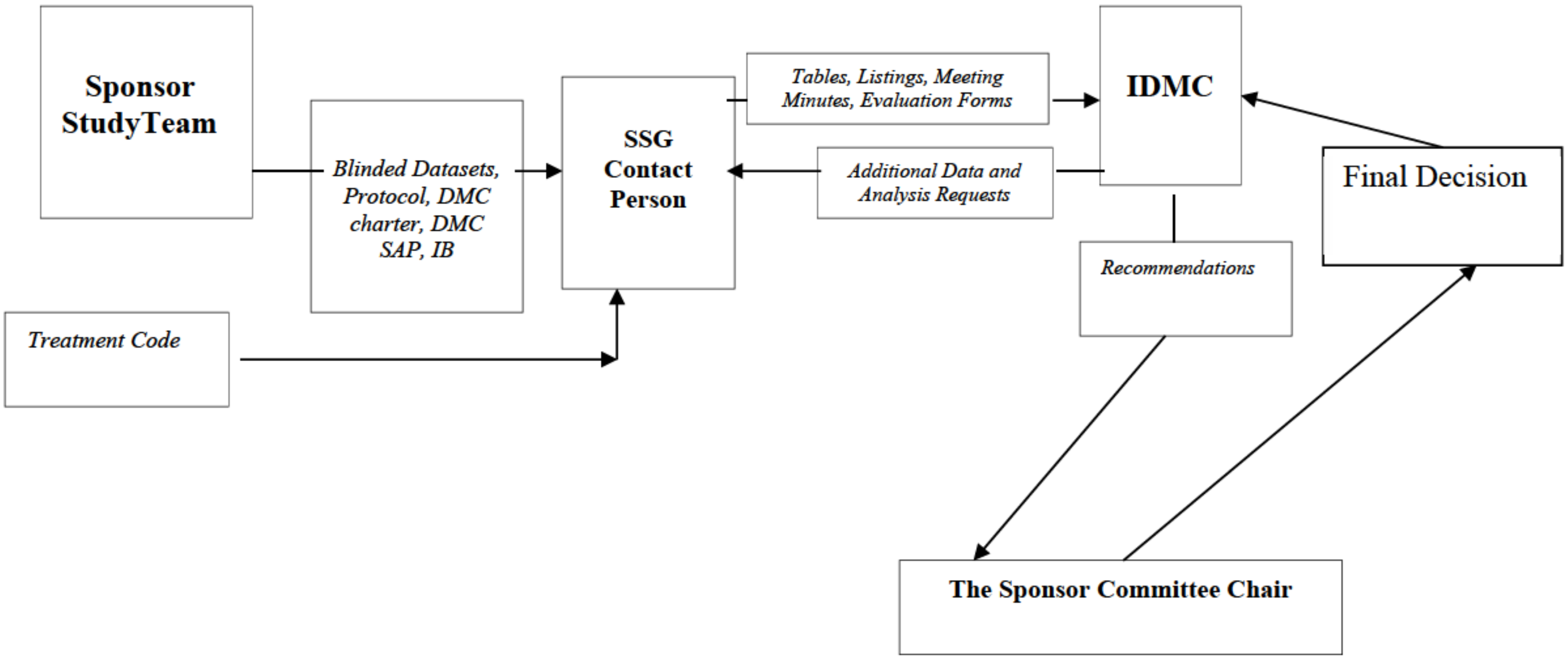

Attachment 1: Independent Data Monitoring Committee Members

IDMC Chairperson:

Name:	PPD
Affiliation:	Harvard Medical School
Address:	Division of Infectious Diseases, Birgham and Women's Hospital,<br>75 Francis Street,<br>PPD, Boston, MA 02115
Phone:	PPD
Fax:	PPD
E-mail address:	PPD

Clinical Specialist:

Name:	PPD
Affiliation:	Duke University Medical Center
Address:	PPD<br>Hanes House, Trent Drive<br>Durham, NC 27710
Phone:	PPD
Phone (cell):	PPD
Fax:	PPD
E-mail address:	PPD

Statistician:

Name:	PPD.
Affiliation:	Universiteit Hasselt
Address:	Martelarenlaan 42, 3500 Hasselt, Belgium
Phone:	PPD
Fax:	PPD
E-mail address:	PPD

Attachment 2: Sponsor Committee Members

Chairperson (Clinician):

Name: PPD

Phone: PPD
Fax:
E-mail address: PPD

GMS Therapeutic Area Safety Head:

Name: PPD

Phone: PPD
Fax:
E-mail address: PPD

Statistician:

Name: PPD

Phone: PPD
Fax:
E-mail address: PPD

Attachment 3: Statistical Supporting Group

4Clinics (SSG for VAC18193 RSV3002/3/4/5/6)

Statistician:

Name:	PPD
Phone:	PPD
Fax:	
E-mail address:	PPD

Project Manager:

Name:	PPD
Phone:	PPD
Fax:	
E-mail address:	PPD

Programmer:

Name:	PPD
Phone:	PPD
Fax:	
E-mail address:	PPD

DATAMAP GmbH (SSG for VAC18193RSV3001)

Statistician:

Name:	PPD
Phone:	PPD
Fax:	PPD
E-mail address:	PPD

Project Manager:

Name:	PPD
Phone:	PPD
Fax:	PPD
E-mail address:	PPD

Programmer:

Name:	PPD
Phone:	PPD
Fax:	PPD
E-mail address:	PPD

Attachment 4: Independent Data Monitoring Committee Meeting Report

To: Sponsor Committee Chairperson
Meeting Date:
Protocol: [Specify the protocols reviewed in the considered meeting]
Meeting Attendees:

The IDMC charged with the review of safety data for the VAC18193 clinical study(ies) reviewed Data package number [ ] dated [dd/Mon/yyyy] considering studies [specify the protocol nrs reviewed].

Summary of discussion in the open session of the meeting:

As a result, the IDMC recommendation is:

☐ To continue study unmodified until next scheduled meeting

☐ To continue study unmodified, and plan an additional meeting
The following date is proposed for the additional meeting: [dd/Mon/yyy] (to be confirmed with Sponsor Committee Chairperson)

☐ To continue study unmodified, and request additional expert review/analyses

[Describe and provide timelines of additional review:]

☐ To continue study and amend protocol(s) as described:

[Describe sections below and list protocol(s) to be amended]

☐ After IDMC review of VAC18193RSV3001 efficacy Interim Analysis: To start the primary analysis, due to superiority

☐ After IDMC review of VAC18193RSV3001 efficacy Interim Analysis: To continue with the study as described in the Protocol

☐ To stop the study(ies):

[Indicate which study(ies) should be stopped]

☐ To set up a meeting with the Sponsor Committee to discuss concerns of safety within the clinical study as outlined below, within [#] weeks after the IDMC meeting

Additional Comments:

Chairperson, Independent Data Monitoring Committee for Study [insert study number(s)]	Date

Attachment 5: Checklist for Quality Review of Prepared Tables/Listings

Protocol VAC18193[insert number] [JNJ-64400141/JNJ-64213175]

☐ Planned data package

☐ Additional requests

☐ I have reviewed all of the hand-tabulated tables and/or listings for accuracy.

☐ I have reviewed all of the programmed tables and/or listings for accuracy.

Signature Date (dd/MMM/yyy)

Attachment 6: Checklist of Documents Prepared for Independent Data Monitoring Committee Members

Protocol VAC18193[insert study number] (JNJ-64400141/JNJ-64213175)

☐ Planned data package ☐ Additional request review
☐ Other, specify:

For SSG Statistician or designee:

I have prepared the following documents for the IDMC members

- ☐ Final IDMC charter
- ☐ IDMC charter amendment
- ☐ ICF, updated ICF
- ☐ IB, updated IB
- ☐ Protocol [specify study number]
- ☐ Protocol amendment [specify study number]
- ☐ SAP/ IDMC DPS [specify study number]
- ☐ Safety letters
- ☐ Planned data package [specify study number]
- ☐ Additional requests
- ☐ Other: [specify]

Signature Date (dd/MMM/yyy)

For IDMC member:

- ☐ I have received all the documents listed above.
- ☐ I have the following items missing from the package: [specify].
- ☐ I have deleted all the electronic documents received for this study from my server. [specify]

Signature Date (dd/MMM/yyy)

For SSG Statistician or designee:

- ☐ I received confirmation that the IDMC members have deleted the electronic documents received from their servers
- ☐ I have verified that all the documents from the IDMC members have been returned.
- ☐ Not all the documents were returned from the IDMC members. [Specify the missing items and reasons (if the IDMC member is not able to produce these items)]

Signature Date (dd/MMM/yyy)

Attachment 7: Plan for Communication of Independent Data Monitoring Committee (IDMC) Recommendations and Sponsor Actions VAC18193RSV3001/2/3/4/5/6

Responsible Person/Actions	Estimated Timing	Audience
1. Sponsor Committee Chairperson informs CDTL and others of recommendations/actions from the IDMC<br>• [If the recommendation is to continue the trial with a major unplanned change or to terminate the trial, the Sponsor Committee Chairperson should inform additional appropriate management. Note that certain selected Sponsor individuals may be unblinded if the recommendation is trial termination.]	[insert date(s)]	Communicate all IDMC recommendations to:<br>• Sponsor Committee member(s)<br>• CDTL<br>• Safety Management Team<br><br>The CDTL is responsible for notifying the core members of the CDT. In turn, these individuals are responsible for appropriate communication in their departments/organizations.<br><br>The CDTL may inform others given the nature of the recommendations.
2. If there is a planned or unplanned adaptation to the study design per IDMC recommendation and Sponsor Committee approval, such as changes in the number of treatment arms or sample size (that will impact drug supply/clinical supply chain), the Sponsor Committee Chairperson informs the appropriate Clinical Biostatistics Head/Medical Affairs Statistics Head of the recommendation.		Clinical Biostatistics Head/Medical Affairs Statistics Head communicates IDMC recommendations to:<br>• IVRS/IWRS Supplier<br>• Unblinded External Partnerships Manager<br>• Clinical Supply Chain
**Note** Further notification of recommendations/action plan beyond this point only as necessary when there is a MAJOR unplanned change in the trials, when the trial is to be terminated, or when circumstances warrant notification of new safety information. The CDTL will determine/approve further notification in consultation with the CDT members, Management, and Communications.		

The CDTL in collaboration with the Global Therapeutic Area Head or Medical Affairs equivalent may determine that communication to others is warranted, including but not limited to:

- Johnson & Johnson Legal Department (in the event an SEC release is required)
- R&D Directors of the local operating companies participating in the trials
- Regional R&D Directors
- Health Authorities
- Clinical Research Organizations involved in trial execution
- Investigators and Key Opinion Leaders

Attachment 8: Brief summary of the studies in scope for the IDMC (VAC18193RSV3001/2/3/4/5/6)

VAC18193RSV3001 is a randomized, double-blind, multicenter, placebo-controlled Phase 3 study aiming to demonstrate the efficacy of the Ad26/protein preF RSV vaccine in the Prevention of Lower Respiratory Tract Disease Caused by RSV in Adults Aged 60 Years and Older. Up to approximately 27,500 participants (with up to 23,000 in the Global cohort, up to approximately 4,500 participants in specific Asian countries under local protocols, in China ~3600 participants) will be enrolled and randomized in parallel in a 2:1 ratio to 1 of 2 groups to receive active study vaccine or placebo.

Note that some additional local cohort enrollment, beyond the global cohort enrollment may be allowed if required by local health authorities for the purpose of local health authority consideration.

- The Global cohort is defined as all participants (including the Safety subset and the Immuno subset) who are recruited up to the point that global enrollment is stopped.
- Local cohorts are defined as participants from the considered country/region and under local protocols.

From the study vaccination until 24 months post vaccination (ie, the full duration of the study), participants will be followed-up to identify potential cases of RSV infection.

SAEs will be collected in all participants from administration of study vaccine (Day 1) until 6 months after vaccination. AEs leading to discontinuation from vaccination and/or study will be collected for the duration of the study.

Solicited AEs during the first 7 days after vaccination and unsolicited AEs during the first 28 days after vaccination are captured in a subset of at least 1050 subset, the so-called ‘Safety Subset’.

Blood samples for humoral immunogenicity are collected at Day 1 and Day 15 in all participants. In the immunogenicity subset, which consists of at least 360 participants, humoral samples at other timepoints during the study as well as cellular samples are collected. For more details on this study, refer to the VAC18193RSV3001CTP.

Study Design of VAC18193RSV3001

Group	N	Day 1
1	15,340	Ad26/protein preF RSV vaccine (1×10^{11} vp/150 µg)
2	7,670	Placebo

Note: The study will be discontinued if the primary analysis is performed by the sponsor and the outcome is negative; however, if at this timepoint, some participants have not completed 6 months of follow-up after their vaccination, the study will continue until this follow-up period is complete.
N=number of participants, vp=viral particles

VAC18193RSV3002 is a randomized, double-blind, placebo-controlled, multicenter Phase 3 study in adult participants aged 60 years and older to study the effect of concomitant administration of Ad26/protein preF RSV vaccine and Fluarix on the immunogenicity of both vaccines.

Approximately 800 participants will be randomized in parallel in this study in a 1:1 ratio to 1 of 2 groups. Group 1 will receive Ad26/protein preF RSV vaccine on Day 1 administered at the same time as Fluarix, and placebo on Day 29. Group 2 will receive placebo on Day 1 administered at the same time as Fluarix, and Ad26/protein preF RSV vaccine on Day 29.

In all participant's solicited AEs are collected during the first 7 days after each vaccination, unsolicited AEs during the first 28 days after each vaccination and SAEs and AEs leading to discontinuation are captured during the whole study.

Blood will be collected from all participants to assess humoral immune responses pre-vaccination on each vaccination day and at 28 days after the second study vaccination

Study design of VAC18193RSV3002

Group	N	Day 1	Day 29
1 (CoAd group)	400	Ad26/protein preF RSV vaccine + Fluarix	Placebo
2 (Control group)	400	Placebo + Fluarix	Ad26/protein preF RSV vaccine

N = number of participants.

VAC18193RSV3003 is a randomized, double-blind, multicenter, interventional Phase 3 study in adult participants aged 60 years to less than 75 years to study humoral immune response of aged material. Demonstrating non-inferior humoral immune responses of aged material relative to near release material will allow to infer consistent efficacy throughout the vaccine shelf life. A target of 750 participants will be randomized in parallel in a 1:1:1 ratio to 1 of 3 groups (see table below). All participants will receive study vaccine (Ad26/protein preF RSV vaccine or placebo) on Day 1.

In all participant's solicited AEs are collected during the first 7 days after each vaccination, unsolicited AEs during the first 28 days after vaccination and SAEs and AEs leading to discontinuation are captured during the whole study.

Blood will be collected from all participants to assess humoral immune responses pre-vaccination and at 14 days and 6 months post-vaccination.

Study design of VAC18193RSV3003

Group	N	Vaccination on Day 1
1	250	Ad26.RSV.preF 1×10^{11} vp + RSV preF protein 150 µg<br>(representing critical quality attributes around release)
2	250	Ad26.RSV.preF 3.3×10^{10} vp + RSV preF protein 150 µg<br>(representing critical quality attributes around the intermediate shelf-life)
3	250	Ad26.RSV.preF 1.1×10^{10} vp + RSV preF protein 150 µg<br>(representing critical quality attributes near end of shelf-life)

N = number of participants; vp = viral particles

VAC18193RSV3004 is a randomized, double-blind, multicenter, interventional Phase 3 study in adult participants aged 60 to less than 65 years to compare the immunogenicity of clinical trial material of an Ad26.RSV.preF-based vaccine for Phase 3 with clinical trial material representative of Phase 2b in adults aged 60 to less than 75 years. A target of 240 participants will be randomized in parallel in a 1:1 ratio to 1 of 2 groups. All participants will receive Ad26/protein preF RSV vaccine on Day 1.

In all participant's solicited AEs are collected during the first 7 days after each vaccination, unsolicited AEs during the first 28 days after each vaccination and SAEs and AEs leading to discontinuation are captured during the whole study.

Blood will be collected from all participants to assess humoral immune responses pre-vaccination and at 14 days post-vaccination.

Study design of VAC18193RSV3004

Group	N	
1	120	Phase 3 CTM[a]
2	120	Phase 2b CTM[b]

CTM = clinical trial material; N = number of participants
[a] representative of future commercial manufacturing process material (Ad26.RSV.preF Lot 2 + RSV preF protein Lot b)
[b] representative of Phase 2b (VAC18193RSV2001) CTM (Ad26.RSV.preF Lot 4 + RSV preF protein Lot d)

VAC18193RSV3005 is a randomized, double-blind, placebo-controlled, multicenter Phase 3 study in adult participants aged 65 years and older to study the effect of concomitant administration of Ad26/protein preF RSV vaccine and Fluzone HD Quadrivalent on the immunogenicity of both vaccines.

Approximately 750 participants will be randomized in parallel in this study in a 1:1 ratio to 1 of 2 groups. Group 1 will receive Ad26/protein preF RSV vaccine on Day 1 administered at the same time as Fluzone HD Quadrivalent, and placebo on Day 29. Group 2 will receive placebo on Day 1 administered at the same time as Fluzone HD Quadrivalent, and Ad26/protein preF RSV vaccine on Day 29.

In all participant's solicited AEs are collected during the first 7 days after each vaccination, unsolicited AEs during the first 28 days after each vaccination and SAEs and AEs leading to discontinuation are captured during the whole study.

Blood will be collected from all participants to assess humoral immune responses pre-vaccination on each vaccination day and at 28 days after the second study vaccination.

Study design of VAC18193RSV3005

Group	N	Day 1	Day 29
1 (CoAd group)	375	Ad26/protein preF RSV vaccine + Fluzone HD Quadrivalent	Placebo
2 (Control group)	375	Placebo + Fluzone HD Quadrivalent	Ad26/protein preF RSV vaccine

N = number of participants.

VAC18193RSV3006 is a randomized, double-blind, placebo-controlled, multicenter, interventional Phase 3 study to evaluate the safety and immunogenicity of the Ad26/protein preF RSV vaccine in adults aged 18 to 59 years, including those who are at high risk of severe RSV disease. A cohort of adults aged 65 years and older will also be included. A target of 1113 participants will be enrolled: 385 healthy adults aged 18 to 59 years will be enrolled in Cohort 1 and randomized in a 9:2 ratio to receive Ad26/protein preF RSV vaccine or placebo; 385 high-risk adults aged 18 to 59 years will be enrolled in Cohort 2 and randomized in a 9:2 ratio to receive Ad26/protein preF RSV vaccine or placebo, 343 adults aged 65 years and older will be enrolled in Cohort 3 and randomized in a 45:4 ratio to receive Ad26/protein preF RSV vaccine or placebo. All cohorts will be enrolled in parallel. Participants will be stratified for COVID-19 vaccine administration (Janssen COVID-19 vaccine, AstraZeneca, other vaccine or no vaccine).

In all participants solicited AEs are collected during the first 7 days after each vaccination, unsolicited AEs during the first 28 days after each vaccination and SAEs and AEs leading to discontinuation are captured during the whole study.

Blood will be collected for all subjects to assess humoral immune responses pre-vaccination and at 14 days, 84 days and 6 months post-vaccination. In a subset of the immunosubset, PBMC samples will be collected to assess cellular immune response.

Study design of VAC18193RSV3006

Cohort		Group	N	Day 1 (Vaccination)
1	healthy adults,	1	315	Ad26.RSV.preF 1×10^{11} vp + RSV preF protein 150 µg
	18 to 59 years	2	70	Placebo
2	high-risk adults,	3	315	Ad26.RSV.preF 1×10^{11} vp + RSV preF protein 150 µg
	18 to 59 years [a]	4	70	Placebo
3	adults, 65 years and older	5	315	Ad26.RSV.preF 1×10^{11} vp + RSV preF protein 150 µg
		6	28	Placebo

N = number of participants, vp = virus particles.
In all cohorts, participants will be stratified for COVID-19 vaccine administration.

Independent Data Monitoring Committee (IDMC)

IDMC Charter covering safety review of phase 3 efficacy study (VAC18193RSV3001) and the supportive phase 3 studies
Version:27 July 2022 (Amendment 3)

Approved by:

PPD

04 August 2022

PPD
Cha[redacted]

Date (Day Month Year)

PPD
Member, Data Monitoring Committee

Date (Day Month Year)

PPD
Member, Data Monitoring Committee

Date (Day Month Year)

PPD
Chairperson, Sponsor Committee

Date (Day Month Year)

Independent Data Monitoring Committee (IDMC)

IDMC Charter covering safety review of phase 3 efficacy study (VAC18193RSV3001) and the supportive phase 3 studies

Version:27 July 2022 (Amendment 3)

Approved by:

PPD

Chairperson, Data Monitoring Committee

Date (Day Month Year)

PPD

PPD

Member, Data Monitoring Committee

Date (Day Month Year)

PPD

Member, Data Monitoring Committee

Date (Day Month Year)

PPD

Chairperson, Sponsor Committee

Date (Day Month Year)

Independent Data Monitoring Committee (IDMC)

IDMC Charter covering safety review of phase 3 efficacy study (VAC18193RSV3001) and the supportive phase 3 studies
Version:27 July 2022 (Amendment 3)

Approved by:

PPD
Chairperson, Data Monitoring Committee
Date (Day Month Year)

PPD
Member, Data Monitoring Committee
Date (Day Month Year)

PPD

27 JULY 2022
PPD
Member, Data Monitoring Committee
Date (Day Month Year)

PPD
Chairperson, Sponsor Committee
Date (Day Month Year)
