# Supplementary material for: A randomised, double-blind, multicentre, placebo-controlled phase 3 efficacy, safety, and immunogenicity study of an Ad26.RSV.preF/RSV prefusion F-protein vaccine for the prevention of respiratory syncytial virus-mediated lower respiratory tract disease in adults aged 60 years and older
Source: eBioMedicine. 2026 Nov 4;133:106513. doi: 10.1016/j.ebiom.2026.106513 (PMC13760371; doi:10.1016/j.ebiom.2026.106513)
Supplement: Supplementary file 4 [file mmc4.docx]

**S****u****pplementary Materials**

A randomised, double-blind, multicentre, placebo-controlled phase 3 efficacy, safety, and immunogenicity study of an Ad26.RSV.preF/RSV prefusion F-protein vaccine in the prevention of respiratory syncytial virus-mediated lower respiratory tract disease in adults aged 60 years and older

**Table of Contents**

Protocol Amendments	4

Institutional Review Boards and Ethics Committees (IRB/EC) by Country/Region and Site	4

The senior Investigator was Dr Hongxing Pan, China. The central ethics committee in China was the Ethics Committee of Jiangsu Provincial Center for Disease Control and Prevention, Nanjing (approval number: SL2021-A031-01).	4

Eligibility Criteria	19

Laboratory Analyses	21

Thrombotic events to be reported as potential adverse events of special interest^1^	22

Statistical analyses	24

Interim analysis plan	24

Statistical Analyses Amendments	25

Sensitivity Analyses	25

Data Protection and Quality Assurance	26

Independent Data Monitoring Committee (IDMC)	26

Exploratory efficacy objectives and endpoints	28

Supplementary Figure S1. Sensitivity analyses for first occurrence of RT-PCR–confirmed RSV-mediated LRTD.	30

Supplementary Figure S2. Exploratory endpoint. Total RiiQ™ symptom scores in participants with RT-PCR–confirmed RSV ARIs. (per protocol efficacy set).	31

Supplementary Figure S3. Exploratory endpoint (a) Time to return to usual health for participants with RT-PCR-confirmed RSV LRTD. (b) Time to return to usual health for participants with RT-PCR–confirmed RSV ARI (per protocol efficacy set; Primary analysis).	32

Supplementary Figure S4. Exploratory endpoint. RSV A viral load restricted to participants with an RSV A infection and RSV B viral load for participants with an RSV B infection a) first year, b) second year, c) whole study (per protocol efficacy set)	33

Supplementary Figure S5. Exploratory endpoint. Titres of neutralising antibodies to RSV B strain (Clinical Isolate 2017): plot of the actual values over time (per protocol immunogenicity set)	34

Supplementary Figure S6. Exploratory endpoint. Boxplots of area under the curve (AUC) of EQ-5D-5L visual analogue scale (VAS) for participants with RT-PCR-confirmed RSV-mediated ARI or LRTD (per protocol efficacy set)	35

Supplementary Figure S7. Immunogenicity of Ad26.RSV.preF/RSV preF protein at Days 1 and 15 in the immunogenicity subset: RSV A2 neutralising antibodies.	36

Supplementary Figure S8. Immunogenicity of Ad26.RSV.preF/RSV preF protein at Days 1 and 15 in the immunogenicity subset: RSV preF A IgG serum antibody response (ELISA).	37

Supplementary Figure S9. Immunogenicity of Ad26.RSV.preF/RSV preF protein at Days 1 and 15 in the immunogenicity subset: RSV preF B IgG serum antibody response (ELISA).	38

Supplementary Figure S10. Immunogenicity of Ad26.RSV.preF/RSV preF protein at Days 1 and 15 in the immunogenicity subset: RSV-F–specific T-cell response (IFN-γ ELISpot).	38

Supplementary Figure S11. Immunogenicity of Ad26.RSV.preF/RSV preF protein at Days 1 and 15 in the immunogenicity subset by age: RSV A2 neutralising antibodies.	39

Supplementary Figure S12. Immunogenicity of Ad26.RSV.preF/RSV preF protein at Days 1 and 15 in the immunogenicity subset by age: RSV preF A IgG serum antibodies (ELISA).	39

Supplementary Figure S13. Immunogenicity of Ad26.RSV.preF/RSV preF protein at Days 1 and 15 in the immunogenicity subset by age: RSV-F–specific T-cell response (IFN-γ ELISpot).	40

Supplementary Figure S14. Immunogenicity of Ad26.RSV.preF/RSV preF protein at Days 1 and 15 in the immunogenicity subset by risk level: RSV A2 neutralising antibodies.	40

Supplementary Figure S15. Immunogenicity of Ad26.RSV.preF/RSV preF protein at Days 1 and 15 in the immunogenicity subset by risk level: RSV preF A IgG serum antibodies (ELISA).	41

Supplementary Figure S16. Immunogenicity of Ad26.RSV.preF/RSV preF protein at Days 1 and 15 in the immunogenicity subset by risk level: RSV-F–specific T-cell response (IFN-γ ELISpot).	41

Supplementary Figure S17. Any solicited local adverse events 28 days postvaccination (safety subset, final analysis).	42

Supplementary Figure S18. Systemic adverse events 28 days postvaccination (safety subset, final analysis).	42

Supplementary Table S1. Baseline characteristics of the trial participants in the Full analysis set (final analysis)	43

Supplementary Table S2. Secondary confirmatory endpoints: percentage of participants per group meeting the secondary endpoints tested at the end of study and vaccine efficacy of their first occurrence (per protocol efficacy set)	44

Supplementary Table S3. Exploratory efficacy endpoints: percentage of participants per group meeting the exploratory endpoints related to any confirmed RSV ARI and vaccine efficacy of their first occurrence (per protocol efficacy set, Final analysis)	45

Supplementary Table S4. Exploratory endpoint: percentage of participants per group meeting the exploratory endpoints related to any confirmed RSV A or B ARI and vaccine efficacy of their first occurrence (per protocol efficacy set, Final analysis)	46

Supplementary Table S5. Exploratory endpoint Clinical Evaluation Committee assessment of severity (Primary analysis)	47

Supplementary Table S6. Exploratory endpoint. Clinically relevant diseases: percentage of participants per group with clinically relevant diseases; per protocol efficacy set	48

Supplementary Table S7. Exploratory endpoint. Titers of neutralising antibodies against the Ad26 vector (Per Protocol Immunogenicity Set)	49

Supplementary Table S8. Baseline characteristics of participants in the per protocol immunogenicity subset (Primary analysis)	49

Supplementary Table S9. Baseline characteristics of participants in the safety subset	50

Supplementary Table S10. Participants with at least one grade 3 or higher unsolicited adverse event until 28 days after vaccination (safety subset, final analysis)	51

Supplementary Table S11. Participants with at least one AE leading to death during the whole study by System Organ Class (FAS, final analysis)	52

Supplementary Table S12. Concomitant medications (major drug classes) reported after vaccination and by at least 0.5% of participants in either group (FAS-Safety subset, Final analysis)	53

References	55

# Protocol Amendments

There were five amendments to the study protocol. In the first amendment (3 May 2021), several changes were made to follow recommendations from regulatory authorities (eg, update of the primary hypothesis of vaccine efficacy [VE] against reverse transcription polymerase chain reaction [RT-PCR]–confirmed respiratory syncytial virus [RSV] mediated lower respiratory tract disease [LRTD], stratification, and minimisation factors for the randomisation, secondary endpoints testing strategy). In the second amendment (19 July 2021), revisions were made to align the protocol with information and guidance for investigators on signs and symptoms and on medical management of thrombosis with thrombocytopenia syndrome events across the investigational RSV vaccine programme. In amendment three (10 November 2021), the protocol was amended to state that all cases meeting the programmed case definition of RT-PCR–confirmed RSV LRTD should be reviewed by the clinical evaluation committee. Additionally, to increase the probability of having a primary analysis at the end of the first RSV season, an interim analysis by the independent data monitoring committee was added if the number of observed RSV cases was ≥24 to <62. If the interim analysis by the independent data monitoring committee has positive results, then the study will be unblinded and the primary analysis will be performed by the sponsor. In the fourth amendment (8 April 2022), the second vaccine dose at Month 12 was removed from the protocol and any references to revaccination were also removed. Furthermore, the interim analysis plan specified in the third amendment was also modified, such that interim analysis was no longer dependent on the number of observed cases at the end of the first RSV season. Rather, interim analysis was based on a database cut-off of 15 September 2022. Amendment five (11 October 2022) was enacted to provide clarification around the timing of the end-of-study analyses and population included, align risk language with available safety data, and provide additional clarification around follow-up conducted via phone call versus onsite visit.

# Institutional Review Boards and Ethics Committees (IRB/EC) by Country/Region and Site

# The senior Investigator was Dr Hongxing Pan, China. The central ethics committee in China was the Ethics Committee of Jiangsu Provincial Center for Disease Control and Prevention, Nanjing (approval number: SL2021-A031-01).

| **IRB/EC Name/Address** | **Site** |
| --- | --- |
| **Australia** |  |
| Bellberry Human Research Ethics Committees. South Australia, 5065, Australia | Paratus Clinical Research, 654 Pacific Highway, Suite A8, Kanwal Medical Complex Kanwal, NSW, 259, Australia |
|  | Northside Health, 14 Edgar Street, Coffs Harbour, NSW, 2450. Australia |
|  | Paratus Clinical Research, Cnr Allawoona Street & Ginninderra Drive, Canberra. ACT. 2617 Australia |
|  | Mater Hospital, 32 Raymond Terrace Brisbane, Qld, 4101 Australia |
| **Brazil** |  |
| CEP do Hospital Universitario - CAS/UFJF/MG Rua Catulo Breviglieri, S/N Juiz de For a, Minas Gerais 36036-110, Brazil | CMiP - Centro Mineiro de Pesquisa Rua Delfim Moreira, 186, Centro Juiz de Fora, Minas Gerais, 36010-570, Brazil |
| CONEP - Comissão Nacional de Ética em Pesquisa, SRNTV 701, Via W 5 Norte Edifício PO 700, 3º andar- Asa Norte. Distrito Federal, Brasília. 70719-049 . Brazil | CMiP - Centro Mineiro de Pesquisa Rua Delfim Moreira, 186, Centro Juiz de Fora, Minas Gerais, 36010-570, Brazil |
| CEP da Universidade Federal de Roraima Avenida Capitão Ene Garcez, 2413 - Campus Paricarana-Aeroporto, Boa Vista, Roraima, 69304-000 Brazil | UFRR - Universidade Federal de Roraima, Avenida Capitão Ene Garcez, 2413 - Campus Paricarana, Aeroporto, Boa Vista, Roraima, 69304-000 Brazil. |
| CONEP - Comissão Nacional de Ética em Pesquisa, SRNTV 701, Via W 5 Norte Edifício PO 700, 3º andar- Asa Norte. Distrito Federal, Brasília. 70719-049 . Brazil | UFRR - Universidade Federal de Roraima, Avenida Capitão Ene Garcez, 2413 - Campus Paricarana, Aeroporto, Boa Vista, Roraima 69304-000, Brazil |
| CEP da Faculdade de Medicina de São José do Rio Preto – FAMERP Av. Brigadeiro Faria Lima, 5416 São José do Rio Preto, Sao Paulo 15090-000, Brazil | Fundação Faculdade Regional de Medicina de São José do Rio Preto Av. Brigadeiro Faria Lima, 5544 - 2º andar, Sao Jose Rio Preto Sao Paulo, 15090-000, Brazil |
| CONEP - Comissão Nacional de Ética em Pesquisa, SRNTV 701, Via W 5 Norte, Edifício PO 700, 3º andar- Asa Norte. Distrito Federal, Brasília. 70719-049 . Brazil | Fundação Faculdade Regional de Medicina de São José do Rio Preto Av. Brigadeiro Faria Lima, 5544 - 2º andar, Sao Jose Rio Preto Sao Paulo, 15090-000 Brazil |
| CEP do Hospital de Clínicas de Porto Alegre/HCPA, Rua Ramiro Barcelos, 2350 - 2º andar - Sala 2227, Porto Alegre, Rio Grande do Sul 90035-007, Brazil | Hospital de Clínicas de Porto Alegre Rua Ramiro Barcelos, 2350, Build 21; Office 21308-B, Porto Alegre, Rio Grande do Sul 90035-903, Brazil |
| CONEP - Comissão Nacional de Ética em Pesquisa, SRNTV 701, Via W 5 Norte, Edifício PO 700, 3º andar- Asa Norte. Distrito Federal, Brasília. 70719-049 . Brazil | Hospital de Clínicas de Porto Alegre Rua Ramiro Barcelos, 2350, Build 21; Office 21308-B, Porto Alegre, Rio Grande do Sul 90035-903, Brazil |
| CEP do Instituto de Saude e Bem Estar da Mulher - ISBEM / SP, Avenida Indianópolis, 2700, Planalto Paulista, São Paulo, São Paulo, 04062- 003, Brazil | CPQuali Pesquisa Clínica Ltda. Avenida Angélica, 916 - Conjunto 506, Santa Cecília, São Paulo, 01228-000 Brazil |
| CONEP - Comissão Nacional de Ética em Pesquisa, SRNTV 701, Via W 5 Norte, Edifício PO 700, 3º andar- Asa Norte. Distrito Federal, Brasília. 70719-049 . Brazil | CPQuali Pesquisa Clínica Ltda. Avenida Angélica, 916 - Conjunto 506, Santa Cecília, São Paulo, 01228-000 Brazil |
| **Canada** |  |
| WCG IRB, 1019 39th Avenue SE Suite 120 Puyallup, Washington 98374 Canada | Centricity Research Levis - DBA LMC Manna Research Quebec 1190 A, Rue De Courchevel, Local 203, Levis |
|  | Centricity Research Pointe-Claire - DBA LMC Manna Research Montreal, 175 Stillview Avenue, Suite 145, Pointe-Claire, Quebec, H9R 4S3 Canada |
|  | Centricity Research Toronto North West - DBA LMC Manna Research Toronto, 2291 Kipling Avenue Suite 117B, Toronto, Ontario, M9W 4L6 Canada |
|  | Centricity Research Burlington - DBA LMC Manna Research Burlington, 2951 Walkers Line, 2nd floor Burlington, L7M 4Y1, Ontario, Canada |
|  | Centricity Research Toronto Uptown - DBA LMC Manna Research Bayview, 1929 Bayview Avenue Suite 125 Toronto, Ontario, M4G 3E8 Canada |
|  | DIEX Recherche Victoriaville Inc. 39, rue Laurier Est, Suite 4 Victoriaville, Quebec, G6P 6P6, Canada |
|  | DIEX Recherche Quebec Inc. 2600 Boul. Laurier, Suite 293,, Quebec, Quebec, G1V 4T3 Canada |
|  | DIEX Recherche Sherbrooke Inc. 15 J.A. Bombardier, Suite A-160 Sherbrooke, Quebec, J1L 0H8 Canada |
|  | OCT Research ULC 204-1353 Ellis Street, Kelowna, British Columbia V1Y 1Z9, Canada |
|  | DIEX Recherche Joliette Inc. 28 Chemin du Golf Est, Suite 102-104, Saint-Charles-Borromée Quebec, J6E 2B4 Canada |
| **Chile** |  |
| Comité Ético-Científico Servicio de Salud Metropolitano Sur Oriente Av. Concha y Toro 3459 Santiago, 8207257, Chile | CENRESIN, San Martin 502, Quilota, 2260734 Chile |
| Comité de Ética de Investigación en Seres Humanos Independencia 1027, Santiago, 8380453 Chile. | CeCim Biocinetic, Paseo Bulnes 79, Oficina 27 Santiago, Region Met, 8331143, Chile |
| Comité Ético-Científico Servicio de Salud Metropolitano Sur Oriente, Av. Concha y Toro 3459 Santiago, 8207257, Chile | Centro de Investigacion del Maule 9 ½ Oriente 1457, Edificio médico del Maule Talca, 3465586 |
| **China** |  |
| EC of Jiangsu Provincial Center for Disease Control and Prevention, No.172 Jiangsu Road, Gulou District Nanjing, 210009 | No.23 Gangcheng Road, Binhai County, Yancheng City Jiangsu, 224554, China |
|  | Funing County Center for Disease Control and Prevention, No. 551, Hong Kong Road, Chengnan New District, Funing County, Yancheng, Jiangsu, 224400 China |
|  | Donghai County Center for Disease prevention and Control Office Building of Center for Disease Control and Prevention, North Side of Jingdu Avenue, Donghai County Lianyungang, Jiangsu, 222300, China |
| **Estonia** |  |
| Estonia EU CTIS Part II - Ethics Committee for Medicinal Products, Nooruse 1 Street, Tartu, 50411 | Clinical Research Centre Sõbra 54, Tartu, 50106 Estonia |
| Research Ethics Committee of the National Institute for Health Development, Hiiu 42, Tallinn, 11619 | Clinical Research Centre Sõbra 54, Tartu, 50106 Estonia |
| Estonia EU CTIS Part II - Ethics Committee for Medicinal Products, Nooruse 1 Street, Tartu, 50411 | OÜ Innomedica Narva mnt 7, Tallinn, 10117 Estonia |
| Research Ethics Committee of the National Institute for Health Development, Hiiu 42, Tallinn, 11619 | OÜ Innomedica Narva mnt 7, Tallinn, 10117 Estonia |
| Estonia EU CTIS Part II - Ethics Committee for Medicinal Products, Nooruse 1 Street, Tartu, 50411 | Center for Clinical and Basic Research, J.Pärna 4, Tallinn, 10128 Estonia |
| Research Ethics Committee of the National Institute for Health Development, Hiiu 42, Tallinn, 11619 | Center for Clinical and Basic Research, J.Pärna 4, Tallinn, 10128 Estonia. |
| **Finland** |  |
| TUKIJA, Ratapihantie 9, 00520, Helsinki, PL 43, 00521 Finland | Järvenpään rokotetutkimusklinikka Mannilantie 44, 2krs Järvenpää, 04400, Finland |
|  | Turun rokotetutkimusklinikka Lemminkäisenkatu 14-18 B, 4. Krs, Turku, 20520 Finland |
|  | Etelä-Helsingin rokotetutkimusklinikka Vuorikatu 18, 3. Krs Helsinki, 00100 Finland |
|  | Oulun rokotetutkimusklinikka Kiviharjunlenkki 6, Oulu, 90220 Finland |
|  | Seinäjoen rokotetutkimusklinikka Kauppatori 1-3, 2. krs,, Kauppakeskus Torikeskus, Seinäjoki, 60100 Finland |
|  | Kokkolan rokotetutkimusklinikka Rantakatu 16, 6th floor, Kokkola, 67100 Finland |
|  | Porin rokotetutkimusklinikka Yrjönkatu 23, 4. Krs, Pori, 28100 Finland |
|  | Tampereen rokotetutkimusklinikka Pinninkatu 47, 1 floor, Tampere, 33100 Finland |
|  | Itä-Helsingin rokotetutkimusklinikka Itäkeskuksen kauppakeskus, Agenttitalo, Turunlinnantie 8, Helsinki,00930 |
|  | Espoon rokotetutkimusklinikka Piispansilta 11, Ison Omenan toimistotorni, 7. krs Espoo, 02230, Finland. |
| **New Zealand** |  |
| Central Health and Disability Ethics Committee 20 Aitken Street, Ministry of Health, Ethics Department, Reception - Ground Floor, Thorndon, Wellington, 6011 New Zealand | Southern Clinical Trials Christchurch Forte 2, Level 2, 132 Peterborough Street, Christchurch 8013, New Zealand |
|  | Optimal Clinical Trials, Level Two, 97 Grafton Road, Grafton Auckland, 1010, New Zealand |
|  | Southern Clinical Trials Waitemata Level 1, 29 Birkenhead Avenue, Birkenhead, Auckland, 626 |
|  | Lakeland Clinical Trials 1289 Haupapa Street Rotorua, 3010, New Zealand |
|  | Southern Clinical Trials Tasman 469 Main Road Stoke, Main Road Stoke 7011, New Zealand |
|  | Southern Clinical Trials Totara Level 1, 1 McCrae Way, New Lynn Auckland 0600, New Zealand |
|  | P3 Research Limited (Kapiti) 44A Ihakara Street, Paraparaumu, 5032, New Zealand |
|  | Lakeland Clinical Trials Waikato 6 Avalon Drive, Nawton, Hamilton 3243 New Zealand |
|  | Lakeland Clinical Trials Wellington Upper Hutt Health Centre, Queen Street,, Ebdentown, 5018 New Zealand |
|  | P3 Research Limited (Hawkes Bay) The Village Health Centre, 33 Napier Road, Havelock North, 3410 New Zealand |
|  | Central, Suite 11, Promed House 71 Tenth Avenue, Tauranga, 3110 New Zealand |
|  | P3 Research Limited (Wellington) 121 Adelaide Road, Mt Cook Level 1, Wellington, 6021, New Zealand |
|  | P3 Research Limited (Palmerston North), 273 Broadway Avenue, Palmerston North, 4414 New Zealand |
| **Poland** |  |
| Komisja Bioetyczna przy DIL ul. Kazimierza Wielkiego 45 Wroclaw, 50-077, Poland | Synexus Polska Sp. z o.o. Oddział w Warszawie, ul. Leszno 12, Warszawa, 01-192 Poland |
|  | Synexus Polska SCM Sp. z o.o. Gdansku, Beniowskiego 23, Gdansk, 80-382 Poland |
|  | Synexus Polska Sp. z o.o. Oddzial w Czestochowie, Wały Ge, nerała Józefa Dwernickiego 43/45 Czestochowa, 42-202, Poland |
|  | Synexus Polska sp. z o.o. Oddzial w Poznaniu, ul. Glogowska 31/33,Poznan, 60-702 Poland |
|  | Synexus Polska Sp. z o.o. Oddział w Lodzi, ul. Skladowa 35, Lodz, 90-127, Poland |
|  | Synexus Polska Sp. z o.o. Oddzial w Katowicach, ul. Konckiego 3, Katowice, 40-040 Poland |
|  | Synexus Polska Sp. z o.o. Oddział we Wroclawiu, ul. Marii Curie-Skłodowskiej 12 Wrocław, 50-381, Poland |
|  | KO-MED Centra Kliniczne Zamosc ul. Peowiaków 1, Zamosc, 22-400 Poland |
|  | KO-MED Centra Kliniczne Staszow ul. 11 Listopada 78, Staszow, 28-200 Poland |
|  | KO-MED Centra Kliniczne Puławy Wacława, Sieroszewskiego 34, Puławy, 24-100 |
|  | KO-MED Centra Kliniczne Lublin II ul. Przerwy - Tetmajera 21, Lublin, 20-362 Poland |
|  | Synexus Polska Sp. z o.o. Oddzial w Gdyni, ul. Luzycka 3c Gdynia, 81-537 Poland |
|  | Centrum Medyczne Pratia Poznan ul. Poznańska 14, Skórzewo, 60-185 Poland |
| **Thailand** |  |
| Central Research Ethics Committee (CREC) National Research Council of Thailand, 196 Phahonyothin Rd, Lat Yao, Chatuchak Bangkok, 10900, Thailand | Srinagarind Hospital 123 Mittraparb Road, Muang, Khon Kaen, 40002 Thailand |
| The Khon Kaen University Ethics Committee for Human Research, 17th Floor, Room no. 1733, Somdetprasrinakarintra Building, Faculty of Medicine, Khon Kaen University, Nai Muang Sub-district, Muang, Khon Kaen, 40002 Thailand | Srinagarind Hospital 123 Mittraparb Road, Muang, Khon Kaen, 40002 Thailand |
| Central Research Ethics Committee (CREC) National Research Council of Thailand, 196 Phahonyothin Rd, Lat Yao, Chatuchak Bangkok, 10900, Thailand | King Chulalongkorn Memorial Hospital, 1873 Rama IV Road Pathumwan, Bangkok 10330, Thailand |
| The Institutional Review Board, Faculty of Medicine, Chulalongkorn University, 3Rd Floor Ananthamahidol Building, Faculty of Medicine, Bangkok, 10330 Thailand | King Chulalongkorn Memorial Hospital, 1873 Rama IV Road Pathumwan, Bangkok 10330, Thailand |
| Ethics Committee of the Faculty of Tropical Medicine, Mahidol University, 420/6 Ratchawithi Road., Ratchathewi, 10400 Thailand | Vaccine Trial Centre, Faculty of Tropical Medicine, Mahidol University, 420/6 Ratchawithi Road, Ratchathewi, Bangkok, 10400 Thailand |
| Institutional Review Board, Royal Thai Army Medical Department, 5th Floor Phramongkutklaowejwithaya Building, Phramongkutklao College of Medicine, 315 Rajavithi Road, Thung Phaya Thai Ratchathewi, Bangkok, 10700, Thailand | Armed Forces Research Institute of Medical Sciences (AFRIMS), 315/6 Rajvithi Road Rajathevi, Bangkok, 10400 Thailand |
| Research Ethics Committee, Faculty of Medicine, Chiangmai University, Thailand, 110 Intavaroros Road Muang, 50200, Thailand | Maharaj Nakorn Chiang Mai Hospital. 110 Intavaroros Road, Tambon Sritum, Muang, Chiang Mai, 50200 Thailand |
| Siriraj Institutional Review Board (SiRB) Research Ethics Committee, Faculty of Medicine, Chiangmai University  110 Intavaroros Road, Faculty of Medicine Chiangmai University Muang, Chiang Mai, 50200, Thailand | Siriraj Hospital, Mahidol University 2 Prannok Road Bangkok-noi, Bangkok, 10700 Thailand |
| **Taiwan, Republic of China** |  |
| Tri-Service General Hospital, Institutional Review Board  No.325, Sec. 2, Cheng-Kung Rd., Neihu Taipei, 11490  Taiwan, Republic of China | Tri-Service General Hospital, 325, Sec 2, Cheng-Kung Road, Neihu District, Taipei, 11490, Taiwan, Republic of China |
| National Taiwan University Hospital, Research Ethics Committee, Rm. 521, 5F, No. 33, Linsen S. Rd. Zhongzheng Dist, Taipei 100025, Taiwan, Republic of China | National Taiwan University Hospital, 7, Chung-Shan South Road, Taipei, 100, Taiwan, Republic of China |
| Taipei Veterans General Hospital, Institutional Review Board, 201, Sec. 2, Shih-Pai Rd, Taipei, 11217, Taiwan, Republic of China | Taipei Veterans General Hospital No. 201 Sec. 2 Shih-Pai Rd., Taipei, 11217, Taiwan, Republic of China |
| Taipei Medical University, Joint Institutional Review Board, 17F., No. 172-1, Sec. 2, Keelung Rd, Da’an Dist. Taipei City, 106339 Taiwan, Republic of China | Taipei Medical University Hospital No. 252, Wuxing St, Xinyi District, Taipei City, 110, Taiwan, Republic of China |
| China Medical University Hospital, Institutional Review Board, 6th Meeting Room, B1, 1st Medical Buidling No.2 Yuh-Der Road, Taichung, 40447, Taiwan, Republic of China | China Medical University Hospital No. 91, Xueshi Rd., North Dist., LiFu Building 5 F-Clinical Trial Center, Taichung, 40447 Taiwan, Republic of China |
| Chang Gung Medical Foundation, Institutional Review Board, No. 123, Dinghu Rd., Jioulu Village, Gueishan Township, B2, Taoyuan, 333, Taiwan, Republic of China | Chang Gung Memorial Hospital,Linkou, No. 5, Fusing St., Gueishan Township, Taoyuan County, 333 Taiwan, Republic of China |
| Taipei Medical University, Joint Institutional Review Board, 17F., No. 172-1, Sec. 2, Keelung Rd, Da’an Dist. Taipei City, 106339 Taiwan, Republic of China | Taipei Municipal Wanfang Hospital Managed by Taipei Medical University, No.111, Section 3, Hsing-Long Rd., Wenshan Dist, Taipei, 116, Taiwan, Republic of China |
| Kaohsiung Medical University Chung-Ho Memorial Hospital, Institutional Review Board, No.100 Tzyou 1st Road 6F, S Building Kaohsiung, 807, Taiwan, Republic of China | Kaohsiung Medical University Chung-Ho Memorial Hospital No.100, Tzyou 1st Road Kaohsiung, 807  Taiwan, Republic of China |
| **South Africa** |  |
| Pharma Ethics 123 Amcor Road  Lyttelton Manor, Pretoria, Gauteng, 0157 | Tread Research, Room 41, 8th Floor Green Avenue, Francie van Zijl Drive, Parow South Africa |
|  | Josha Research Rubins Building, 28 East Burger Street, Bloemfontein, Free State, 9301, South Africa |
|  | Newtown Clinical Research, Suite 3, Newgate Centre, First Floor, Johannesburg,, Gauteng, 2001 South Africa |
|  | Synexus SA Watermeyer Clinical Research Centre, 60 Stamvrug Street, Val de Grace, Ground Floor Synexus Building Pretoria, Gauteng, 0184, South Africa |
|  | Tiervlei Trial Centre, c/o Mike Pienaar & Frans Conradie Avenue, Karl Bremer Hospital, Basement Level, Bellville, Cape Town, Western Cape, Cape Town, 7530, South Africa |
|  | TASK Central, 1 De Lange street, Western Cape Cape Town 7530, South Africa |
|  | Synexus Helderberg Clinical Research Centre, Suite 7G and H Arun Place, Sir Lowrys Pass Road, Somerset West Cape Town, Western Cape, 7130 South Africa |
|  | Synexus SA - Stanza Clinical Research Centre, 2 Shilovhane Street, Mamelodi East, Gauteng, 0122 South Africa |
|  | DJW Research, 70-72 Shannon Street, Noordheuwel, Krugersdorp, Gauteng, 1739 |
|  | Allergy & Immunology Unit University of Cape Town Lung Institute, George Street, Mowbray, Cape Town Western Cape, 7700, South Africa |
| University of Cape Town HREC Faculty of Health Sciences Research EC E52-24 Old Main Building  Groote Schuur Hospital, Observatory | Allergy & Immunology Unit University of Cape Town Lung Institute, George Street, Mowbray, Cape Town Western Cape, 7700, South Africa |
| Pharma Ethics 123 Amcor Road  Lyttelton Manor, Pretoria, Gauteng, 0157 | Be Part Yoluntu Centre, 4 Madikane Street, Mbekweni Paarl, Western, Western Cape 7626 Western Cape South Africa |
|  | Madibeng Centre for Research (MCR), 40 Pienaar Street, Brits, North West, 0250, South Africa |
|  | Paarl Research Centre 12 Verster Street, Paarl Western Cape, 7646 South Africa |
|  | The Aurum Institute Rustenburg Clinical Research Centre  Heritage Building 178 Beyers Naude Drive, Rustenburg North West 0299 South Africa |
|  | Iatros International, 20 Captain Proctor Street Bloemfontein, Free State, 9301 South Africa |
|  | Worthwhile Clinical Trials 1 Mowbray Avenue, Benoni, Johannesburg, Gauteng, 1500 South Africa |
|  | REIMED Vosloorus Ext 24198 Zakhe Street  Extension 2, Vosloorus, Vosloorus South Africa |
|  | CRISMO Research Centre, Villa Heidi Nurses Home Building Ground floor, Cnr Joubert and Hospital St, Germiston, Johannesburg South Africa |
|  | TASK Eden, 4 Victoria Street, George Central, George Central, Western Cape, 6530 South Africa |
|  | Global Clinical Trials Pretoria Shop 29 Nedbank Plaza, 175 Steve Biko Street, Arcadia Pretoria Gauteng, 0001, South Africa |
|  | Clinresco Centres (Pty) Ltd, Central Professional Suites, 1st floor, 20 Central Avenue, Kempton Park, Gauteng 1619, South Africa |
|  | REIMED Welkom, Shop 43 Checkers Centre Ararat Street, Doorn, Welkom Free State, 9460, South Africa |
|  | Synergy Biomed Research Institute (SBRI), Suite 3 Tudor Court, 280 Oxford Road, East London East London5241, South Africa |
| **United Kingdom** |  |
| NRES Committee East Midlands-Nottingham 2 The Old Chapel, Royal Standard Place Nottingham, Nottinghamshire, NG1 6FS United Kingdom | Nowgen Centre 29 Grafton Street, Manchester University NHS Foundation Trust, Manchester, Greater Manchester, M13 9WU, United Kingdom |
|  | Synexus North Tees Clinical Research Centre United Hardwick Road, Ground Floor, Middlefield Centre, University Hospital of North Tees Stockton on Tees, Durham, TS19 8PE United Kingdom |
|  | Synexus Hexham Clinical Research Centre, Corbridge Road, Hexham, Northumberland NE46 1QJ, United Kingdom |
| Synexus - Amendments Sandringham House-Ackhurst Park Chorley, Lancashire, PR7 1NY United Kingdom | Synexus Hexham Clinical Research Centre-Corbridge Road, Hexham, Northumberland, NE46 1QJ United Kingdom |
| NRES Committee East Midlands-Nottingham 2 The Old Chapel Royal Standard Place Nottingham, Nottinghamshire, NG1 6FS United Kingdom | Synexus Birmingham Clinical Research Centre, Vincent Drive, Edgbaston Birmingham, West Midlands B15 2SQ, United Kingdom |
| Synexus - Amendments Sandringham House-Ackhurst Park Chorley, Lancashire, PR7 1NY United Kingdom | Synexus Birmingham Clinical Research Centre-Vincent Drive Birmingham, West Midlands, B15 2SQ, United Kingdom |
| NRES Committee East Midlands-Nottingham 2 The Old Chapel, Royal Standard Place Nottingham Nottinghamshire, NG1 6FS United Kingdom | Synexus Merseyside Clinical Research Centre, Burlington House, 3rd Floor, South Wing Crosby Road North Liverpool, Merseyside L22 0LG, United Kingdom |
| Synexus - Amendments Sandringham House-Ackhurst Park Chorley, Lancashire, PR7 1NY United Kingdom | Synexus Merseyside Clinical Research Centre-Burlington House Liverpool, Merseyside, L22 0LG United Kingdom |
| NRES Committee East Midlands-Nottingham 2 The Old Chapel, Royal Standard Place Nottingham Nottinghamshire, NG1 6FS United Kingdom | Synexus Scotland Clinical Research Centre, Synexus Scotland Dedicated Research Centre, Pheonix Cres, Belshill Glasgow, North Lanarkshire ML4 3NJ, United Kingdom |
| Synexus - Amendments Sandringham House-Ackhurst Park Chorley, Lancashire, PR7 1NY United Kingdom | Synexus Scotland Clinical Research Centre-Synexus Scotland Dedicated Research Centre, Glasgow, North Lanarkshire, ML4 3NJ, United Kingdom |
| NRES Committee East Midlands-Nottingham 2 The Old Chapel, Royal Standard Place Nottingham Nottinghamshire, NG1 6FS United Kingdom | Synexus Wales Clinical Research Centre-2 Riverside Court, Cardiff, South Glamorgan, CF15 9SS United Kingdom |
| NRES Committee East Midlands-Nottingham 2 The Old Chapel, Royal Standard Place Nottingham Nottinghamshire, NG1 6FS United Kingdom | Synexus Lancashire Clinical Research Centre, 24 Eaton Avenue, Matrix Park Buckshaw Village, Chorley Lancashire, PR7 7NA, United Kingdom |
| Synexus - Amendments Sandringham House-Ackhurst Park Chorley, Lancashire, PR7 1NY United Kingdom | Synexus Lancashire Clinical Research Centre-24 Eaton Avenue, Chorley, Lancashire, PR7 7NA United Kingdom |
| NRES Committee East Midlands-Nottingham 2 The Old Chapel, Royal Standard Place Nottingham Nottinghamshire, NG1 6FS United Kingdom | Synexus Manchester Clinical Research Centre, Lloyd Street North Manchester, Greater Manchester, M15 6SX United Kingdom |
| Synexus - Amendments Sandringham House-Ackhurst Park Chorley, Lancashire, PR7 1NY United Kingdom | Synexus Manchester Clinical Research Centre-Lloyd Street North Manchester, Greater Manchester, M15 6SX, United Kingdom |
| NRES Committee East Midlands-Nottingham 2 The Old Chapel, Royal Standard Place Nottingham Nottinghamshire, NG1 6FS United Kingdom | FutureMeds Liverpool 45 Bridge Road Eastham Bromborough, Merseyside, CH62 6EE |
| FutureMeds - Amendments 213 Derbyshire Lane-  Sheffield, South Yorkshire, S8 8SA United Kingdom | FutureMeds Liverpool 45 Bridge Road Eastham Bromborough, Merseyside, CH62 6EE |
| NRES Committee East Midlands-Nottingham 2 The Old Chapel, Royal Standard Place Nottingham Nottinghamshire, NG1 6FS United Kingdom | Panthera Biopartners (Glasgow) Queen Elizabeth University Hospital 3rd Floor ICE Building, Glasgow, Strathclyde G51 4TF, United Kingdom |
| Panthera Biopartners – Amendments, 73 St Gregory Road-ISSA Medical Centre Preston, Lancashire, PR1 6YA, United Kingdom | Panthera Biopartners (Glasgow) Queen Elizabeth University Hospital 3rd Floor ICE Building, Glasgow, Strathclyde G51 4TF, United Kingdom |
| NRES Committee East Midlands-Nottingham 2 The Old Chapel, Royal Standard Place Nottingham Nottinghamshire, NG1 6FS United Kingdom | Bradford Royal Infirmary Dept of Respiratory Medicine Gate 4, Duckworth Lane, Bradford Teaching Hospitals NHS Foundation Trust, Bradford, West Yorkshire BD9 6RJ, United Kingdom |
| R&D - Bradford Teaching Hospitals NHS Foundation Trust, Bradford Royal Infirmary-Department of RespiratoryMedicine, Bradfod, West Yorkshire, BD9 6RJ United Kingdom | Bradford Royal Infirmary Dept of Respiratory Medicine Gate 4, Duckworth Lane, Bradford Teaching Hospitals NHS Foundation Trust, Bradford, West Yorkshire BD9 6RJ, United Kingdom |
| NRES Committee East Midlands-Nottingham 2 The Old Chapel, Royal Standard Place Nottingham, Nottinghamshire, NG1 6FS United Kingdom | Panthera Biopartners Ltd (Preston) The Fatima Health Centre, 228 Garstang Road Fulwood, Preston, PR2 9RB United Kingdom |
| Panthera Biopartners – Amendments, 73 St Gregory Road-ISSA Medical Centre Preston, Lancashire, PR1 6YA  United Kingdom | Panthera Biopartners Ltd (North London)  1 Woodall Road Panthera Biopartners Ground Floor Enfield, Greater London EN3 4GS, United Kingdom |
| NRES Committee East Midlands-Nottingham 2 The Old Chapel, Royal Standard Place Nottingham Nottinghamshire, NG1 6FS United Kingdom | Panthera Biopartners Ltd (Preston)- The Fatima Health Centre, Preston, Lancashire, PR2 9RB United Kingdom |
| Panthera Biopartners – Amendments, 73 St Gregory Road-ISSA Medical Centre Preston, Lancashire, PR1 6YA  United Kingdom | Panthera Biopartners Ltd (Preston)- The Fatima Health Centre, Preston, Lancashire, PR2 9RB United Kingdom |
| NRES Committee East Midlands-Nottingham, Nottingham Nottinghamshire, NG1 6FS United Kingdom | FutureMeds Birmingham 247-251 Soho Road Birmingham, West Midlands B21 9RY, United Kingdom |
| **United States** |  |
| WCG IRB, 1019 39th Avenue SE Suite 120, Puyallup, Washington, 98374 United states | MedPharmics, LLC, 3800 Houma Blvd, Metairie Louisiana, 70006, United States |
|  | Paradigm Clinical Research Centers, Inc. 3652 Eureka Way, Redding, California, 96001 United states |
|  | Velocity Clinical Research, Cleveland, 3619 Park East Drive, Suite 300 Beachwood, Cleveland, Ohio, 44122 United States |
|  | Meridian Clinical Research LLC 415 Hooper Road, Endwell, New York 13760, United states |
|  | ARS - DeLand Clinical Research Unit, 860 Peachwood Drive, Deland 32720, United States |
|  | Velocity Clinical Research, Boise 2950 E. Magic View Drive, Suite 182, Boise, Idaho, 83642 United States |
|  | Meridian Clinical Research, LLC 3400 West Norfolk Ave, Norfolk, Nebraska, 68701 United States |
|  | MedPharmics – Biloxi, 15444 Dedeaux Road, Suite B Gulfport, Mississippi, 39503, United States |
|  | Medication Management, LLC 2006 North Church Street, Greensboro, North Carolina, 27405 United States |
|  | Jacksonville Center for Clinical Research, 4085 University Boulevard South Suite 1, Jacksonville, Florida 32216, United States |
|  | HOPE Research Institute 2525 W. Greenway Road Suite 240, Phoenix, Arizona, 85023 United States |
|  | Velocity Clinical Research, Austin 1900 Cypress Creek Road, Suite 100, Cedar Park Texas, 78613, United States |
|  | Meridian Clinical Research, LLC 340 Eisenhower Drive, Bldg. 1500, Savannah, Georgia, 31406 United States |
|  | Harmony Clinical Research, Inc. 18260 NE 19th Avenue, Suite 103, North Miami Beach Florida, 33162, United States |
|  | Westside Center for Clinical Research, 810 Lane Avenue, South Jacksonville, Florida, 32205, United States |
|  | Meridian Clinical Research LLC 1290 Upper Front Street Binghamton, New York, 13901 New York, United States |
|  | Meridian Clinical Research, LLC 4806 Sunnybrook Drive, Sioux City, Iowa, 51106 United States |
|  | Meridian Clinical Research, LLC 3345 N. 107th St, Omaha Nebraska, 68134, United States |
|  | Velocity Clinical Research, Providence, 1598 South County Trail, Suite 204 East Greenwich, Rhode Island 02818, United States |
|  | Lynn Health Science Institute 3555 NW 58th Street, Suite 800, Oklahoma City Oklahoma, 73112, United States |
|  | M3 Wake Research, Inc. 3100 Duraleigh Road, Suites 302, 303, & 304 Raleigh, North Carolina 27612, United States |
|  | Synexus Clinical Research US, Inc 5251 South Green Street., Suite 300 Murray, Utah, 84123 United States |
|  | AMR Tempe/Phoenix, Formerly Clinical Research Consortium Arizona, an AMR company 1492 S Mill Ave, Suite 312, Arizona, 85281 United States. |
|  | Optimal Research, LLC 4911 N Executive Drive Peoria, Illinois, 61614, United States |
|  | Synexus Clinical Research US, Inc. 2081 W. Frye Road, Chandler, Arizona, 85224 United States |
|  | Synexus Clinical Research US, Inc. 11020 Prairie Brook Road, Omaha, Nebraska, 68144 United States |
|  | Optimal Research, LLC, 3030 Venture Lane, Suite 101, Melbourne, Florida, 32934 United States |
|  | Synexus Clinical Research US, Inc. 13111 E Briarwood Ave, Suite 310, Centennial, Colorado, 80112 United States |
|  | AMR East Wichita, Formerly Heartland Associates East Wichita, an AMR company, 1709 South Rock Rd, Wichita Kansas, 67207, United States |
|  | Clinical Research Associates, Inc. 1500 Church Street, Suite 100, Nashville, Tennessee, 37203 United States |
|  | Optimal Research, LLC 15201 Shady Grove Road Suite 202, Rockville, Maryland, 20850 United States |
|  | AMR El Dorado, Formerly Heartland Associates El Dorado, an AMR company  700 West Central, Suite 101 El Dorado, Kansas 67042 |
|  | AMR Kansas City, Formerly Center for Pharmaceutical Research, an AMR company, 1010 Carondelet Drive, Suite 426 Kansas City, Missouri, 64114 United States |
|  | Optimal Research, LLC 2089 Cecil Ashburn Drive Suite 203, 35802, Huntsville, Alabama, 35802 United States |
|  | Synexus Clinical Research US, Inc. 8250 Kenwood Crossing Way Suite 100, Cincinnati, Ohio, 45236, United States |
|  | Synexus Clinical Research US, Inc. 7600 N 15th Street. Suite 185, Phoenix, Arizona, 85020 United States |
|  | Coastal Carolina Research Center, Inc, 4920 O’Hear Avenue, Suite 101 Suite 101, North Charleston, South Carolina, 29405, United States |
|  | Synexus Clinical Research US, Inc. 6601 Lyndale Avenue, South Suite 210, Richfield, Minnesota United States |
|  | Synexus Clinical Research US, Inc. 605 Old Ballas Road, Suite 220 Creve Coeur, Missouri, 63141, United States |
|  | Optimal Research, LLC 5920 Friars Road, Suite 200 San Diego, California, 92108, United States |
|  | AMR Newton, Formerly Heartland Associates Newton, an AMR company, 700 Medical Center Drive, Suite 110, Newton, Kansas, 67114 United States |
|  | DM Clinical Research, 13406 Medical Complex Drive Suites 150, 180, 190, Tomball, Texas, 77375 United States |
|  | The Iowa Clinic, PC 5950 University Ave West Des Moines, Iowa, 50266, United States |
|  | DM Clinical Research, 710 Lawrence Street, Tomball Texas, 77375, United States |
|  | Velocity Clinical Research, Phoenix 6707 N. 19th Avenue, Suite 105, Phoenix, Arizona, 85015 United States |
|  | Synexus Clinical Research US, Inc. 530 South Main Street, Suite 1712, Akron, Ohio, 44311 United States |
|  | Optimal Research, LLC 3200 Red River  Suite 301, Austin, Texas, 78705 United States |
|  | Synexus Clinical Research US, Inc. – Orlando, 100 West Gore Street, Suite 102, Orlando, Florida, 32806 United States |
|  | Trial Management Associates, LLC 1221 Floral Parkway, Suite 101, Wilmington, North Carolina, 28403 United States |
|  | Wilmington Health, 1907 Tradd Court, Wilmington North Carolina, 28401, United States |
|  | Alliance for Multispecialty Research – Norfolk, 6161 Kempsville Circle, Suite 225, Norfolk, Virginia, 23502 United States |
|  | Spartanburg Medical Research 485 Simuel Road, Spartanburg South Carolina, 29303, United States |
|  | Paradigm Clinical Research Centers, Inc., 7777 W 38th Ave. #A-118, Wheat Ridge, Colorado, 80033 United States |
|  | Prestige Clinical Research Center, Inc., 555 Biltmore Way, Suite 205 Coral Gables, Florida, 33134 |
|  | Velocity Clinical Research, Banning 264 N Highland Springs Ave, Suite 5A, Banning, California, 92220 United States |
|  | AMR West Wichita, Formerly Heartland Associates West Wichita, an AMR company, 2260 N. Ridge Road, Suite 240, Wichita, Kansas 67205-1138, United States |
|  | Lynn Institute of Norman 2256 36th Ave NW, Suite 100, Norman, Oklahoma, 73072 United States |
|  | Suncoast Research Group, LLC 2128 West Flagler St, 1st Floor Miami, Florida, 33135, United States |
|  | Texas Center for Drug Development, PA, 6550 Mapleridge St, Ste 201, Houston, Texas, 77081 United States |
|  | AMR Lexington, Formerly Central Kentucky Research Associates, an AMR company, 3475 Richmond Road 3rd Floor, Lexington Kentucky, 40509 United States |
|  | Synexus Clinical Research US, Inc. 6065 Roswell Road, Suite 820, Atlanta, Georgia, 30328 United States |
|  | Johnson County Clin-Trials, LLC 16400 College Blvd, Lenexa, Kansas 66219, United States |
|  | Centennial Medical Group, PC 8186 Lark Brow Road, Suite 201, Elkridge, Maryland, 21075 United States |
|  | Velocity Clinical Research, Cincinnati, 4260 Glendale Milford Road, Suite 201, Cincinnati, Ohio, 45040 United States |
|  | Accellacare, 1958 West State Street Bristol, Tennessee, 37620, United States |
|  | AMR Las Vegas, Formerly Clinical Research Consortium Nevada, an AMR company, 2110 E. Flamingo Road Suite 110, Las Vegas Nevada, 89119, United States |
|  | Synexus Clinical Research US, Inc. 8122 Datapoint Drive, Suite 1010 San Antonio, Texas, 78229, United States |
|  | Accellacare, 180 Wingo Way, Suite 203 Mount Pleasant, South Carolina 29464, United States |
|  | Tekton Research, 4534 West Gate Blvd. Suite 110, Austin, Texas, 78745 United States |
|  | Preferred Primary Care Physicians 1050 Bower Hill Road, Suite 202 Pittsburgh, Pennsylvania, 15243, United States |
|  | Tekton Research, Inc, 1804 Commons Circle, Suite B Yukon, Oklahoma, 73099, United States |
|  | Velocity Clinical Research, Salt Lake City, 3590 West 9000 South, #300, West Jordan, Utah, 84088 United States |
|  | AMR Coral Gables/Miami, Formerly Clinical Research of South Florida, an AMR company, 370 Minorca Avenue, 2nd Floor, Coral Gables, Florida, 33134 United States |
|  | Velocity Clinical Research, Hallandale Beach, 911 E Hallandale Bch Blvd, Hallandale Beach, Florida, 33009 United States |
|  | Clinical Research Atlanta, 175 Country Club Drive, Suite 200A, Stockbridge, Georgia, 30038, United States |
|  | AMR Mobile, Formerly Coastal Clinical Research, an AMR company 100 Memorial Hospital Drive, Annex Building, Suite 3-B Mobile, Alabama, 36608 United States |
|  | Sundance Clinical Research 711 Old Ballas Road, Suite 105 St. Louis, Missouri, 63141 United States |
|  | Synexus Clinical Research US, Inc. - The Villages, 3201 Wedgewood Lane, Suite 100 The Villages, Florida, 32162, United States |
|  | JEM Research Institute, 130 JFK Drive, Suite 201/203 Atlantis, Florida, 33462 United States |
|  | Synexus Clinical Research US, Inc. 861 Coronado Center Drive, Suite 210, Henderson, Nevada, 89052 United States |
|  | Velocity Clinical Research, Albuquerque, 883 Lead Avenue SE Suite B, Albuquerque, New Mexico, 87102 United States |
|  | Wasatch Peak Family Practice 1580 W. Antelope Drive Suite 200(c) & 250(r), Layton, Utah, Utah United States |
|  | Velocity Clinical Research, Spartanburg, 141 Harold Fleming Court Spartanburg, South Carolina, 29303 United States |
|  | Synexus Clinical Research US, Inc. 1275 Olentangy River Road, Suite 202 Columbus, Ohio, 43212 United States |
|  | Amherst Family Practice, PC 1867 Amherst Street, Suite 101, Winchester, Virginia, 22601 United States |
|  | Simon-Williamson Clinic 832 Princeton Ave SW, Birmingham, Alabama, 35211 United States |
|  | Synexus Clinical Research US, Inc. 1657 East Greenville Street Anderson, South Carolina, 29621-2062 United States |
|  | Synexus Clinical Research US, Inc. - St. Petersburg, 6010 Park Boulevard Pinellas Park, Florida, 33781 United States |
|  | Synexus Clinical Research US, Inc. 33 North Dearborn, Suite 500, Chicago, Illinois, 60602 United States |
|  | OGFP Building, LLC DBA Orange Grove Family Practice, 2155 West Orange Grove Road, Tucson, Arizona, 85741 United States |
|  | Synexus Clinical Research US, Inc. 9 Medical Parkway, Plaza 4, Suite 202, Dallas, Texas, 75234 United States |
|  | AMR Fort Myers, Formerly Clinical Physiology Associates, an AMR company, 13670 Metropolis Avenue, Suite 105 Fort Myers, Florida, 33912, United States |
|  | Christie Clinic Clinical Research, LLC, 1801 Windsor Road, Champaign Illinois, 61822, United States |
|  | Charlottesville Medical Research, LLC, 325 Winding River Lane, Suite 102, Charlottesville, Virginia, 22911 United States |
|  | Headlands Research LLC, 7300 Sand Lake Commons Blvd, Suite 200, Med Plex A, Orlando, Florida, 32819 United States |
|  | Preferred Primary Care Physicians 202 Jacob Murphy Lane, Suite 201, Uniontown, Pennsylvania, 15401 United States |
|  | Spectrum Medical, Inc. 109 Bridge Street, Danville Virginia, 24541, United States |
|  | Accellacare, 3521 Haworth Drive, Suite 100 Raleigh, North Carolina, 27609 United States |
|  | Centex Studies Inc., 751 Bayou Pines East Drive, Suite L Lake Charles, Louisiana, 70601 United States |
|  | Accellacare Research of Cary 530 New Waverly Place Suite 200A, Cary, North Carolina, 27518 United States |
|  | AMR Knoxville, Formerly New Orleans Center for Clinical Research - Knoxville, an AMR company 801 N. Weisgarber Road, Suite 100, Knoxville, Tennessee, 37909 United States |
|  | Accellacare, 1901 South Hawthorne Road, Suite 306, Winston-Salem, North Carolina 27103, United States |
|  | Encompass Clinical Research 10225 Austin Drive, Suite 203 Spring Valley, California 91978 United States |
|  | Synexus Clinical Research US, Inc. 11080 E. Artesia Boulevard, Suite A Cerritos, 90703, United States |
|  | Clinical Trials of Texas, Inc. San Antonio Austin Cluster 5430 Fredericksburg Road, Suite 200, San Antonio, Texas, 78229 United States |
|  | Health Research of Hampton Roads, Inc, 720 Thimble Shoals Blvd., Suite 110, Newport News, Virginia, 23606 United States |
|  | The South Bend Clinic Center for Research, 211 N. Eddy Street, South Bend, Indiana, 46617, United States |
|  | Synexus Clinical Research US, Inc. 144 East 44th Street, Floor 2, New York, New York, 10017, United States |
|  | Velocity Clinical Research, Grants Pass, 1722 Williams Highway Suite 109A, Grants Pass, Oregon, 97527 United States |
|  | Central Maine Medical Center 76 High Street, Suite 204, Lewiston, Maine, 04240 United States |
|  | Healthcare Clinical Data Inc. 1065 N.E. 125th Street, Suite 417, North Miami, Florida 33161 |
|  | AMR New Orleans, Formerly New Orleans Center for Clinical Research - New Orleans, an AMR company 2820 Canal Steet, New Orleans Louisiana, 70119 United States |
|  | Velocity Clinical Research, Medford 3121 Signature Court, Medford Oregon, 97504, United States |
|  | Velocity Clinical Research, Chula Vista, 752 Medical Center Court Chula Vista, California, 91911, United States |
|  | Velocity Clinical Research, Valparaiso, 55 University Drive, Suite 106 Valparaiso, Indiana, 46383 United States |
|  | Velocity Clinical Research, San Diego, 5565 Grossmont Center Drive Building 2, Suite 1 San Diego, California, 91942 United States |
|  | Baptist Health Center for Clinical Research, 9601 Baptist Health Drive, Medical Towers 1, Suite 310, Little Rock, Arkansas, 72205 United States |
|  | ARS - Sandhill Research 4751 66th St. N. St. Petersburg, Florida, 33709 United Sates |
|  | Research Your Health 6020 West Parker Road, Suite 305, Plano, Texas,75093 United States |
|  | Velocity Clinical Research, Gulfport 401 Youngsville Highway |
|  | Tekton Research, Inc., 3560 Delaware Street, Suite 105 Beaumont, Beaumont, Texas 77706, United States |
|  | Velocity Clinical Research, New Smyrna Beach, 1410 S Ridgewood Avenue Edgewater, Florida, 32132 United States |
|  | MediSphere Medical Research Center, an AMR affiliate, 2345 West Franklin Street, Suite 101B, Evansville, Indiana, 47712 United States |
|  | Synexus Clinical Research US, Inc.- 161 Thunder Drive, Suite 106 Vista, California, 92083 United States |
|  | Tekton Research – San Antonio 4805 Fredericksburg Rd, San Antonio, Texas, 78229 United States |
|  | Mercury Street Medical Group 300 West Mercury Street Butte, Montana, 59701, United States |
|  | Tekton Research, Inc, 2126 Milestone Drive Suite 101 Fort Collins, Colorado, 80525 United States |
|  | Finger Lakes Clinical Research 885 Winton Road South Rochester, New York, 14618 United States |
|  | ARS - Lake Oconee 119 Harmony Crossing, Suite 3, Eatonton, Georgia, 31024 United States |
|  | CenExel Atlanta Center for Medical Research, 501 Fairburn Road, SW, Atlanta, Georgia, 30331 United States |
|  | Tulane University School of Medicine, 1430 Tulane Ave, New Orelans Louisiana, 70112 United States |
|  | Clinical Trials of America 3201 Armand Street Monroe, Louisiana, 71201 United States |
|  | Monroe Biomedical Research 343 Venus Street Monroe, North Carolina, 28112 United States |
|  | Palm Beach Research Center 2277 Palm Beach Lakes Blvd. West Palm Beach, Florida, 33409 United States |
|  | Velocity Clinical Research, Gaffney 1529 N. Limestone St. Gaffney, South Carolina, 29340 United States |
|  | Clinical Neuroscience Solutions, Inc. 618 East South Street Suite 100, Orlando, Florida 32801 United States |
|  | American Health Research, Inc. 8045 Providence Road, Suite 200 Charlotte, North Carolina, 28277 United States |
|  | Vilo Research Group Inc, 3100 Broadway St, Suite 104E Houston, Texas, 77017, United States |
|  | U of L - Physicians Pulmonology 501 E. Broadway, Louisville, Kentucky, 40202 United States |
|  | North Texas Infectious Diseases Consultants, 3409 Worth Street, Suite 710/740 Dallas, Texas, 75246, United States |
|  | Richmond Behavioral Associates ERG Clinical Research - New York PLLC, 260 Chrisotpher Lane Staten Island, New York, 10314 United States |
|  | Harlem Hospital Center, 215 West 125th St., Suite 302 New York, New York, 10027 United States |
|  | Jefferson City Medical Group 1241 W Stadium Blvd Jefferson City, Missouri, 65109 United States |
|  | United Medical Research 5111 South Ridgewood Port Orange, Florida, 32127 United States |
|  | M3-Emerging Medical Research 120 William Penn Plaza Durham, North Carolina, 27704 United States |
|  | Hope Clinical Research, 22030 Sherman Way, Suite 101 & 110 Canoga Park, California, 91303 United States |
|  | CHEAR Center LLC, 384 East 149th Street, Suite 205 Bronx, New York, 10455 United States |
|  | Central Texas Clinical Research, LLC, 900 East 30th Street, Suite 302, Austin, Texas, 78705 United States |
|  | Suncoast Research Associates, LLC 9260 Sunset Drive, Suite 107, Miami, Florida, 33173 United States |
|  | Tekton Research, Inc 275 South Main, Street #206, Longmont, Colorado, 80501 United States |
|  | Altus Research, Inc. 4671 S Congress Avenue, Suite 200B, Lake Worth, Florida, 33461 United States |
|  | DBC Research, Corp 18503 Pines Blvd, Pembroke Pines, Florida, 33029-1404 United States |
|  | ARS - Birmingham Clinical Research Unit, 2017 Canyon Road, Suite 41, Birmingham, Alabama, 35216 United States |
|  | Central Research Associates, Inc. 2660 10th Ave. S., Building 1, Suite 735, Birmingham, Alabama, 35205 United States |
|  | DBC Research, Corp 7707 N. University Dr., Suite 106, Tamarac, Florida, 33321 United States |
|  | Accel Research Sites 2135 Harden Blvd., Lakeland, Florida, 33803 United States |
|  | Clinical Site Partners, LLC, CSP-Miami 12600 SW 120th street, Suite 117, Miami, Florida, 33186 United States |
|  | New Horizon Research Center, Inc 11401 SW 40th Street, Suite 101, Miami, Florida, 33165 United States |

# Eligibility Criteria

Potential participants had to satisfy all of the following criteria to be enrolled in the current study. The eligibility criteria outlined below were current as of the final amendment (amendment five [11 October 2022]).

*Inclusion criteria*

1. Each participant must sign an informed consent form (ICF) indicating that the participant understands the purpose, procedures and potential risks and benefits of the study, and is willing to participate in the study.
2. Participants must be willing and able to adhere to the prohibitions and restrictions specified in this protocol.
3. Participants must be ≥60 years old on the day of signing the ICF and expected to be available for the duration of the study.
4. Before randomisation, a participant must be:
   a. Postmenopausal (defined as no menses for 12 months without an alternative medical cause); and
   b. Not intending to conceive by any method.

Note: Participants who have had hysterectomies are also eligible for the study.

5. In the investigator’s clinical judgment, the participant must be in stable health at the time of vaccination. Participants may have underlying illnesses, such as hypertension, congestive heart failure (CHF), chronic obstructive pulmonary disease (COPD), type 2 diabetes, hyperlipoproteinaemia, or hypothyroidism, as long as their symptoms and signs are stable and medically controlled in the judgment of the investigator at the time of vaccination, and these conditions receive routine follow-up by the participant’s healthcare provider. Participants will be included on the basis of medical history and vital signs taken between ICF signature and vaccination.
6. From the time of vaccination through 3 months postvaccination, the participant agrees not to donate blood.
7. Must be able to read, understand, and complete questionnaires in the eDiary.
8. Must be willing to provide verifiable identification and to have means to be contacted and to contact the investigator during the study.
9. Must be able to work with smartphones/tablets/computers.

*Exclusion criteria*

Any potential participant who met any of the following criteria was excluded from participating in the study:

1. Those with a serious clinically unstable condition (eg, end-stage renal disease with or without dialysis, clinically unstable cardiac disease), Alzheimer’s disease, or any other condition for which, in the opinion of the investigator, participation would not be in the best interest of the participant (eg, compromise well-being) or that could prevent, confound, or limit the protocol-specified assessments.
2. Those with a history of malignancy within 5 years before screening not in the following categories:
   a. Potential participants with squamous and basal cell carcinomas of the skin and carcinoma in situ of the cervix may be enrolled at the discretion of the investigator.
   b. Potential participants with a history of malignancy within 5 years, which is considered cured with minimal risk of recurrence per investigator’s judgment, can be enrolled.
3. Those who had major surgery (eg, major cardiopulmonary or abdominal operations) as per the investigator’s judgment within 4 weeks before vaccination, or will not have fully recovered from surgery, or has major surgery planned during the time the participant is expected to participate in the study.
4. Those who had abnormal function of the immune system resulting from:
   a. Clinical conditions (eg, autoimmune disease or immunodeficiency) expected to have an impact on the immune response elicited by the study vaccine. Participants with autoimmune disease (eg, autoimmune-mediated thyroid disease, autoimmune inflammatory rheumatic disease such as rheumatoid arthritis, and type 1 diabetes) that is stable and inactive without the use of systemic immunomodulators and glucocorticoids may be enrolled at the discretion of the investigator. Participants with stable/well-controlled HIV infection were also allowed to enroll in the study.
   b. Chronic or recurrent use of systemic corticosteroids within 2 months before administration of study vaccine and during the study. A substantially immunosuppressive steroid dose is considered to be ≥2 weeks of daily receipt of 20 mg of prednisone or equivalent.

Note: Ocular, topical, intra-articular, or inhaled steroids were allowed.

c. Administration of antineoplastic and immunomodulating agents (eg, cancer chemotherapeutic agents, or radiotherapy within 6 months before administration of study vaccine and during the study).

Note: Ocular and topical antineoplastic and immunomodulating agents were allowed.

5. Those who have received or planned to receive:
   a. Licensed live-attenuated vaccines within 28 days before or after planned administration of study vaccination.
   b. Other licensed (not live) vaccines within 14 days before or after planned administration of study vaccination.
6. Those with an acute illness (including acute respiratory illnesses) or body temperature of ≥38·0ºC (≥100·4ºF) within 24 hours prior to administration of study vaccine.

Note: Enrolment at a later date was permitted.

7. Those who had major psychiatric illness and/or drug or alcohol abuse that, in the investigator’s opinion, would compromise the participant’s safety and/or compliance with the study procedures.
8. Those with known or suspected allergy or history of anaphylaxis or other serious adverse reactions to vaccines or vaccine components (including any of the constituents of the study vaccine). Refer to the latest version of the Investigator’s Brochure for Ad26/protein preF RSV vaccine.
9. Those with a history of acute polyneuropathy (eg, Guillain-Barré syndrome) or chronic idiopathic demyelinating polyneuropathy.
10. Those who received hematopoietic stem cell transplant in medical history, immunoglobulins in the 2 months, immunoglobulins specific to RSV, human metapneumovirus, or parainfluenza viruses in the 12 months, apheresis therapies in the 4 months, or blood products in the 4 months before the planned administration of the study vaccine or had any plans to receive such treatment during the study.

Note: Use of monoclonal antibodies (mAbs) before administration of study vaccine and

during the study conduct was permitted with the exception of those targeting T cells (anti-

CD3, CD4, CD33, and CD52), B cells (anti-CD45, CD19, CD20, CD22, CD27, CD38,

and CD138) and check point inhibitors (PD-1, PDL-1 and CTLA-4). Examples of disallowed mAbs are: muromonab-CD3, gemtuzumab ozogamicin, alemtuzumab, bevacizumab, rituximab, ofatumumab, ocrelizumab, tositumomab, veltuzumab, binutuzumab, epratuzumab, pembrolizumab, nivolumab, atezolizumab, avelumab, durvalumab and CTLA-4 targeting ipilimumab. Participants receiving these mAbs in the 2 months before the planned administration of the study vaccine or who planned to receive these mAbs during the study were excluded from participating in the study.

11. Those who received an investigational drug or used an invasive investigational medical device within 30 days or received an investigational vaccine within 6 months before the planned administration of the study vaccine or were currently enrolled or planned to participate in another investigational study during this study.

Note: Participation in an observational clinical study (ie, without intervention) or in the

observational phase of interventional studies was allowed upon approval of the sponsor or its delegate.

12. Those with a contraindication to intramuscular injections and blood draws (eg, bleeding disorders).
13. Those who received an RSV vaccine in a previous RSV vaccine study.
14. Employees of the investigator or study site, with direct involvement in the proposed study or other studies under the direction of that investigator or study site, as well as family members of the employees or the investigator, or an employee of the sponsor.
15. Those who, in the opinion of the investigator, were unlikely to adhere to the requirements of the study or were unlikely to complete the vaccination and observation.
16. Those who could not communicate reliably with the investigator.
17. For participants in the safety subset only: Participants who had considerable scarring, tattoos, abrasions, cuts, or infections over the deltoid region of both arms that, in the investigator’s opinion, could interfere with evaluation of injection-site local reactions.
18. Those who received or planned to receive a severe acute respiratory syndrome coronavirus 2 (SARS-CoV-2) vaccine:
    a. Live-attenuated SARS-CoV-2 vaccine within 28 days before or after planned administration of the study vaccine.
    b. Nonlive SARS-CoV-2 vaccine within 14 days before or after planned administration of the study vaccine.
    c. A viral-vectored SARS-CoV-2 vaccine within 28 days before or after planned administration of the study vaccine.
19. Those who received or planned to receive an Ad26-vectored vaccine at any time prior to randomisation until 28 days after study vaccination (this does not apply to SARS-CoV-2 vaccines; please refer to exclusion criterion 18).
20. Those with a history of thrombosis with thrombocytopenia syndrome or heparin-induced thrombocytopenia and thrombosis.

# Laboratory Analyses

*Confirmation of RSV infection using RT-PCR assays*

Confirmation of RSV infection in nasal swabs was performed at the site laboratory by RT-PCR using a BioFire^®^ Filmarray^®^ Respiratory panel (RP2·1-EZ) according to manufacturer instructions. If at least one nasal swab from an acute respiratory infection (ARI) episode could not be tested locally at the site (i.e., due to instrument malfunction, unavailability of the device in the country/territory or at the site, or shortage of testing reagents), sample(s) were sent to a central laboratory for testing the with BioFire^®^ Filmarray^®^ Respiratory panel (RP2·1-EZ). Sputum samples were tested centrally using the BioFire^®^ Filmarray^®^ Pneumonia panel (BioFire Diagnostics, LLC, Salt Lake City, UT). GeneXpert^®^ (Cepheid^®^, Sunnyvale, CA) was used for participants from China.

*Virus neutralisation assays*

Virus neutralisation assays used a recombinant RSV-A2-firefly luciferase (FFL) virus or an RSV-B virus (RSV-B1Agdup-FFL) expressing either the RSV-B–Buenos Aires strain with 60-nucleotide duplication in the G gene or the RSV-B1 strain F protein.

Virus neutralising antibodies were quantified in A549 cells based on the reduction in FFL reporter gene expression after a single RSV infection. Briefly, a fixed number of RSV viral particles were mixed with serially diluted serum samples. Following a 1-hour incubation, A549 cells were added. RSV infection was measured after ~20 hours using a luciferase gene expression system. Luciferase activity levels in serially diluted samples were plotted in a four-parameter logistic regression curve, and the geometric mean half-maximum inhibitory concentration (IC_50_) was reported as the virus neutralising antibody titre.

*Enzyme-linked immunosorbent assays*

Immunoglobulin G (IgG) binding to biotinylated RSV prefusion conformation-stabilised F (preF) protein was evaluated in serially diluted serum samples according to a standard ELISA protocol. Anti-RSV preF serum IgG concentrations were calculated using Gen5™ Secure 2·0 (BioTek) software by referring the luminescence readout of samples to the four-parameter logistic fit of the standard curve.

*Enzyme-linked immune absorbent spot assay*

RSV-F–specific interferon-γ (IFN-γ) producing T cells were quantified using IFN-γ enzyme-linked immune absorbent spot (ELISpot) following RSV F peptide stimulation. Briefly, peripheral blood mononuclear cells (PBMCs) collected from participants in the immunogenicity subset were thawed, rested overnight, and then stimulated with peptide mixtures (15-amino acid peptides overlapping by 11 amino acids; 2 µg/mL per peptide with 2×10^5^ cells per well) matched to the RSV preF protein vaccine insert for 16 to 28 hours. Spot-forming cells (SFCs) per well were counted with an ELISpot reader and reported as SFCs per 1 million PBMCs after subtraction of the background IFN-γ secretion in the absence of antigen-specific stimulation.

# Thrombotic events to be reported as potential adverse events of special interest^1^

Prior to amendment two, symptoms, signs, and conditions indicating thrombosis (suspected deep vessel venous or arterial thrombotic events) and/or thrombocytopenia (platelet count below the lower limit of normal for the testing laboratory) were recorded and prospectively reported as potential adverse events of special interest (AESIs) by investigators within 24 hours of awareness. A retrospective case retrieval was also performed to allow assessment of cases that occurred prior to protocol amendment two using standardised Medical Dictionary for Regulatory Activities (Version 24·1) queries that captured thrombotic and thrombocytopenic events; such events were described as potential AESIs by programmed definition. The list of thrombotic events to be reported to the sponsor as potential AESIs were MedDRA preferred terms for large vessel thrombosis and embolism, including aortic embolus, aortic thrombosis, aseptic cavernous sinus thrombosis, brain stem embolism, brain stem thrombosis, carotid arterial embolus, carotid artery thrombosis, cavernous sinus thrombosis, cerebral artery thrombosis, cerebral venous sinus thrombosis, cerebral venous thrombosis, superior sagittal sinus thrombosis, transverse sinus thrombosis, mesenteric artery embolism, mesenteric artery thrombosis, mesenteric vein thrombosis, splenic artery thrombosis, splenic embolism, splenic thrombosis, thrombosis mesenteric vessel, visceral venous thrombosis, hepatic artery embolism, hepatic artery thrombosis, hepatic vein embolism, hepatic vein thrombosis, portal vein embolism, portal vein thrombosis, portosplenomesenteric venous thrombosis, splenic vein thrombosis, spontaneous heparin-induced thrombocytopenia syndrome, femoral artery embolism, iliac artery embolism, jugular vein embolism, jugular vein thrombosis, subclavian artery embolism, subclavian vein thrombosis, obstetrical pulmonary embolism, pulmonary artery thrombosis, pulmonary thrombosis, pulmonary venous thrombosis, renal artery thrombosis, renal embolism, renal vein embolism, renal vein thrombosis, brachiocephalic vein thrombosis, vena cava embolism, vena cava thrombosis, truncus coeliacus thrombosis. Other potential AESIs to be reported included MedDRA preferred terms for more common thrombotic events, including axillary vein thrombosis, deep vein thrombosis, pulmonary embolism, acute myocardial infarction, and stroke.^2^

Both prospectively and retrospectively identified AESIs with concurrent thrombosis and thrombocytopenia were qualified for assessment and were further assessed by a thrombosis with thrombocytopenia syndrome assessment committee consisting of two independent external experts that assessed risk level according to one of the following three criteria: Brighton Collaboration level (level 1-5), Centers for Disease Control and Prevention (CDC) tier (non-tier 1/2, tier 1, or tier 2), and Pharmacovigilance Risk Assessment Committee criteria (confirmed, possible, probable, unlikely, or criteria not met).

# Statistical analyses

Sample size was determined assuming VE for RT-PCR–confirmed RSV-mediated LRTD of 65% during both the first and second years, an incidence of RT-PCR–confirmed RSV-mediated LRTD of ≥0·2% during the first RSV season (2021-2022) since the impact of anti-COVID measures on RSV circulation was unknown at the time of protocol development, an incidence of 0·5% in later RSV seasons in placebo recipients, a randomisation ratio of 2:1 (active vaccine:placebo), an interim analysis plan as previously described, a one-sided α of 2·5%, and an exclusion rate of 10% per year. Simulations showed that, with 15,340 participants in the vaccine group and 7670 participants in the placebo group, the study had ≥90% total power to demonstrate VE against the primary endpoint statistically significant >20% and a point estimate for VE >50%.

The per protocol immunogenicity subset was to include at least 360 participants, with ~50% at increased risk for severe RSV disease and ~50% aged ≥75 years. This subset was also to contain at least 180 participants from North America, at least 180 from Europe, and ~120 from each of the other regions that were a part of the study. For each region, ~50% of the per protocol immunogenicity subset was to be at increased risk of severe RSV disease and ~50% were to be aged ≥75 years.

The simulations and calculations were performed using R version 4.1.3.

# Interim analysis plan

The total alpha will be controlled at 2·5%. A Pocock-like alpha spending function as described by DeMets,^3^ with a target number of events of 76 will be used to spend the alpha over the different analyses. The different alphas to be used during every interim analyses and calculations are provided below.

DB, database; IA, interim analysis; IDMC, independent data monitoring committee; PA: Primary analysis; POS, primary endpoint met

**Alpha spending at the prespecified analysis timepoints**

Alpha Description of how to obtain alpha

α1 – Alpha obtained from Pocock-like alpha spending function with information fractions = [# RSV LRTD events by 15 September 2022 / 76¥, 1]

α2 – Alpha obtained from Pocock-like alpha spending function with information fractions = [# RSV LRTD events by 15 September 2022 / 76¥, # RSV LRTD events by 24 October 2022 /76¥, 1]

α3 – Alpha obtained from Pocock-like alpha spending function with information fractions = [# RSV LRTD events by 15 September 2022 / 76¥, # RSV LRTD events by 24 October 2022 / 76¥, # RSV LRTD events by 22 Jan 2023 / 76¥, 1]

α4 – Alpha obtained from the remaining alpha to be spent (2·5% - total alpha spent during the interim analyses) and the actual information fractions observed at all analyses. That is, the information fraction, IF = [# RSV LRTD events by 15 September 2022 / # RSV LRTD events by 30 April 2023, # RSV LRTD events by 24 October 2022 / # RSV LRTD events by 30 April 2023, # RSV LRTD events by 22 January 2023 / # RSV LRTD events by 30 April 2023, 1]. The final alpha will be calculated using the user-defined alpha spending function approach.

# Statistical Analyses Amendments

There were three amendments to the statistical analysis plan. In the first amendment (19 November 2021), amendments were made to align with the changes made in the clinical trial protocol amendment 3 (outlined in the *Protocol Amendments* section above). In the second amendment (23 September 2022), additional revisions were made to align with protocol amendment 4. Furthermore, additions were made to clarify the way potential adverse events of special interest were to be analysed, the primary analysis definition, the timing used for the end-of-study analysis and population used for this analysis, and how to handle missing RSV ARI end dates that occurred close to the database lock. In addition, methods used to evaluate nasal swabs from local sites in China were updated, and new exploratory endpoints were added related to clinically relevant diseases and the EuroQol-5D (EQ-5D). In the third amendment (5 June 2023), revisions were made to provide information pertaining to the discontinuation of the study, to clarify additional analyses added, to include imputation for humoral assays with both lower limits of quantification and levels of detection reported, and to add new preferred terms per an update to Medical Dictionary for Regulatory Activities, Version 25·1. In addition, minor updates were made to align the definitions of the end of the first year and the start of the second year between safety and efficacy.

# Sensitivity Analyses

Sensitivity analysis was repeated as described for statistical analysis of the primary endpoint based on the full analysis set (FAS), not taking into account the restrictions on time of LRTD onset (≥14 days). Additional sensitivity analyses included an exact binomial test, which did not take into account strata or follow-up time, based on the per protocol efficacy population and the FAS. The analysis of the primary endpoint was repeated, but participants with RSV infection and a coinfection with at least one other respiratory virus (ie, confirmed using the BioFire^®^ Filmarray Respiratory panel for nasal swabs, or the BioFire^®^ Filmarray Pneumonia panel for a sputum sample, when available) were not considered as a case for that episode as the aetiology of symptoms was indeterminate. The analysis of the primary endpoint was repeated, taking into account only the RT-PCR test results from the study-specified BioFire^®^ Filmarray Respiratory panel for nasal swabs and the BioFire^®^ Filmarray Pneumonia panel for a sputum sample, when available (i.e., excluding hospital test results with a locally approved RT-PCR test for participants who were hospitalised). Another sensitivity analysis was focused on the programmed case definition of RT-PCR–confirmed RSV-mediated LRTD, not taking into account the clinical evaluation committee adjudication. For sensitivity analyses, VE (=1−relative risk) and the corresponding corrected two-sided confidence interval based on the exact binomial was calculated. The same α′ as in the primary analysis was used for calculating the confidence interval.

A sensitivity analysis using multiple imputations was performed to evaluate the impact of missing RT-PCR results on the primary endpoint. For ARIs where no RT-PCR result could be linked to the considered ARI episode the RT-PCR result (positive or negative) was imputed using the period the ARI occurred, the geographic location (Europe, Canada, the RSV census regions for USA sites [West, North East, South, Midwest], Latin America [Brazil and Chile], South Africa, Thailand and Oceania [Australia and New Zealand]), the age, the risk level (CDC definition) of the participants and the severity of the episode (LRTD or not LRTD) as parameters in the model. The ARI periods were defined based on the distribution of the cases observed during the study.

If one or more RT-PCR test results were available, no imputation was performed. Moreover, the primary analysis model already accounted for dropouts, since participant follow up time was taken into consideration.

# Data Protection and Quality Assurance

The collection and processing of personal data from participants enrolled in the study was limited to those data that were necessary to fulfill the study objectives. The data were collected and processed with adequate precautions to ensure confidentiality and compliance with applicable data privacy protection laws and regulations. Appropriate technical and organisational measures to protect the personal data against unauthorised disclosures or access, accidental or unlawful destruction, or accidental loss or alteration were applied. Sponsor personnel whose responsibilities requiring access to personal data agreed to keep the identity of participants confidential. The informed consent obtained from the participants included explicit consent for the processing of personal data and for the investigator/institution to allow direct access to their original medical records (source data/documents) for study-related monitoring, auditing, independent ethics committee/institutional review board review, and regulatory inspection. This consent also addressed the transfer of the data to other entities and to other countries/territories. Participants had the right to request through the investigator access to their personal data and the right to request rectification of any data that were not correct or complete. Reasonable steps were taken to respond to such a request, taking into consideration the nature of the request, the conditions of the study, and the applicable laws and regulations.

Steps were taken to ensure data accuracy and reliability including the selection of qualified investigators and appropriate study sites, review of protocol procedures with the investigator and study site personnel before the study, and periodic monitoring visits by the sponsor. Written instructions were provided for collection, handling, storage, and shipment of samples. Guidelines for electronic case report form completion were provided and reviewed with study site personnel before the start of the study. The sponsor reviewed the electronic case report form for accuracy and completeness during on-site monitoring visits and after transmission to the sponsor; any discrepancies were resolved with the investigator or designee, as appropriate. After data upload into the study database, data were verified for accuracy and consistency with the data sources.

# Independent Data Monitoring Committee (IDMC)

The IDMC was responsible for monitoring data (both safety and efficacy) and ultimately deciding whether the study was to continue based on outcomes. The IDMC comprised a statistician from Universiteit Hasselt, Belgium, a clinical specialist from Duke University Medical Center, and a chairperson from Harvard Medical School.

A redacted copy of the IDMC Charter can be found at the end of this Supplementary Material. The IDMC received monthly safety updates, starting 6 weeks post first participant dosed until the day that the last participant dosed in the safety subset reached their 28- day post dose visit, and received updates every 2 to 3 months after this until the end of the study. All IDMC reviews were based on a snapshot of the database. The IDMC reviewed unblinded data; with the data package to be reviewed (summary data) displaying the real vaccine identity. The study team was responsible for transferring the blinded data to the statistical support group, whereas the interactive web response system vendor or Secure Data vendor were responsible for securely transferring unblinded randomization data to the statistical support group. In addition to monitoring safety data, the IDMC formally monitored the efficacy endpoints at specified timepoints in an unblinded fashion to determine if success criteria were met.

The IDMC data package (summary data) followed the same statistical methods described in the statistical analysis plan (SAP). The IDMC safety data package consisted of the following tabulations: participant disposition and demographics, serious adverse events (SAEs), related SAEs, AEs with fatal outcome, related AEs with fatal outcome, solicited and unsolicited grade 3 AEs, related AEs, AEs leading to discontinuation. Potential adverse events of special interest (AESIs) qualified for assessment and potential AESI were listed. Other safety summaries could also be requested.

The IDMC efficacy evaluation package contained one table summarizing the vaccine efficacy and corresponding confidence intervals. Data packages were distributed by the statistical support group to the IDMC members via a secure electronic environment to ensure no inappropriate unblinding. A separate data package was made available to the study team where blinded summary data were presented for the pooled groups. The SAP was used to support any IDMC analyses.

The IDMC assessed the null hypothesis of a lower limit (LL) of ≤20% for RT-PCR-confirmed RSV-mediated LRTD versus the alternative hypothesis of a LL >20%. For vaccine efficacy to be demonstrated, in addition, the observed vaccine efficacy was to be >50%. Conclusions from the IDMC review (ie, whether success criteria had been met) were then communicated to the sponsor.

The composition and role of the statistical support group is provided in the IDMC charter. The statistical support group was comprised of a Statistician, a Project Manager, and a Programmer and received blinded datasets from the study team for the purposes of safety updates and interim analyses. The IDMC (with the help of the external statistical support group) was responsible for performing the interim analyses and formulating recommendations for the Sponsor Committee. The role of the Sponsor Committee was to communicate the IDMC recommendations within the Sponsor and identify appropriate actions based on the recommendations of the IDMC.

# Exploratory efficacy objectives and endpoints

Due to the early termination of the study, the sponsor performed a limited selection of the exploratory endpoints at the end-of-study analysis. Results from the Primary analysis are shown when the end of study analysis was not performed.

| **Objective** | **Endpoint** | **Status/location** |
| --- | --- | --- |
| To demonstrate the efficacy of active study vaccine in the prevention of RT-PCR-confirmed RSV-mediated LRTD during the first year when compared to placebo in adults aged 60 years and above | First occurrence of RT-PCR-confirmed RSV- mediated LRTD during the first year, with onset at least 14 days after dosing of study vaccine, and prior to study Day 365 | Final analysis. Supplementary Table S3 |
| To demonstrate the efficacy of active study vaccine in the prevention of RT-PCR-confirmed RSV-mediated ARI during the first year when compared to placebo | First occurrence of RT-PCR-confirmed RSV- mediated ARI during the first year, with onset at least 14 days after dosing of study vaccine, and prior to study Day 365 | Final analysis. Supplementary Table S3 |
| To explore the effect of active study vaccine on RSV A and B infection when compared to placebo | Assessment of the RSV A and B viral load by quantitative RT-PCR | Final analysis. Supplementary Figure S4 |
| To explore the efficacy of active study vaccine in the prevention of any RT-PCR-confirmed RSV- mediated LRTD caused by an RSV A and/or B strain when compared to placebo  -during the first year  -during the second year  -over the whole study | First occurrence of any RT-PCR-confirmed  RSV-mediated LRTD caused by an RSV A strain and/or RSV B strain, respectively, during the considered years;  - with onset at least 14 days after dosing of study vaccine and prior to study Day 365 (first year comparison)  - with onset after study Day 365 (second year comparison)  - with onset at least 14 days after dosing of study vaccine (whole study comparison) | Final analysis. Supplementary Table S4 |
| To explore the efficacy of active study vaccine in the prevention of any RT-PCR-confirmed RSV- mediated ARI caused by an RSV A and/or B strain when compared to placebo  -during the first year  -during the second year  -over the whole study | First occurrence of any RT-PCR-confirmed RSV-mediated ARI caused by an RSV A strain and/or RSV B strain, respectively, during the considered years  - with onset at least 14 days after dosing of study vaccine and prior to study Day 365 (first year comparison)  - with onset after study Day 365 (second year comparison)  - with onset at least 14 days after dosing of study vaccine (whole study comparison) | Final analysis. Supplementary Table S4 |
| To explore the efficacy of active study vaccine in the prevention of at least mild, at least moderate, and at least severe RT-PCR-confirmed RSV LRTD as assessed by the CEC compared to placebo  -during the first year  -during the second year  -over the whole study | First occurrence of any RT-PCR-confirmed RSV LRTD assessed by the CEC as:  -at least mild LRTI  -at least moderate LRTI  -at least severe LRTI during the considered year:  - with onset at least 14 days after dosing of study  vaccine and prior to study Day 365 (first year comparison)  - with onset after study Day 365 (second year comparison)  - with onset at least 14 days after dosing of study vaccine (whole study comparison) | Primary analysis only. Supplementary Table S5 |
| To explore the reduction of symptom severity and time to return to usual health in participants with an RT-PCR-confirmed RSV-mediated ARI and in participants with RT-PCR-confirmed RSV-mediated LRTD when compared to placebo  -during the first year  -during the second year  -over the whole study | In participants with an RT-PCR-confirmed RSV-mediated ARI and in participants with RT-PCR- confirmed RSV-mediated LRTD (during the first year, the second year, or over the whole study, respectively):  o the AUC of the change from baseline in RiiQ Total Symptom score  - the time to return to usual health | Final analysis (RiiQ). Supplementary Figure S2  Primary analysis (time to usual health). Supplementary Figure S3 |
| To explore the reduction of disease severity in participants with an RT-PCR-confirmed RSV-mediated ARI and in participants with RT-PCR- confirmed RSV-mediated LRTD when compared to placebo, by subtype (RSV A, RSV B)  -during the first year  -during the second year  -over the whole study | In participants with an RT-PCR-confirmed RSV-mediated ARI and in participants with RT-PCR- confirmed RSV-mediated LRTD (during the first year, the second year or over the whole study respectively), by subtype (RSV A, RSV B):  -the AUC of the change from baseline in RiiQ Total Symptom score  -the time to return to usual health | Not done |
| To explore the impact of active study vaccine on the course of an RT-PCR-confirmed RSV- mediated ARI and of hMPV- and influenza- mediated respiratory infections, general health status and HRQoL measures when compared to placebo | HRQoL and health status reported by participants on the RiiQ, patient global impression scales, and EQ-5D-5L in participants with an RT-PCR- confirmed RSV-mediated ARI or hMPV and influenza-mediated respiratory infections | Primary analysis only (RSV only)  Supplementary Figure S6 |
| To explore the effect of active study vaccine on each of the following separately: the potential complications (including pneumonia), hospitalizations, emerging therapeutic use and MRU of an RT-PCR-confirmed RSV-mediated ARI, an RT-PCR-confirmed RSV-mediated LRTD, and of hMPV and respiratory infections when compared to placebo  -during the first year  -during the second year  -over the whole study  For influenza, the objective is to explore the incidence of these endpoints | For each of the following separately:  (1) complications, (2) pneumonia, (3) hospitalizations, (4) emerging therapeutic interventions and (5) MRU, all associated with RT-PCR-confirmed RSV-mediated ARI or RT- PCR-confirmed RSV-mediated LRTD during the first year, the second year and over the whole study  - --with onset at least 14 days after dosing of study vaccine and prior to study Day 365 (first year comparison)  - with onset after study Day 365 (second year comparison)  - with onset at least 14 days after dosing of study vaccine (whole study comparison)  Complications, pneumonia, hospitalizations, emerging therapeutic use and MRU associated with hMPV and influenza-mediated respiratory infections will be defined similarly | Final analysis.  Supplementary Table S6 |
| To explore the efficacy of active study vaccine in the prevention of RT-PCR-confirmed hMPV-mediated ARI and RT-PCR-confirmed hMPV-mediated LRTD when compared to placebo in adults aged 60 years and above | First occurrence of RT-PCR-confirmed, hMPV- mediated ARI and RT-PCR-confirmed hMPV- mediated LRTD with onset at least 14 days after dosing of study vaccine | Not done |
| To explore the immune response biomarkers in study participants as correlates of risk of RSV disease and as correlates of protection induced by the active study vaccine | Assessment of the correlation of immune responses with emphasis on neutralizing and binding antibodies with the risk of RSV disease and protection induced by the vaccine | Published separately |
| To explore biomarkers for the diagnosis of RSV infection, RSV-mediated LRTD and hMPV and influenza- mediated respiratory infections | Assessment of blood samples collected during ARI episodes for biomarkers that correlate with RSV infection, RSV-mediated LRTD and hMPV and influenza-mediated respiratory infections | Published separately |
| To explore additional vaccine-elicited immune responses in the Immuno Subset | Assays that may be used include, but are not limited to:  -RSV cross-neutralization of B and/or other A strain(s)  -Antigen specific antibody functional and molecular characterization  -Analysis of neutralizing antibodies to Ad26  -Antigen-specific T-cell immune responses using detailed immunoprofiling | Primary analysis only  (anti-vector antibodies and B17 cross-neutralisation only)  Supplementary Table S7  Supplementary Figure S5 |
| To increase the information on prior medical history (electronic health records, claims, laboratory data from other care settings) in order to further evaluate its potential effect on the response to immunization and the impact of immunization on efficacy and duration of efficacy as well as AEs that may occur during and after completion of the study | Utilization of tokenization and matching procedures for exploratory analysis of participant’s medical data prior to, during, and following participation in the study (real-world data). Analysis will be performed to relate real-world data to vaccine immune responses, efficacy and duration of protection, and AEs | Not done |

# Supplementary Figure S1. Sensitivity analyses for first occurrence of RT-PCR–confirmed RSV-mediated LRTD.

^a^Or GeneXpert^®^ for participants from China.

Ad26, adenovirus type 26; FAS, full analysis set; LRTD, lower respiratory tract disease; preF, prefusion conformation-stabilised RSV F protein; RT-PCR, reverse transcription polymerase chain reaction; RSV, respiratory syncytial virus.

# Supplementary Figure S2. Exploratory endpoint. Total RiiQ™ symptom scores in participants with RT-PCR–confirmed RSV ARIs. (per protocol efficacy set).

Ad26, adenovirus type 26; ARI, acute respiratory infection; AUC, area under the curve; LRTD, lower respiratory tract disease; RSV Vx, prefusion conformation-stabilised RSV F protein; RiiQ™, Respiratory Infection Intensity and Impact Questionnaire; RT-PCR, reverse transcription polymerase chain reaction; RSV, respiratory syncytial virus.

Medians, Q1 and Q3 are presented.

AUCs below the tenth percentile and above the 90th percentile (based on all participants with an RT-PCR–confirmed RSV-mediated ARI) are censored and presented with hollow symbols.

# Supplementary Figure S3. Exploratory endpoint (a) Time to return to usual health for participants with RT-PCR-confirmed RSV LRTD. (b) Time to return to usual health for participants with RT-PCR–confirmed RSV ARI (per protocol efficacy set; Primary analysis).

**a)**

**a)**

**b)**

Return to usual health is depicted using reverse Kaplan-Meier curves.

LRTD, lower respiratory tract disease; RSV Vx, prefusion conformation-stabilised RSV F protein; RT-PCR, reverse transcription polymerase chain reaction; RSV, respiratory syncytial virus.

# Supplementary Figure S4. Exploratory endpoint. RSV A viral load restricted to participants with an RSV A infection and RSV B viral load for participants with an RSV B infection a) first year, b) second year, c) whole study (per protocol efficacy set)

**a)**

**b)**

**c)**

ARI, acute respiratory infection; RSV Vx, prefusion conformation-stabilised RSV F protein;

Mean ±standard error are presented. Viral loads are presented in log10 copies/ml. ARI surveillance stopped on 29 March 2023.

# Supplementary Figure S5. Exploratory endpoint. Titres of neutralising antibodies to RSV B strain (Clinical Isolate 2017): plot of the actual values over time (per protocol immunogenicity set)

GMI geometric mean increase; GMT, geometric mean titre; N: number of participants with data

RSV Vx, prefusion conformation-stabilised RSV F protein; ULOQ, upper limit of quantification. Geometric mean titres with 95% confidence intervals shown in the figure

# Supplementary Figure S6. Exploratory endpoint. Boxplots of area under the curve (AUC) of EQ-5D-5L visual analogue scale (VAS) for participants with RT-PCR-confirmed RSV-mediated ARI or LRTD (per protocol efficacy set)

ARI, acute respiratory infection; EQ-5D-5L, EuroQoL, 5-Dimension 5-Level; LRTD, lower respiratory tract disease; RT-PCR, reverse transcriptase polymerase chain reaction; VAS, visual analogue scale

Median, Q1 and Q3 are presented. AUCs below the 10th percentile and above the 90th percentile (based on all participants with an RT-PCR-confirmed RSV-mediated ARI) are censored and presented with hollow symbols.

# Supplementary Figure S7. Immunogenicity of Ad26.RSV.preF/RSV preF protein at Days 1 and 15 in the immunogenicity subset: RSV A2 neutralising antibodies.

Ad26, adenovirus type 26; GMI, geometric mean increase; GMT, geometric mean titre; IC_50_, half-maximum inhibitory concentration; preF, prefusion conformation-stabilised RSV F protein; RSV, respiratory syncytial virus.

# Supplementary Figure S8. Immunogenicity of Ad26.RSV.preF/RSV preF protein at Days 1 and 15 in the immunogenicity subset: RSV preF A IgG serum antibody response (ELISA).

Ad26, adenovirus type 26; ELISA, enzyme-linked immunosorbent assay; GMI, geometric mean increase; GMT, geometric mean titre; IgG, immunoglobulin G; preF, prefusion conformation-stabilised RSV F protein; RSV, respiratory syncytial virus.

# Supplementary Figure S9. Immunogenicity of Ad26.RSV.preF/RSV preF protein at Days 1 and 15 in the immunogenicity subset: RSV preF B IgG serum antibody response (ELISA).

Ad26, adenovirus type 26; ELISA, enzyme-linked immunosorbent assay; GMI, geometric mean increase; GMT, geometric mean titre; IgG, immunoglobulin G; preF, prefusion conformation-stabilised RSV F protein; RSV, respiratory syncytial virus.

# Supplementary Figure S10. Immunogenicity of Ad26.RSV.preF/RSV preF protein at Days 1 and 15 in the immunogenicity subset: RSV-F–specific T-cell response (IFN-γ ELISpot).

Ad26, adenovirus type 26; ELISpot, enzyme-linked immune absorbent spot; IFN-γ, interferon-γ; LLOQ, lower limit of quantification; PBMC, peripheral blood mononuclear cell; preF, prefusion conformation-stabilised RSV F protein; RSV, respiratory syncytial virus; SFC, spot-forming cell.

# Supplementary Figure S11. Immunogenicity of Ad26.RSV.preF/RSV preF protein at Days 1 and 15 in the immunogenicity subset by age: RSV A2 neutralising antibodies.

Ad26, adenovirus type 26; GMT, geometric mean titre; IC_50_, half-maximum inhibitory concentration; preF, prefusion conformation-stabilised RSV F protein; RSV, respiratory syncytial virus.

# Supplementary Figure S12. Immunogenicity of Ad26.RSV.preF/RSV preF protein at Days 1 and 15 in the immunogenicity subset by age: RSV preF A IgG serum antibodies (ELISA).

Ad26, adenovirus type 26; ELISA, enzyme-linked immunosorbent assay; GMT, geometric mean titre; IgG, immunoglobulin G; preF, prefusion conformation-stabilised RSV F protein; RSV, respiratory syncytial virus.

# Supplementary Figure S13. Immunogenicity of Ad26.RSV.preF/RSV preF protein at Days 1 and 15 in the immunogenicity subset by age: RSV-F–specific T-cell response (IFN-γ ELISpot).

Ad26, adenovirus type 26; ELISpot, enzyme-linked immune absorbent spot; IFN-γ, interferon-γ; LLOQ, lower limit of quantification; PBMC, peripheral blood mononuclear cell; preF, prefusion conformation-stabilised RSV F protein; RSV, respiratory syncytial virus; SFC, spot-forming cell.

# Supplementary Figure S14. Immunogenicity of Ad26.RSV.preF/RSV preF protein at Days 1 and 15 in the immunogenicity subset by risk level: RSV A2 neutralising antibodies.

Ad26, adenovirus type 26; GMT, geometric mean titre; IC_50_, half-maximum inhibitory concentration; preF, prefusion conformation-stabilised RSV F protein; RSV, respiratory syncytial virus.

# Supplementary Figure S15. Immunogenicity of Ad26.RSV.preF/RSV preF protein at Days 1 and 15 in the immunogenicity subset by risk level: RSV preF A IgG serum antibodies (ELISA).

Ad26, adenovirus type 26; ELISA, enzyme-linked immunosorbent assay; GMT, geometric mean titre; IgG, immunoglobulin G; preF, prefusion conformation-stabilised RSV F protein; RSV, respiratory syncytial virus.

# Supplementary Figure S16. Immunogenicity of Ad26.RSV.preF/RSV preF protein at Days 1 and 15 in the immunogenicity subset by risk level: RSV-F–specific T-cell response (IFN-γ ELISpot).

Ad26, adenovirus type 26; ELISpot, enzyme-linked immune absorbent spot; IFN-γ, interferon-γ; LLOQ, lower limit of quantification; PBMC, peripheral blood mononuclear cell; preF, prefusion conformation-stabilised RSV F protein; RSV, respiratory syncytial virus; SFC, spot-forming cell.

# Supplementary Figure S17. Any solicited local adverse events 28 days postvaccination (safety subset, final analysis).

Ad26, adenovirus type 26; preF, prefusion conformation-stabilised RSV F protein; RSV, respiratory syncytial virus.

# Supplementary Figure S18. Systemic adverse events 28 days postvaccination (safety subset, final analysis).

Ad26, adenovirus type 26; preF, prefusion conformation-stabilised RSV F protein; RSV, respiratory syncytial virus

# Supplementary Table S1. Baseline characteristics of the trial participants in the Full analysis set (final analysis)

|  | **Ad26.RSV.preF/**  **RSV preF protein**  **(n=16,709)** | **Placebo**  **(n=8,367)** |
| --- | --- | --- |
| Age, median (Q1, Q3), years | 69 (66, 73) | 69 (66, 73) |
| Age category, n (%) |  |  |
| 60-64 | 1,377 (8·8) | 692 (8·8) |
| 65-74 | 11,340 (72·2) | 5,671 (72·2) |
| 75-84 | 2,807 (17·9) | 1,406 (17·9) |
| ≥85 | 174 (1·1) | 88 (1·1) |
| Sex,* n (%) |  |  |
| Female | 9,063 (54·2%) | 4,452 (53·2%) |
| Male | 7,645 (45·8%) | 3,913 (46·8%) |
| Unknown | 1 (<0·1%) | 2 (<0·1%) |
| Region, n (%) |  |  |
| North America | 10,850 (64·9%) | 5,422 (64·8%) |
| Europe | 2,176 (13·0%) | 1,090 (13·0%) |
| Africa | 1,273 (7·6%) | 639 (7·6%) |
| Asia | 1,653 (9·9%) | 834 (10·0%) |
| Australia/New Zealand | 472 (2·8%) | 238 (2·8%) |
| South America | 285 (1·7%) | 144 (1·7%) |
| Race,* n | 15,697 | 7,857 |
| White, n (%) | 12,649 (75·7%) | 6,297 (75·3%) |
| Black or African American, n (%) | 1,820 (10·9%) | 935 (11·2%) |
| Asian, n (%) | 1,757 (10·5%) | 897 (10·7%) |
|  |  |  |
| Multiple, n (%) | 189 (1·1%) | 101 (1·2%) |
| Native Hawaiian or other Pacific Islander, n (%) | 98 (0·6) | 49 (0·6) |
| Not reported, n (%) | 84 (0·5) | 46 (0·6) |
| American Indian or Alaska Native, n (%) | 58 (0·3) | 17 (0·2) |
| Unknown, n (%) | 53 (0·3) | 25 (0·3) |
| Ethnicity, n (%) |  |  |
| Not Hispanic or Latino | 14,903 (89·2%) | 7,496 (89·6%) |
| Hispanic or Latino | 1,555 (9·3) | 741 (8·9) |
| Not reported | 203 (1·2%) | 96 (1·1%) |
| Unknown | 48 (0·3%) | 34 (0·4%) |
| Smoking status, n | 16,708 | 8,367 |
| Smoker, n (%) | 7,006 (41·9%) | 3,459 (41·3%) |
| Non-smoker, n (%) | 9,702 (58·1%) | 4,908 (58·7%) |
| At increased risk of severe RSV disease, n (%) |  |  |
| No increased risk | 12,494 (74·8%) | 6,217 (74·3%) |
| Increased risk^†^ | 4,215 (25·2%) | 2,150 (25·7%) |
| Asthma | 1,098 (26·0%) | 546 (25·4%) |
| COPD | 301 (7·1%) | 159 (7·4%) |
| CHF | 1,521 (36·1%) | 781 (36·3%) |
| Other chronic heart diseases | 1,719 (40·8%) | 843 (39·2%) |
| Other chronic lung diseases^‡^ | 369 (8·8%) | 182 (8·5%) |

Abbreviations: Ad26, adenovirus type 26; CHF, congestive heart failure; COPD, chronic obstructive pulmonary disease; preF, prefusion conformation-stabilised RSV F protein; Q, quartile; RSV, respiratory syncytial virus.

*Sex and race were self-reported.

^†^Participants at increased risk of severe RSV disease were defined as those with underlying chronic heart disease (CHF, coronary artery disease [eg, angina pectoris, ischemic cardiomyopathy, history of myocardial infarction, history of coronary artery bypass graft, or coronary artery stent]) and chronic lung diseases (eg, asthma and chronic COPD).

^‡^The electronic case report form included a category labelled “other chronic lung diseases” that could be chosen at the discretion of the investigator.

# Supplementary Table S2. Secondary confirmatory endpoints: percentage of participants per group meeting the secondary endpoints tested at the end of study and vaccine efficacy of their first occurrence (per protocol efficacy set)

|  | **Ad26.RSV.preF/RSV preF protein** | **Placebo** | **Vaccine efficacy (95% CI)** | **Alpha** |
| --- | --- | --- | --- | --- |
| Number of participants primary analysis | 15,337 | 7683 |  |  |
| First occurrence of: |  |  |  |  |
| Any RT-PCR-confirmed RSV-mediated ARI* | 32 (0.2%) | 49 (0.6%) | 67.5 (46.7, 80.5) | 1.837% |
| Number of participants end of study analysis | 16,263 | 8152 |  |  |
| First occurrence of: |  |  |  |  |
| Any RT-PCR-confirmed RSV-mediated LRTD during the second year | 16 (0·2%) | 29 (0·6%) | 72·3 (47·3, 86·0) | 2.5% |
| Any RT-PCR-confirmed RSV-mediated ARI during the second year | 39 (0·4%) | 44 (0·9%) | 55·8 (30·4, 72·0) | 2.5% |
| Clinically relevant diseases associated with any RT-PCR-confirmed RSV-mediated ARI over the whole study | 4 (<0·1%) | 11 (0·1%) | 81·8 (38·7, 95·8) | 2.5% |

CI, confidence interval; N, number of participants.

The vaccine efficacy for the secondary endpoint was calculated based on an Exact Poisson regression, with the event rate, defined as the number of cases (with onset at least 14 days after vaccination) over the follow-up time (off-set) as dependent variable and the vaccination group, being at increased risk of RSV disease and age strata (both variables as stratified) as independent variables.

*96.326% CI for the first occurrence of RT-PCR confirmed RSV mediated ARI (since alpha=1.837).

The ARI surveillance stopped on the 29th of March 2023.

# Supplementary Table S3. Exploratory efficacy endpoints: percentage of participants per group meeting the exploratory endpoints related to any confirmed RSV ARI and vaccine efficacy of their first occurrence (per protocol efficacy set, Final analysis)

|  | **Ad26.RSV.preF/**  **RSV preF protein**  n (%) | **Placebo** | **Vaccine efficacy**  **(95% CI)** |
| --- | --- | --- | --- |
|  |  | n (%) |  |
| **Protocol Efficacy Set** | 16,263 | 8,152 |  |
| **First Year** | 16,263 | 8,152 |  |
| **First occurrence of:** |  |  |  |
| Any RT-PCR-confirmed RSV-mediated LRTD | 44 (0·3%) | 63 (0·8%) | 65·2 (48·0, 76·9) |
| Clinically relevant diseases* associated with any RT-PCR- confirmed RSV-mediated LRTD | 3 (<0·1%) | 7 (0·1%) | 78·6 (6·3, 96·4) |
| Any RT-PCR-confirmed RSV-mediated ARI | 77 (0·5%) | 101 (1·2%) | 62·0 (48·4, 72·1) |
| Clinically relevant diseases* associated with any RT-PCR- confirmed RSV-mediated ARI | 4 (<0·1%) | 9 (0·1%) | 77·8 (20·5, 95·0) |
| **Second Year** | 10,031 | 5,011 |  |
| **First occurrence of:** |  |  |  |
| Clinically relevant diseases* associated with any RT-PCR- confirmed RSV-mediated LRTD | 0 | 2 (<0·1%) | 100 (-166·4, 100) |
| Clinically relevant diseases* associated with any RT-PCR- confirmed RSV-mediated ARI | 0 | 2 (<0·1%) | 100 (-166·4, 100) |
| **Whole Study** | 16,263 | 8,152 |  |
| **First occurrence of:** |  |  |  |
| Any RT-PCR-confirmed RSV-mediated LRTD | 60 (0·4%) | 92 (1·1%) | 67·5 (54·5, 76·9) |
| Clinically relevant diseases* associated with any RT-PCR- confirmed RSV-mediated LRTD | 3 (<0·1%) | 9 (0·1%) | 83·4 (33·3, 97·1) |
| Any RT-PCR-confirmed RSV-mediated ARI | 116 (0·7%) | 144 (1·8%) | 59·9 (48·4, 68·9) |

Abbreviations: Ad26, adenovirus type 26; ARI, acute respiratory infection; LRTD, lower respiratory tract disease; preF, prefusion conformation-stabilised RSV F protein; RSV, respiratory syncytial virus; RT-PCR, reverse transcriptase polymerase chain reaction.

Vaccine efficacy was calculated based on an Exact Poisson regression, with the event rate, defined as the number of cases (with onset at least 14 days after vaccination for cases during the first year and the whole study, and after Day 365 for cases during the second year) over the follow-up time (off-set) as dependent variable and the vaccination group, being at increased risk of RSV disease and age strata (both variables as stratified) as independent variables.

The ARI surveillance stopped on the 29th of March 2023.

*defined as Any of the following is associated with RT-PCR-confirmed RSV-mediated ARI: hospitalization, Emergency department visit, per clinical judgement, at least one of the following complications: asthma exacerbation, chronic obstructive pulmonary disease exacerbation, respiratory distress, bronchitis, bronchial hyperreactivity, congestive heart failure exacerbation, cardiac arrhythmia, renal impairment or the presence of X-ray or radiological confirmed pneumonia, respiratory arrest and/or failure, pulmonary embolism, pleural effusion, atelectasis, acute coronary events, acute cerebrovascular events, altered mental status, seizure, syncope, systemic inflammatory response syndrome, new neurological deficit, asthenia, dehydration or metabolic disturbances, decreased oxygen saturation, tachypnoea,need for supplemental oxygen, hypotension, pulmonary function test results supporting diagnosis of LRTD, arterial blood gas results supporting diagnosis of LRTD.

# Supplementary Table S4. Exploratory endpoint: percentage of participants per group meeting the exploratory endpoints related to any confirmed RSV A or B ARI and vaccine efficacy of their first occurrence (per protocol efficacy set, Final analysis)

|  | **Ad26.RSV.preF/**  **RSV preF protein** | **Placebo** | **Vaccine Efficacy**  **(95% CI)** |
| --- | --- | --- | --- |
|  | **n (%)** | **n (%)** |  |
| **Per protocol efficacy set** | 16263 | 8152 |  |
| **First Year** | 16263 | 8152 |  |
| **First occurrence of:**  Any RT-PCR-confirmed RSV A-mediated LRTD | 21 (0·1%) | 20 (0·2%) | 47·5 (-2·0, 72·9) |
| Any RT-PCR-confirmed RSV B-mediated LRTD | 22 (0·1%) | 40 (0·5%) | 72·6 (52·8, 84·5) |
| Any RT-PCR-confirmed RSV A-mediated ARI | 30 (0·2%) | 33 (0·4%) | 54·6 (23·2, 73·3) |
| Any RT-PCR-confirmed RSV B-mediated ARI | 40 (0·2%) | 64 (0·8%) | 68·8 (52·9, 79·5) |
| **Second Year** | 10031 | 5011 |  |
| **First occurrence of:**  Any RT-PCR-confirmed RSV A-mediated LRTD | 9 (0·1%) | 13 (0·3%) | 65·4 (12·4, 86·9) |
| Any RT-PCR-confirmed RSV B-mediated LRTD | 7 (0·1%) | 15 (0·3%) | 76·4 (38·6, 91·9) |
| Any RT-PCR-confirmed RSV A-mediated ARI | 22 (0·2%) | 24 (0·5%) | 54·4 (15·2, 75·6) |
| Any RT-PCR-confirmed RSV B-mediated ARI | 17 (0·2%) | 19 (0·4%) | 55·1 (8·9, 78·1) |
| **Whole Study** | 16263 | 8152 |  |
| **First occurrence of:**  Any RT-PCR-confirmed RSV A-mediated LRTD | 30 (0·2%) | 33 (0·4%) | 54·6 (23·2, 73·3) |
| Any RT-PCR-confirmed RSV B-mediated LRTD | 29 (0·2%) | 55 (0·7%) | 73·7 (58·0, 83·8) |
| Any RT-PCR-confirmed RSV A-mediated ARI | 52 (0·3%) | 56 (0·7%) | 53·7 (31·2, 68·9) |
| Any RT-PCR-confirmed RSV B-mediated ARI | 57 (0·4%) | 83 (1·0%) | 65·7 (51·4, 76·0) |

Abbreviations: Ad26, adenovirus type 26; ARI, acute respiratory infection; LRTD, lower respiratory tract disease; preF, prefusion conformation-stabilised RSV F protein; RSV, respiratory syncytial virus; RT-PCR, reverse transcriptase polymerase chain reaction.

The vaccine efficacy was calculated based on an Exact Poisson regression, with the event rate, defined as the number of cases (with onset at least 14 days after vaccination for cases during the first year and the whole study, and after Day 365 for cases during the second year) over the follow-up time (off-set) as dependent variable and the vaccination group, being at increased risk of RSV disease and age strata (both variables as stratified) as independent variables.

# Supplementary Table S5. Exploratory endpoint Clinical Evaluation Committee assessment of severity (Primary analysis)

|  | **Clinical Evaluation Committee Severity** | | | |
| --- | --- | --- | --- | --- |
|  | **Severe** | **Moderate** | **Mild** | **Unable to assess** |
| RT-PCR-confirmed RSV-mediated LRTD per protocol | 1/48 (2·1%) | 46/48 (95·8%) | 1/48 (2·1%) | 0 |

Only participants with a CEC evaluation are presented. Vaccine and placebo recipients are pooled together.

# Supplementary Table S6. Exploratory endpoint. Clinically relevant diseases: percentage of participants per group with clinically relevant diseases; per protocol efficacy set

|  | **All participants** | | **Increased risk** | | **No increased risk** | |
| --- | --- | --- | --- | --- | --- | --- |
|  | **Ad26.RSV.preF/**  **RSV preF protein** | **Placebo** | **Ad26.RSV.preF/**  **RSV preF protein** | **Placebo** | **Ad26.RSV.preF/**  **RSV preF protein** | **Placebo** |
|  | **n (%)** | **n (%)** | **n (%)** | **n (%)** | **n (%)** | **n (%)** |
| **First Year** | 16263 | 8152 | 4064 | 2098 | 12199 | 6054 |
| **Participants with any RT-PCR-confirmed RSV-mediated LRTD** | 44 (0·3%) | 63 (0·8%) | 11 (0·3%) | 14 (0·7%) | 33 (0·3%) | 49 (0·8%) |
| Any clinically relevant disease | 3 (<0·1%) | 7 (0·1%) | 1 (<0·1%) | 2 (0·1%) | 2 (<0·1%) | 5 (0·1%) |
| Hospitalization | 0 | 2 (<0·1%) | 0 | 0 | 0 | 2 (<0·1%) |
| Emergency Department visit | 1 (<0·1%) | 3 (<0·1%) | 0 | 0 | 1 (<0·1%) | 3 (<0·1%) |
| Complications | 2 (<0·1%) | 6 (0·1%) | 1 (<0·1%) | 2 (0·1%) | 1 (<0·1%) | 4 (0·1%) |
| Decreased oxygen saturation | 0 | 1 (<0·1%) | 0 | 0 | 0 | 1 (<0·1%) |
| Tachypnoea | 1 (<0·1%) | 1 (<0·1%) | 0 | 0 | 1 (<0·1%) | 1 (<0·1%) |
| Need for supplemental oxygen | 0 | 2 (<0·1%) | 0 | 0 | 0 | 2 (<0·1%) |
| **Participants with any RT-PCR-confirmed** | 77 (0·5%) | 101 | 20 (0·5%) | 25 (1·2%) | 57 (0·5%) | 76 (1·3%) |
| **RSV-mediated ARI** |  | (1·2%) |  |  |  |  |
| Any clinically relevant disease | 4 (<0·1%) | 9 (0·1%) | 1 (<0·1%) | 3 (0·1%) | 3 (<0·1%) | 6 (0·1%) |
| Hospitalization | 0 | 2 (<0·1%) | 0 | 0 | 0 | 2 (<0·1%) |
| Emergency Department visit | 1 (<0·1%) | 4 (<0·1%) | 0 | 1 (<0·1%) | 1 (<0·1%) | 3 (<0·1%) |
| Complications | 2 (<0·1%) | 7 (0·1%) | 1 (<0·1%) | 3 (0·1%) | 1 (<0·1%) | 4 (0·1%) |
| Decreased oxygen saturation | 1 (<0·1%) | 1 (<0·1%) | 0 | 0 | 1 (<0·1%) | 1 (<0·1%) |
| Tachypnoea | 1 (<0·1%) | 3 (<0·1%) | 0 | 1 (<0·1%) | 1 (<0·1%) | 2 (<0·1%) |
| Need for supplemental oxygen | 0 | 2 (<0·1%) | 0 | 0 | 0 | 2 (<0·1%) |
| Pulmonary function test results supporting diagnosis of LRTD | 0 | 1 (<0·1%) | 0 | 1 (<0·1%) | 0 | 0 |
| **Second Year** | 10031 | 5011 | 2588 | 1345 | 7443 | 3666 |
| **Participants with any RT-PCR-confirmed RSV-mediated LRTD** | 16 (0·2%) | 29 (0·6%) | 4 (0·2%) | 12 (0·9%) | 12 (0·2%) | 17 (0·5%) |
| Any clinically relevant disease | 0 | 2 (<0·1%) | 0 | 0 | 0 | 2 (0·1%) |
| Emergency Department visit | 0 | 1 (<0·1%) | 0 | 0 | 0 | 1 (<0·1%) |
| Tachypnoea | 0 | 1 (<0·1%) | 0 | 0 | 0 | 1 (<0·1%) |
| **Participants with any RT-PCR-confirmed RSV-mediated ARI** | 39 (0·4%) | 44 (0·9%) | 8 (0·3%) | 15 (1·1%) | 31 (0·4%) | 29 (0·8%) |
| Any clinically relevant disease | 0 | 2 (<0·1%) | 0 | 0 | 0 | 2 (0·1%) |
| Emergency Department visit | 0 | 1 (<0·1%) | 0 | 0 | 0 | 1 (<0·1%) |
| Tachypnoea | 0 | 1 (<0·1%) | 0 | 0 | 0 | 1 (<0·1%) |
| **Whole Study** | 16263 | 8152 | 4064 | 2098 | 12199 | 6054 |
| **Participants with any RT-PCR-confirmed RSV-mediated LRTD** | 60 (0·4%) | 92 (1·1%) | 15 (0·4%) | 26 (1·2%) | 45 (0·4%) | 66 (1·1%) |
| Any clinically relevant disease | 3 (<0·1%) | 9 (0·1%) | 1 (<0·1%) | 2 (0·1%) | 2 (<0·1%) | 7 (0·1%) |
| Hospitalization | 0 | 2 (<0·1%) | 0 | 0 | 0 | 2 (<0·1%) |
| Emergency Department visit | 1 (<0·1%) | 4 (<0·1%) | 0 | 0 | 1 (<0·1%) | 4 (0·1%) |
| Complications | 2 (<0·1%) | 6 (0·1%) | 1 (<0·1%) | 2 (0·1%) | 1 (<0·1%) | 4 (0·1%) |
| Decreased oxygen saturation | 0 | 1 (<0·1%) | 0 | 0 | 0 | 1 (<0·1%) |
| Tachypnoea | 1 (<0·1%) | 2 (<0·1%) | 0 | 0 | 1 (<0·1%) | 2 (<0·1%) |
| Need for supplemental oxygen | 0 | 2 (<0·1%) | 0 | 0 | 0 | 2 (<0·1%) |
| **Participants with any RT-PCR-confirmed** | 116 | 144 | 28 (0·7%) | 40 (1·9%) | 88 (0·7%) | 104 |
| **RSV-mediated ARI** | (0·7%) | (1·8%) |  |  |  | (1·7%) |
| Any clinically relevant disease | 4 (<0·1%) | 11 (0·1%) | 1 (<0·1%) | 3 (0·1%) | 3 (<0·1%) | 8 (0·1%) |
| Hospitalization | 0 | 2 (<0·1%) | 0 | 0 | 0 | 2 (<0·1%) |
| Emergency Department visit | 1 (<0·1%) | 5 (0·1%) | 0 | 1 (<0·1%) | 1 (<0·1%) | 4 (0·1%) |
| Complications | 2 (<0·1%) | 7 (0·1%) | 1 (<0·1%) | 3 (0·1%) | 1 (<0·1%) | 4 (0·1%) |
| Decreased oxygen saturation | 1 (<0·1%) | 1 (<0·1%) | 0 | 0 | 1 (<0·1%) | 1 (<0·1%) |
| Tachypnoea | 1 (<0·1%) | 4 (<0·1%) | 0 | 1 (<0·1%) | 1 (<0·1%) | 3 (<0·1%) |
| Need for supplemental oxygen | 0 | 2 (<0·1%) | 0 | 0 | 0 | 2 (<0·1%) |
| Pulmonary function test results supporting diagnosis of LRTD | 0 | 1 (<0·1%) | 0 | 1 (<0·1%) | 0 | 0 |

The ARI surveillance stopped on the 29th of March 2023. The risk level is presented as collected based on the CDC definition. The number of participants from the Per Protocol Efficacy Set is used as denominator. The first occurrence is summarised.

# Supplementary Table S7. Exploratory endpoint. Titers of neutralising antibodies against the Ad26 vector (Per Protocol Immunogenicity Set)

|  | **Ad26.RSV.preF/**  **RSV preF protein**  **N=235** | **Placebo**  **N=117** |
| --- | --- | --- |
| N | 234 | 117 |
| Geometric mean (95% CI) | 49 (37; 64) | 48 (32; 70) |
| Percentage ≥ LOD, n (%) | 122 (52·1%) | 60 (51·3%) |

N, number of participants with data; CI, confidence interval; LOD, limit of detection

# Supplementary Table S8. Baseline characteristics of participants in the per protocol immunogenicity subset (Primary analysis)

|  | **Ad26.RSV.preF/**  **RSV preF protein**  **(n=216)** | **Placebo**  **(n=109)** |
| --- | --- | --- |
| Age, median (Q1, Q3), years | 70 (67, 75) | 70 (67, 75) |
| Age category, n (%) |  |  |
| 60-64 | 18 (8·3) | 9 (8·3) |
| 65-74 | 139 (64·4) | 67 (61·5) |
| 75-84 | 58 (26·9) | 31 (28·4) |
| ≥85 | 1 (0·5) | 2 (1·8) |
| Sex,* n (%) |  |  |
| Female | 109 (50·5) | 46 (42·2) |
| Male | 107 (49·5) | 63 (57·8) |
| Region, n (%) |  |  |
| North America | 81 (37·5) | 45 (41·3) |
| Europe | 66 (30·6) | 37 (33·9) |
| Africa | 69 (31·9) | 27 (24·8) |
| Race,* n (%) |  |  |
| White | 157 (72·7) | 84 (77·1) |
| Black or African American | 38 (17·6) | 15 (13·8) |
| Multiple | 13 (6·0) | 6 (5·5) |
| Native Hawaiian or other Pacific Islander | 4 (1·9) | 1 (0·9) |
| American Indian or Alaska Native | 1 (0·5) | 0 |
| Asian | 1 (0·5) | 0 |
| Not reported | 0 | 1 (0·9) |
| Unknown | 2 (0·9) | 2 (1·8) |
| At increased risk of severe RSV disease, n (%) |  |  |
| No increased risk | 144 (66·7) | 66 (60·6) |
| Increased risk^†^ | 72 (33·3) | 43 (39·4) |
| Asthma | 19 (8·8) | 9 (8·3) |
| COPD | 18 (8·3) | 12 (11·0) |
| CHF | 4 (1·9) | 7 (6·4) |
| Other chronic heart diseases | 37 (17·1) | 18 (16·5) |
| Other chronic lung diseases^‡^ | 9 (4·2) | 2 (1·8) |

Ad26, adenovirus type 26; CHF, congestive heart failure; COPD, chronic obstructive pulmonary disease; preF, prefusion conformation-stabilised RSV F protein; Q, quartile; RSV, respiratory syncytial virus.

*Sex and race were self-reported.

^†^Participants at increased risk of severe RSV disease were defined as those with underlying chronic heart disease (CHF, coronary artery disease [eg, angina pectoris, ischemic cardiomyopathy, history of myocardial infarction, history of coronary artery bypass graft, or coronary artery stent]) and chronic lung diseases (eg, asthma and chronic COPD).

^‡^The electronic case report form included a category labelled “other chronic lung diseases” that could be chosen at the discretion of the investigator.

# Supplementary Table S9. Baseline characteristics of participants in the safety subset

|  | **Ad26.RSV.preF/**  **RSV preF protein**  **(n=2330)** | **Placebo**  **(n=1165)** |
| --- | --- | --- |
| Age, median (Q1, Q3), years | 70 (66, 75) | 70 (66, 75) |
| Age category, n (%) |  |  |
| 60-64 | 311 (13·3%) | 157 (13·5%) |
| 65-74 | 1353 (58·1%) | 678 (58·2%) |
| 75-84 | 632 (27·1%) | 314 (27·0%) |
| ≥85 | 34 (1·5%) | 16 (1·4%) |
| Sex,* n (%) |  |  |
| Female | 1097 (47·1%) | 541 (46·4%) |
| Male | 1233 (52·9%) | 624 (53·6%) |
| Region, n (%) |  |  |
| North America | 494 (21·2) | 247 (21·2) |
| Europe | 195 (8·4) | 93 (8·0) |
| Asia | 1442 (61·9%) | 728 (62·5%) |
| Africa | 153 (6·6) | 74 (6·4) |
| Australia/New Zealand | 37 (1·6) | 17 (1·5) |
| South America | 9 (0·4) | 6 (0·5) |
| Race,* n (%) |  |  |
| White | 712 (30·6) | 355 (30·5) |
| Asian | 1450 (62·2) | 730 (62·7%) |
| Black or African American | 97 (4·2) | 56 (4·8) |
| Multiple | 46 (2·0) | 16 (1·4) |
| Native Hawaiian or other Pacific Islander | 9 (0·4) | 3 (0·3) |
| American Indian or Alaska Native | 6 (0·3) | 1 (0·1) |
| Unknown | 6 (0·3) | 2 (0·2) |
| Not reported | 4 (0·2) | 2 (0·2) |
| At increased risk of severe RSV disease, n (%) |  |  |
| No increased risk | 1617 (69·4%) | 810 (69·5%) |
| Increased risk^†^ | 713 (30·6%) | 355 (30·5%) |
| Asthma | 153 (21·5%) | 64 (18·0%) |
| COPD | 191 (26·8%) | 89 (25·1%) |
| CHF | 47 (6·6%) | 25 (7·0%) |
| Other chronic heart diseases | 296 (41·5%) | 155 (43·7%) |
| Other chronic lung diseases^‡^ | 146 (20·5%) | 74 (20·8%) |

Ad26, adenovirus type 26; CHF, congestive heart failure; COPD, chronic obstructive pulmonary disease; preF, prefusion conformation-stabilised RSV F protein; Q, quartile; RSV, respiratory syncytial virus.

*Sex and race were self-reported.

^†^Participants at increased risk of severe RSV disease were defined as those with underlying chronic heart disease (CHF, coronary artery disease [eg, angina pectoris, ischemic cardiomyopathy, history of myocardial infarction, history of coronary artery bypass graft, or coronary artery stent]) and chronic lung diseases (eg, asthma and chronic COPD).

^‡^The electronic case report form included a category labelled “other chronic lung diseases” that could be chosen at the discretion of the investigator.

# Supplementary Table S10. Participants with at least one grade 3 or higher unsolicited adverse event until 28 days after vaccination (safety subset, final analysis)

| **System Organ Class**  **Preferred Term, n (%)** | **Ad26.RSV.preF/**  **RSV preF protein**  **(n=2330)** | **Placebo**  **(n=1165)** |
| --- | --- | --- |
| Participants with ≥1 unsolicited AE of at least grade 3 | 110 (4·7) | 34 (2·9) |
| Investigations | 81 (3·5) | 25 (2·1) |
| Blood pressure systolic increased | 68 (2·9) | 19 (1·6) |
| Blood pressure diastolic increased | 14 (0·6) | 4 (0·3) |
| Respiratory rate increased | 5 (0·2) | 5 (0·4) |
| Heart rate decreased | 2 (0·1) | 0 |
| Heart rate increased | 1 (<0·1) | 0 |
| Gastrointestinal disorders | 8 (0·3) | 0 |
| Vomiting | 2 (0·1) | 0 |
| Diarrhoea | 1 (<0·1) | 0 |
| Gastrointestinal haemorrhage | 1 (<0·1) | 0 |
| Inguinal hernia | 1 (<0·1) | 0 |
| Intestinal obstruction | 1 (<0·1) | 0 |
| Irritable bowel syndrome | 1 (<0·1) | 0 |
| Pancreatitis acute | 1 (<0·1) | 0 |
| Rectal polyp | 1 (<0·1) | 0 |
| Infections and infestations | 4 (0·2) | 2 (0·2) |
| Upper respiratory tract infection | 1 (<0·1) | 2 (0·2) |
| Pneumonia | 2 (0·1) | 0 |
| Bronchitis | 0 | 1 (0·1) |
| Erysipelas | 1 (<0·1) | 0 |
| Lower respiratory tract infection | 0 | 1 (0·1) |
| General disorders and administration site conditions | 5 (0·2) | 0 |
| Pain | 4 (0·2) | 0 |
| Pyrexia | 1 (<0·1) | 0 |
| Neoplasms benign, malignant and unspecified (incl cysts and polyps) | 3 (0·1) | 1 (0·1) |
| Basal cell carcinoma | 1 (<0·1) | 0 |
| Gastric cancer | 0 | 1 (0·1) |
| Lung neoplasm malignant | 1 (<0·1) | 0 |
| Metastatic renal cell carcinoma | 1 (<0·1) | 0 |
| Respiratory, thoracic and mediastinal disorders | 4 (0·2) | 0 |
| Asthma | 1 (<0·1) | 0 |
| Chronic obstructive pulmonary disease | 1 (<0·1) | 0 |
| Cough | 1 (<0·1) | 0 |
| Hydrothorax | 1 (<0·1) | 0 |
| Vascular disorders | 3 (0·1) | 1 (0·1) |
| Hypertension | 2 (0·1) | 0 |
| Aortic dissection | 0 | 1 (0·1) |
| Arteriosclerosis | 1 (<0·1) | 0 |
| Injury, poisoning and procedural complications | 2 (0·1) | 1 (0·1) |
| Bone contusion | 1 (<0·1) | 0 |
| Facial bones fracture | 0 | 1 (0·1) |
| Periprosthetic fracture | 1 (<0·1) | 0 |
| Subdural haemorrhage | 1 (<0·1) | 0 |
| Traumatic intracranial haematoma | 0 | 1 (0·1) |
| Musculoskeletal and connective tissue disorders | 2 (0·1) | 1 (0·1) |
| Myalgia | 1 (<0·1) | 0 |
| Neck pain | 1 (<0·1) | 0 |
| Osteoarthritis | 0 | 1 (0·1) |
| Psychiatric disorders | 2 (0·1) | 0 |
| Depression | 1 (<0·1) | 0 |
| Insomnia | 1 (<0·1) | 0 |
| Renal and urinary disorders | 0 | 2 (0·2) |
| Chronic kidney disease | 0 | 1 (0·1) |
| Renal haemorrhage | 0 | 1 (0·1) |
| Renal injury | 0 | 1 (0·1) |
| Cardiac disorders | 0 | 1 (0·1) |
| Atrial fibrillation | 0 | 1 (0·1) |
| Cardiac failure congestive | 0 | 1 (0·1) |
| Metabolism and nutrition disorders | 0 | 1 (0·1) |
| Decreased appetite | 0 | 1 (0·1) |
| Nervous system disorders | 1 (<0·1) | 0 |
| Cerebral haemorrhage | 1 (<0·1) | 0 |

AE, adverse event; Ad26, adenovirus type 26; preF, prefusion conformation-stabilised RSV F protein; RSV, respiratory syncytial virus.

Participants were counted only once within a period for any given event, regardless of the number of times they actually experienced the event in that period. AEs were coded using the Medical Dictionary for Regulatory Activities, Version 24·1. The number of participants from the safety subset was used as the denominator.

# Supplementary Table S11. Participants with at least one AE leading to death during the whole study by System Organ Class (FAS, final analysis)

| **System Organ Class, n (%)** | **Ad26.RSV.preF/**  **RSV preF protein**  **(n=16,709)** | **Placebo**  **(n=8367)** |
| --- | --- | --- |
| Participants with ≥1 AE leading to death | 105 (0·628) | 61 (0·729) |
| General disorders and administration site conditions | 24 (0·144) | 22 (0·263) |
| Cardiac disorders | 28 (0·168) | 10 (0·120) |
| Infections and infestations | 11 (0·066) | 11 (0·131) |
| Respiratory, thoracic and mediastinal disorders | 14 (0·084) | 7 (0·084) |
| Neoplasms benign, malignant and unspecified (incl cysts and polyps) | 12 (0·072) | 5 (0·060) |
| Nervous system disorders | 7 (0·042) | 9 (0·108) |
| Gastrointestinal disorders | 6 (0·036) | 0 |
| Respiratory, thoracic and mediastinal disorders | 14 (0·084) | 7 (0·084) |
| Injury, poisoning and procedural complications | 3 (0·018) | 2 (0·024) |
| Vascular disorders | 2 (0·012) | 2 (0·024) |
| Hepatobiliary disorders | 0 | 3 (0·036) |
| Psychiatric disorders | 2 (0·012) | 0 |
| Renal and urinary disorders | 0 | 2 (0·024) |
| Blood and lymphatic system disorders | 1 (0·006) | 0 |
| Metabolism and nutrition disorders | 1 (0·006) | 0 |
| Musculoskeletal and connective tissue disorders | 1 (0·006) | 0 |

AE, adverse event; Ad26, adenovirus type 26; FAS, full analysis set; preF, prefusion conformation-stabilised RSV F protein; RSV, respiratory syncytial virus; SOC, system organ class.

Participants were counted only once within a period for any given event, regardless of the number of times they actually experienced the event in that period. AEs were coded using the Medical Dictionary for Regulatory Activities, Version 24·1. The number of participants from the FAS was used as the denominator.

# Supplementary Table S12. Concomitant medications (major drug classes) reported after vaccination and by at least 0.5% of participants in either group (FAS-Safety subset, Final analysis)

|  | **Ad26.RSV.preF/**  **RSV preF protein** | **Placebo** |
| --- | --- | --- |
| **Full analysis set - Safety subset** | 1325 | 656 |
| Participants with one or more concomitant medications | 1052 (79·4%) | 504 (76·8%) |
| HMG COA reductase inhibitors | 473 (35·7%) | 244 (37·2%) |
| Platelet aggregation inhibitors excl. Heparin | 271 (20·5%) | 132 (20·1%) |
| Dihydropyridine derivatives | 243 (18·3%) | 113 (17·2%) |
| Proton pump inhibitors | 228 (17·2%) | 109 (16·6%) |
| Anilides | 216 (16·3%) | 76 (11·6%) |
| Ace inhibitors, plain | 199 (15·0%) | 96 (14·6%) |
| Beta blocking agents, selective | 183 (13·8%) | 105 (16·0%) |
| Angiotensin ii receptor blockers (arbs), plain | 171 (12·9%) | 108 (16·5%) |
| Biguanides | 166 (12·5%) | 91 (13·9%) |
| Selective beta-2-adrenoreceptor agonists | 136 (10·3%) | 62 (9·5%) |
| Sulfonamides, plain | 127 (9·6%) | 72 (11·0%) |
| Thyroid hormones | 119 (9·0%) | 70 (10·7%) |
| Thiazides, plain | 115 (8·7%) | 48 (7·3%) |
| Alpha-adrenoreceptor antagonists | 97 (7·3%) | 43 (6·6%) |
| Other analgesics and antipyretics | 87 (6·6%) | 31 (4·7%) |
| Selective serotonin reuptake inhibitors | 84 (6·3%) | 42 (6·4%) |
| Propionic acid derivatives | 83 (6·3%) | 38 (5·8%) |
| Other antidepressants | 81 (6·1%) | 33 (5·0%) |
| Glucocorticoids | 80 (6·0%) | 26 (4·0%) |
| Other viral vaccines | 78 (5·9%) | 32 (4·9%) |
| Adrenergics in combination with corticosteroids or other drugs, excl. anticholinergics | 78 (5·9%) | 30 (4·6%) |
| Vitamin D and analogues | 75 (5·7%) | 38 (5·8%) |
| Sulfonylureas | 65 (4·9%) | 32 (4·9%) |
| Piperazine derivatives | 60 (4·5%) | 21 (3·2%) |
| Influenza vaccines | 60 (4·5%) | 29 (4·4%) |
| Corticosteroids | 48 (3·6%) | 22 (3·4%) |
| Preparations inhibiting uric acid production | 47 (3·5%) | 26 (4·0%) |
| Leukotriene receptor antagonists | 47 (3·5%) | 20 (3·0%) |
| Other antihistamines for systemic use | 46 (3·5%) | 30 (4·6%) |
| Direct factor xa inhibitors | 44 (3·3%) | 27 (4·1%) |
| Benzodiazepine derivatives | 43 (3·2%) | 20 (3·0%) |
| Other opioids | 41 (3·1%) | 19 (2·9%) |
| Drugs for urinary frequency and incontinence | 40 (3·0%) | 22 (3·4%) |
| Alpha and beta blocking agents | 36 (2·7%) | 24 (3·7%) |
| Acetic acid derivatives and related substances | 36 (2·7%) | 10 (1·5%) |
| Potassium | 32 (2·4%) | 16 (2·4%) |
| Other lipid modifying agents | 32 (2·4%) | 16 (2·4%) |
| Anticholinergics | 31 (2·3%) | 8 (1·2%) |
| Other centrally acting agents | 29 (2·2%) | 9 (1·4%) |
| Testosterone-5-alpha reductase inhibitors | 28 (2·1%) | 24 (3·7%) |
| Adrenergics in combination with anticholinergics incl. Triple combinations with corticosteroids | 28 (2·1%) | 13 (2·0%) |
| Aldosterone antagonists | 27 (2·0%) | 13 (2·0%) |
| Other cardiac preparations | 26 (2·0%) | 10 (1·5%) |
| Angiotensin ii receptor blockers (arbs) and diuretics | 26 (2·0%) | 21 (3·2%) |
| Vitamin b12 (cyanocobalamin and analogues) | 24 (1·8%) | 7 (1·1%) |
| H2-receptor antagonists | 24 (1·8%) | 9 (1·4%) |
| Dipeptidyl peptidase 4 (dpp-4) inhibitors | 24 (1·8%) | 5 (0·8%) |
| Calcium | 23 (1·7%) | 12 (1·8%) |
| Benzodiazepine related drugs | 23 (1·7%) | 9 (1·4%) |
| Multivitamins, plain | 22 (1·7%) | 7 (1·1%) |
| Insulins and analogues for injection, long-acting | 22 (1·7%) | 11 (1·7%) |
| Bisphosphonates | 22 (1·7%) | 4 (0·6%) |
| Salicylic acid and derivatives | 21 (1·6%) | 5 (0·8%) |
| Organic nitrates | 21 (1·6%) | 14 (2·1%) |
| Magnesium | 21 (1·6%) | 5 (0·8%) |
| Other combinations of nutrients | 20 (1·5%) | 3 (0·5%) |
| Opioids in combination with non-opioid analgesics | 20 (1·5%) | 11 (1·7%) |
| Ascorbic acid (vitamin c), plain | 20 (1·5%) | 5 (0·8%) |
| Vitamin K antagonists | 19 (1·4%) | 9 (1·4%) |
| Other anxiolytics | 19 (1·4%) | 7 (1·1%) |
| Insulins and analogues for injection, fast-acting | 19 (1·4%) | 12 (1·8%) |
| Rauwolfia alkaloids and diuretics in combination | 18 (1·4%) | 12 (1·8%) |
| Oxicams | 18 (1·4%) | 17 (2·6%) |
| Other plain vitamin preparations | 18 (1·4%) | 4 (0·6%) |
| Other ophthalmologicals | 18 (1·4%) | 10 (1·5%) |
| Natural opium alkaloids | 17 (1·3%) | 8 (1·2%) |
| Natural and semisynthetic estrogens, plain | 17 (1·3%) | 10 (1·5%) |
| Drugs used in erectile dysfunction | 17 (1·3%) | 10 (1·5%) |
| Penicillins with extended spectrum | 16 (1·2%) | 5 (0·8%) |
| Fibrates | 16 (1·2%) | 12 (1·8%) |
| Dopamine agonists | 16 (1·2%) | 6 (0·9%) |
| Beta blocking agents, non-selective | 16 (1·2%) | 5 (0·8%) |
| Iron bivalent, oral preparations | 15 (1·1%) | 9 (1·4%) |
| Glucagon-like peptide-1 (glp-1) analogues | 15 (1·1%) | 9 (1·4%) |
| Ace inhibitors and diuretics | 15 (1·1%) | 8 (1·2%) |
| Sodium-glucose co-transporter 2 (sglt2) inhibitors | 14 (1·1%) | 11 (1·7%) |
| Calcium, combinations with vitamin D and/or other drugs | 14 (1·1%) | 2 (0·3%) |
| Benzothiazepine derivatives | 14 (1·1%) | 12 (1·8%) |
| Xanthines | 13 (1·0%) | 1 (0·2%) |
| Nucleosides and nucleotides excl. Reverse transcriptase inhibitors | 13 (1·0%) | 2 (0·3%) |
| Non-selective monoamine reuptake inhibitors | 13 (1·0%) | 1 (0·2%) |
| Coxibs | 13 (1·0%) | 3 (0·5%) |
| Azaspirodecanedione derivatives | 13 (1·0%) | 3 (0·5%) |
| Other antiinflammatory and antirheumatic agents, non-steroids | 12 (0·9%) | 1 (0·2%) |
| Ace inhibitors and calcium channel blockers | 12 (0·9%) | 5 (0·8%) |
| Prostaglandin analogues | 11 (0·8%) | 11 (1·7%) |
| Insulins and analogues for injection, intermediate-acting | 11 (0·8%) | 6 (0·9%) |
| Antiarrhythmics, class iii | 11 (0·8%) | 4 (0·6%) |
| Vitamin b-complex, plain | 10 (0·8%) | 3 (0·5%) |
| Tetracyclines | 10 (0·8%) | 3 (0·5%) |
| Selective serotonin (5ht1) agonists | 10 (0·8%) | 4 (0·6%) |
| Macrolides | 10 (0·8%) | 0 |
| Antiinflammatory preparations, non-steroids for topical use | 10 (0·8%) | 3 (0·5%) |
| 3-oxoandrosten (4) derivatives | 10 (0·8%) | 4 (0·6%) |
| Various alimentary tract and metabolism products | 9 (0·7%) | 2 (0·3%) |
| Softeners, emollients | 9 (0·7%) | 5 (0·8%) |
| Pyrazolones | 9 (0·7%) | 0 |
| Insulins and analogues for injection, intermediate- or long-acting combined with fast-acting | 9 (0·7%) | 3 (0·5%) |
| Beta blocking agents | 9 (0·7%) | 3 (0·5%) |
| Aminoalkyl ethers | 9 (0·7%) | 6 (0·9%) |
| Vitamins with minerals | 8 (0·6%) | 1 (0·2%) |
| Substituted alkylamines | 8 (0·6%) | 3 (0·5%) |
| Other antipsychotics | 8 (0·6%) | 1 (0·2%) |
| Other aminoglycosides | 8 (0·6%) | 3 (0·5%) |
| Lincosamides | 8 (0·6%) | 2 (0·3%) |
| Herbal antiinflammatory and antirheumatic remedies | 8 (0·6%) | 2 (0·3%) |
| Direct thrombin inhibitors | 8 (0·6%) | 5 (0·8%) |
| Combinations of penicillins, incl. Beta-lactamase inhibitors | 8 (0·6%) | 4 (0·6%) |
| Zinc | 7 (0·5%) | 2 (0·3%) |
| Multivitamins with minerals | 7 (0·5%) | 3 (0·5%) |
| Melatonin receptor agonists | 7 (0·5%) | 6 (0·9%) |
| Imidazoline receptor agonists | 7 (0·5%) | 6 (0·9%) |
| Expectorants | 7 (0·5%) | 3 (0·5%) |
| Contact laxatives | 7 (0·5%) | 3 (0·5%) |
| Antiallergic agents, excl. Corticosteroids | 7 (0·5%) | 2 (0·3%) |
| Synthetic anticholinergics, esters with tertiary amino group | 6 (0·5%) | 1 (0·2%) |
| Phenylalkylamine derivatives | 6 (0·5%) | 2 (0·3%) |
| Other psychostimulants and nootropics | 6 (0·5%) | 6 (0·9%) |
| Other nervous system drugs | 6 (0·5%) | 2 (0·3%) |
| Other drugs for peptic ulcer and gastro-oesophageal reflux disease (gord) | 6 (0·5%) | 4 (0·6%) |
| Other drugs affecting bone structure and mineralization | 6 (0·5%) | 1 (0·2%) |
| Other antimigraine preparations | 6 (0·5%) | 3 (0·5%) |
| Opium derivatives and expectorants | 6 (0·5%) | 2 (0·3%) |
| Mucolytics | 6 (0·5%) | 2 (0·3%) |
| Fluoroquinolones | 6 (0·5%) | 7 (1·1%) |
| Digitalis glycosides | 6 (0·5%) | 3 (0·5%) |
| Corticosteroids, very potent (group iv) | 6 (0·5%) | 2 (0·3%) |
| Corticosteroids, potent (group iii) | 6 (0·5%) | 4 (0·6%) |
| Combinations of vitamins | 6 (0·5%) | 2 (0·3%) |
| Sympathomimetics | 5 (0·4%) | 4 (0·6%) |
| Hydrazinophthalazine derivatives | 5 (0·4%) | 3 (0·5%) |
| Corticosteroids, moderately potent (group ii) | 5 (0·4%) | 6 (0·9%) |
| Combinations of oral blood glucose lowering drugs | 5 (0·4%) | 6 (0·9%) |
| Angiotensin ii receptor blockers (arbs), other combinations | 5 (0·4%) | 4 (0·6%) |
| Osmotically acting laxatives | 4 (0·3%) | 4 (0·6%) |
| Medical gases | 4 (0·3%) | 4 (0·6%) |
| Low-ceiling diuretics and potassium-sparing agents | 4 (0·3%) | 7 (1·1%) |
| Imidazole and triazole derivatives | 4 (0·3%) | 3 (0·5%) |
| Centrally acting sympathomimetics | 4 (0·3%) | 3 (0·5%) |
| Serotonin (5ht3) antagonists | 3 (0·2%) | 4 (0·6%) |
| Antiarrhythmics, class ic | 3 (0·2%) | 3 (0·5%) |
| Sympathomimetics in glaucoma therapy | 2 (0·2%) | 3 (0·5%) |
| Other cough suppressants | 1 (0·1%) | 3 (0·5%) |
| Uncoded | 42 (3·2%) | 18 (2·7%) |

FAS, full analysis set

# References

1. Shimabukuro T, CDC COVID-19 Vaccine Task Force. Thrombosis with thrombocytopenia syndrome (TTS) following Janssen COVID-19 vaccine. Advisory Committee on Immunization Practices (ACIP). 23 April 2021. <https://www.cdc.gov/vaccines/acip/meetings/slides-2021-04-23.html.> Accessed 5 April 2024.
1. CDC. Vaccine Adverse Event Reporting System (VAERS) standard operating procedures for COVID-19 (as of 29 January 2021). [https://www.cdc.gov/vaccinesafety/pdf/VAERS-v2-SOP.pdf. Accessed 5 April 2024](https://www.cdc.gov/vaccinesafety/pdf/VAERS-v2-SOP.pdf.%20Accessed%205%20April%202024).
2. DeMets DL, Lan KK. Interim analysis: the alpha spending function approach*. Stat Med*. 1994;**13**(13-14):1341-52.
